# The *Cycas* genome and the early evolution of seed plants

**DOI:** 10.1038/s41477-022-01129-7

**Published:** 2022-04-18

**Authors:** Yang Liu, Sibo Wang, Linzhou Li, Ting Yang, Shanshan Dong, Tong Wei, Shengdan Wu, Yongbo Liu, Yiqing Gong, Xiuyan Feng, Jianchao Ma, Guanxiao Chang, Jinling Huang, Yong Yang, Hongli Wang, Min Liu, Yan Xu, Hongping Liang, Jin Yu, Yuqing Cai, Zhaowu Zhang, Yannan Fan, Weixue Mu, Sunil Kumar Sahu, Shuchun Liu, Xiaoan Lang, Leilei Yang, Na Li, Sadaf Habib, Yongqiong Yang, Anders J. Lindstrom, Pei Liang, Bernard Goffinet, Sumaira Zaman, Jill L. Wegrzyn, Dexiang Li, Jian Liu, Jie Cui, Eva C. Sonnenschein, Xiaobo Wang, Jue Ruan, Jia-Yu Xue, Zhu-Qing Shao, Chi Song, Guangyi Fan, Zhen Li, Liangsheng Zhang, Jianquan Liu, Zhong-Jian Liu, Yuannian Jiao, Xiao-Quan Wang, Hong Wu, Ertao Wang, Michael Lisby, Huanming Yang, Jian Wang, Xin Liu, Xun Xu, Nan Li, Pamela S. Soltis, Yves Van de Peer, Douglas E. Soltis, Xun Gong, Huan Liu, Shouzhou Zhang

**Affiliations:** 1 State Key Laboratory of Agricultural Genomics, BGI-Shenzhen, Shenzhen, China; 2 Key Laboratory of Southern Subtropical Plant Diversity, Fairy Lake Botanical Garden, Shenzhen & Chinese Academy of Sciences, Shenzhen, China; 3 State Key Laboratory of Grassland Agro-Ecosystems, College of Ecology, Lanzhou University, Lanzhou, China; 4 State Environmental Protection Key Laboratory of Regional Eco-process and Function Assessment, Chinese Research Academy of Environmental Sciences, Beijing, China; 5 Key Laboratory for Plant Diversity and Biogeography of East Asia, Kunming Institute of Botany, Chinese Academy of Sciences, Kunming, China; 6 Key Laboratory of Plant Stress Biology, State Key Laboratory of Crop Stress Adaptation and Improvement, Henan University, Kaifeng, China; 7 Department of Biology, East Carolina University, Greenville, NC USA; 8 College of Biology and Environment, Nanjing Forestry University, Nanjing, China; 9 College of Life Sciences, University of Chinese Academy of Sciences, Beijing, China; 10 Nanning Botanical Garden, Nanning, China; 11 School of Life Sciences, Sun Yat-sen University, Guangzhou, China; 12 Sichuan Cycas panzhihuaensis National Nature Reserve, Panzhihua, China; 13 Global Biodiversity Conservancy, Chonburi, Thailand; 14 Department of Entomology, China Agricultural University, Beijing, China; 15 Department of Ecology and Evolutionary Biology, University of Connecticut, Storrs, CT USA; 16 Guangdong Provincial Key Laboratory for Plant Epigenetics, Longhua Institute of Innovative Biotechnology, College of Life Sciences and Oceanography, Shenzhen University, Shenzhen, China; 17 Department of Biotechnology and Biomedicine, Technical University of Denmark, Lyngby, Denmark; 18 Shenzhen Agricultural Genome Research Institute, Chinese Academy of Agricultural Sciences, Shenzhen, China; 19 College of Horticulture, Academy for Advanced Interdisciplinary Studies, Nanjing Agricultural University, Nanjing, China; 20 State Key Laboratory of Pharmaceutical Biotechnology, School of Life Sciences, Nanjing University, Nanjing, China; 21 Chengdu University of Traditional Chinese Medicine, Chengdu, China; 22 Department of Plant Biotechnology and Bioinformatics, Ghent University, VIB UGent Center for Plant Systems Biology, Gent, Belgium; 23 College of Agriculture and Biotechnology, Zhejiang University, Hangzhou, China; 24 Hainan Institute of Zhejiang University, Sanya, China; 25 The College of Life Sciences, Sichuan University, Chengdu, China; 26 Key Laboratory of Orchid Conservation and Utilization of National Forestry and Grassland Administration at College of Landscape Architecture, Fujian Agriculture and Forestry University, Fuzhou, China; 27 State Key Laboratory of Systematic and Evolutionary Botany, Institute of Botany, Chinese Academy of Sciences, Beijing, China; 28 College of Life Sciences, South China Agricultural University, Guangzhou, China; 29 National Key Laboratory of Plant Molecular Genetics, Chinese Academy of Sciences Center for Excellence in Molecular Plant Sciences, Institute of Plant Physiology and Ecology, Chinese Academy of Sciences, Shanghai, China; 30 Department of Biology, University of Copenhagen, Copenhagen, Denmark; 31 Florida Museum of Natural History, University of Florida, Gainesville, FL USA; 32 Department of Biochemistry, Genetics and Microbiology, University of Pretoria, Pretoria, South Africa; 33 Department of Biology, University of Florida, Gainesville, FL USA

**Keywords:** Plant sciences, Evolution

## Abstract

Cycads represent one of the most ancient lineages of living seed plants. Identifying genomic features uniquely shared by cycads and other extant seed plants, but not non-seed-producing plants, may shed light on the origin of key innovations, as well as the early diversification of seed plants. Here, we report the 10.5-Gb reference genome of *Cycas panzhihuaensis*, complemented by the transcriptomes of 339 cycad species. Nuclear and plastid phylogenomic analyses strongly suggest that cycads and *Ginkgo* form a clade sister to all other living gymnosperms, in contrast to mitochondrial data, which place cycads alone in this position. We found evidence for an ancient whole-genome duplication in the common ancestor of extant gymnosperms. The *Cycas* genome contains four homologues of the *fitD* gene family that were likely acquired via horizontal gene transfer from fungi, and these genes confer herbivore resistance in cycads. The male-specific region of the Y chromosome of *C. panzhihuaensis* contains a MADS-box transcription factor expressed exclusively in male cones that is similar to a system reported in *Ginkgo*, suggesting that a sex determination mechanism controlled by MADS-box genes may have originated in the common ancestor of cycads and *Ginkgo*. The *C. panzhihuaensis* genome provides an important new resource of broad utility for biologists.

## Abstract (web summary)

The study assembled a chromosome-level genome of *Cycas panzhihuaensis*, the last major lineage of seed plants for which a high-quality genome assembly was lacking. The study closes an important gap in our understanding of genome structure and evolution in seed plants.

## Main

**Fig. 1:**
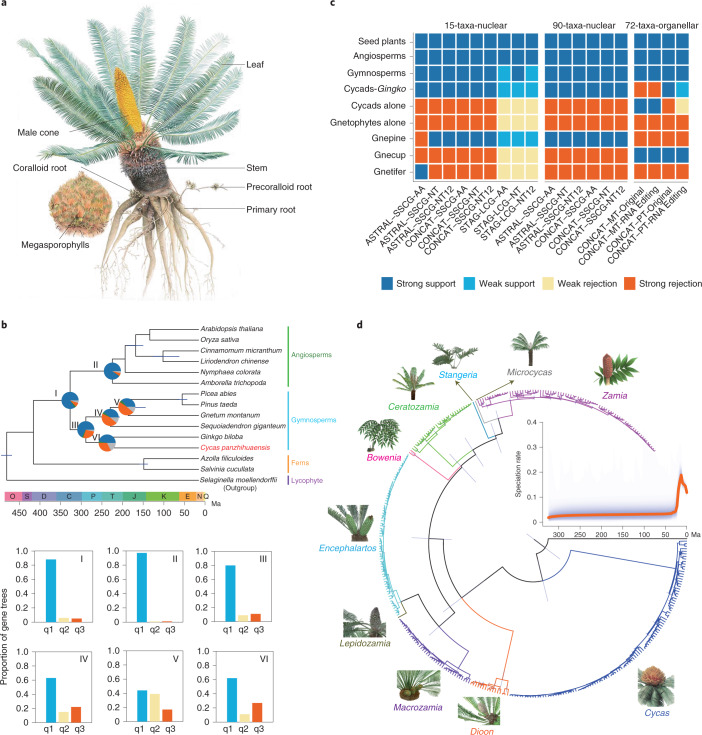
Phylogenomic analyses of cycads and seed plants. **a**, Illustration of *Cycas panzhihuaensis*. **b**, Chronogram of seed plants on the basis of the SSCG-NT12 dataset inferred using MCMCTree. All branches are maximally supported by bootstrap values (ML) and posterior probabilities (ASTRAL). I, II, III, VI, V and VI indicate internal branches for which the pie charts depicting gene tree incongruence are complemented by histograms (lower panel) showing quartet support for the main topology (q1), the first alternative topology (q2) and the second alternative topology (q3). O, Ordovician; S, Silurian; D, Devonian; C, Carboniferous; P, Permian; T, Triassic; J, Jurassic; K, Cretaceous; Pg, Palaeogene; N, Neogene; Q, Quaternary; Ma, million years ago. **c**, DiscoVista species tree analysis: rows correspond to the nine hypothetical groups tested (see Supplementary Note 5 for details) and columns correspond to the results derived from the use of different datasets and methods. SSCG, single-copy genes; LCG, low-copy genes; MT, mitochondrial genes; PT, plastid genes; AA, amino acid sequences; NT, nucleotide sequences; NT12, codon 1st + 2nd positions; ASTRAL, coalescent tree inference method using ASTRAL; CONCAT, maximum likelihood tree inferred with IQ-TREE based on concatenated datasets; STAG, species tree inference using software STAG with low-copy genes (one to four copies); Original, original organellar nucleotide sequences; RNA Editing, organellar genes with RNA editing site modified. Strong support, the clade is reconstructed with a support value >95%. Weak support, the clade is reconstructed with support value <95%. Weak rejection, the clade is not recovered, but the alternative topology is not conflict if poorly supported branches (<85%) are collapsed. Strong rejection, the clade is not recovered, and the alternative topology is conflict even when poorly supported branches (<85%) are collapsed. **d**, Diversification of Cycadales. The chronogram of 339 cycad species was inferred with MCMCTree based on 100 nuclear single-copy genes with concordant evolutionary histories. All illustrations are specifically created for this study (a high-resolution version is available at https://db.cngb.org/codeplot/datasets/public_dataset?id=PwRftGHfPs5qG3gE).

Cycads are often referred to as ‘living fossils’; they originated in the mid-Permian and dominated terrestrial ecosystems during the Mesozoic, a period called the ‘age of cycads and dinosaurs’^1^. Although the major cycad lineages are ancient, modern cycad species emerged from several relatively recent diversifications^2,3^. Cycads are long-lived woody plants that, unlike other extant gymnosperms, bear frond-like leaves clustered at the tip of the stem^4^. Extant cycads comprise 10 genera and approximately 360 species, two-thirds of which are on the International Union for Conservation of Nature Red List of threatened species^5^. All living cycad species are dioecious, with individual plants developing either male or female cones (except in *Cycas*, which produces a loose cluster of megasporophylls rather than a true female cone; Fig. 1a)^6^. Unlike other extant seed plants, cycads and *Ginkgo* retain flagellated sperm, an ancestral trait shared with bryophytes, lycophytes and ferns^7^. Cycads exhibit other special features, such as the accumulation of toxins that deter herbivores^8^ in seeds and vegetative tissues. They also produce coralloid roots that host symbiotic cyanobacteria, making them the only gymnosperm associated with nitrogen-fixing symbionts^9^. The origin of the seed marked one of the most important events of plant evolution^10^. As one of the four extant gymnosperm groups (cycads, *Ginkgo*, conifers and gnetophytes), cycads hold an important evolutionary position for understanding the origin and early evolution of seed plants. We therefore generated a high-quality genome assembly for a species of *Cyca*s to explore fundamental questions in seed plant evolution, including the phylogenetic position of cycads, the occurrence of ancient whole-genome duplications (WGDs), innovation in gene function and the evolution of sex determination.

## A chromosome-scale genome assembly

**Extended Data Fig. 1:**
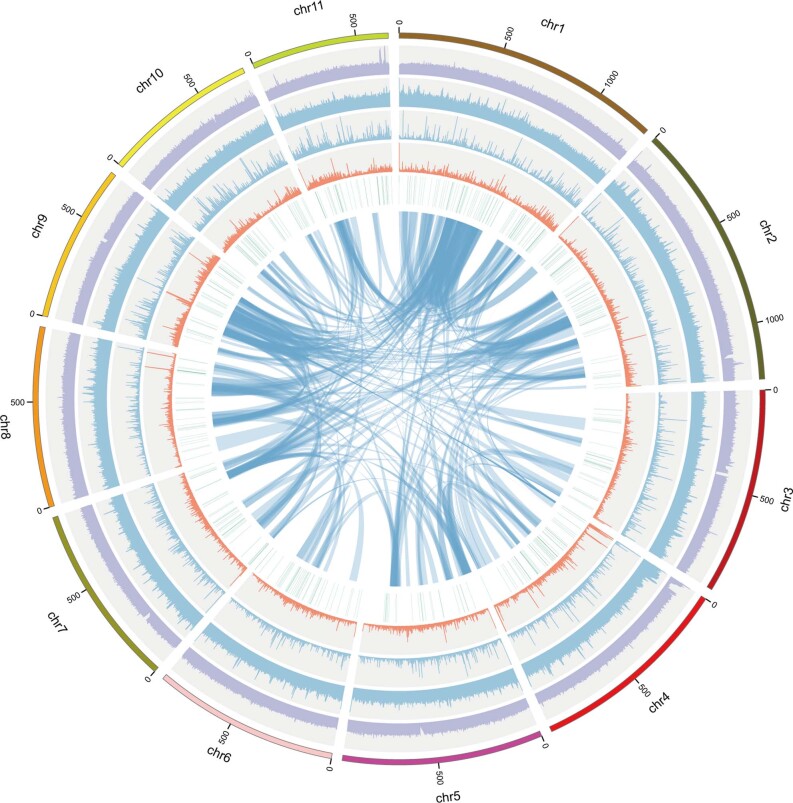
Genome features of *C. panzhihuaensis*. Outer ring: The 11 chromosomes are labeled from Chr1 to Chr11. Inner rings 1-4 (from outside to inside): Repeat elements number shown in light purple. GC content colored indicated in light blue (y-axis min-max: 0.27–0.48). Expressed base percentage colored in light blue (y-axis min-max: 0–0.20). Gene numbers colored in light orange (y-axis min-max: 0-30). The sliding window of the inner rings 1-4 is 1 Mb. The inner ring 5 indicates the miRNA location over the genome. The blue lines inside represent the syntenic regions in *Cycas*.

Here, we report a high-quality, chromosome-level genome assembly of *Cycas panzhihuaensis* based on sequencing of the haploid megagametophyte using a combination of MGI-SEQ short-read, Oxford Nanopore long-read and Hi-C sequencing methods (Supplementary Note 2). The genome comprises 10.5 Gb assembled in 5,123 contigs (N50 = 12 Mb), with 95.3% of these contigs anchored to the largest 11 pseudomolecules, corresponding to the 11 chromosomes (*n* = 11) of the *C. panzhihuaensis* karyotype^11^ (Supplementary Note 3 and Extended Data Fig. 1). The annotated genome describes 32,353 protein-coding genes and is mostly composed of repetitive elements adding up to 7.8 Gb (Supplementary Note 4). Based on BUSCO^12^ estimation, the gene space completeness of the *C. panzhihuaensis* genome assembly is 91.6% (Supplementary Note 4).

**Extended Data Fig. 2:**
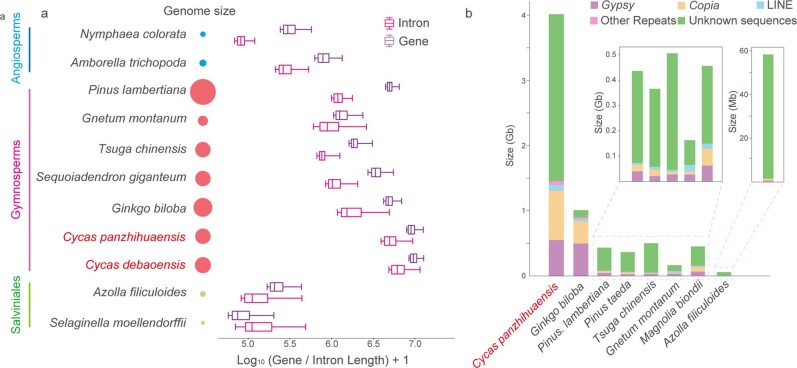
Comparative analysis of *C. panzhihuaensis*. Extended Data Fig. 2. Comparative analysis of C. panzhihuaensis. (**a**) Comparison of the longest 10% of introns and gene in the representative land plants. The minimum, first quartile (Q1), median, third quartile (Q3), and maximum value was indicated in the box-plot by order after excluding the outliers. (**b**) Comparison of components of intron across the selected plants.

Compared with other gymnosperms, the size of the *Cycas* genome is similar to that of *Ginkgo* (10.6 Gb)^13,14^ and intermediate between the relatively compact genome of *Gnetum* (4.1 Gb)^15^ and the very large genomes of conifers (for example, ~20-Gb genomes of *Picea* and *Pinus*)^16–18^. As in other gymnosperm genomes, a large portion (76.14%) of the *C. panzhihuaensis* genome consists of ancient repetitive elements (Supplementary Note 4). In addition, the genome contains almost equal proportions of *copia* and *gypsy* long terminal repeat (LTR) elements, in contrast to other gymnosperm genomes, in which *gypsy* repeats are more frequent^14,15^ (Supplementary Note 6). Among all sequenced plant genomes, *C. panzhihuaensis* has the longest average introns (~30.8 kb) and genes (~121.3 kb) (Extended Data Fig. 2a), surpassing those of *Ginkgo*^14^. In comparison with *Ginkgo*, in which LTRs dominate intron content, the introns of *C. panzhihuaensis* contain a large portion of unknown sequences (Extended Data Fig. 2b). The longest gene, *CYCAS_013063*, encoding a kinesin-like protein KIF3A, covers 2.1 Mb in the *C. panzhihuaensis* genome; the longest intron is approximately 1.5 Mb and was detected in *CYCAS_030563*, a gene that encodes a photosystem II CP43 reaction centre protein. Both genes are expressed, as evidenced by our long-read transcriptome data.

## Phylogeny of cycads and seed plants

**Extended Data Fig. 3:**
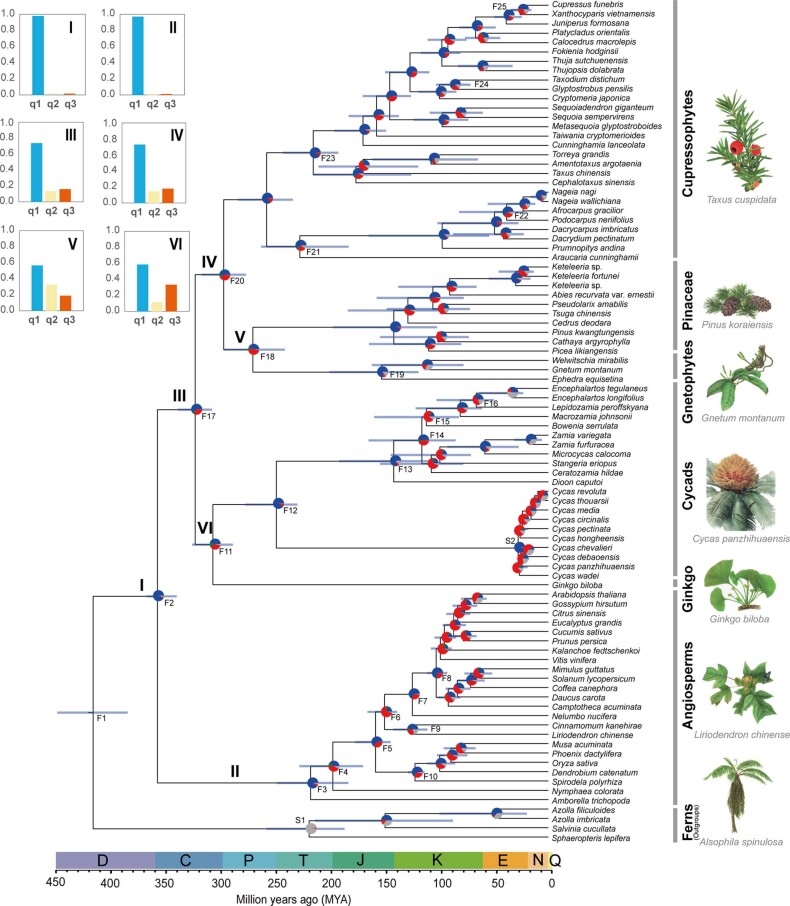
The chronogram of 90 vascular plant species inferred with MCMCTree based on 100. nuclear single copy genes with concordant evolutionary histories. 25 fossil calibrations and 2 secondary calibrations were used. Individual gene trees (1,569 NT tree) were mapped on the nuclear coalescent tree with Phyparts. The pie charts at each node show the proportion of genes in concordance (blue), conflict (green = a single dominant alternative; red = all other conflicting trees), and without enough information (gray). Quartet support for six internal branches I, II, III, IV, V, VI were indicated on the left panel as barcharts. Image courtesy of Zanqian Li and Xiaolian Zeng.

The *C. panzhihuaensis* genome provides an opportunity to revisit the long-standing debate on the evolutionary relationships among living seed plants. On the basis of molecular phylogenetic analyses, extant gymnosperms are resolved as a monophyletic group, but the branching order among their major lineages has remained controversial^19–23^. Our phylogenetic analyses of separate nuclear (Fig. 1b, Extended Data Fig. 3 and Supplementary Note 5) and plastid datasets strongly support cycads plus *Ginkgo* as sister to the remaining extant gymnosperms, in agreement with several other analyses^23,24^, whereas mitochondrial data resolve cycads alone in that position (Fig. 1c). This conflict arising from the mitochondrial data cannot be explained by the presence of extensive RNA editing sites in the mitochondrial data (Fig. 1c), which in some cases has been reported to bias phylogenetic inferences^25,26^, and instead may be best explained by incomplete lineage sorting, which is supported by our PhyloNet^27^ and coalescent analyses of nuclear genes (Supplementary Note 5).

The extant diversity of cycads was previously considered to have arisen synchronously within the past 9–50 million years (Myr)^2,3^. Our inferences, based on 1,170 low-copy nuclear genes sampled for 339 cycad species and 6 fossil calibrations^3^ corroborate recent broad analyses of gymnosperms indicating that extant species-rich cycad genera emerged from rapid radiations ranging from 11 to 20 Myr ago, which may have been a consequence of dramatic Miocene global temperature changes^24,28^. Notably, major temperate and tropical radiations in several major clades of flowering plants have been shown to be associated with Miocene cooling in the past 15 Myr (refs. ^29–31^).

## *Cycas* is an ancient polyploid

**Fig. 2:**
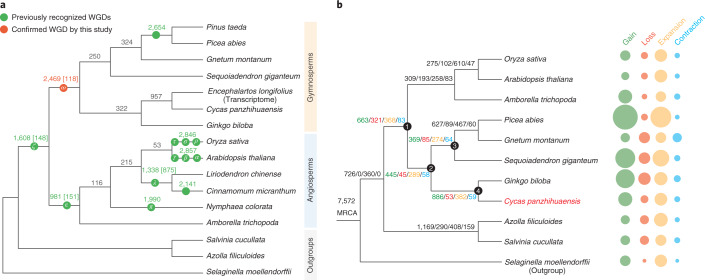
Ancient polyploidy events and evolution of gene families in seed plants. **a**, Inference of the number of gene families with duplicated genes surviving after WGD events mapped on a phylogenetic tree depicting the relationships among 16 vascular plants included in this study. The number of gene families with retained gene duplicates reconciled on a particular branch of the species tree are shown above the branch across the phylogeny (Methods). Numbers in square brackets denote the number of gene families with duplicated genes also supported by synteny evidence. **b**, Evolutionary analyses and phylogenetic profiles depicting the gains (light green), losses (light red), expansions (light yellow) and contractions (light blue) of orthogroups, according to the reconstruction of the ancestral gene content at key nodes and the dynamic changes of the lineage-specific gene characteristics.

**Extended Data Fig. 4:**
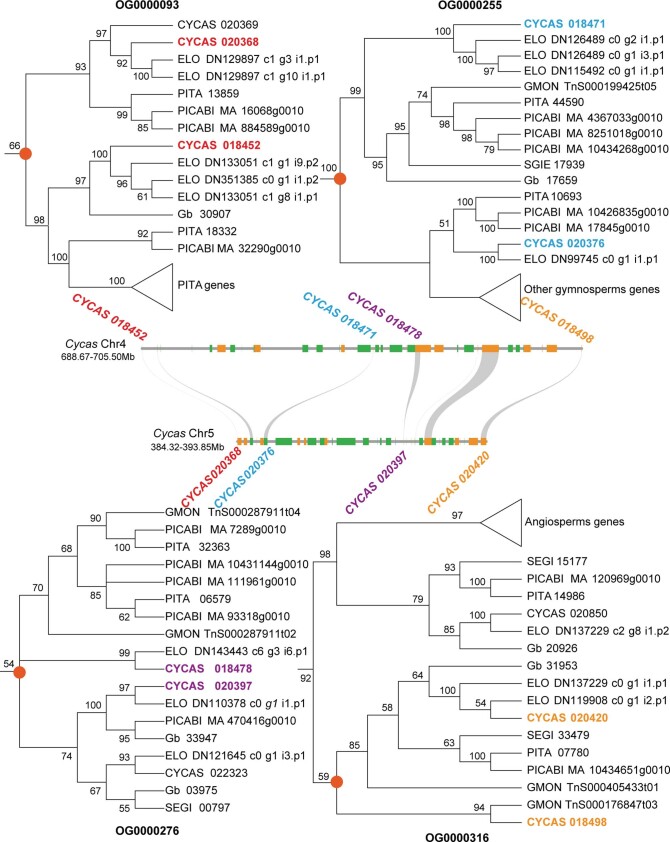
Ancestral polyploidy events in extant gymnosperms. Example showing both the phylogenomic and syntenic evidence supporting an ancestral polyploidy event in extant gymnosperms. Four pairs of paralogous genes in OG0000093, OG0000255, OG00000276 and OG0000316 were duplicated before the divergence of gymnosperms and after the split of angiosperms and gymnosperms based on phylogenetic trees. These pairs of duplicated genes are located on the same syntenic block identified in the *C. panzhihuaensis* genome. The abbreviated name given before the protein ID represents species name: CYCAS: *Cycas panzhihuaensis*, Gb: *Ginkgo biloba*, ELO: *Encephalartos longifolius*, SEGI: *Sequoiadendron giganteum*, GMON: *Gnetum montanum*, PICABI: *Picea abies*, PITA: *Pinus taeda*.

WGD is a major driving force in the evolution of land plants and has dramatically promoted the diversification of flowering plants^23,32^. Synonymous substitutions per synonymous site (*K*_*S*_) analysis of duplicate genes^33^ revealed a clear peak at similar *K*_*S*_ values (~0.85, range 0.5–1.2) for both *Cycas* and *Ginkgo*, suggestive of an ancient WGD possibly shared by these two lineages (Supplementary Note 7)^34^. However, the precise evolutionary position of this WGD event remains ambiguous. Our phylogenomic analyses based on 15 genomes and 1 transcriptome revealed 2,469 gymnosperm-wide duplications in 9,545 gene families and indicate that this WGD event dates to the most recent common ancestor (MRCA) of extant gymnosperms (Fig. 2a), supporting recent findings based on transcriptome data^24^. We also identified 69 ancient syntenic genomic segments that further support a gymnosperm-wide WGD (Extended Data Fig. 3, Supplementary Fig. 23 and Supplementary Tables 24 and 25). Furthermore, a mixed dataset with increased sampling—29 genomes and 61 transcriptomes—also yielded the same result (Fig. 2a and Extended Data Fig. 4). This gymnosperm-wide WGD, here named omega (ω), is independent of the WGD preceding the split between gymnosperms and angiosperms^35^ and may have contributed to the subsequent evolution of gymnosperm-specific genes involved in plant hormone signal transduction, biosynthesis of secondary metabolites, plant–pathogen interaction and terpenoid biosynthesis (Supplementary Note 7).

## Ancestral gene innovation in the origin of the seed

The origin of seed plants is marked by the emergence of key traits including the seed, pollen and secondary growth of xylem and phloem^36^. Reconstruction of the evolution of gene families across the seed plant tree of life revealed that 663 orthogroups were gained and 368 expanded in the MRCA of extant seed plants compared with non-seed plants (Fig. 2b, node 1). Among these, 106 of the new orthogroups and 55 of the expanded orthogroups are associated with seed development in *Arabidopsis*^37^, including the regulation of development during early embryogenesis, seed dormancy and germination, and seed coat formation, as well as in immunity and stress response of the seed (Supplementary Note 6).

**Extended Data Fig. 5:**
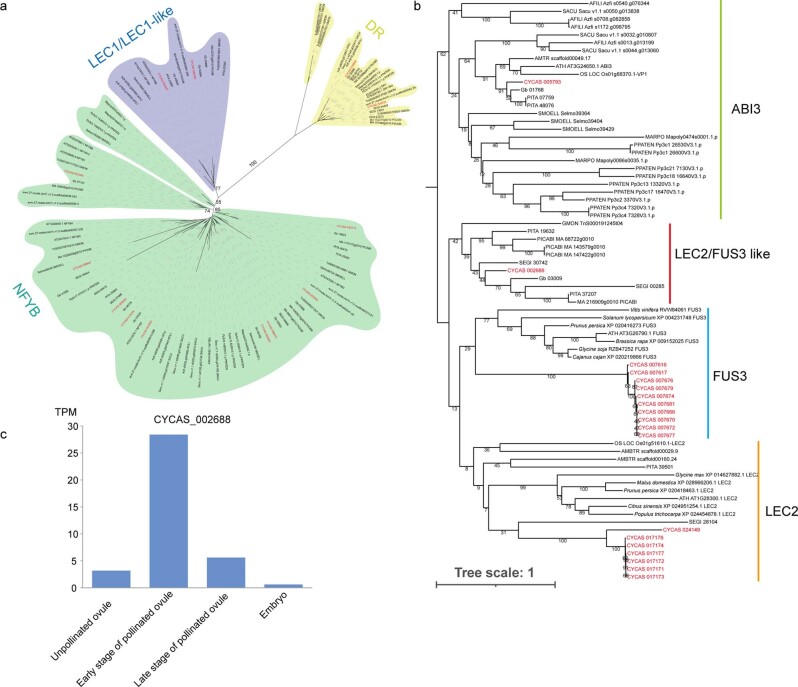
The phylogeny of LAFL(NF-YB, ABI3, FUS3, and LEC2) transcriptional regulators. (**a**) Phylogenetic tree of the NF-YB. The tree was constructed using the maximum likelihood method with 500 bootstrap replicates. The bootstrap values are shown on the branches. (**b**) Phylogenetic tree of the B3 domain containing the gene family of *C. panzhihuaensis*. Bootstrap values are shown on the braches. (**c**) Transcript expression level is indicated by TPM during seed development. The phylogenetic trees were built using RAxML (estimating branch support values by bootstrap iterations with 500 replicates) with PROTGAMMAGTRX amino acid substitution model. The abbreviated name given before the protein ID represents species name: CYCAS: *Cycas panzhihuaensis*, Gb: *Ginkgo biloba*, SEGI: *Sequoiadendron giganteum*, GMON: *Gnetum montanum*, PICABI: *Picea abies*, PITA: *Pinus taeda*, ATH, *Arabidopsis thaliana*, DEBAO: *Cycas debaoensis*, AMTR: *Amborella trichopoda*, OS: *Oryza sativa*, AFILI: *Azolla filiculoides*, SACU: *Salvinia cucullata*, SELMO: *Selaginella moellendorffii*, PPATEH: *Physcomitrella patens*, MARPO: *Marchantia polymorpha*.

Genes of the LAFL family are well-known as core regulatory genes of seed development, including *LEAFY COTYLEDON1* (*LEC1*), *ABSCISIC ACID INSENSITIVE3* (*ABI3*), *LEAFY COTYLEDON2* (*LEC2*) and *FUSCA3* (*FUS3*), which encode master transcriptional regulators, interacting to form complexes that control embryo development and maturation^38^. *LEC1* genes are found only in vascular plants, but *ABI3* is widely distributed in embryophytes (Supplementary Note 10.6). *Cycas* and *Ginkgo* each contain a small number of *LEC1* (two and three in each, respectively) and *ABI3* (one in each) genes, whereas *C. panzhihuaensis* encodes a burst of *FUS3* (ten) and *LEC2* (seven) genes in the form of tandem repeats. *FUS3* and *LEC2* are shared by all living seed plants; the *Cycas* and other gymnosperm genomes contain genes composing a new clade of B3 domain proteins, that is, the *FUS3*/*LEC2*-like clade, which is sister to the clade of *FUS3* and *LEC2* (Extended Data Fig. 5). The *FUS3*/*LEC2*-like families are unique to gymnosperms, show significant expression after pollination in *C. panzhihuaensis* (Extended Data Fig. 5c) and may play specific roles in initiating embryogenesis in gymnosperms.

## Regulation of seed development in *Cycas*

**Fig. 3:**
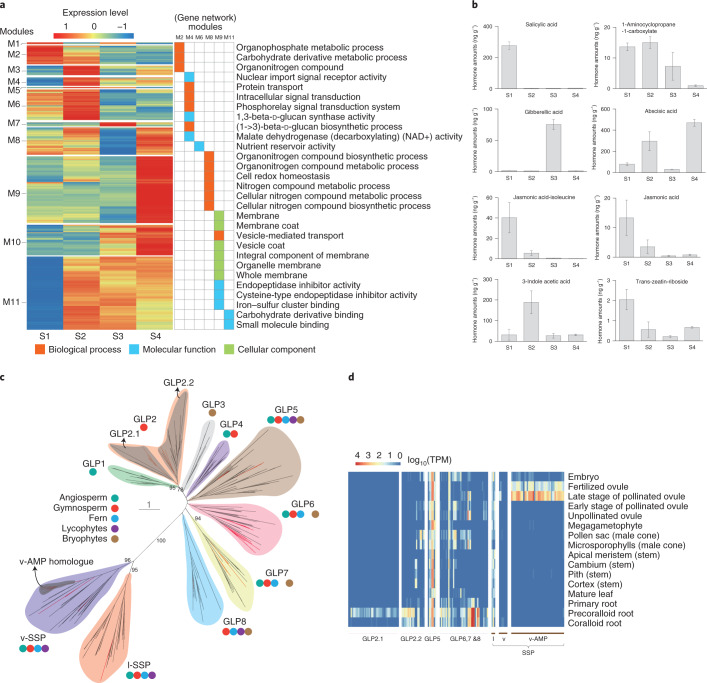
Gene expression and phytohormone synthesis at different developmental stages of the seed of *Cycas* and the evolution of seed storage proteins. **a**, Heatmap showing relative expression of genes in 11 co-expression modules by WGCNA across 4 developmental stages of the seed: S1, unpollinated ovule; S2, early stage of pollinated ovule; S3, late stage of pollinated ovule; and S4, fertilized ovule. **b**, Quantification of eight plant phytohormone amounts in the same four developmental stages of the *Cycas* seed as above. The grey histogram represents the amount of hormone (*n* = 2 biologically independent experiments) and the error bar represents the standard error. **c**, Phylogeny of SSPs in some representative species in land plants. The SSPs analysed include germin-like protein (GLP), legumin-like SSP (l-SSP), vicilin-like SSP (v-SSP) and v-AMP. A maximum likelihood tree with 500 bootstrap replicates was constructed using RAxML. Bootstrap values (≥50%) for each major clade (highlighted in colour) and the relationships among them are provided. The *Cycas* sequences are highlighted in red. **d**, Expression levels of SSP in different tissues of *C. panzhihuaensis*.

To better understand the dynamic changes in gene regulation and regulatory programmes during ovule pollination and fertilization, we performed a weighted correlation network analysis (WGCNA) and identified 11 co-expression modules at different developmental stages of the *C. panzhihuaensis* ovule and seed (Fig. 3a). The modules are enriched in seed nutrition metabolic processes (M2, M6 and M8), membrane biosynthesis (M9, which may relate to the development of the integument) and genes synthesizing callose, a major component of the pollen tube (M4) (Supplementary Note 10). A survey of phytohormones showed that salicylic acid and jasmonic acid, which are both involved in pathogen resistance, were produced at higher levels in unpollinated ovules versus post-pollinated ovules (Fig. 3b), and genes involved in the biosynthesis of these two phytohormones were also more highly expressed in unpollinated ovules, indicating the higher demand for these hormones as agents of pathogen resistance in the unpollinated ovule. Gibberellin, which is reported to regulate integument development in the ovules of flowering plants^39^, accumulated in the late stage of the pollinated ovule in *Cycas*. We also found gene families related to integument development (for example, those involved in cutin, suberine and wax biosynthesis), with increased expression levels at the late stage of the pollinated ovule. Fertilized ovules accumulated a high level of abscisic acid and expressed the genes related to cell wall organization and biogenesis, indicating their activity in embryo development, seed coat formation, and seed maturation and dormancy^40^ (Supplementary Note 10.1–10.5).

Among genes related to seed development, the most notable is the cupin protein family, expanded in *C. panzhihuaensis* compared with all other green plants. Phylogenetic analysis revealed that the cupin family can be subdivided into two groups: the germin-like and seed storage protein (SSP)-encoding genes. Surprisingly, we identified a new type of gene encoding vicilin-like storage proteins in *C. panzhihuaensis*; this type appears to be homologous to the vicilin-like antimicrobial peptides (v-AMP) and is organized as a tandem gene array in the *C. panzhihuaensis* genome (Fig. 3c). These v-AMP homologues are mostly expressed in *C. panzhihuaensis* at the late stage of pollinated ovules and fertilized ovules, with expression gradually decreasing during embryogenesis, suggesting the potentially important role of v-AMP genes in seed development (Fig. 3d and Supplementary Note 10.6).

## Secondary growth and cell wall synthesis

Secondary growth is also a major innovation of seed plants^36^, and it has been recognized from fossils of now-extinct progymnosperms, which predated the origin of seed plants^36,41^. Secondary phloem and xylem are produced by the activity of a bifacial vascular cambium (secondary meristem). We found that several genes that are known in angiosperms to regulate secondary growth in the positioning of the xylem, or in xylem/phloem patterning, underwent obvious expansions in the MRCA of extant seed plants compared with non-seed plants, including the MYB family member *ALTERED PHLOEM DEVELOPMENT* (*APL*), *WOL* and *BRASSINOSTEROID-INSENSITIVE LIKE 1* (*BRL1*) and *BRL3*. The *APL* gene is expressed in the phloem and cambium in vascular plants, and its encoded protein promotes phloem differentiation^42^. The expression of *APL* is regulated by *WOL* in the procambium^43^. The *BRL1* and *BRL3* genes encode brassinosteroid receptors that play major roles in xylem differentiation and phloem/xylem patterning in angiosperms^44^. Many copies of these genes were found to be highly expressed in cambium or apical meristem of *C. panzhihuaensis* (Supplementary Note 6).

**Extended Data Fig. 6:**
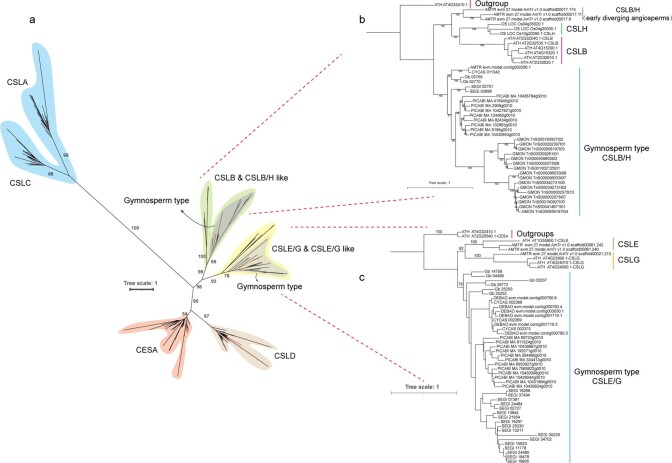
Phylogenetic tree of CESA/CSL gene families. (**a**) Phylogenetic trees of CESA and CSL gene families. (**b**) Phylogenetic tree of CSLB and CSLH genes. (**c**) The phylogenetic tree of CSLE and CSLG genes. The CSLE/G from gymnosperm are the ancestral form of the angiosperm CSLE and CSLG. The phylogenetic trees were generated using RAxML with PROTCATGTR model and 500 bootstrap replicates. Bootstrap values ≥ 50% are shown. The abbreviated name given before the protein ID represents species name: CYCAS: *Cycas panzhihuaensis*, Gb: *Ginkgo biloba*, SEGI: *Sequoiadendron giganteum*, GMON: *Gnetum montanum*, PICABI: *Picea abies*, PITA: *Pinus taeda*, ATH, *Arabidopsis thaliana*, DEBAO: *Cycas debaoensis*, AMTR: *Amborella trichopoda*, OS: *Oryza sativa*.

Many gymnosperms are tall, woody plants with cell walls containing large quantities of cellulose, xyloglucan, glucomannan, homogalacturonans and rhamnogalacturonans^45^. In the *cellulose synthase* (*CESA*/*CSL*) superfamily^46^, we discovered the existence of putative ancestral *cellulose synthase-like B*/*H* (*CSLB*/*H*) and *CSLE*/*G* that are specifically shared by gymnosperms, and both gene groups originated before the divergence of *CSLB* and *CSLH* in angiosperms (Extended Data Fig. 6). Cycads have manoxylic wood, with a large pith, large amounts of parenchyma and relatively few tracheids, in contrast to most other gymnosperms, which have pycnoxylic wood, with small amounts of pith, cortex and parenchyma, and a greater density of tracheids^4^. The *glutamyltransferase 77* (*GT77*) family, involved in the synthesis of rhamnogalacturonan II, which is essential for cell wall synthesis in rapidly growing tissues^47^, is expanded in *C. panzhihuaensis* compared with other gymnosperms (Supplementary Note 11). In addition, gene families related to cell wall extension and loosening are uniquely expanded in *C. panzhihuaensis*, including those encoding hydroxyproline-rich glycoproteins, which are seven times more abundant in *Cycas* than in *Ginkgo*, and the fasciclin-like arabinogalactan proteins, which are twice as numerous in *Cycas* as in *Ginkgo*, *Sequoiadendron giganteum* and *Pseudotsuga menziesii*. How all these gene families related to wood features are regulated in cycads relative to other gymnosperms will be important for understanding the differences in wood density.

## The evolution of pollen, pollen tube and sperm

Another major innovation during seed plant evolution is the production of pollen and the pollen tube^36^. We found that many genes regulating pollen and pollen tube development (pollen maturation, pollen tube growth, pollen tube perception and prevention of multiple-pollen tube attraction) were gained (or the respective gene family expanded) in the MRCA of extant seed plants (Fig. 2b), as might be predicted for these features. For instance, those genes encoding egg cell-secreted proteins that prevent attraction of multiple pollen tubes^48^ originated in the MRCA of living seed plants. The *Ole e 1*-like gene families, which encode proteins that accumulate in the pollen tube cell wall and play a role in pollen germination and pollen tube growth^49^, are remarkably expanded in the MRCA of extant seed plants compared with non-seed plants (Supplementary Note 6). Such expansion also includes *polcalcin*, which is involved in calcium signalling to guide pollen tube growth^50^ (Supplementary Note 11). Both the *COBRA* and *COBRA*-like protein gene families are expanded in *Cycas* and other seed plants compared with non-seed plants, and the *COBRA*-like protein localizes at the tip of the pollen tube membrane and plays an important role in pollen tube growth and guidance^51^ (Supplementary Note 11).

**Extended Data Fig. 7:**
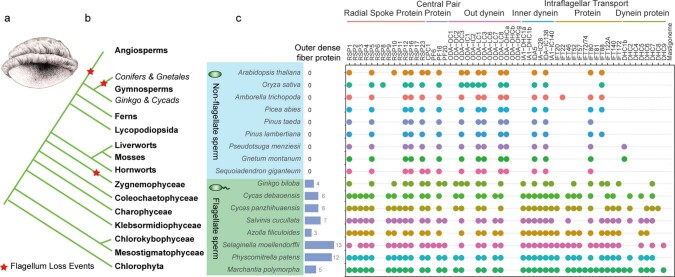
The Evolution of flagella related genes in embrophyta. (**a**) Sketch of the Cycas sperm. (**b**) Schematic diagram of flagellum loss events in green linage. (c) Distribution of outer dense fiber protein and other key flagellar proteins across representative embrophyta.

All seed plants produce pollen and deliver their sperm through the growth of a pollen tube, whereas all non-seed land plants (that is, bryophytes, lycophytes and ferns) rely on free-swimming motile sperm for sexual reproduction, as do the ancestors of land plants^1,4^ (Extended Data Fig. 7a,b). The exceptions among seed plants are cycads and *Ginkgo*, both of which have pollen grains that release motile spermatozoids that, following pollination, swim the remaining minute distance within the ovule to fertilize the egg^52^ (Supplementary Video 1). Sperm motility is conferred by a flagellar apparatus, and most genes related to its assembly occur in the *C. panzhihuaensis* genome. *Ginkgo also* retains flagellar genes, although fewer, and most notably lacks those encoding radial spoke proteins (RSP) (that is, RSP2, RSP3, RSP9 and RSP11; Extended Data Fig. 7c). By contrast, *Gnetum*, conifers and angiosperms, which develop non-flagellated spermatozoa, lost many flagellar structural genes (Supplementary Note 12). Outer dense fibres are unique accessory structures that maintain the structural integrity of flagella and are vital for flagellar function^53^. Outer dense fibres exist in *C. panzhihuaensis* and *Gingko biloba*, as well as all non-seed land plants, but are absent in *Gnetum*, conifers and angiosperms, all of which have non-motile sperm (Extended Data Fig. 7c). The shift from swimming to non-motile sperm is a major innovation in land plant evolution, and *C. panzhihuaensis* and *G. biloba* exhibit an ancestral gene content that is part of the shift from producing flagellate to non-flagellate sperm cells.

## Sex chromosomes and sex determination in *Cycas*

**Fig. 4:**
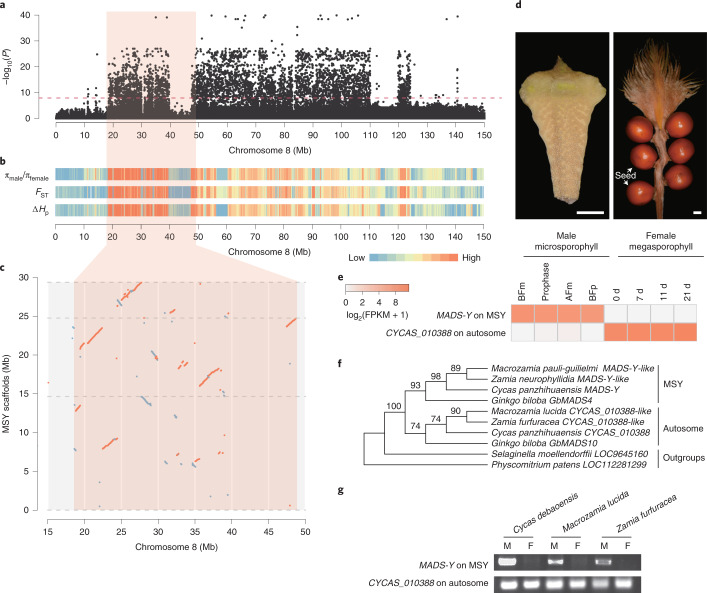
Identification of male-specific chromosomal region in *Cycas*. **a**, Manhattan plot of GWAS analysis of sex differentiation in 31 male and 31 female *Cycas* samples. The red horizontal dashed line represents the Bonferroni-corrected threshold for genome-wide significance (α = 0.05). *P* values were calculated from a mixed linear model association of SNPs. Association analyses were performed once with a population of 31 male and 31 female individuals. **b**, Ratio of *π*, *F*_ST_ and difference of pooled heterozygosity (Δ*H*_p_) within a 100-kb sliding window between the female and male sequences. Colour represents values from low (blue) to high (red). **c**, Genome alignment of the MSY scaffolds with the corresponding female-specific region on chromosome 8. Scaffolds are separated by grey dashed lines. Red lines represent alignments >5 kb on the forward strand, and blue lines represent those on the reverse strand. Pink boxes in **a**–**c** represent the most differentiated regions between the sex chromosomes. **d**, Photographs of microsporophyll and megasporophyll of *C. panzhihuaensis*. Bar, 1 cm. **e**, Sex-specific expression of *MADS-Y* (*CYCAS_034085*) and *CYCAS_010388* in male and female reproductive organs. Microsporophyll tissues were collected before meiosis (BFm), during prophase (Prophase), after meiosis (AFm) and before pollination (BFp); female tissues were collected at 0, 7, 11 and 21 days post-pollination. **f**, Phylogeny of *MADS-Y* homologues across land plants. Genes from MSY and autosomes are marked on the right, and those from *Selaginella* and *Physcomitrium* are used as outgroups. Numbers above branches represent bootstrap scores from IQ-TREE. **g**, Molecular genotyping of male and female cycad samples from *Cycas debaoensis*, *Macrozamia lucida* and *Zamia furfuracea* using primers specific to homologues of *MADS-Y* and *CYCAS_010388*. Source data

Heteromorphic chromosomes have been reported to be associated with sex determination in *Cycas*^54^. To reveal the underlying genetic mechanism of sex determination, we carried out genome-wide association studies (GWAS) analysis of sex as a binary phenotype for *C. panzhihuaensis* and identified the most significant association signals on chromosome 8, spanning the first 124 Mb on the reference female genome (Fig. 4a). This sex-associated region is also the most differentiated between male and female *Cycas* genomes, with the largest fixation index (*F*_ST_; Supplementary Fig. 37) and the most differentiated nucleotide diversity (*π*) and heterozygosity ratios characterizing the window between 18 and 50 Mb on chromosome 8 (Fig. 4b and Supplementary Note 13). These results confirm that *Cycas* possesses an XY sex determination system positioned on chromosome 8.

**Extended Data Fig. 8:**
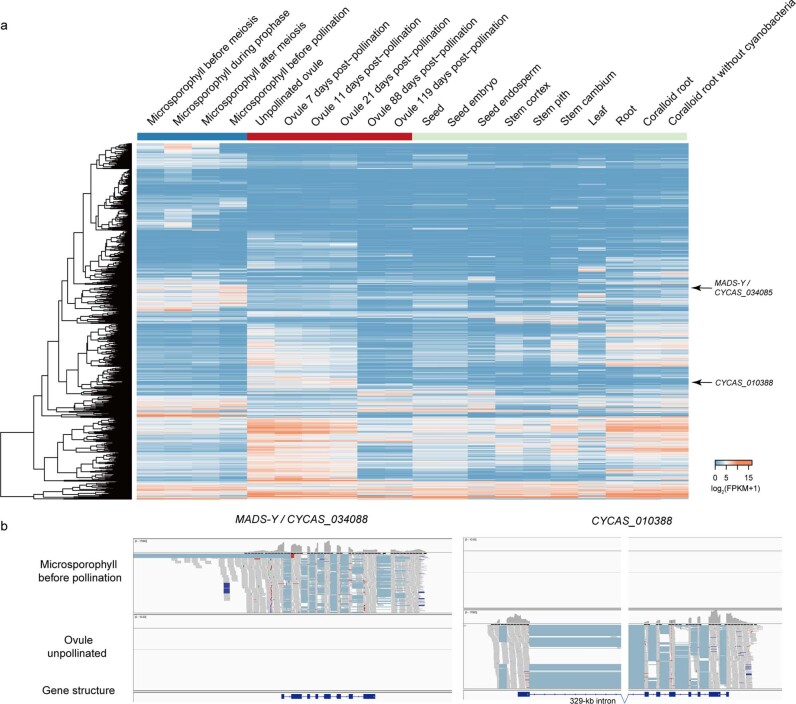
The phylogeny and expression level of TPS. (**a**) Phylogenetic tree of the TPS gene family. The tree was constructed using RAxML (the maximum-likelihood method) with PROTCATGTR amino acid substitution model and 500 bootstrap replicates. The bootstrap values ≥ 50% are shown in the central branches. The red colors in the tree represent the cycas genes. (**b**) Heatmap of TPS gene family in different tissues of *C. panzhihuaensis*. The * denotes the *C*. *panzhihuaensis* specific TPS genes.

Assembling the male-specific region of the Y chromosome (MSY) based on Nanopore long-read and Hi-C data resulted in 45.5 Mb of sequence distributed over 43 scaffolds, most of which aligned to the sex-differentiation region on chromosome 8 (Fig. 4c and Supplementary Fig. 38). The assembled MSY had an almost 80-Mb difference in length from the corresponding region on the X chromosome, which agrees with the heteromorphy of the *Cycas* sex chromosomes. We annotated 624 putative protein-coding genes within the MSY, 11 of which were highly expressed (transcripts per million (TPM) > 1) in the microsporophylls. The most highly expressed gene in the MSY and also the most differentially regulated gene between the two sexes is *CYCAS_034085* (Fig. 4d,e and Extended Data Fig. 8), which encodes a *GGM13*-like MADS-box transcription factor (TF), belonging to a lineage sister to the angiosperm *AP3/PI* clade that plays crucial roles in floral development. Its closest homologue, *CYCAS_010388*, was identified on autosomal chromosome 2. In contrast to *CYCAS_034085*, *CYCAS_010388* was much more highly expressed in the ovule than in the microsporophyll (Fig. 4e). A male-specific polymerase chain reaction (PCR) product of *CYCAS_034085* was amplified from all tested male cycad samples, but was not detected in female samples, whereas a *CYCAS_010388*-specific PCR product was amplified in both males and females (Fig. 4g and Supplementary Fig. 39b). Because of the presence in MSY and its exclusive expression pattern in males, we named *CYCAS_034085* as *MADS-Y*, a potential sex determination gene.

The reduced size of MSY compared with the X chromosome indicates that the Y chromosome of *Cycas*, unlike that reported for some angiosperms^55^, underwent severe degeneration and gene loss. The most divergent 32-Mb region (between the 18 and 50 Mb locations) between the X and Y chromosomes probably represents an ancient evolutionary segment in the *Cycas* sex chromosomes. The broad association of the *MADS-Y* homologue with sex in cycads indicates a conserved sex determination system within this ancient lineage (Fig. 4f and Supplementary Fig. 39). Moreover, the presence of *GbMADS4*, a homologue of the *Cycas MADS-Y*, in *Ginkgo* male-specific contigs^56^ suggests that the same mechanism for sex determination might have originated before the split of cycads and *Ginkgo*, thus representing an ancient system of sex determination in seed plants.

## Evolution of disease and herbivore resistance genes

All three types of immune receptors—*CC-NBS-LRR* (*CNL*), *TIR-NBS-LRR* (*TNL*) and *RPW8-NBS-LRR* (*RNL*)—show patterns of expansion in *C. panzhihuaensis* and other gymnosperms, compared with non-seed plants (Supplementary Note 14). *CNLs* are expanded widely in both gymnosperms and angiosperms, whereas the *TNL* family tends to have been more expanded in gymnosperms than in most angiosperms, indicating different evolutionary patterns of plant resistance (*R*) genes in these two lineages. Our data suggest that *RNL* genes occur widely in gymnosperms. The *RNL* family plays a critical role in downstream resistance signal transduction in angiosperms, and the broad occurrence of the *RNL* family in gymnosperms suggests that this signalling pathway may have been established no later than the origin of seed plants. Gene families encoding resistance-related proteins are greatly expanded in *C. panzhihuaensis* and other gymnosperm genomes compared with non-seed plants (Supplementary Note 14). For example, genes encoding endochitinases and chitinases as defences against chitin-containing fungal pathogens are expanded as tandem repeats in the *C. panzhihuaensis* and most gymnosperm genomes compared with other land plants.

Cycads comprise many more living species^57^ than *Ginkgo*, which was once diverse in the Mesozoic but includes only one extant species^58^. One possible explanation is that cycads may have acquired enhanced resistance to pathogens and herbivores through encoding diversified resistance-related genes and the biosynthesis of diversified secondary compounds^4,8^. Indeed, comparisons of the *Cycas* and *Ginkgo* genomes reveal many *Cycas*-specific orthogroups enriched in pathogen interaction pathways (Supplementary Note 14), and *C. panzhihuaensis* also shows remarkable expansions in plant immunity and stress response gene families compared with *Ginkgo*, including genes that encode programmed cell death, abiotic stress response, serine protease inhibitors against pests and ginkbilobin with antibacterial and antifungal activities (Supplementary Note 14).

**Extended Data Fig. 9:**
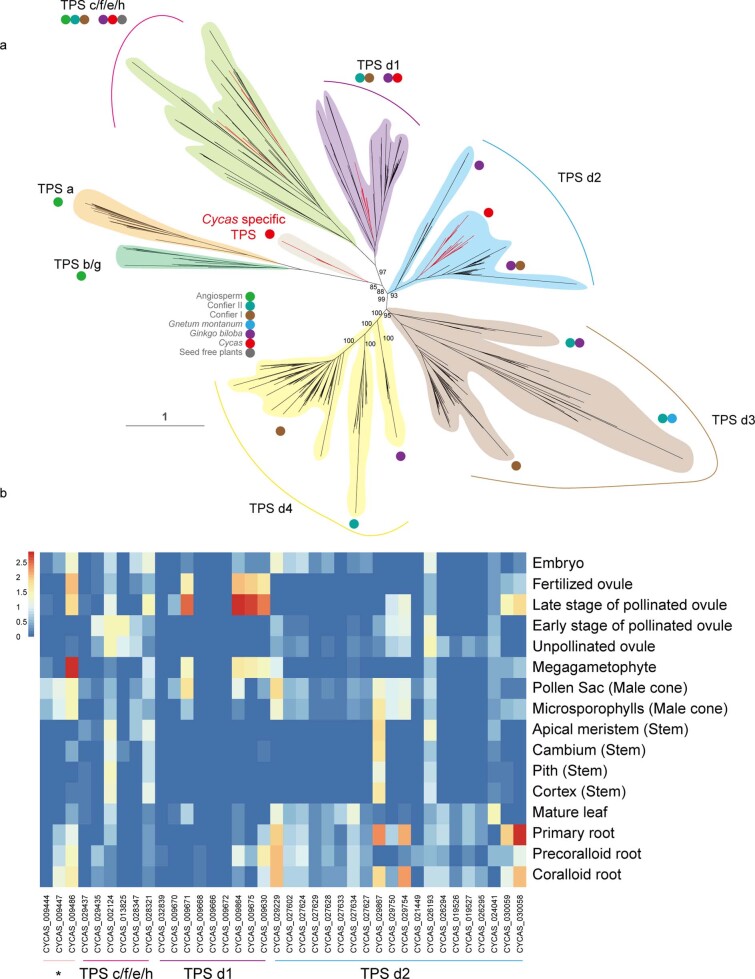
Two MADS-box transcription factor genes differentially expressed in reproductive organs of *C. panzhihuaensis*. (**a**) Heatmap of 1,971 genes differentially expressed in males and females’ organs. Arrows indicate CYCAS_034085 on the MSY and CYCAS_010388 on chromosome 2. (**b**) Expression of CYCAS_034085 on MSY and CYCAS_010388 on chromosome 2 in male microsporophyll and in the ovule.

Terpenoids are a diverse group of secondary metabolites encoded by terpene synthase (*TPS*) genes^59^. Several TPS subfamilies (*TPS-a* to *TPS*-*h*) are known in plants^60^, among which the *TPS-d* family is unique to gymnosperms, and three of the four types of *TPS-d* were found in *C. panzhihuaensis*, with remarkable expansions of *TPS-d2* compared with *Ginkgo* and most other gymnosperms (Supplementary Note 15). In addition, we identified a novel *TPS* subfamily in *Cycas*, with three copies in *C. panzhihuaensis* and eight copies in *Cycas debaoensis* (Extended Data Fig. 9a). The gene expression levels of all *TPS* genes across different *C. panzhihuaensis* tissues (Extended Data Fig. 9b) reveal that many *TPS* genes are mainly expressed in the root (especially primary root and coralloid root), microsporophyll and pollen sac, late stage of the pollinated ovule and fertilized ovule. The three *Cycas*-specific *TPS* genes were mainly expressed in the root and male cone, but one of them (*CYCAS_009486*) is particularly highly expressed in the megagametophyte and in the post-pollination and fertilized ovule.

## *Cycas* obtained a cytotoxin defence gene via horizontal gene transfer

**Fig. 5:**
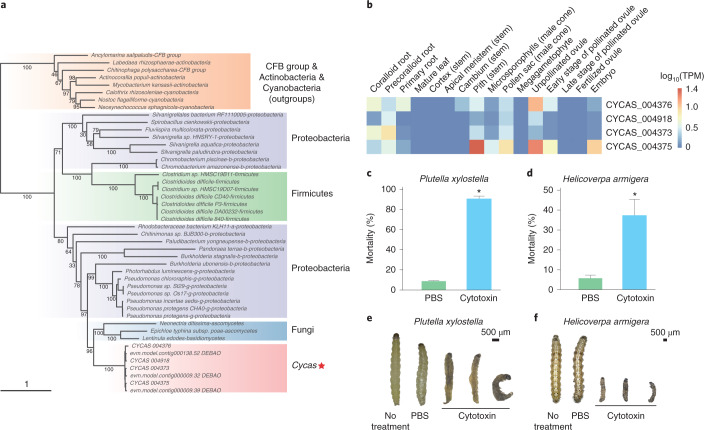
Origin of a *Cycas* insecticidal protein. **a**, Phylogenetic analysis of the TcdA/TcdB pore-forming domain containing proteins shows that the genes encoding four cytotoxin proteins of *Cycas* were likely acquired from fungi through an ancient horizontal gene transfer event. The maximum likelihood tree was generated by RAxML with the PROTCATGTR model and 1,000 bootstrap replicates. The numbers above the branches are bootstrap support values. **b**, The expression level of four cytotoxin proteins in different tissues of *C. panzhihuaensis*. The digital expression values were normalized using the TPM method. **c**,**d**, Mortalities of *Plutella xylostella* (**c**) and *Helicoverpa armigera* (**d**) after treatment with phosphate buffered saline (PBS) and cytotoxin. The asterisk indicates a significant difference (two-sided Student’s *t*-test, *P* < 0.05, *n* = 3 biologically independent experiments), whereas the error bar represents the standard error. **e**,**f**, Morphologies of *Plutella xylostella* (**e**) and *Helicoverpa armigera* (**f**) after receiving PBS and cytotoxin treatments.

Genes of fungal or bacterial origin are rare in seed plants^61^. However, we identified a gene family in the *C. panzhihuaensis* genome that appears to have been acquired from a microbial organism and that codes for a *Pseudomonas* fluorescens insecticidal toxin (*fitD*). The acquired genes are flanked by vertically inherited plant sequences. We further confirmed that the relevant assembled regions were free of bacterial contamination. Transcriptomes and PCR amplification from genomic DNA indicated that these genes occur in many *Cycas* species (Supplementary Note 16). The *fitD* gene family comprises four gene copies in the *C. panzhihuaensis* genome and three copies in the *C. debaoensis* genome (Supplementary Table 51); each copy encodes a protein that is similar to the fit toxin and the ‘makes caterpillars floppy’ (mcf) toxin of the bacterium *Photorhabdus luminescens*, a lethal pathogen of insects. Both fit and mcf toxins are known for their insecticidal properties, and fit- or mcf-producing bacteria are often used in pest biocontrol^62–64^. Phylogenetic analyses suggest that the *fitD* genes might have been acquired from fungi and then expanded before the divergence of *C. panzhihuaensis* and *C. debaoensis* (Fig. 5a). The *fitD* family genes are mainly expressed in roots, reproductive tissues such as male cones, unpollinated or early stages of pollinated ovules and embryos (Fig. 5b). Injection of the synthesized *C. panzhihuaensis fitD* protein resulted in significantly higher mortality in larvae of both the diamondback moth (*Plutella xylostella*) and cotton bollworm (*Helicoverpa armigera*) (Fig. 5c,d). The acquisition of the *fitD* gene family may have provided an important defence for *Cycas* against insect pests.

## Conclusions

The high-quality genome sequence for *Cycas*, the last major lineage of seed plants for which a high-quality genome assembly was lacking, closes an important gap in our understanding of genome structure and evolution in seed plants. This genome enables comparative genomics and phylogenomic analyses to unravel the genetic control of important traits in cycads and other gymnosperms, including a WGD shared by gymnosperms, a sex determination mechanism that appears to be shared by cycads and *Ginkgo*, and critical gene innovations including those that enable seed and pollen tube formation, as well as chemical defence.

## Methods

### Plant materials

Fresh megagametophytes of *Cycas panzhihuaensis*, cultivated in the garden of the Kunming Institute of Botany, Chinese Academy of Sciences, were collected for genome sequencing. The plant was originally transplanted from the Pudu River, Luquan county, Yunnan, China (25° 57′ 35.2584″ N, 102° 43′ 41.5848″ E) and the voucher specimen (collection number: PZHF03) has been deposited in the Herbarium of the Kunming Institute of Botany (KUN). For transcriptome sequencing, we sampled 12 different types of organs and tissues from *C. panzhihuaensis*, including megagametophyte, pollen sac, microsporophylls, apical meristem of stem, cortex of stem, pith of stem, cambium of stem, mature leaf, young leaf, primary root, precoralloid roots and coralloid roots (Supplementary Table 2). Ovule material was collected from two artificially pollinated individuals, and we divided the development stages into four: unpollinated ovule (before the artificial pollination), early stage of pollinated ovule (21 d after the artificial pollination), late stage of pollinated ovule (88 d after the artificial pollination) and fertilized ovule or seed (119 d after the artificial pollination) (Supplementary Tables 2 and 19). In addition, stem and root tissues of *C. panzhihuaensis* were used to generate full-length transcriptomes (Supplementary Table 2). For phylogenomic analyses, we newly generated transcriptomes of 47 gymnosperms (Supplementary Tables 2 and 13). We also sequenced transcriptomes of 339 cycad species (Supplementary Tables 2 and 14). For population resequencing, fresh leaf samples were collected for 31 male and 31 female plants that were randomly sampled in the *Cycas panzhihuaensis* National Natural Reserve in Sichuan, China, where there is a population of approximately 38,000 *C. panzhihuaensis* individuals (Supplementary Table 4).

### DNA and RNA sequencing

For genome sequencing, the genomic DNA was extracted by the QIAGEN Genomic kit followed the manufacturer’s instructions^65^. Nanodrop and Qubit (Invitrogen) were used to quantify the DNA. Nanopore libraries were prepared by SQK-LSK108 and sequenced using a Nanopore PromethION sequencer. The rest of the DNA was used to generate short-read sequences using an MGI-SEQ platform, with 150-bp read length and 300–500 DNA-fragment insert size. Hi-C libraries were created from fresh megagametophyte, following a previously published method^66^. Briefly, the tissue was fixed in formaldehyde, lysed and the cross-linked DNA was digested overnight with HindIII. Sticky ends were biotinylated and proximity-ligated to generate chimeric junctions, which were subsequently physically sheared to 500–700 bp in size. The initial cross-linked long-distance physical interactions were then represented by chimeric fragments, which were processed into paired-end sequencing libraries. Paired-end reads were produced on both the MGI-SEQ and Illumina HiSeq X platforms. See Supplementary Note 3 for details on transcriptome, organelle genome and small RNA sequencing.

### Genome assembly

About 1,010 Gb (~100×) Nanopore long-read data were used for genome assembly using NextDenovo (https://github.com/Nextomics/NextDenovo) with default parameters (read_cutoff = 1k, seed_cutoff = 12k, minimap2_options_cns = -x ava-ont -k17 -w17). To further enhance assembly contiguity, about 456 Gb of Hi-C data were used to execute Hi-C chromosome conformation in conjunction with 3D-dna algorithm^67^. The accuracy of Hi-C based chromosomal assembly was assessed using Juicerbox’s chromatin contact matrix.

### Repeat annotation

We identified tandem repeats and transposable elements throughout the genome. Tandem repeats were predicted using Tandem Repeat Finder (v.4.07)^68^ with the following parameters: ‘Match = 2, Mismatch = 7, Delta = 7, PM = 80, PI = 10, Minscore = 50 and MaxPeriod = 2,000’. To maximize the opportunity of identifying transposable elements, a combination of de novo and homology-based approaches was performed following the Repeat Library Construction-Advanced pipeline (http://weatherby.genetics.utah.edu/MAKER/wiki/index.php/Repeat_Library_Construction-Advanced). RepeatMasker^69^ and RepeatProteinMask^69^ were used to search for known repeat sequences; MITE-hunter^70^, LTR_retriever^71^, LTR_FINDER (v.1.0.6)^72^ and RepeatModeler^73^ were then used to search the repeats de novo. The MITE, LTR and consensus repetitive libraries generated by RepeatModeler were combined and further used as the input data for RepeatMasker.

### LTR identification and estimation of LTR insertion times

All the candidate LTR elements were first identified using LTR_FINDER and LTR_retriever. LTR_STRUC^74^ was then used to extract the complete 5′- and 3′-ends of the LTR elements. RepeatClassifier was then used to classify the candidate LTR. Distmat from the EMBOSS (v.6.5.7.0) package was then used to calculate the *K* value of the retrotransposons’ 5′- and 3′-LTR sequences. Finally, the insertion time (*T*) of LTRs was calculated using the formula *T* = *K*/2*r*, where *r* is the average substitution rate of 2.2 × 10^−9^ substitutions per year per synonymous site.

### Gene annotation and functional annotation

Three types of evidence were used to predict protein-coding genes in the *C. panzhihuaensis* genome. For protein evidence, Genewise^75^ was used to predict gene models based on *Cycas* proteins downloaded from the UniProt protein database and other proteins collected from representative plant species. Next, Hisat^76^ was used to map the transcriptome to the genome, and then StringTie^77^ was used to predict transcriptome-based gene models. Next, a custom training hint parameter was used to predict ab initio-based gene models in AUGUSTUS^78^. All the evidence was finally combined and integrated by EVidenceModeler^79^. To maximize the opportunity of identifying high-confidence genes, we further filtered the genes that were not expressed in the full-length transcriptome or did not match to functional annotation results. For functional annotation, the gene models were blasted against the UniProt, TrEMBL, KEGG, KOG and NR databases. The domain and gene ontology of the gene models was identified by InterProScan^80^ (using data from Pfam, PRINTS, SMART, ProDom and PROSITE).

### Identification of key candidate functional genes

Based on the following criteria, all candidate genes were screened: first, candidate gene sequences were detected by BLAST searches with an e value cut-off of 1 × 10^−5^to the collected query gene sequences gathered from previous studies or public databases; and second, features of candidate genes should be similar to the online functional annotation or UniProt functional annotation as the query genes. With regard to the identification of flagellar genes, 58 flagellar-related genes were collected from previous studies^81^. The Reciprocal Best Blast hit method was employed to identify flagella-related genes. For seed-related genes, we searched the genes against both the known seed database (seedgenes.org/) and previous studies. We firstly used an e value (<1 × 10^−20^) as a cut-off to filter candidates and then filtered the candidates with functional annotation. Regarding the identification of TFs, we used the HMMER search method. HMMER domain structure models were downloaded from the Pfam website (https://pfam.xfam.org/), for each TF as present in the TAPscan v.2 database for TFs (https://plantcode.online.uni-marburg.de/tapscan/). Preliminary TF candidate genes were collected for each species (<1 × 10^−5^) by searching the Hidden Markov Model profile. Parts of genes were then filtered if they were not the homologues according to their functional annotation of SwissProt (<1 × 10^−5^). In the end, we filtered genes containing a wrong domain under the TAPscan v.2 transcription factor database domain rules. Phylogenetic tree analysis was used to verify the majority of TFs and transcriptional regulators. Details about phylogenetic tree reconstruction for each TF can be found in the figure captions.

### Phylogenetic reconstruction and divergence-time estimation

#### Nuclear phylogenetic reconstruction

The downloaded genome sequences and the newly generated genome sequences of *C. panzhihuaensis* were used to construct the orthogroups using OrthoFinder^82^ with default settings. The software KinFin^83^ was used to select single-copy gene families for phylogenetic reconstruction with default parameters. TranslatorX^84^ was used to build gene alignments for codon (nt), codon 1st + 2nd (nt12) and amino acid (aa) sequences (command: perl translatorx_vLocal.pl -i gene.fa -o gene.out -p F -t F -w 1 -c 1 -g "-b1="$b1" -b2="$b1" -b3=8 -b4=5 -b5=h -b6=y"). IQ-TREE 2 (ref. ^85^) was used to infer the maximum likelihood trees with an initial partition scheme of codon positions combing ModelFinder, tree search, and ultrafast bootstrap. ASTRAL^86^ was used to summarize the coalescent species tree and the quartet supports with default settings (-t 8). ASTRAL uses the quartet trees of the maximum likelihood phylogenies of each gene to produce the topology of the species tree while quartet supports (bar charts) show the percentage of quartets that agree with a specific branch in the species tree. STAG (https://github.com/davidemms/STAG) was also used to construct the species tree with default settings using low-copy genes (one to four copies). The software PHYPARTS^87^ was used to infer and visualize the gene tree conflicts on the species tree topology with default settings. The software DISCOVISTA^88^ was used to summarize the conflicts among different analytical methods and datasets, regarding several focal phylogenetic relationships.

#### Molecular dating and diversification analysis

The transcriptome sequencing reads from 339 cycad species were generated in the current study. Clean reads were assembled with TRINITY^89^, and the longest transcripts were selected and translated with TRANSDECODER (https://github.com/TransDecoder). OrthoFinder^82^ was then used to construct orthogroups for all the cycad species using *Ginkgo* as the outgroup. The software KinFin^83^ was used to select the mostly single-copy genes for phylogenetic reconstruction with default settings. TranslatorX^84^, IQ-TREE 2 (ref. ^85^) and ASTRAL^86^ were used to align the sequences and to infer the species tree for cycads as aforementioned. The software SORTADATE^90^ was used to select genes with mostly concordant evolutionary histories for dating analyses using MCMCTREE within the software PAML 4 (ref. ^91^). Rate priors and time priors were set following the method of Morris et al.^92^. A total of 27 fossils were used to calibrate the chronogram of seed plants, and six fossils for the chronogram of cycads. The diversification pattern for cycads were analysed with Bayesian analysis of macroevolutionary mixture (www.bamm-project.org) following Condamine et al.^93^

See Supplementary Note 5 for details on organellar phylogenetic reconstruction, evaluation of the impact of RNA editing and investigation of cyto-nuclear incongruences.

### Identification of whole-genome duplication

An integrated phylogenomic approach and a method to analyse synteny as described previously^35,94,95^ were used to identify the WGD events in seed plant evolution. The protein-coding sequences of 15 completely sequenced genomes and 1 transcriptome, representing seven gymnosperms (*C. panzhihuaensis*, *Encephalatos longifolius, G. biloba*, *Gnetum montanum*, *Picea abies*, *Pinus taeda* and *Sequoiadendron giganteum*), six angiosperms (*Arabidopsis thaliana*, *Amborella trichopoda*, *Cinnamomum micranthum*, *Liriodendron chinense*, *Nymphaea colorata* and *Oryza sativa*) and three other vascular plant outgroups (*Azolla filiculoides*, *Salvinia cucullate* and *Selaginella moellendorffii*), were classified into putative gene families/subfamilies by OrthoFinder^82^, and then scored for gene duplications across global gene families. For the phylogenetic analysis of gene families, amino acid sequences of each gene family were first aligned with MAFFT^96^, the program PAL2NAL^97^ was then used to construct their corresponding nucleotide sequence alignments. We used trimAl^98^ to remove poorly aligned portions of alignments using the ‘automated1’ option, which implements a heuristic algorithm to optimize the process for trimming the alignment. Finally, maximum likelihood trees were calculated using RAxML^99^ with the GTRGAMMA model and bootstrap support was estimated based on 100 replicates. Following Wu et al.^95^, we applied two basic requirements for the determination of a reliable duplication event: (1) at least one common species’ genes are present in two child branches; and (2) the bootstrap values of the parental node and one of the child nodes are both ≥50%. After scoring gene duplications in a large-scale analysis on gene families, we were able to confidently identify the nodes with concentrated gene duplications across the phylogeny, which possibly support the WGD events. Furthermore, because syntenic information is the most solid evidence for WGD, and the legacy of syntenic blocks may be found if the concentrated gene duplications are indeed derived from WGD events, we also looked into whether such syntenic blocks exist. The intra- and intergenomic syntenic analyses were conducted using MCscanX^100^, with the default settings.

In addition, the Nei–Gojobori method^101^ as implemented in the PAML package’s yn00 program^91^ was used to estimate synonymous substitutions per synonymous site (*K*_*S*_) for pairwise comparisons of paralogous genes located on syntenic blocks. To search for genome-wide duplications, we used DupGen_finder (https://github.com/qiao-xin/DupGen_finder) to identify duplicated genes that were classified into five different categories: WGD duplicates, tandem duplicates, proximal duplicates, transposed duplicates and dispersed duplicates.

### Identification of the sex-differentiation region

To identify the sex-differentiation region in the *Cycas* genome, a GWAS approach was adopted on sequence variations from 31 male and 31 female individuals with sex treated as a binary phenotype. Briefly, raw reads were filtered by Trimmomatic (v.0.38) (ILLUMINACLIP:adapter.fa:2:30:10 HEADCROP:10 LEADING:3 TRAILING:3 SLIDINGWINDOW:5:15 MINLEN:140), and read alignment and single-nucleotide polymorphism (SNP) calling were performed using the Sentieon pipeline^102^. SNPs were filtered using the following criteria: (1) SNPs were filtered by GATK VariantFiltrations with ‘QD < 2.0 || FS > 60.0 || MQ < 40.0 || SOR > 3.0 || MQRankSum < −12.5 || ReadPosRankSum < −8.0’, and indels with ‘QD < 2.0 || FS > 200.0 || SOR > 10.0 || MQRankSum < −12.5 || ReadPosRankSum < −8.0’; (2) total depth <80 or >1,300; (3) variants with more than two alleles; (4) variants with a missing rate >10% or minor allele frequencies <0.1 were removed; and (5) a linkage disequilibrium pruning with PLINK (v.1.9) using a window size of 10 kb with a step size of one SNP and *r*^2^ threshold of 0.5, resulting a 4.65-million pruned SNP set for association analysis of sex differentiation. GWAS analysis of sex differentiation was performed on the linkage disequilibrium-pruned SNP set using the EMMAX program^103^ (beta-07Mar2010 version). The BN kinship matrix and the first five components calculated from the principal component analysis^104^ (v.1.91.4beta3) were included as random effects. Genetic differentiation (*F*_ST_) and nucleotide diversity (*π*) were calculated within a non-overlapping 100-kb window using VCFtools^105^ (v.0.1.13). See Supplementary Note 13 for details on assembly of *Cycas* male-specific regions, phylogenetic analysis of MADS-Y and CYCAS_010388 homologues, and genotyping of cycad male and female samples.

### Analysis of the differentially expressed genes

Transcriptome sequencing reads were trimmed using Trimmomatic^106^ program (ILLUMINACLIP:adapter.fa:2:30:10 HEADCROP:10 LEADING:3 TRAILING:3 SLIDINGWINDOW:5:15 MINLEN:140) and mapped against *C. panzhihuaensis* annotated gene models using bowtie2 (with sensitive mode and default alignment parameters) by retaining the best alignments. TPM were calculated using the eXpress program, which was incorporated in the Trinity^89^ package. Furthermore, differentially expressed genes with a differential expression level of false discovery rate ≤ 0.01 and at least a twofold expression change were identified using DESeq2 (ref. ^107^). To identify the co-expressed genes during the seed development, we used the R package WGCNA^108^ on the basis of the TPM data of the genes whose expression showed a coefficient of variation >0.5 across the four stages. To better visualize the expression levels, we normalized the expression results. For each gene, the TPM value normalized by the maximum TPM value of all stages is shown. Fisher’s exact test was used to examine whether the functional categories were over-represented. The resulting *P* values were adjusted to *Q* values by the false discovery rate correction.

### Identification of the horizontally transferred cytotoxin genes in *C. panzhihuaensis*

The cytotoxin protein sequences of *Cycas* were used as query to perform BLASTP searches against the NCBI nr protein sequence database using the cut-off e value = 1 × 10^−5^ and max_target_seqs = 20,000. We also performed additional BLAST searches against the OneKP database and many other available genomes. See Supplementary Note 16 for details on verification and phylogenetic analysis of the cytotoxin gene.

### Assessing the effectiveness of cytotoxin

To improve the expression efficiency of cytotoxin in the prokaryotic system, the full-length coding sequence of the *C. panzhihuaensis* cytotoxin protein was optimized for its codons. *C. panzhihuaensis*, the optimized sequence was synthesized and ligated to the pET-28a vector. The pET-28a-CR toxin plasmid was transformed into *Escherichia coli* BL 21 (DE3) pLysS cells, the resulting strain was used for expression and purification of recombinant proteins under the control of isopropyl-β-d-thiogalactoside-inducible T7 promoter. Overnight-grown cultures were diluted 100-fold with 200 ml of fresh LB medium and further grown at 37 °C and 220 r.p.m. rotation until the optical density at 600nm reached 0.5. The culture was induced by adding a 0.01 mM final concentration of isopropyl-β-d-thiogalactoside and incubated at 28 °C for 6 h. Cells were then harvested and suspended with 20 ml 50of mM Tris–HCl buffer with pH 8 at 4 °C, containing 200 mM NaCl, then disrupted by sonication at 4 °C. In an RC5 plus centrifuge, the cell lysate was spun at 13,800*g* for 40 min at 4 °C. The preceding step’s supernatant was put onto a Ni-NTA agarose column that had been pre-equilibrated with Tris–NaCl buffer at 4 °C. Tris–NaCl buffer containing 20 mM imidazole was used to thoroughly wash the column, and the 6× His-tagged protein was eluted with Tris–NaCl buffer containing 250 mM imidazole. The elution product containing pure protein were washed three times with Tris–NaCl buffer and concentrated using centricon (Millipore PM10). Using an horseradish peroxidase-conjugated monoclonal antibody and a western blot assay, the purified His-tagged protein was identified (HRP-66005). See Supplementary Note 16 for further details on experimental verification of the function of *Cycas* cytotoxin.

### Detection of metabolites and phytohormones

The plant tissues were collected and stored in liquid nitrogen, then transferred to freezer at −80 °C. For detection of metabolites, tissue samples were preliminarily disposed using 2-chlorophenylalanine (4 ppm) methanol. Samples and glass beads were then put into a tissue grinder for 90 s at 55 Hz, followed by centrifugation at 13,780*g* at 4 °C for 10 min, taking the supernatant and filtering through a 0.22-μm membrane, and transferring the filtrate into the detection bottle before liquid chromatography mass spectrometry analysis. The sample extracts were the analysed using the ultra high-performance liquid chromatography system Vanquish (ThermoFisher Scientific) and Q Exactive HF-X (ThermoFisher Scientific). For the quantitative detection of phytohormones (auxin, cytokinins, ethylene, abscisic acid, jasmonic acid, gibberellin, salicylic acid and brassinolide), tissue samples of primary root, precoralloid roots and coralloid roots, unpollinated ovule, early stage of pollinated ovule, late stage of pollinated ovule, fertilized ovule and mature embryo were collected. Vanquish (ThermoFisher Scientific) and the Q Exactive HF-X (ThermoFisher Scientific) were used for the detection of various phytohormones. The qualitative study was carried out using a self-constructed database that was built using the reference standards. To accomplish quantitative analysis, different concentrations of standard were utilized.

### Reporting Summary

Further information on research design is available in the Nature Research Reporting Summary linked to this article.

## Supplementary information

Supplementary Information Supplementary notes and figures for additional information of the manuscript.

Reporting Summary

Supplementary Video 1. A swimming sperm of *Cycas*.

Supplementary Table 1. Supplementary tables.

Supplementary Data. Raw gels figure for Supplementary Fig. 39.

## Source data

Source Data Fig. 4. Two unprocessed gel figures of Fig. 4

## Data Availability

The genome and transcriptome data, genome assemblies and annotations can be found at https://db.cngb.org/codeplot/datasets/public_dataset?id=PwRftGHfPs5qG3gE. The raw genomic, transcriptomic and Hi-C data generated in this study were deposited in the NCBI Sequence Read Archive (SRA, BioProject PRJNA734434) and the CNGB data center (https://db.cngb.org/) under project number CNP0001756. The genome assembly and annotation of C. debaoensis have been deposited in the National Genomics Data Center (NGDC) database under BioProject PRJCA063894. Source data are provided with this paper.
