# Supplementary material for: The Cycas genome and the early evolution of seed plants
Source: Nat Plants. 2022 Apr 18;8(4):389–401. doi: 10.1038/s41477-022-01129-7 (PMC9023351; doi:10.1038/s41477-022-01129-7)
Supplement: Supplementary file 1 — Supplementary notes and figures for additional information of the manuscript. [file 41477_2022_1129_MOESM1_ESM.pdf]

nature portfolio

https://doi.org/10.1038/s41477-022-01129-7

Supplementary information

The *Cycas* genome and the early evolution of seed plants

In the format provided by the authors and unedited

Supplementary Information

The *Cycas* genome and the early evolution of seed plants

Yang Liu[1,2#*], Sibo Wang[1#], Linzhou Li[1#], Ting Yang[1#], Shanshan Dong[2#], Tong Wei[1#], Shengdan Wu[3#], Yongbo Liu[4#], Yiqing Gong[2], Xiuyan Feng[5], Jianchao Ma[6], Guanxiao Chang[6], Jinling Huang[5,6,26], Yong Yang[7], Hongli Wang[1,8], Min Liu[1], Yan Xu[1,8], Hongping Liang[1,8], Jin Yu[1,8], Yuqing Cai[1,8], Zhaowu Zhang[1,8], Yannan Fan[1], Weixue Mu[1], Sunil Kumar Sahu[1], Shuchun Liu[2], Xiaoan Lang[2,14], Leilei Yang[2], Na Li[2], Sadaf Habib[2,9], Yongqiong Yang[10], Anders J. Lindstrom[11], Pei Liang[12], Bernard Goffinet[13], Sumaira Zaman[13], Jill L. Wegrzyn[13], Dexiang Li[14], Jian Liu[5], Jie Cui[15], Eva C. Sonnenschein[16], Xiaobo Wang[17], Jue Ruan[17], Jia-Yu Xue[18], Zhu-Qing Shao[19], Chi Song[20], Guangyi Fan[1], Zhen Li[21], Liangsheng Zhang[22], Jianquan Liu[23], Zhong-Jian Liu[24], Yuannian Jiao[25], Xiao-Quan Wang[25], Hong Wu[27], Ertao Wang[28], Michael Lisby[29], Huanming Yang[1], Jian Wang[1], Xin Liu[1], Xun Xu[1], Nan Li[2], Pamela S. Soltis[30], Yves Van de Peer[18,21,31*], Douglas E. Soltis[30,32*], Xun Gong[5*], Huan Liu[1*], Shouzhou Zhang[2*]

[1]State Key Laboratory of Agricultural Genomics, BGI-Shenzhen, Shenzhen 518083, China.

[2]Key Laboratory of Southern Subtropical Plant Diversity, Fairy Lake Botanical Garden, Shenzhen & Chinese Academy of Sciences, Shenzhen 518004, Guangdong, China.

[3]State Key Laboratory of Grassland Agro-Ecosystem, Institute of Innovation Ecology, Lanzhou University, Lanzhou 730000, China.

[4]State Environmental Protection Key Laboratory of Regional Eco-process and Function Assessment, Chinese Research Academy of Environmental Sciences, Beijing 100012, China.

[5]Key Laboratory for Plant Diversity and Biogeography of East Asia, Kunming Institute of Botany, Chinese Academy of Sciences, Kunming 650201, Yunnan, China.

[6]Key Laboratory of Plant Stress Biology, State Key Laboratory of Crop Stress Adaptation and Improvement, School of Life Sciences, Henan University, Kaifeng 475004, China.

[7]College of Biology and Environment, Nanjing Forestry University, Nanjing 210037, Jiangsu, China.
[8]College of Life Sciences, University of Chinese Academy of Sciences, Beijing 100049, China.
[9]School of Life Sciences, Sun Yat-sen University, Guangzhou 510275, Guangdong, China.
[10]Sichuan *Cycas panzhihuaensis* National Nature Reserve, Panzhihua 617000, Sichuan, China.
[11]Global Biodiversity Conservancy 144/124 Moo 3, Soi Bua Thong, Bangsalae, Sattahip, Chonburi 20250, Thailand.
[12]Department of Entomology, China Agricultural University, Beijing 100193, China.
[13]Department of Ecology and Evolutionary Biology, University of Connecticut, Storrs, CT 06269-3043, USA.
[14]Nanning Botanical Garden, Nanning 530000, Guangxi, China.
[15]Guangdong Provincial Key Laboratory for Plant Epigenetics, Longhua Institute of Innovative Biotechnology, College of Life Sciences and Oceanography, Shenzhen University, Shenzhen 518060, Guangdong, China.
[16]Department of Biotechnology and Biomedicine, Technical University of Denmark, Søltofts Plads 221, 2800 Kgs. Lyngby, Denmark.
[17]Shenzhen Agricultural Genome Research Institute, Chinese Academy of Agricultural Sciences, Shenzhen 518120, China.
[18]College of Horticulture, Academy for Advanced Interdisciplinary Studies, Nanjing Agricultural University, Nanjing 210095, China.
[19]State Key Laboratory of Pharmaceutical Biotechnology, School of Life Sciences, Nanjing University, Nanjing 210023, China.
[20]Chengdu University of Traditional Chinese Medicine, Chengdu 611137, Sichuan, China.
[21]Department of Plant Biotechnology and Bioinformatics, Ghent University and VIB Center for Plant Systems Biology, Gent, Belgium.
[22]College of Agriculture and Biotechnology, Zhejiang University. Hangzhou 310058, Zhejiang, China.
[23]The College of Life Sciences, Sichuan University. Chengdu 610207, China.

[24]Key Laboratory of Orchid Conservation and Utilization of National Forestry and Grassland
Administration at College of Landscape Architecture, Fujian Agriculture and Forestry University, Fuzhou 350002, China.
[25]State Key Laboratory of Systematic and Evolutionary Botany, Institute of Botany, Chinese Academy of Sciences, Beijing 100093, China.
[26]Department of Biology, East Carolina University, Greenville, NC 28590, USA.
[27]College of Life Sciences, South China Agricultural University, Guangzhou 510642, China.
[28]National Key Laboratory of Plant Molecular Genetics, Chinese Academy of Sciences Center for Excellence in Molecular Plant Sciences, Institute of Plant Physiology and Ecology, Chinese Academy of Sciences, Shanghai 200032, China.
[29]Department of Biology, University of Copenhagen, DK-2100 Copenhagen, Denmark.
[30]Florida Museum of Natural History, University of Florida, Gainesville, FL 32611, USA.
[31]Department of Biochemistry, Genetics and Microbiology, University of Pretoria, Pretoria, South Africa.
[32]Department of Biology, University of Florida, Gainesville, FL 32611, USA.

[#]These authors contributed equally.
[*]Corresponding authors, email: yang.liu0508@gmail.com; yvpee@psb.vib-ugent.be; dsoltis@ufl.edu; gongxun@mail.kib.ac.cn; liuhuan@genomics.cn; shouzhouz@szbg.ac.cn.

This PDF file includes:

Supplementary Notes
Supplementary References
Supplementary Figures. S1-S44

Contents

1. Introduction to cycads and *Cycas panzhihuaensis* 6
1.1 Cycads 6
1.2 *Cycas panzhihuaensis* 6
2. Sample collection 7
2.1 Sample collection for genome sequencing 7
2.2 Sample collection for transcriptome sequencing 7
2.3 Sample collection for generating the organellar sequences 7
2.4 Sample collection for sex determination system study 8
2.5 Sample collection for microRNA sequencing 8
3. Genome sequencing and assembly 8
3.1 Genome size estimation 8
3.2 Genome, transcriptome sequencing and assembly 9
4. Annotation and gene mining 10
4.1 Repetitive elements and comparison of repetitive elements among representative plants 10
4.2 MicroRNAs of *C. panzhihuaensis* and comparison of microRNAs among representative plants 10
4.3 Gene annotation of *C. panzhihuaensis* 11
5. Phylogeny of cycads and seed plants 11
5.1 Phylogenetic analyses using nuclear genes 11
5.2 Phylogenetic analyses based on organellar genes and the impact of RNA editing 12
5.3 Cyto-nuclear incongruence and investigation 14
5.4 Molecular dating and diversification rate analyses 16
5.5 Discussion of phylogeny and diversification 16
6. Comparative genomics of *C. panzhihuaensis* and other gymnosperms 18
7. Whole-genome duplication 19
7.1 Intra- and inter-genomic collinearity analyses 19
7.2 Identifying gene duplication events 19
7.3 Finding remnants of WGD through collinearity and synteny 20
7.4 Classification of the duplicated genes in *C. panzhihuaensis* 21
8. Transcription factors and phytohormones 21
8.1 Statistics of transcription factors among selected plants 21

8.2 Comparison of gene families operating in the biosynthesis and signaling networks of phytohormones...23
9. Comparative transcriptome analysis of tissue-specific expressed genes 25
10. Transcriptome landscape of ovule pollination, fertilization and development of *C. panzhihuaensis*..25
10.1 Expression patterns of genes related to ovule development...................25
10.2 Expression patterns of seed-specific genes 26
10.3 Phytohormone analyses of different developmental processes..............27
10.4 Metabolome analysis during developmental processes 27
10.5 The expression levels of phytohormone related genes in different seed developmental stages 28
10.6 Expansion of genes related to seed physiologies and development 29
11. Cell wall Carbohydrate-Active Enzymes (CAZymes) of *C. panzhihuaensis* ..32
11.1 Evolution of genes related to cell walls in *C. panzhihuaensis*.................33
11.2 Expansion of genes related to cell wall loosening, expansion and extensibilityin *C. panzhihuaensis* 34
11.3 Expansion of many other GH families in the *C. panzhihuaensis* genome 37
11.4 Expansion of genes related to lignin..39
11.5 Expansion of genes related to pollen tube physiology 39
12. Analysis of flagellar genes and their phylogenetic distribution.......................41
12.1 An overview of presence/absence of orthologogous genes related to the flagellar structure among representative plants..41
13. Sex chromosome assembly, annotation, and analysis.......................................41
13.1 Identification differentiated sex chromosome region in genome...........41
13.2 Male-specific region of Y chromosome (MSY) 42
13.3 Variant calling of Y chromosome...44
14. Pathogen and predator resistance 44
14.1 Immune receptor genes in the *C. panzhihuaensis* genome.....................44
14.2 Expansion of pathogenesis-related genes in the *C. panzhihuaensis* genome 45
14.3 Expansion of programmed cell death-related genes in the *C. panzhihuaensis* genome...45

14.4 Expansion of stress-tolerance related genes in the *C. panzhihuaensis* genome 46
14.5 Expansion of potato type protease inhibitor in *C. panzhihuaensis* 47
14.6 Expansion of immune signaling transduction related gene families 48
15. Analysis of the terpene synthase gene family in *C. panzhihuaensis* 49
16. Horizontal gene transfer and evolution of toxin genes in the *C. panzhihuaensis* genome 51
16.1 Identification of the horizontally transferred cytotoxin genes in *C. panzhihuaensis* 51
16.2 Experimental verification of the function of cytotoxin 52
17. Transcriptome, phytohormones, and metabolome analyses of roots of *C. panzhihuaensis* 53
17.1 Comparative transcriptomics among the primary root, precoralloid roots, and coralloid roots of *C. panzhihuaensis*, respectively 53
17.2 Phytohormone analyses among the primary root and coralloid roots *C. panzhihuaensis* with and without cyanobacteria 54
17.3 Metabolomic analyses among the primary root and coralloid roots of *C. panzhihuaensis* with and without cyanobacteria 55
17.4 Phylogenomic analyses of the symbiosis-related genes 55
17.5 Identification of the cyanobacterial-derived genes in the *C. panzhihuaensis* nuclear genome 56
18. Expansion of cell cycle related genes in *C. panzhihuaensis* 56
19. Expansion of other gene families 57

1. Introduction to cycads and *Cycas panzhihuaensis*

1.1 Cycads

Cycads are long-lived, woody, and dioecious gymnosperms, characterized by frond-like leaves, and develop cones and reproduce by seeds[1]. Today, they are one of the largest lineages of gymnosperms comprising ca. 360 living species[2] that are widely distributed across tropical and subtropical regions. Cycads were a major component of forests during the Mesozoic era[3]. However, most modern cycad species diverged within the last 50 my[4]. Unlike other seed plants, except *Ginkgo*, cycads rely on swimming sperm cells for sexual reproduction. Fertilization in cycads occurs in the archegonium, an organ first evolved in streptophyte algae (e.g., *Chara*) and maintained in sporic land plants (i.e., bryophytes and ferns) and gymnosperms, but lost in flowering plants[5]. Cycads develop a complex root system consisting of thickened and fleshy main roots and upright-growing and branched roots, the coralloid roots, which host symbiotic nitrogen-fixing cyanobacteria, a unique feature among gymnosperms[6].

1.2 *Cycas panzhihuaensis*

Extant cycads comprise two families, i.e., Cycadaceae and Zamiaecae. *Cycas* L. is the only genus of Cycadaceae and mostly occurs in tropical and subtropical areas of East and Southeast Asia, Oceania, and the surrounding islands, as well as East Africa and Australia. It is the largest genus of extant cycads with about 120 extant species[2]. *Cycas panzhihuaensis* L. Zhou & S. Y. Yang is an endangered *Cycas* species that is endemic to the dry-hot valley of the Jinsha River basin in Southwest China[7,8]. Morphologically, *C. panzhihuaensis* is recognized by its columnar, brown-gray, and scaly trunk, densely tomentose at the trunk's apex, pinnate leaves with flat to slightly curved leaflet margins, and the orange-red sarcotesta of the mature seed. Previous phylogenies and the current study all strongly supported *C. panzhihuaensis* (Section *Panzhihuaenses*) as sister to Section *Asiorientales*, which comprises *Cycas revoluta* and *Cycas taitungensis*[9,10]. However, the phylogenetic position of the *Panzhihuaenses*+*Asiorientales* clade is debated. Our transcriptomic data resolve this clade as sister to Section *Stangerioides* rather than as sister to Section *Wadeae* as suggested by Xiao and Moller[10] or to the remaining *Cycas* species as suggested by Liu et al.[9].

2. Sample collection

2.1 Sample collection for genome sequencing

Fresh megagametophytes from a more than 30-year-old cultivated female individual of *C. panzhihuaensis* from the Kunming Institute of Botany, Chinese Academy of Sciences, Yunnan, China, originally collected from Puduhe zones, Luqian county, Yunnan, China (PZHF03, voucher specimen kept in KUN), and fresh young leaves from a male individual of *C. panzhihuaensis* from living collection (PZHM1110, voucher specimen kept in SZG) in Fairy Lake Botanical Garden, Shenzhen, Guangdong (China), were sampled, treated with liquid nitrogen, and maintained at -80 °C until used for DNA extraction (Supplementary Table 1).

2.2 Sample collection for transcriptome sequencing

We collected 17 types of tissues from *C. panzhihuaensis* (i.e., pollen sac of male cone, microsporophylls of male cone, apical meristem of stem of male cone, cortex of stem, pith of stem, cambium of stem, mature leaf, young leaf, primary root, precoralloid roots, coralloid roots, megagametophyte, unpollinated ovule, early stage of pollinated ovule, late stage of pollinated ovule, fertilized ovule, and mature embryo) for transcriptome sequencing to enhance genome annotation and for comparative transcriptomics. Stem and root tissues of *C. panzhihuaensis* were used to generate full-length transcriptomes using the Oxford Nanopore platform (Supplementary Table 2). For phylogenomic analyses, we newly generated transcriptomes of 47 gymnosperms (Supplementary Table 2, voucher specimens kept in SZG). We also sequenced the transcriptomes of 341 cycad species, and most of the cycad materials were sampled from living collections from the Nong Nooch Tropical Botanical Garden, Chonburi, Thailand (Supplementary Table 2).

2.3 Sample collection for generating the organellar sequences

For ‘organellar’ phylogenetic analyses, we sampled 47 representative gymnosperms and three fern outgroups. Fresh leaves were collected from Fairy Lake Botanical Garden, Shenzhen, Guangdong, China, and preserved at -80 ℃. For these samples, DNA and RNA extraction were performed using the FastPure Plant DNA Isolation Mini Kit (Vazyme, Nanjing) and RNA-easy^{TM} Isolation Reagent (Vazyme, Nanjing),

respectively (Supplementary Table 3). Following the quality control steps, DNA and RNA samples were processed for library preparation and sequencing at BGI (Shenzhen) and Novogene Co., Ltd. (Beijing), respectively, yielding 2x150-bp reads amounting to 20 Gb and 12 Gb for each DNA and RNA library, respectively.

2.4 Sample collection for sex determination system study

To study the sex determination system of *C. panzhihuaensis*, we sampled 31 males and 31 females of *C. panzhihuaensis*. Fresh leaf samples were randomly collected from the *Cycas panzhihuaensis* National Natural Reserve at Sichuan, China. DNA extraction, library preparation, and whole-genome sequencing were conducted at China National GeneBank (CNGB), Shenzhen, China. Approximately 100 Gb of sequence data (2×100 bp paired-end reads) were generated on a DIPSEQ-T1 sequencing platform for each sample, resulting in an average of 10× sequencing depth. In addition, one male and one female sample were sequenced to a greater depth of 30× (see Supplementary Table 4 for details).

2.5 Sample collection for microRNA sequencing

Fresh leaves obtained from a more than 30-year-old cultivated male *C. panzhihuaensis* from Fairy Lake Botanical Garden, Shenzhen, Guangdong, China were used for miRNA library construction and sequencing (Supplementary Table 5). The tissues were treated with liquid nitrogen and stored at -80 ℃ until used for RNA extraction.

3. Genome sequencing and assembly

3.1 Genome size estimation

The *C. panzhihuaensis* genome size was estimated by both flow cytometry and *K*-mer analysis (Supplementary Fig. 1). Estimates via flow cytometry were obtained by comparison of *C. panzhihuaensis* with the standard of *Zea mays* (B37-type) (n = 2.3 Gb)[11]. The average genome size of female *C. panzhihuaensis* was estimated as 10.98 Gb/1C and that of male *C. panzhihuaensis* was estimated as 10.78 Gb/1C (Supplementary Table 6). Former flow cytometry analyses suggested that *Cycas* has the smallest genomes (13.1 Gb/1C on average) among all cycad genera (ranging from 19.4 to 31.7 Gb/1C on average)[12], and our result indicated *C. panzhihuaensis* has a

genome smaller than any *Cycas* species with a genome size yet reported (https://cvalues.science.kew.org/). For the *K*-mer-based estimation, the peak of *K*-mer frequency (M) was determined by the real sequencing depth of the genome (N), read length (L), and the length of the *K*-mer (*K*) following the formula: $M = N \times (L - K + 1)/L$. This formula enables an accurate estimation of *N* and hence an estimation of the genome size for homozygous diploid or haploid genomes. Compared to the estimates from flow cytometry, we identified 8,930,138,579 *K*-mers with *K*-mer size 21. The genome size was estimated to be ~11.69 Gb (1C), and the repeat ratio was estimated to be 66.58%.

3.2 Genome, transcriptome sequencing and assembly

The preliminary assembly of the Nanopore long-read data yielded a total of 5,123 contigs with a contig N50 length of 12.10 Mb. Hi-C data were used to execute Hi-C chromosome conformation in conjunction with 3d-dna algorithm (Supplementary Fig. 2). As a result, we obtained the final genome assembly with a size of 10.48 Gb, organized into 11 pseudo-chromosomes, covering >95% of the estimated *C. panzhihuaensis* genome (Supplementary Table 7).

Transcriptome sequencing included short-read data generated by the MGI-seq platform and long-read data by the Nanopore platform. For transcriptome sequencing, RNA was extracted by the Spectrum Total RNA Plant Kit (Sigma-Aldrich). The transcriptome libraries were prepared by BGI and sequenced either on an Illumina HiSeq or MGI-SEQ platform. In addition, the full-length transcriptomes were sequenced using the Nanopore platform. The short reads were filtered by SOAPfilter and mapped to the genome to calculate the gene expression level using Express. The long reads were mapped to the gene set to filter the unexpressed genes. The quality of the assembly was supported by the high genome coverage of 99.3% (MGI-seq data) and 98.6% (Nanopore data), and mapping rates of 93.3% (MGI-seq data) and 90.9% (Nanopore data) (Supplementary Table 8).

For organelle genome sequencing, DNA and RNA extraction were performed using the FastPure Plant DNA Isolation Mini Kit (Vazyme, Nanjing) and RNA-easy™ Isolation Reagent (Vazyme, Nanjing). Following the quality control steps, DNA and RNA samples were processed for library preparation and sequenced either on the Illumina HiSeq or MGI-SEQ platform.

4. Annotation and gene mining

4.1 Repetitive elements and comparison of repetitive elements among representative plants

The genome of *C. panzhihuaensis* contained a large proportion (76.14%) of repeat sequences (Supplementary Table 9), a proportion that is almost similar to that of *Ginkgo* (i.e. 75.34%)[13], higher than that of conifers (i.e., 54.52%–54.82%), but lower than that of Gnetales (i.e., 85.93%)[14]. Among the TEs of *C. panzhihuaensis,* long terminal repeat (LTRs) retrotransposons and long interspersed elements (LINEs) were the predominant types of TEs, together representing 68.97% of the assembly. An estimation of divergence time indicated that the LTR expansion in the *C. panzhihuaensis* genome occurred around 17–24 mya, which is similar to previous studies that suggested that LTR-RTs of conifers, *Ginkgo biloba*[13], and Gnetales[14] accumulated steadily over the last ~25 mya, especially between 16 and 24 mya, a process possibly contributing to their large genome sizes (Supplementary Fig. 16).

4.2 MicroRNAs of *C. panzhihuaensis* and comparison of microRNAs among representative plants

Total RNA was extracted from young leaves of *C. panzhihuaensis,* using the TRIzol reagent. The quantity and purity of the total RNA were analyzed using a NanoDrop 2000 spectrophotometer. The small RNA libraries were constructed using the NEBNext Small RNA Library Prep Set for Illumina (NEB, E7300S) according to the manufacturer's instructions. After quantification via qRT-PCR, the libraries were single-end sequenced on an Illumina MiniSeq System (Illumina, San Diego, CA). We then performed miRNA prediction using the miRDeep2 software[15] with miRbase v22 (http://www.mirbase.org) as the reference database. We filtered the sequences with more than five mapping hits according to the miRDeep2 results. Finally, the analysis predicted 906 miRNA sequences in total, clustered into 54 miRNA families (Supplementary Table 10). The prediction of gene targets by miRNAs was performed with TargetFinder[16], and 3,515 gene targets were annotated. Note that only young leaves were used for microRNA sequencing, and therefore the families of microRNAs detected here may thus be incomplete.

4.3 Gene annotation of *C. panzhihuaensis*

Based on a combination of homology-based, *ab initio*, and transcriptome-based approaches, we predicted 32,353 protein-coding genes in the *C. panzhihuaensis* genome, of which 98% were supported by the transcriptome data. Functional annotations showed that 67.1% of the protein-coding genes have known homologs in protein databases (Supplementary Table 11). Interestingly, we found an aberrant genomic area on chromosome 4 with relatively higher GC content than other genome areas. Further gene ontology (GO) enrichment analyses showed that genes in this region might be involved in the processes of oxidative phosphorylation, photosynthesis, glyoxylate and dicarboxylate metabolism, as well as ribosome synthesis.

5. Phylogeny of cycads and seed plants

To reconstruct the phylogeny and diversification age of cycads and seed plants, we assembled seven independent data matrices: 1) a dataset that consists of 3,282 orthologous low-copy nuclear genes derived from 15 vascular plant genomes; 2) a dataset of 1,569 orthologs from 90 seed plant transcriptomes; 3) a dataset of 82 plastid genes from 72 vascular plants; 4) a dataset of 42 mitochondrial genes from 72 vascular plants; 5) a plastid dataset considering the impact of RNA editing; 6) a mitochondrial dataset considering the impact of RNA editing; and 7) a dataset of 1,170 low-copy nuclear genes sampled from 342 taxa including 339 cycad species (Supplementary Tables 12–15). A concatenation of each dataset was analyzed using the Maximum Likelihood method using IQTREE, and a coalescent tree was also inferred for each nuclear data set using ASTRAL (Supplementary Table 16; Supplementary Fig. 3). Molecular dating analyses were performed using MCMCTREE, and 27 fossils were used to calibrate the seed plant species tree and six fossils for the cycad species tree (Supplementary Table 17).

5.1 Phylogenetic analyses using nuclear genes

The 15-taxon phylogenetic reconstruction of representative vascular plants based on 3,282 mostly single-copy nuclear genes (75% taxon occupancy) recovered a robust topology (Supplementary Fig. 4). For the gymnosperm clade, *Cycas* and *Ginkgo* formed a robust sister clade emerging from the earliest split, with cupressophytes sister

to a clade of Pinaceae and gnetophytes. Species tree inferences with STAG[17] based on 3,002 low-copy nuclear genes (with fewer than 4 copies) with full taxon occupancy yielded identical topologies, albeit with overall lower support values for each branch. Phylogenomic reconstruction of the extended 90-taxon nuclear dataset based on 1,569 nuclear single-copy genes is mostly congruent with the topology found with 15 taxa (Supplementary Fig. 5)[18].

As the current study focused on the evolutionary relationships of gymnosperms, we examined the monophyly of nine groupings in more detail (see main text, Fig. 1 for details): seed plants, angiosperms, gymnosperms, cycads-*Ginkgo,* cycads-other gymnosperms (cycads alone), gnetophytes-other gymnosperms (gnetophytes alone), gnetophytes-Pinaceae (gnepine), gnetophytes-cupressophytes (gnecup), and gnetophytes-Pinaceae+cupressophytes (gnetifer). Although the coalescent species tree inferred by ASTRAL[19] and best ML tree inferred from concatenated datasets unanimously recovered maximal support for the major groups of gymnosperms, we observed strong gene tree incongruences among individual gene trees for the clustering of different groups of gymnosperms, and the placement of gnetophytes seemed especially problematic (Supplementary Fig. 6). The sister relationship between gnetophytes and Pinaceae appears to gain a similar level of gene tree support as that of gnetophytes and Pinaceae+cupressophytes, followed by that of the gnetophytes and other gymnosperms, whereas the sister-group relationship of gnetophytes and cupressophytes gains the least support. Quartet support for six branches also revealed strong conflicts for the clade leading to cupressophytes and gnetophytes+Pinaceae, gnetophytes and Pinaceae, and cycads and *Ginkgo*, respectively. The sister relationship of gnetophytes and Pinaceae revealed similarly high quartet support as that of the gnetophytes and Pinaceae+cupressophytes, whereas the sister relationship of gnetophytes and cupressophytes received lower quartet support, which is consistent with the gene tree conflict analyses (Supplementary Fig. 7). For the phylogenetic position of cycads, the sister relationship to *Ginkgo* received the highest support, whereas that to other gymnosperms also received some support, consistent with the gene tree statistical analyses.

5.2 Phylogenetic analyses based on organellar genes and the impact of RNA editing

The pipeline HybPiper[20] was used to extract the draft mitochondrial scaffolds from the genome skimming data of gymnosperms with the mitochondrial protein sequences from available gymnosperms as baits. NOVOPlasty[21] was used to assemble the plastid genomes for the focal gymnosperms with the available gymnosperm plastid genomes as references. Tophat2[22] was used to map the clean RNA-seq reads to the concordant organellar draft assemblies with default settings. Bowtie2[23] was used to map the DNA-seq reads to the concordant organellar draft assemblies with default settings. Samtools and Bcftools were used to call SNPs with the variant base reaching a read depth of at least 10%. The RNA-seq-identified SNPs (raw RNA editing sites) were filtered against the DNA-seq-identified genomic SNPs to summarize the final RNA editing sites.

For phylogenetic reconstruction of organellar genes, four datasets were compiled: the mitochondrial genomic, mitochondrial RNA editing site modified dataset, plastid genomic, and plastid RNA editing site modified datasets. IQTREE2 was used to infer the Maximum Likelihood (ML) trees with gene concordance factors and site concordance factors, with an initial partitioning scheme of gene and codon positions, combining ModelFinder[24], tree search, and ultrafast bootstrap. We produced draft mitochondrial and plastid genome assemblies with Next Generation Sequencing (NGS) data and annotated empirical RNA editing sites using Long non-coding RNA (Lnc-RNA) sequencing data. Combined with organellar genomes downloaded from GenBank, we compiled a comprehensive organellar dataset with empirical RNA editing sites annotated for 72 vascular plant representatives (Supplementary Fig. 8). The RNA editing site annotation for gymnosperms might therefore be incomplete due to the fragmentary nature of genes and genomes. Our study identified a total of 11,497 mitochondrial (average: 245) and 3,652 plastid (average: 78) RNA editing sites in the protein-coding genes of 47 gymnosperm organellar genomes. Plastid RNA editing is generally less abundant compared to that of the mitochondrial protein-coding genes in seed plants, whereas *G. biloba* has a similar level of plastid and mitochondrial RNA editing. In gymnosperms, gnetophytes have the fewest RNA editing sites, whereas Pinaceae, cycads, and *Ginkgo* have the most, with Cupressophytes having an intermediate number of RNA editing sites (Supplementary Fig. 8).

Organellar phylogenomic reconstruction recovered consistent placements of gnetophytes as the sister group of cupressophytes, but contrasting results regarding the placements of cycads (Fig. 1c, Supplementary Figs. 9–10). Cycads are either resolved

as sister to *Ginkgo* in plastid phylogenies (Supplementary Fig. 9) or sister to the rest of gymnosperms in mitochondrial phylogenies (Supplementary Fig. 10). Both placements received maximal support in IQTREE[25] ML analyses. Inferences from the RNA editing corrected organellar dataset did not differ from the original topology but revealed differences in the support for different branches. For the plastid phylogeny (Supplementary Fig. 9), the ML tree based on an RNA editing corrected dataset showed reduced support for the sister relationship of cycads and *Ginkgo*, i.e., BS (Bootstrap support) declined from 99% to 63%, and GCF (Gene Concordant Factor) decreased from 41% to 33%. For the mitochondrial phylogeny (Supplementary Fig. 10), the RNA editing corrected tree shows weak support for the sister relationship of gnetophytes and cupressophytes compared with that of the original mitochondrial tree, i.e., BS and GCF decreased from 100% and 41%, respectively, in the original tree to 97% and 33%, respectively, in the RNA editing corrected tree.

5.3 Cyto-nuclear incongruence and investigation

DiscoVista was used to summarize/visualize the discordance among the phylogenetic trees. The software simply compares the topologies and support among trees and visualizes the conflicts based on the thresholds defined by the user. In the current study, we defined strongly supported, weakly supported, weakly rejected, and strongly rejected relationships based on our own thresholds (see the legend of Fig. 1 for details). PhyloNet[26] was used to construct the species network using rooted nuclear gene trees with the command InferNetWork_MPL. The R package PHYBASE[27] was used to simulate 400,000 gene trees under the hypothesis of incomplete lineage sorting (ILS) for PhyloNet analyses. The gene tree topologies for the observed and the simulated trees were counted and summarized using TWISST[28]. This part of statistical analyses mainly followed Yang et al.[29] and Wang et al.[30].

Cyto-nuclear incongruence of the deep phylogenetic relationships of gymnosperms mainly pertain to the placements of gnetophytes and cycads (Fig. 1c). Gnetophytes are resolved as sister to the Pinaceae in the nuclear phylogeny and sister to the cupressophytes in the organellar phylogeny. Cycads are sister to *Ginkgo* based on both nuclear and plastid data but sister to all other extant gymnosperms based on mitochondrial data. Nuclear gene trees also show low quartet support for these lineages, suggesting high gene tree discordance. These incongruences persisted with extended

sampling (Fig. 1c, Supplementary Figs. 5, 9, and 10) and could not be explained by RNA editing sites. Hybridization and ILS are frequently evoked to explain such incongruences. To this end, we performed a PhyloNet analysis (InferNetwork_MPL and CalGTProb) and an ILS simulation analysis to examine the impact of hybridization and ILS. PhyloNet results (Supplementary Fig. 11) favored one scenario (Reticulation = 4) in which gnetophytes are of hybrid origin from the ancestor of Pinaceae and the ancestor of gymnosperms. However, extinctions, that may have widely happened during the evolution of gymnosperms may also impact the molecular phylogentic analyes and be responsible for the incongruences observed here.

The gene tree simulation results of the nuclear, plastid. DNA, and mitochondrial DNA revealed similar topological patterns (Supplementary Fig. 12a), suggesting the importance of ILS in the evolutionary history of gymnosperms. The topology frequencies of the observed nuclear gene trees is significantly correlated with that of simulated nuclear trees (Pearson coeffiecient = 0.538 , $P<0.01$), and the topology frequencies of the simulated nuclear gene trees is significantly correlated with that of the plastid (Pearson coeffiecient = 0.894 , $P<0.01$) and mitochondrial (Pearson coeffiecient = 0.415 , $P<0.01$) trees (Supplementary Fig. 12b). The observed and simulated gene trees show similar topological patterns (Supplementary Fig. 13). Of the 105 different topologies, 62.24% and 10.02% of the observed trees show support for the nuclear tree and mitochondrial tree, respectively, and for the simulated tree set, these propotions are 43.87% and 13.95%, respectively (Supplementary Fig. 13b).

For the placements of the two difficult lineages, i.e., gnetophytes and cycads, the ratio of the trees supporting three major alternative topologies between oberserved and simulated datasets of nuclear, mitochondrial, and plastid genes were also summarized (Supplementary Fig. 13c,d). For the topological frequencies of these six relationships, the observed nuclear tree frequencies are not correlated with any of the simulated ones, whereas the topological frequencies of the six relationships among the simulated tree sets are strongly and significantly correlated. The sister relationship of gnetophytes and Pinaceae, and of cycads and *Ginkgo*, gained the highest support across four categories of trees (Supplementary Fig. 13c,d).

Although ILS may have played an important role in the evolution of gymnosperms, ancient hybridization cannot be completely ruled out. Considering the incongruent phylogenetic relationships of the plastid and mitochondrial results regarding cycads and

Ginkgo, as well as the above-mentioned lack of significant correlations between simulated and observed tree topology distributions regarding the placements of these two lineages, ancient hybridization might have also played a role. Indeed, many gymnosperms (e.g., Cephalotacaceae, Pinaceae, Podocarpaceae, Tacaceae) have different systems for cytoplasmic inheritance for the two organelles, although extant cycads, *Ginkgo*, and gnetophytes have consistent maternal inheritance of both plastids and mitochondria[31]. Ancient gene flow among gymnosperm lineages with different cytoplasmic inheritance systems would lead to the gene tree incongruences between mitochondrial and plastid data in phylogeny reconstruction, which may explain the conflict between the mitochondrial and plastid/nuclear results, in terms of the phylogenetic position of the cycad clade.

5.4 Molecular dating and diversification rate analyses

Our chronogram of 339 cycad species with a nearly complete sampling of extant cycads pointed to a Carboniferous origin (268.87–365.96 mya, Median: 324.52 mya) of cycads (Supplementary Fig. 14). The split between Cycadaceae and Zamiaceae occurred in the Mid-Permian (231.87–332.65 mya, Median: 268.06 mya). The crown ages of the extant most species-rich genera (i.e., *Cycas*, *Macrozamia*, *Ceratozamia*, and *Zamia*) are relatively younger and clustered in time, at around 19–20 Ma, with the exception of *Encephalartos* with a crown age of ~13 myaMa. BAMM[32] diversification analysis identified six major shifts of net diversification rates across cycads, three diversification rate increases for crown *Cycas*, *Ceratozamia*, and *Zamia* at 19–20 mya, and three diversification rate increases within *Cycas*, *Macrozamia*, and *Encephalartos* at 11–13 mya. *Cycas*, *Ceratozamia*, and *Zamia* from tropical rain forests (per their present distributions) tend to have undergone rapid radiations near the warmer period of the Mid-Miocene climate optimum at around 17–18 mya, whereas *Cycas*, *Macrozamia*, and *Encephalartos* from more temperate (per their present distributions) and dry areas demonstrate rapid radiations during the cooling period in the Neogene[33].

5.5 Discussion of phylogeny and diversification

Nuclear phylogenomic reconstructions support the relationship of ((*Ginkgo*,cycads),((Pinaceae,gnetophytes),cupressophytes)), despite some gene tree conflict for the nodes involving gnetophytes and cycads, respectively. In contrast to the

nuclear-based inference of Pinaceae and gnetophytes as sisters, organellar data (both mitochondrial and plastid) support the sister relationship of gnetophytes and cupressophytes. In addition, mitochondrial data strongly support cycads as the sister to the rest of the gymnosperms, whereas plastid data agree with nuclear data in supporting the sister relationship of cycads and *Ginkgo*. The strong cyto-nuclear (and mito-plastid) incongruences cannot be reconciled with RNA editing corrected datasets. Involving RNA editing sites does not change the original organellar tree topology, whereas the GCF support of cupressophytes + gnetophytes decreased from 41% to 33%, 35% to 32% in the mitochondrial and plastid trees, respectively. The sister relationship of *Ginkgo* and cycads is more weakly supported (BS=63%, GCF=33%) in the mitochondrial RNA editing revised tree compared to that of the original tree (BS=99%, GCF=41%). The sister relationship of cycads and *Ginkgo* has been proposed by many former molecular phlygentic analyses and confirmed by recent large scale phylogenomic studies, either using nuclear[18,34,35] or chloroplast gene sequences[36].

Hybridization and ILS were thus examined in current datasets to explain the cyto-nuclear incongruences in gymnosperm backbone phylogeny. The conflicting placements of gnetophytes could possibly be explained by ancient hybridization as evidenced by PhyloNet results. The inconsistent placements of cycads might be explained by ILS, as our analyses revealed significant correlation of gene tree frequencies of all 105 possible topologies between the simulated and the observed datasets of individual gene trees. Moreover, the strong correlation between tree topology distribution patterns of the three simulated tree sets of nuclear, mitochondrial, and plastid genes and the similar percentages of gene trees supporting nuclear and mitochondrial trees between the nuclear observed and simulated gene trees, indicate that ILS might have played an important role in the evolution of gymnosperms. Again, the widely happened extinctions may be also be responsible for the incongruences in our phylogenetic analyses of gymnosperms.

Dating analyses based on comprehensive transcriptomic data with complete taxon sampling from cycads yielded a robust time frame for inference of diversification. Our study suggests that extant species-rich genera of cycads emerged from rapid radiations ranging from 11–20 mya, which is consistent with previous studies based on fewer samples[4,37].

6. Comparative genomics of *C. panzhihuaensis* and other gymnosperms

In order to clarify gains and losses of genes and the expansion and contraction of gene families during the evolution of vascular plants, we selected 11 genomes covering angiosperms, gymnosperms, ferns, and lycophytes. In order to avoid bias due to the difference in the number of species, we matched the number of angiosperms and gymnosperms to that in Figure 2a. The three angiosperm species represent the basalmost angiosperm branches, monocotyledons, and eudicots, while the five gymnosperm species represent five distinct groups: cycads, *Ginkgo*, Gnetophytes, Conifer I, and Conifer II. Orthofinder was used to generate the orthogroups. The single-copy orthogroups were then extracted as candidates for constructing phylogenetic trees. Multiple sequence alignment was performed using MAFFT for each orthogroup. For the multiple sequence alignments, only the loci represented by more than half of all species were retained for phylogeny reconstruction. The final maximum likelihood tree was generated by RAxML with the PROTCATGTR model and 500 bootstrap replicates.

The phylogenetic analyses placed *Cycas* and *Ginkgo* as a clade sister to the remaining gymnosperms. The comparative analyses of *Cycas* and other gymnosperms, angiosperms, and non-seed plants indicated that the *Cycas* genome has some unique features. Ancestral and lineage-specific gene families were inferred from the orthogroups across the phylogeny. There are many gained or expanded gene families throughout the evolution of green plants[18]. Following the origin of seed plants, the gained gene families mainly include development of seed (Supplementary Fig. 15), secondary growth, pollen tube development, phenylpropanoid biosynthesis, and glycerophospholipid metabolism, etc. (Supplementary Tables 18–20), while the common ancestor of gymnosperms gained gene families that include plant-pathogen interaction, plant hormone signal transduction, and most importantly, flavonoid biosynthesis and oxidative phosphorylation, before the divergence of *Ginkgo* and *Cycas* from the rest of the gymnosperms. There was also loss of some pathways in other gymnosperm clades, like selenocompound metabolism and protein export pathway. We used a Venn diagram to summarize our results (Supplementary Fig. 16). In total, 10,362 orthogroups were shared between *Cycas* and *Ginkgo*, mostly enriched in photosynthesis and plant hormone signal transduction pathways. Moreover, 11,546 orthogroups were unique to *Cycas*, mainly enriched in pyrimidine metabolism and plant-pathogen interaction pathways (Supplementary Table 21). Gymnosperms and angiosperm

showed a different pattern in shared genes. Angiosperms share those land-plant (embryophyte) most commonly shared genes, while gymnosperms shared most gymnosperm-specific genes (Supplementary Fig. 16b). Finally, gene structure was compared among species. Interestingly, *Cycas* possesses the longest average intron length and gene length. To detect the composition of introns, we compared some model plant species with *Cycas* with respect to the intron sequences (Supplementary Table 22). Although *Ginkgo* and *Cycas* retained the longest introns compared to other species, *Cycas* contains more unknown sequences than *Ginkgo* in the intron. Furthermore, we also compared the repeat divergence rate among gymnosperm species, and our analyses revealed a similar pattern among species, which also matched the insertion time for the LTR sequences over their whole genomes (Supplementary Fig. 18). Therefore, the unknown sequences in the introns may have contributed greatly to genome size.

There are 1,934,254 repetitive elements in the *C. panzhihuaensis* genome, based on transcriptome data from eight tissues, 0.06% of which are expressed. All types of repeat elements including DNA, LINE, SINE, LTR, and simple repeats were expressed but with different patterns in the eight tissues considered. The most abundant LTR/*Copia* and LTR/*Gypsy* were widely expressed at high levels across all tissues (Supplementary Fig. 19; Supplementary Table 23).

7. Whole-genome duplication

7.1 Intra- and inter-genomic collinearity analyses

The intra- and inter-genomic synteny analyses were conducted using MCscanX[38], with default settings (Supplementary Fig. 20). Synonymous substitutions per synonymous site (*K*s) estimates for pairwise comparisons of paralogous genes located on syntenic blocks were obtained using the Nei-Gojobori method[39] implemented in the yn00 program of the PAML package[40].

7.2 Identifying gene duplication events

To identify whole-genome duplication events during the evolution of vascular plants, we selected 15 genomes covering angiosperms, gymnosperms, ferns, and a lycophyte, and a transcriptome from an additional cycad species. Orthogroups were constructed with Orthofinder. The single-copy orthogroups were then extracted as the candidates

for constructing phylogenetic trees. Multiple alignments were performed using MAFFT for each single-copy orthogroup. For the results of multiple sequence alignments, only the loci represented by more than half of all species were retained for phylogenetic tree constructions. The final maximum likelihood tree was generated by RAxML with the PROTCATGTR model and 500 bootstrap replicates. We used a rigorous standard for the gene tree-species tree reconciliation as previously described[41,42] to accurately identify gene duplications (Supplementary Figs. 21, 22; see detals in Methods section in main text). We have two basic requirements for determining a reliable duplication event: 1) at least one common species' genes occurred in two child branches; and 2) both the parental node and one of the child nodes have bootstrap values equal to or greater than 50%. More detailed methods can be found in Wu et al.[42]. Given that tandem duplications may affect the results, we removed them based on their chromosomal positions, defining tandem duplicates as two homologous genes located within five genes on a chromosome. After filtering, 9,545 gene families with more than four sequences and at least one sequence from the outgroups (i.e., *Azolla filiculoides*, *Salvinia cucullate,* and *Selaginella moellendorffii*) were used to construct maximum likelihood trees and to score gene duplication events. To validate our results based on broad sampling across vascular plants but limited sampling of gymnosperms, we also analyzed a dataset with a broader sampling. The dataset consists of a mixture of 29 genomes and 61 transcriptomes that focuses on the sampling of gymnosperms, i.e., with 16 species of Cupressaceae, four of Taxaceae, six of Podocarpaceae, ten of Pinaceae, 11 of Zamiaceae, and ten of Cycadaceae, as well as one species each of Araucariaceae, Welwitschiaceae, Gnetaceae, Ephedraceae, and Ginkgoaceae, covering all the families in gymnosperms. For this dataset, 8,891 selected gene families were used to construct gene family trees and to score gene duplications.

7.3 Finding remnants of WGD through collinearity and synteny

Through gene tree - species tree reconciliation, we can often identify, with some confidence, which node or branch in a phylogeny shows an increased number of gene duplications, possibly supporting a WGD event. However, the strongest evidence for large-scale duplications such as whole-genome duplications is through within-genome collinearity or within-genome synteny. To this end, we further investigated if

duplicated genes can be found on collinear or syntenic blocks (Supplementary Fig. 23; Supplementary Tables 24–25).

7.4 Classification of the duplicated genes in *C. panzhihuaensis*

To investigate the characteristics of duplicated genes of the *C. panzhihuaensis* genome, we categorized duplications using DupGen_finder[43]. We identified 24,239 duplicated genes that were classified into five different categories using DupGen_finder: 1,443 whole-genome duplicates (WGD duplicates, 18.4%), 5,333 tandem duplicates (TD, 14.4%), 2,914 proximal duplicates (PD, 11.3%), 1,298 transposed duplicates (TRD, 19.4%), and 13,251 dispersed duplicates (DSD, 25.6%) (Supplementary Table 26 and Fig. 24).

8. Transcription factors and phytohormones

8.1 Statistics of transcription factors among selected plants

The *C. panzhihuaensis* genome encodes 1,732 transcription factors/transcription regulators (TF/TR), which were classified into 105 (out of 107) types of TF/TR genes annotated in selected plants (see Supplementary Table 25 for details), by TAPscan and rules set for TF/TR annotation (https://plantcode.online.uni-marburg.de) (Supplementary Table 27). The TF/TR numbers of *C. panzhihuaensis* were closer to that of *Sequoiadendron giganteum* (1,795), but remarkably higher than that of *G. biloba* (1,287) and *Gnetum montanum* (1,290). All Conifer I species showed a huge expansion of TF/TR genes, especially *Pinus taeda*, which encodes 3,335 TF/TRs which is almost two-fold higher than the number of TF/TR in *C. panzhihuaensis*. Only one type of TF/TR gene family (i.e., *ULT*) was identified to have originated from *Cycas* and *Ginkgo*. *C2C2_YABBY* homologs are found in red and green algae (e.g., *Porphyra umbilicalis*, *Spirogloea muscicola*, and *Chara braunii*), but evolutionarily lost in seedless plants, and they were proposed to be uniquely distributed in seed plants among land plants[44], However, recent studies found *YABBY* in hornworts and and also in the lycophyte *Huperzia* [45]. We performed gene expression analysis among different tissues of *C. panzhihuaensis* and found that three copies of *YABBY* were mainly expressed in mature leaf, apical meristem, microsporophylls, pollen sac, and embro (Supplementary Fig. 27). In all, 13 types of TF/TR gene families (*MYB*; *mTERF*; AN1 and A20 type

Zinc fingers; E2F/DP transcription factor; TFb2; RB; CCAAT_HAP2; HD_WOX; Whirly; HD_PLINC; AS2/LOB; bHLH_TCP; GeBP) exhibited remarkable expansion in *C. panzhihuaensis* compared to bryophytes, lycophytes, and ferns. However, *RRN3* homologs, which are generally found in all representative seedless plant genomes and other gymnosperms, could not be identified in either *C. panzhihuaensis* or *G. montanum*, suggesting the loss of *RRN3* in *C. panzhihuaensis* and *G. montanum*. Considering the sister relationship between *Ginkgo* and *Cycas*, we compared the TF/TRs between them and found a two-fold higher number of 18 types of TF/TRs (Zinc finger, AN1 and A20 type; CCAAT_Dr1; RWP-RK; E2F/DP; ARID; Sir2; TFb2; GIF; Argonaute; PLATZ; Coactivator p15; ABI3/VP1; Whirly; HD_DDT; PcG_EZ; LUG; PcG_FIE; HRT) in *Cycas* compared to *Ginkgo*, while 6 types of TF/TRs (Sin3; Zinc finger, ZPR1; MADS_MIKC; RRN3; CAMTA; HD_KNOX2 (Supplementary Fig. 25)) of *Cycas* showed a two-fold lower number than *Ginkgo*. In addition, PcG_FIE (TR) and HRT (TF) were evolutionarily lost in *Ginkgo*. Notably, two types of TFs (E2F and Zinc finger, AN1 and A20 type) showed specific expansion in the *C. panzhihuaenesis* genome. MADS-box genes encode a family of transcription factors that control diverse developmental processes, especially the formation of the flower in angiosperms. We identified 14 MADS-box genes in the *C. panzhihuaensis* genome, compared with 27 in *Ginkgo*. Gymnosperms have many fewer MADS-box genes than angiosperms, and the number MADS-box genes encoded in *Cycas* is among the lowest of all gymnosperms. The ABCDE model genes determine floral organ formation in flowering plants. We found that the *APETALA1* gene of class A, *APETALA3* of class B, *SEEDSTICK* and *AGAMOUS* genes of class C, *SHATTERPROOF1* and *SHATTERPROOF2* genes of class D, and *SEPALLATA1-4* genes of class E were all missing in *C. panzhihuaensis* (Supplementary Fig. 26). A huge expansion via tandem repeats of E2F transcription factors was identified in the *C. panzhihuaenesis* genome, and we further performed phylogenetic analysis on the E2F transcription factors. Interestingly, *C. panzhihuaensis* not only contains A-F types of E2F, it also encodes a new group of gymnosperm-specific “E2F” (Supplementary Fig. 29). The phylogeny of AN1 and A20 type Zinc finger genes revealed three groups of AN1 and A20 type Zinc finger, and the expanded AN1 and A20 type Zinc finger of *C. panzhihuaensis* is type 1 (Supplementary Fig. 28).

8.2 Comparison of gene families operating in the biosynthesis and signaling networks of phytohormones

We explored the evolution of phytohormone biosynthesis and transduction pathway-related genes (Supplementary Table 28). Compared to seedless plants, the number of genes related to phytohormone production and signaling in gymnosperms showed remarkably greater expansion. Notably, *SAUR* genes from the auxin signaling pathway and *GID2* genes from the gibberellin signaling pathway significantly expanded in the *C. panzhihuaensis* genome, compared to early embryophytes (the number was two-fold higher than that of seedless plants). We also compared the gene number between *Ginkgo* and *Cycas*. In the abscisic acid signaling pathway, *Cycas* contained significantly higher numbers of *ABI3* and *PYL* homologs than that of *Ginkgo*. For the auxin signaling pathway, the number of SAUR genes in *Cycas* was also higher than that of *Ginkgo*; however, *Ginkgo* contains three times more *GH3* homologs than *Cycas*. In addition, more *CRE* homologs of the cytokinin signaling pathway could be identified in the *Cycas* genome than in *Ginkgo*. In the ethylene signaling pathway, *Ginkgo* contained five gene copies of *ASK1*, a number similar to the seedless plants, but half those found in *Cycas* (10 copies). However, *Ginkgo* encoded four times more PIF genes (gibberellin signaling pathway) than *Cycas*. In the salicylic acid signaling pathway, *NPR3/4* homologs could not be found in *Cycas*, ferns, or lycophytes; however, both *G. montanum* and *G. biloba* encode five *NPR3/4* homologs. Additionally, we found 14 copies of *D14* homologs in the *G. biloba* genome, which is two-fold higher than in *Cycas*.

Regarding phytohormone biosynthesis pathways, three gene families (phytoene desaturase from the abscisic acid biosynthetic pathway, pyruvate decarboxylase from the auxin biosynthetic pathway, and gibberellin 20-oxidase homologs from the gibberellin biosynthetic pathway) were significantly expanded in the *C. panzhihuaensis* genome, compared to seedless plants. A total of 22 gene families corresponding to eight phytohormone biosynthetic pathways showed remarkable difference between the *Cycas* and *Ginkgo* genomes. In the abscisic acid biosynthetic pathway, the *Ginkgo* genome has two-fold more copies of abscisic-aldehyde oxidase and aanthine dehydrogenase than that of *Cycas*. In contrast, *Cycas* encoded more β-carotene hydroxylase genes than *Ginkgo*. The 44 β-carotene hydroxylase homologs were found in the *Pinus lambertiana* genome. For the auxin biosynthetic pathway, we identified

fewer amidase and *YUCCA* genes in the *Ginkgo* genome. Interestingly, *DWF4* homologs from the brassinosteroid biosynthetic pathway could not be identified in *Ginkgo*, although all other gymnosperms contain this gene, suggesting a possibly incomplete brassinosteroid biosynthetic pathway in *Ginkgo*. As in seedless plants, we could not identify isopentenyl transferase (IPT) homologs in the genomes of *C. panzhihuaensis*, *G. biloba,* and *S. giganteum*, and it seems that IPT might have been lost in these three lineages during the evolution. The loss of IPT homologs suggested the incomplete biosynthetic pathway of transzeatin and dihydrozeatin. Surprisingly, phytohormone measurement of *C. panzhihuaensis* tissue revealed it is still able to produce transzeatin, suggesting *C. panzhihuaensis* and perhaps other gymnosperms may use alternative ways to perform biosynthesis of transzeatin and dihydrozeatin. Although they have an incomplete transzeatin and dihydrozeatin biosynthetic pathway, *C. panzhihuaensis*, *G. biloba* and *S. giganteum* encode tRNA-IPT that participates in the cis-zeatin biosynthetic pathway (Supplementary Fig. 30). *Cycas panzhihuaensis* contains two-fold and three-fold higher numbers of 1-aminocyclopropane-1-carboxylate synthase and 1-aminocyclopropane-1-carboxylate oxidase, respectively, than *G. biloba*. Notably, broad-scale comparative genomics indicated 16 copies of ACO in the *C. panzhihuaensis* genome, suggesting a huge expansion compared to seedless plants and angiosperms. Based on our comparative genomics, the genes related to the ethylene biosynthetic pathway in gymnosperms exhibited a significant expansion compared to seedless plants. However, the genes related to the ethylene signaling pathway retained similar copy numbers among gymnosperm and angiosperms. We found 27 copies of *Ent-kaurene oxidase* in the *G. biloba* genome versus three copies in the *C. panzhihuaensis* genome. However, *G. biloba* is appears not to encode *Ent-kaurene synthase*, which is an important enzyme involved in gibberellin biosynthetic pathway. As with the gibberellin patheway, *CCD8* homologs from the strigolactone biosynthetic pathway was also evolutionarily lost in *G. biloba,* but exists in other gymnosperms, indicating strigolactone might not be bio-synthesized in *G. biloba*.

The *G. biloba* genome encodes a higher number of lipoxygenase and 12-oxophytodienoate reductase genes of the jasmonic acid biosynthetic pathway than that of *C. panzhihuaensis*; however, *allene oxide cyclase* exists as a single-copy gene in *G. biloba* versus four copies in the *C. panzhihuaensis* genome. *Isochorismate synthase* was not identified in the genome of *C. panzhihuaensis*, two fern genomes, or *P. patens*,

but these plants could encode the protein PHYLLO, which contains inactive isochorismate synthase as the C-terminal chorismate binding domain is absent. Phytohormone measurement showed *C. panzhihuaensis* is able to biosynthesize salicylic acid, suggesting an alternative salicylic acid biosynthetic pathway might exist in *C. panzhihuaensis*, or that the protein PHYLLO is produced by an active isochorismate synthase in *C. panzhihuaensis*.

9. Comparative transcriptome analysis of tissue-specific expressed genes

We performed a comparative transcriptome analysis to obtain tissue-specific expressed genes and constructed a flower plot, which revealed that 15,875 genes were commonly expressed in all 10 tissues sampled, while most tissues only had a limited number of tissue-specific expressed genes (Supplementary Fig. 31). The extreme case was found in stem pith, in which only 18 genes were specifically expressed, which suggested most tissues of *C. panzhihuaensis* showed a similar pattern of gene expression (Supplementary Fig. 30). Only three tissues had hundreds of tissue-specific expressed genes, such as the apical meristem of stem and mature leaf, which had 264 and 220 specifically expressed genes, respectively. The ovule exhibited the most tissue-specific expressed genes, suggesting its distinct expression pattern compared to other tissues. KEGG enrichment of tissue-specific expressed genes for each tissue was further performed to understand the differences among tissues (Supplementary Table 29). For example, 220 specifically expressed genes of the mature leaf were enriched in KEGG categories related to isoquinoline alkaloid biosynthesis, stilbenoid, diarylheptanoid and gingerol biosynthesis, pantothenate and CoA biosynthesis, and riboflavin metabolism, while KEGG functional enrichments of the ovule specifically expressed genes showed they are related to selenocompound metabolism, pentose and glucuronate interconversions, and ubiquitin-mediated proteolysis.

10. Transcriptome landscape of ovule pollination, fertilization and development of *C. panzhihuaensis*

10.1 Expression patterns of genes related to ovule development

To understand the dynamic changes of the genes and programs expressed during the development of ovule pollination and fertilization, we performed weighted correlation

network analysis (WGCNA) based on the 22,153 expressed genes and found more than 0.5 coefficient of variation across the four stages of ovule development (Supplementary Fig. 32; Figures 3a). In total, 11 co-expression modules were identified, and the number of genes per module ranged from 86 genes (in module M5) to 5,340 genes (in M11). Module-stage association analyses further revealed that six of these modules (i.e., M2, M4, M6, M8, M9, and M11) showed high correlations with one specific stage (PCC $\geq$ 0.85 for one stage and PCC $<$ 0.30 for other stages), whereas other modules were correlated with two or more adjacent stages (PCC $\geq$ 0.30; Fig. 3a). To understand the differences between the 11 modules in functional properties, we also performed GO enrichment analyses (Fig. 3a), and different modules displayed different GO categories.

The GO categories enriched in M2, M6, and M8 are plant nutrition metabolic process genes, for example, organophosphate metabolic, carbohydrate derivative metabolic, and organonitrogen compound process genes. The M4 genes that were highly expressed at the early developmental stage of the pollinated ovule were enriched for a family related to callose synthase (i.e., 1,3-beta-D-glucan synthase activity). The family is proposed to synthesize callose, which is a major component of pollen tubes and also related to salicylic acid-dependent disease resistance[46]. M9-enriched membrane biosynthesis genes may related to developemnt of the integument.

10.2 Expression patterns of seed-specific genes

In comparative transcriptomics analysis of different tissues, we collected 1,290 genes specifically expressed during the four stages of ovule development. A heat map (Fig. 3a) further displays the expression patterns of these seed-specific genes during the development process. There are some genes related to zeatin biosynthesis and selenocompound metabolism expressed in the unpollinated ovule. Genes related to pentose and glucuronate interconversions and cysteine and methionine metabolism pathways were expressed in the early stage of the pollinated ovule. Genes associated with the mRNA surveillance pathway and the RNA transport pathway were expressed in the late stage of the pollinated ovule. Genes related to carotenoid biosynthesis and the proteasome pathway were expressed in the fertilized ovule. Meanwhile, we also indentified the expressed transcription factors in seed development processes (Supplementary Table 30).

10.3 Phytohormone analyses of different developmental processes

Various phytohormones at four stages of ovule development were quantified. Salicylic acid showed higher expression in the unpollinated ovule; however, it was barely detected in the early stages of the pollinated ovule, the late stage of the pollinated ovule, and the fertilized ovule (Supplementary Table 31). 1-Aminocyclopropane-1-carboxylate (ACC), which reflects ethylene content, was detected at higher levels in the unpollinated ovule and the early stage of the pollinated ovule and gradually decreased in the late stages of the pollinated ovule, with very little ethylene detected in the fertilized ovule. A substantial amount of gibberellic acid was detected in the late-stage pollinated ovule, while the level of gibberellic acid in the unpollinated ovule, early-stage pollinated ovule, and fertilized ovule was much less. Abscisic acid was mainly detected in the early-stage pollinated ovule and fertilized ovule, higher than in the unpollinated ovule and late-stage pollinated ovule. Jasmonic acid isoleucine and jasmonic acid were mainly produced in the unpollinated ovule, and decreased levels of the two metabolites were observed in the early-stage pollinated ovule. However, they were almost undetectable in either the late-stage pollinated ovule or fertilized ovule. A substantial amount of 3-indoleacetic acid was detected only in the early-stage pollinated ovule, which showed >5 times more 3-indoleacetic acid than the other three stages. Likewise, a large amount of trans-zeatin riboside was found to accumulate in unpollinated ovules compared to other three stages.

10.4 Metabolome analysis during developmental processes

We compared metabolomic changes among four stages of seed development, i.e., the unpollinated ovule, early-stage pollinated ovule (post-pollination at day 21), late-stage pollinated ovule (post-pollination at day 88), and fertilized ovule (post-pollination at day 119) (Supplementary Table 32). Comparing to the post-pollination stages, the unpollinated ovules accumulate some metabolites at significantly higher levels, e.g., tyrosol, epicatechin, and fisetinidol-4beta-ol. Epicatechin was reported related to pathogen response, and catechin acts as an infection-inhibiting factor in strawberry leaves[47]. Epicatechin and catechin may prevent coffee berry disease in *Coffea arbica* by inhibiting appressorial melanization of *Colletotrichum kahawae*[48].

10.5 The expression levels of phytohormone related genes in different seed developmental stages

Phytohormone analyses of four stages of *C. panzhihuaensis* seed development indicated that four types of phytohormone differed between the unpollinated ovule and the ovule at an early stage of pollination (ovule of post-pollination at day 21), i.e., salicylic acid, jasmonic acid, auxin, and abscisic acid. Both salicylic acid and jasmonic acid are expressed at higher levels in unpollinated ovules compared to post-pollination ovules. The two phytohormones are related to pathogen resistance, and genes encoding these two phytohormones were both detected at higher expression levels in the transcriptomes of the unpollinated ovule. The results may indicate the higher demand/ability of pathogen resistance in the unpollinated ovule. In the early stage of pollination, salicylic acid and jasmonic acid were reduced significantly, while both auxin and abscisic acid accumulated, reflecting the increase of developmental regulation on the seed and greater stress response at this stage; some gene responses to stimuli were detected with increased expression levels at this stage as well (Supplementary Table 33).

Compared to the early-stage pollinated ovule, the late-stage pollinated ovule accumulated only significant amounts of gibberellin, and all other phytohormone levels were reduced in the late-stage pollinated ovule. Gibberellin is essential for seed development; it causes seed germination by breaking the seed's dormancy and acting as a chemical messenger[49], and it was also reported to regulated ovule integument development[50]. Constitutive GA signaling by diminished DELLA function results in changes in the ovule/seed shape due to alterations in integuments/testa, which have consequences in both fertility and seed dormancy. To be noted, in the transcriptome of the late-stage pollinated ovule, we found enhanced expression of gene families related to cutin, suberine, and wax biosynthesis, which are loosely related to integument development. Some gene families related to nutrient reservoir activity are found with higher expression levels in the late-stage pollinated ovule, indicating nutrient accumulation is still activated at this stage. Meanwhile, some nutrition metabolizing genes, like Vitamin B6 metabolism and carotenoid biosynthesis, are enhanced in expression in the late-stage pollinated ovule. However, gene families related to pathogen interaction and hormone signal transduction are reduced in expression, consistent with our phytohormone measurements.

In the fertilized ovule, the most accumulated phytohormones are abscisic acid (ABA) and cytokinins. ABA reversibly arrests embryo development at the brink of radicle growth initiation, inhibiting the water uptake that accompanies embryo growth. Seeds that have been kept dormant by ABA for several days will, after removal of the hormone, rapidly take up water and continue the germination process. Cytokinins are known to play important roles in ovule patterning and development, and they also accelerate germination rates and increase the final germination percentage. We found in the fertilized ovule that genes related to response to auxin stimulus and cell wall organization or biogenesis have higher expression levels than at other stages, reflecting their high activity in seed coat development. Compared to the late-stage pollinated ovule, the fertilized ovule expresses the following gene families at two-fold higher levels: ribosome arachidonic acid metabolism, Beta-alanine metabolism, and the pentose phosphate pathway. Ribosome biogenesis is a prerequisite for cell growth and proliferation, and ribosome biogenesis is a determinant process for organism growth[51]. Within this stage of seed development, genes related to arachidonic acid metabolism increased in expression, indicating that the seed started to be activated in fatty acid metabolism. At this stage, genes related to nutrient reservoir activity are down-regulated.

10.6 Expansion of genes related to seed physiologies and development

We observed significant expansions of seed physiology-related genes (including seed viability, coat development, germination, immunity, nutrition, seed growth and development). Of these, the most notable gene family was the cupin (PF00190) proteins (Supplementary Table 34), which showed a huge expansion of cupin domain in *C. panzhihuaensis* compared to other plants. The functional annotation of the expanded cupin gene family of *C. panzhihuaensis* indicated that they could be classified into germin-like proteins and seed storage proteins. To further understand the relationship of the cupin gene family of *C. panzhihuaensis*, we collected cupin protein from representative bryophytes, lycophytes, ferns, gymnosperms and angiosperms and performed a phylogenetic analysis (Fig. 3 c). We found nine groups of germin-like protein (GLP1) among these plants. GLP1 is only present in angiosperms, suggesting it might have originated in the ancestor of angiosperms. GLP2 includes only the gymnosperms germin-like proteins; furthermore, GLP2 exhibited a remarkable

expansion in *C. panzhihuaensis* in tandem repeat form. GLP3 only has germin-like proteins from bryophyte genomes, indicating it might be similar to the ancestral GLP and specifically functions in early embryophytes. GLP4 contains genes from gymnosperms and angiosperms. Interestingly, although *G. biloba*, *S. giganteum*, *G. montanum*, and *P. abies* encode GLP4 homologs, GLP4 was evolutionarily lost in the *C. panzhihuaensis* genome. GLP5 contained all representative taxa, indicating it is widely distributed across plant lineages. *Cycas panzhihuaensis* did not show any expansion in this GLP5 group. GLP6 contained genes from ferns, gymnosperms, and angiosperms and one homolog in *P. patens*; however, we could not identify its homologs in lycophytes. A remarkable expansion of GLP6 was observed in *C. panzhihuaensis* compared to that of *G. biloba*. Although GLP7 was lost in lycophytes, and it appears to be lost in angiosperms (like *A. thaliana*), a moderate expansion of GLP7 via tandem repeat was observed in the *C. panzhihuaensis* genome, but not in *G. biloba*. However, only *G. biloba*, *C. panzhihuaensis*, and *S. giganteum* encode GLP7 homologs. GLP8 homologs were found in the genomes of bryophytes, lycophytes, ferns, and gymnosperms, but appear to have been lost in angiosperms, at least *A. thaliana*. Conifer (including *P. abies* and *S. giganteum*) encoded numerous GLP8 homologous proteins, but *C. panzhihuaensis* and *G. biloba* only contained one and two GLP8 genes, respectively. The phylogeny of germin-like proteins (another clade of cupin-domain-containing seed storage proteins) showed two main groups of seed storage proteins, the legumin-type globulin storage protein (12S seed storage protein) family (L-ssp) and the vicillin-like storage protein family (V-ssp). The L-ssp family dates back to the ancestor of lycophytes given that no legumin-type globulin storage protein was found in *P. patens*. *Cycas panzhihuaensis* only encodes three homologous proteins of 12S seed storage, which is fewer than in *G. biloba* (nine copies). *Cycas panzhihuaensis* encodes seven copies of vicillin-like storage proteins, but only one homolog of vicillin-like storage protein could be identified in *G. biloba*. Similarly, the *P. patens* genome does not appear to encode vicillin-like storage proteins. We identified a new group of vicillin-like storage proteins homologous to the vicilin-like antimicrobial peptides that expanded via tandem repeats in *C. panzhihuaensis*. To further understand the possible function of the expanded germin-like proteins and seed storage proteins in *C. panzhihuaensis*, gene expression analysis of all the proteins in different tissues was performed. Interestingly, GLP2.1 homologs of *C. panzhihuaensis* were only expressed

in coralloid roots, especially in the precoralloid roots. The vicilin-like antimicrobial peptide groups in *C. panzhihuaensis* were nearly restricted in expression to the late-stage pollinated ovule and fertilized ovule, and their expression level gradually decreased with the development of the embryo.

The LAFL genes, namely LEC1, ABI3, FUS3, and LEC2, encode master transcriptional regulators that induce and maintain these different phases of seed development and repress precocious seed germination and vegetative growth in *Arabidopsis*. *LEC1* (*LEAFY COTYLEDON1*) is a member of the NF-YB protein family and has a closely related homolog, *LEC1*-like (*L1L*, *NF-YB6*), in *Arabidopsis*. *ABI3* (*ABSCISIC ACID INSENSITIVE3*), *FUS3* (*FUSCA3*), and LEC2 (*LEAFY COTYLEDON2*) belong to the plant-specific family of B3 domain transcription factors and thus are also named "AFL-B3" regulators. We searched the LAFL genes across the selected plant genomes. Eleven *NFYB* genes were identified in both *C. panzhihuaensis* and *G. biloba*. A phylogenetic analysis of *NFYB* genes showed that the *C. panzhihuaensis* genome contains two *LEC1*/*LEC1*-like, as does *Arabidopsis*. However, the *G. biloba* genome encoded three *LEC1*/*LEC1*-like homologs. We also constructed a phylogenetic tree of B3-domain-containing proteins. *ABI3* types of B3-domain-containing proteins did not show expansion in the *G. biloba* genome, while we found that the *FUS3* and *LEC2* types of B3-domain-containing proteins remarkably expanded in the form of tandem repeats in the *C. panzhihuaensis* genome. Additionally, we identified the appearance of one new clade of B3-domain-containing protein before the divergence of the *FUS3* and *LEC2* cladess.

A novel single-chained antifungal protein (Ginkbilobin) with a molecular weight of 13 kDa was isolated from the seeds of *G. biloba*. It exhibits moderate antibacterial action against *Staphylococcus aureus, Pseudomonas aeruginosa,* and *Escherichia coli*. It also suppresses the activity of HIV-1 reverse transcriptase and the proliferation of murine splenocytes. However, the *G. biloba* genome contains only one copy of the gene encoding Ginkbilobin (Supplementary Fig. 33; Supplementary Table 35). Interestingly, *C. panzhihuaensis* encodes 16 homologs of the gene for Ginkbilobin, among the highest found in the selected plants. A phylogenetic tree suggested Ginkbilobin might have originated at the ancestor of lycophytes and later it was evolutionarily lost after the divergence of gymnosperms, as we could not identify the homologs in early embryophytes and angiosperms. Ginkbilobin genes fall into four main clades. Of these,

Ginkbilobin clade 3 only contains genes from conifers. Ginkbilobin genes of *C. panzhihuaensis* were distributed in clades A, B, and D, and Ginkbilobin clade A displayed noteworthy expansion in *C. panzhihuaensis*.

We identified nine homologs of genes encoding GLE1 protein, which encode nucleoprin, in the *C. panzhihuaensis* genome. Although GLE1 protein is reported to be involved in seed viability in *Arabidopsis*, many plants only contain one or two copies of *GLE1* homologs (Supplementary Fig. 34; Supplementary Table 35). The tandem repeats of GLE1 protein genes in the *C. panzhihuaensis* genome might also be associated with the regulation of seed physiologies. Interestingly, when we carefully searched all the nuclear pore complex protein, *C. panzhihuaensis* showed an almost two-fold higher number of nuclear pore complex protein genes than did *G. biloba*, seedless plants, and angiosperms (Supplementary Table 36). Interestingly, we identified that embryo-specific protein ATSs were remarkably expanded in *Cycas* and *Ginkgo* compared to seedless plants. Embryo-specific protein ATSs were identified in developing *Arabidopsis thaliana* seeds, and they might play an important role during embryo development[52]. Thus, the expanded embryo-specific protein ATSs may have contributed to the evolution of seed formation during the transformation from seedless to seed plants (Supplementary Table 35).

Late embryogenesis abundant proteins (LEA proteins) play important roles in desiccation tolerance of seeds, and they are also reported to be related to the protection of plants against other stresses like cold and high salinity. They are also found functioning in other plant tissues and in some invertebrates, fungi, protists, and prokaryotes. All identified LEA genes were grouped into eight groups, ranging from LEA 1 to LEA 6, dehydrin, and seed maturation protein (SMP). We screened LEA protein genes among the genomes of selected streptophytes, and found they expanded in most gymnosperms except in *Cycas* and *Ginkgo*. Compared to seedless plants, each subfamily of LEA expanded in seed plants, especially LEA3, dehydrin, and seed maturation protein (SMP) (Supplementary Fig. 35).

11. Cell wall Carbohydrate-Active Enzymes (CAZymes) of *C. panzhihuaensis*

The CAZyme families were identified using dbCAN2[53], and unless indicated, the default thresholds (E-value < 1e-15 and coverage > 35%) were used to delineate each gene family. Gymnosperm genomes contained expanded CAZyme families compared to seedless plants. *Cycas panzhihuaensis* encoded 414 glycosyl hydrolase (GH), 432

glycosyl transferase (GT), 64 carbohydrate esterase (CE), 21 andpolysaccharidelyase (PL), 201 carbohydrate binding modules (CBMs), and 224 auxiliary activities (AAs) genes. The total number of CAZymes of *C. panzhihuaensis* (1,356) is higher than in *G. biloba* (1,169) and *G. montanum* (1,058); however, it is much lower than that of conifers (Supplementary Table 37).

11.1 Evolution of genes related to cell walls in *C. panzhihuaensis*

Gymnosperms represent a major group of seed-bearing plants, and most gymnosperms are tall, woody plants. Seeds carry nutrition and develop a seed coat to protect the embryo from desiccation. Seeds are believed to be the most important reason for the successful spread of seed plants into diverse and extreme environmental conditions. Cell wall compositions of gymnosperm seeds are not available, but the analyses of some angiosperms show the presence of higher amounts of mannose-containing hemicelluloses in the thick non-lignified walls of seeds[54], while some other angiosperm seeds contain large amounts of xyloglucan (XGs) and starch as storage polymers[55]. In cycads, the leaves and reproductive organs are borne at the top of an unbranched stem that keeps these vital parts away from soil-borne pathogens and herbivores.

Cell walls of gymnosperms contain high quantities of cellulose, fucoside XG, glucomannan, homogalacturonans (HGs), RGI, and RGII[56,57]. The XGs, however, have a higher number of fucose side chains, and RGII has a lower number of methylated side chains compared with seedless plants[54,58]. Such changes may play a role in the increase in tensile strength of the cell walls to support the tall upright plant body and its more advanced water transport systems than those of seedless plants (noting that angiosperms have more efficient water transport than gymnosperms). The cell wall components of gymnosperms, especially the lignins, can withstand high mechanical pressure exerted by the gravitational pull and the load of the tall plant body, and act as so-called anti-gravitation materials[59], providing mechanical strength to the tall trees. In addition, gymnosperms contain a high amount of lignins in the secondary cell walls of their woody stems, which render the cell walls highly recalcitrant. The phenolic compounds are organized into complex polymers through chemical radical reactions, and therefore the polymers do not display a predictable organization for which appropriate enzyme activity could have evolved. Hence, lignin provides not only mechanical strength but also an advanced level of protection from cell wall-degrading enzymes secreted by

pathogens[60]. The emergence of woody cell walls has been extraordinarily successful among plant survival strategies and has resulted in plants that have a life span of hundreds of years while resisting microbial attack and the threat of herbivorous animals.

We investigated the cell wall biosynthesis-related genes across representative plant genomes (Supplementary Table 38). (1) We performed a phylogenetic analysis of cellulose synthase and cellulose synthase-like genes. Our results show that the homologous proteins of *cellulose synthase-like H* (*CSLH*) originated in gymnosperms, *C. panzhihuaensis* encodes one copy of homologous proteins of *CSLH*, while three copies of homologous proteins of *CSLH* could be identified in *G. biloba*. (2) Additionally, we could not find angiosperm-like *CSLB* homologs in any gymnosperm genomes, suggesting the *CSLB* apparently originated in the ancestor of angiosperms. (3) Another noteworthy point is the apparent expansion of the *GT77* family of *Cycas* compared to other representative plant genomes. These genes encode UDP-D-xylose:L-fucose alpha-1,3-D-xylosyltransferase, which catalyzes the transfer of D-xylose from UDP-alpha-D-xylose into L-fucose. A further phylogenetic analysis clearly identify these expanded UDP-D-xylose:L-fucose alpha-1,3-D-xylosyltransferase genes as homologous to MGP4s/RGXTs of *Arabidopsis* (Supplementary Fig. 36). In plants, MGP4s/RGXTs are probably involved in the biosynthesis of rhamnogalacturonan II (RG-II) through xylosylation of the internal fucose moiety of the A-chain of RG-II, a structurally complex pectic polysaccharide of the primary cell wall[61]. RG-II is essential for the cell wall integrity of rapidly growing tissues such as roots and pollen tube growth and elongation[62].

11.2 Expansion of genes related to cell wall loosening, expansion and extensibilityin *C. panzhihuaensis*

Plant cells are enclosed within sturdy cell walls that make them tolerant of turgor pressure. However, in growing plant cells, the cell wall is loosened to increase its extensibility, and turgor pressure stretches the loosened cell wall until the turgor pressure and tensile strength of the cell wall counterbalance each other. The control of wall extensibility determines cell size in plants and plays a critical role in the development of plant organs[58].

The plant cell wall is composed of polysaccharides, proteins, and phenolic compounds[63]. Cellulose microfibrils are paracrystalline fibers which are primarily

responsible for the tensile strength of the cell wall. The cellulose fibers are embedded in a matrix consisting of hemicellulose and pectin[64]. Phenolic compounds are abundant in the secondary cell wall that is deposited inside the primary cell wall after cell expansion is completed. Structural proteins, as well as proteins which play roles in defense, environmental sensing, and intercellular signaling, are found in the cell wall. The plant cell wall is a dynamic compartment in which the composition and organization of its constituents are modified according to developmental programs or in response to environmental cues. Cell expansion in plants accompanies cell wall modification, and proteins involved in cell wall loosening have been shown to contribute to cell expansion[65].

We found that many gene families related to cell wall loosening, cell wall expansion, extendability were apparently expanded in all gymnosperm genomes on different levels compared to the angiosperms and seedless plants. Extensins are a family of flexuous, rodlike, hydroxyproline-rich glycoproteins (HRGPs) of the plant cell wall[66]. They are highly abundant proteins. They form crosslinked networks in the young cell wall. Typically, they have two major diagnostic repetitive peptide motifs, one hydrophilic and the other hydrophobic, with potential for crosslinking. Extensins are thought to act as self-assembling amphiphiles essential for cell-wall assembly and growth by cell extension and expansion[67]. The name "extensin" encapsulates the hypothesis that they are involved in cell extension[68]. The *C. panzhihuaensis* genome contains 56 extension-like proteins, which is seven times higher than that of *G. biloba* (Supplementary Table 39).

Arabinogalactan protein (APG) is another family member of HRGPs, and we found that a subfamily of APGs, namely fasciclin-like arabinogalactan proteins (FLAs), were also remarkably expanded in *C. panzhihuaensis,* suggesting their possible role in normal cell expansion and other roles such as participating in plant growth, development, defense against abiotic stresses, and, notably, cell wall biosynthesis. The number of FLAs of *C. panzhihuaensis* was more than two-fold higher than that of seedless plants. However, the expansion of FLAs was not observed in all gymnosperms. For instance, *G. biloba*, *S. giganteum*, and *Pseudotsuga menzesii* had 14, 12, and 13 copies of FLA proteins, respectively, which is similar to that of seedless plants.

Cycas and *Ginkgo* contain slightly more expansin genes than bryophytes, but nearly two-fold more than ferns. A huge burst of expansin genes was identified in

conifer genomes. Expansins are a class of proteins that are believed to break the load-bearing hydrogen bonds between xyloglucans and cellulose, thereby increasing cell wall extensibility[69]. Expansion might also cause loosening and extension of plant cell walls by disrupting non-covalent bonding between cellulose microfibrils and matrix glucans[70]. In addition, expansins (such as Expansion-B homologs) could aid fertilization by loosening the cell wall of the stigma and style, thereby facilitating penetration of the pollen tube as reported in *Nicotiana tabacum*[71].

The genomes of *C. panzhihuaensis* and conifers encode a higher number of homologs of COBRA or COBRA-like proteins than seedless plants (except for *P. patens*). Previous studies indicated COBRA/ COBRA-like proteins might be involved in determining the orientation of cell expansion[72], probably by playing an important role in cellulose deposition, and they may act by recruiting cellulose synthesizing complexes to discrete positions on the cell surface. Additionally, in *Arabidopsis* COBRA or COBRA-like proteins might participate in the deposition of the apical pectin cap and cellulose microfibrils in pollen tubes; they are essential for pollen tube growth in the female transmitting tract of the carpel and toward the micropyles, via the perception of ovule guidance cues[73]. They also might be involved in regulation of pollen development, hydration, or germination. Apart from *G. montanum*, *P. taeda*, and *P. lambertiana*, other gymnosperm genomes contain many copies of polygalacturonase 1 beta-like proteins (PLGs) compared to angiosperm and seedless plants. Plant PLGs might be involved in cell size determination and serve as a chaperone for expansins through the secretory pathway. Park et al.[74] provided evidence that PGL3 of *Arabidopsis* was an apoplastic BURP domain protein playing a role in cell expansion.

A few copies of Alpha-galactosidase homologs could be found in both seedless plant and angiosperm genomes (except for *S. moellendorffii*, which contains 12 Alpha-galactosidase); however, apparent expansion of Alpha-galactosidase could be observed in *C. panzhihuaensis*, *G. biloba*, and conifers (but not in *G. montanum*); 101 Alpha-galactosidases were found in the genome of *P. menzesii*. Alpha-galactosidase proteins were reported to be involved in leaf and possibly other organ development by functioning in cell wall loosening and cell wall expansion[75].

Genes encoding the xyloglucan endotransglucosylase/hydrolase proteins (XTHs) from the GH16 family also displayed expansion in all of the gymnosperm genomes compared to angiosperms and seedless plants. XTHs belong to cell wall-modifying

proteins, and they also have the capacity to loosen cell walls[76]. Most XTHs cut and rejoin xyloglucan by xyloglucan endotransglucosylase action, whereas some XTHs hydrolyse xyloglucan by xyloglucan hydrolase action[77]. Both activities may affect cell expansion. Additionally, previous study indicated XTH expression and XET activity were correlated with plant growth and cell wall mechanics. Some XTHs could alter tissue tensile strength, or flexibility, enabling adaptation to mechanically stressful environments[78].

We could not identify the ROOT HAIR DEFECTIVE 3 (RHD3) protein in fern genomes, but *C. panzhihuaensis* encoded seven homologs of RHD3. RHD3 is a probable GTP-binding protein that might be involved in cell wall expansion. It is required for appropriate root and root hair cell enlargement, and it might inhibit vacuole enlargement during root hair cell expansion[79].

11.3 Expansion of many other GH families in the *C. panzhihuaensis* genome

Numerous GH families apparently underwent expansion in *C. panzhihuaensis* and other gymnosperms compared to the seedless plants examined. Gymnosperm genomes exhibited an explosion in the number of endochitinase and chitinase genes. These genes expanded in the form of tandem repeats in the *C. panzhihuaensis* genome.

Endochitinase/chitinase is involved in the chitin degradation pathway[80]. The expanded number of genes of endochitinase/chitinase in gymnosperms might participate in defense against chitin-containing fungal pathogens. Another expanded GH is endo-1,4-beta glucanase and endo-1,3(4)-beta-glucanase from GH9 and GH5 families. We found a large number of endo-1,4-beta glucanase and endo-1,3(4)-beta-glucanase genes in gymnosperm genomes compared to seedless plants (Supplementary Table 40). GH5 and GH9 are known to degrade cellulose in embryophytes[81], and they play an important role in plant cell wall development, formation and reassembly[82]. We further analyzed all the enzymes of the GH5 family across all selected plant genomes because the GH5 family was remarkably expanded in gymnosperms compared to seedless plants. We found that the number of genes encoding glycoforms of a newly identified pollen allergen, Cha o 3, which belong to GH5, was greatly expanded in *C. panzhihuaensis*. However, the exact function of the Cha o 3 pollen allergen is not yet clear. Additionally, genes encoding the major pollen allergen Cha o 2 from GH28, which functions as a polygalacturonase, appeared to expand in all the gymnosperm

genomes except *G. biloba*. Polygalacturonases belong to the GH28 family and are key HG hydrolyzing enzymes that have been implicated with a wide range of plant developmental processes such as cell elongation, organ abscission, fruit ripening, microspore release, pollen tube growth, and pollen maturation[83].

The genomes of gymnosperms contain several copies of genes that encode glucan endo-1,3-alpha-glucosidase agn1 homologs, belonging to the GH71 subfamily. We found seven and three copies of glucan endo-1,3-alpha-glucosidase agn1 in the genomes of *G. biloba* and *C. panzhihuaensis*, respectively, while it could not be found in most of the angiosperm and seedless plant genomes. Glucan endo-1,3-alpha-glucosidase agn1 has a role in cell separation, where it is required for the degradation of the cell wall material surrounding the septum, which must be hydrolyzed before full separation of the daughter cells[84]. Notably, the presence of homologs of glucan endo-1,3-alpha-glucosidase agn1 in genomes of a few seedless plants (*S. moellendorffii* and *M. polymorpha*) and some gymnosperms suggests a complicated evolutionary history, given that we could not find the homologs of glucan endo-1,3-alpha-glucosidase agn1 in representative genomes of streptophyte algae, chlorophyte algae, Prasinodermophyta, red algae, or glaucophyte algae. This gene was highly similar to the glucan endo-1,3-alpha-glucosidase agn1 of fungi and bacteria; thus, the homologs of glucan endo-1,3-alpha-glucosidase agn1 might have been acquired from fungi or bacteria through horizontal gene transfer after plant terrestrialization.

Compared to the number of glucan endo-1,3-beta-glucosidase (GH17) in genomes of seedless plants, *C. panzhihuaensis* and other gymnosperms contain more copies of glucan endo-1,3-beta-glucosidase, especially *P. taeda* and *P. lambertiana*, which encode 229 and 131 homologs of glucan endo-1,3-beta-glucosidase, respectively. Similar in function to endochitinase/chitinase, endo-1,3-beta-glucosidases are also thought to be an important plant defense-related product against fungal pathogens[85]. They are capable of releasing soluble and highly active elicitor molecules from fungus cell walls.

We also found that genes encoding mannan endo-1,4-beta-mannosidase (endo-β-mannanases) from GH26 displayed remarkable expansion in gymnosperm genomes compared to seedless plants. Mannans are hemicellulosic polysaccharides in the plant primary cell wall with two major physiological roles: as storage polysaccharides that provide energy for the growing seedling and as structural components of the

hemicellulose–cellulose network with a similar function to xyloglucans[86]. Endo-β-mannanases are hydrolytic enzymes that cleave the mannan backbone. They are active during seed germination and during processes of growth or senescence. Recent studies indicated endo-β-mannanases are required for both loosening of the micropylar endosperm and rupture of the seed coat in germinating of angiosperm seeds[87]. Endo-β-mannanases might participate in the hydrolysis of the mannans in the cell wall of germinating seeds.

Additionally, with the exception of *G. biloba*, we also found a big expansion of Beta-glucosidase genes in all gymnosperm genomes compared to seedless plants, even more than in angiosperms. Beta-glucosidases that belong to the GH1 family catalyze the hydrolysis of the b-glucosidic bond between two carbohydrate moieties or a carbohydrate and an aglucone moiety. In plants, beta-glucosidases play important roles in diverse aspects of plant physiology, e.g. (1) formation of intermediates in cell wall lignification[88], (2) cell wall degradation in endosperm during germination[89] of angiosperm seeds, (3) activation of phytohormones[90], and (4) activation of chemical defense compounds[91].

11.4 Expansion of genes related to lignin

The majority of gymnosperms are tall, woody plants. Lignins play a crucial role in the formation of cell walls, particularly wood and bark[92]. Coniferyl alcohol, sinapyl alcohol, and p-coumaryl alcohol are the three natural phenylpropane alcohols that make up lignin[93].

We looked into the genes involved in the lignin biosynthesis process because of the importance of woody tissues in gymnosperms. Interestingly, almost all gymnosperms had a significant increase in genes involved in the lignin biosynthesis process compared to the other land plants (including the angiosperm *Amborella trichopoda*). Surprisingly, *G. biloba* did not display an increase in the number of genes involved in the lignin biosynthesis pathway (Supplementary Table 41). Notably, we were unable to find homologs of ferulate 5-hydroxylase in all gymnosperms, implying that gymnosperms and angiosperms could have different lignin metabolism.

11.5 Expansion of genes related to pollen tube physiology

The genome of *C. panzhihuaensis* possesses many expanded gene families that are related to cell wall loosening, expansion, elongation, and extensibility compared to seedless plants. Notably, these features of the cell wall play vital roles in pollen tube growth and development. In addition to the aforementioned gene families of expansins, RGXTs, COBRA/COBRA-like proteins ,polygalacturonases, and major pollen allergen Cha o 2, which were reported to be directly involved in pollen tube physiology, we also found several other expanded gene families that might be related to pollen tube physiology. (1) We found that Ole e 1-homologous proteins were greatly expanded in gymnosperms compared to the seedless plants (except for *S. moellendorffii*). Notably, the number of Ole e 1-homologous proteins in *C. panzhihuaensis* was over two-fold higher than in *G. biloba*. Pollen allergens (Ole e 1-homologous proteins) mainly accumulate in pollen tube cell walls and may have a role in pollen germination and pollen tube growth. This gene family might be also involved in recognition between pollen-stigma and pollen tube-style cells in angiosperms, and some Ole e 1-homologous proteins could be the components of functional scf complexes and essential for pollen tube elongation[94]. (2) Protein POLLEN DEFECTIVE IN GUIDANCE (PCD) mainly exists in single-copy form in many plant genomes[95]. However, we found five copies in the genome of *C. panzhihuaensis*. PCD is a probable component of the calreticulin 3 (CRT3) complex, acting probably as a co-chaperone involved in protein retention in the endoplasmic reticulum lumen. It is also required for micropylar pollen tube guidance and plays an essential role in cell plate orientation or positioning in early embryo patterning[95]. (3) We found that the egg cell-secreted protein may have originated in the ancestor of seed plants. This protein might be involved in the regulation of gamete interactions during double fertilization in angiosperms and prevent multiple-pollen tube attraction, and it also mediates the redistribution of the gamete fusogen HAP2/GCS1 to the cell surface after secretion upon sperm arrival. (4) The *C. panzhihuaensis* genome contains seven homologs of Profilin, which is nearly two-fold higher than the seedless plants; however, this gene does not show any expansion in *G. biloba*. Profilins regulate actin polymerization by sequestering or releasing actin monomers during pollen growth. (5) The polcalcin gene family was apparently expanded in all gymnosperm genomes compared to the angiosperms and seedless plants. Polcalcins are reported to be involved in calcium signaling to help guide pollen tube growth (Supplementary Table 42).

Another expanded gene, HOTHEAD, is an *Arabidopsis thaliana* gene that encodes a flavin adenine dinucleotide-containing oxidoreductase. This gene has a role in the origination of the carpel during the formation of flowers through the fusion of epidermal cells. It should be noted that HOTHEAD-Like HTH1 was also reported to be involved in cutin biosynthesis, and it is required for pollen fertility in rice[96]. The cuticle layer that covers the surface of organs plays a pivotal role in plant adaptation to adverse environmental conditions[97]. In addition to providing physical support for pollen development, the cuticle layer on the outermost epidermis of the anther is important for protecting the inner tissues from various stresses and pathogen attacks[98]. Therefore, the expansion of HOTHEAD in gymnosperms might contribute to pollen development and physiology.

12. Analysis of flagellar genes and their phylogenetic distribution

12.1 An overview of presence/absence of orthologogous genes related to the flagellar structure among representative plants

Phylogenetic inference indicated there were three flagellar loss events in the evolution of Streptophyta (i.e., Zygnematales branch, Gnetales plus conifiers, and angiosperms). We performed a comparative analysis of key proteins related to flagellar structure, and our results revealed that 1) *C. panzhihuaensis* and seedless plants with flagellar features retain most key proteins related to flagellar structure; 2) A slightly decreased number of key flagellar proteins was observed in the *G. biloba* genome; the RSP proteins (RSP2, 3, 9, 11) were distributed in all bryophytes, lycophytes, ferns, and *C. panzhihuaensis*; however, the genes encoding these proteins were lost in *G. biloba* genome. In addition, genes for the intraflagellar transporter and dynein protein also displayed varying degrees of loss. Compared to *C. panzhihuaensis*, *Gnetum*, conifers, and angiosperms exhibited the loss of genes for key proteins related to flagellar structure. Additionally, the loss of these genes might also affect sperm motility, ultimately leading to flagellar loss.

13. Sex chromosome assembly, annotation, and analysis

13.1 Identification differentiated sex chromosome region in genome

Our genome-wide association study (GWAS) of sex as a binary phenotype identified the most significant association signals on chromosome 8, expanding from 0 to 124 Mb on the reference genome. This 124-Mb region also overlapped with the most differentiated regions between males and females identified by nucleotide diversity (π) and population differentiation index (F_{ST}) (Fig. 4a; Supplementary Fig. 37). We observed the greatest differences in nucleotide diversity and heterozygosity ratio spanning 18-50 Mb, accompanied by the highest F_{ST} value between the male and female populations (Fig. 4a,b). These results pinpointed the most sex-differentiated region on chromosome 8 in the *C. panzhihuaensis* genome; cycads represent the most ancient lineage with heteromorphic sex chromosomes in seed plants.

13.2 Male-specific region of Y chromosome (MSY)

To obtain the male-specific region of the Y chromosome (MSY), we sequenced a male individual with Nanopore long-read sequencing and Hi-C sequencing methods. Phased reads were extracted from the 877.3 Gb of Nanopore reads and used for contig assembly, followed by scaffolding with phased Hi-C reads. The SSK_finder pipeline (https://github.com/BGI-Qingdao/SSK_finder) was used to extract male-specific 21 k-mers, which were used to select male-specific Nanopore reads with at least a coverage of 0.2%. Contigs were assembled from these male-specific long reads by NextDenovo (https://github.com/Nextomics/Nextdenovo) and polished with male-specific short reads by NextPolish (https://github.com/Nextomics/NextPolish). Then, the contigs were assembled into scaffolds using Juicer and 3D-DNA[99] with male-specific Hi-C reads extracted by the same pipeline as for short reads. The male-specific scaffolds were aligned against the *C. panzhihuaensis* genome using MUMmer[100]. A total of 45.5-Mb sequence was assembled into 43 scaffolds. The MSY sequences were annotated following the same pipeline as for the reference genome. RNA-seq reads were aligned against the *C. panzhihuaensis* reference and MSY scaffolds using STAR[101] (vesion 2.5.3); read alignment was visualized using Integrative Genomics Viewer (IGV)[102]. Comparative genomic analysis of MSY aligned most MSY scaffolds to the putative sex determination region on chromosome 8 identified by association analysis (Supplementary Fig. 38). The longest two assembled scaffolds, which account for more than half of the MSY assembly, were aligned to the most differentiated 18-50-Mb region on chromosome 8. Identification of a sex co-segregating region in chromosome

8 and the assembly of the MSY region agreed with previous karyotype analyses showing that *C. pectinate* and *C. revoluta* contained a pair of heteromorphic sex chromosomes in their genomes[103,104].

A total of 624 putative protein-coding genes were annotated within the MSY, with 11 highly expressed in male reproductive organs (TPM > 1). The most highly expressed gene, CYCAS_034085, also the most differentially regulated gene between two sexes, encodes a GGM13-like MADS-box transcription factor that belongs to an ancient lineage sister to the angiosperm *AP3/PI* clade (Supplementary Fig. 39). Its homologous gene, CYCAS_010388, was identified on one of the *Cycas* autosomes. In contrast to the male-exclusive expression of CYCAS034085, CYCAS_010388 from chromosome 2 showed a significantly higher expression in ovules than microsporophylls. MADS-Y/CYCAS_034085 and CYCAS_010388 were used to search against the NCBI protein database for their homologs using blast-p with an E-value cutoff of 1e-50. MADS protein-coding genes were processed with TranslatorX[105] using MAFFT[106] to align the amino acid sequences and generated the corresponding nucleotide alignments. Poorly aligned positions were trimmed by trimAl[107] with the gappyout option. Maximum likelihood (ML) analyses were performed using IQ-TREE2[108] with 1000 ultrafast bootstrap replicates. The best-fitting model was GTR+F+I+G4 determined based on AICc. Phylogenetic analyses indicate that the investigated cycad species as well as *G. biloba* contain these two B-sister members before their divergence.

To examine the presence or absence of the sex-related genes in males and females of other cycad species, we designed gene-specific primers for the loci *MADS-Y/CYCAS_034085* (forward primer, 5'- AAGTGACATTCTCCAAGCGTAG-3'; reverse primer, 5'- TTCGAACACGAGTTGCTGAAAT-3') and *CYCAS_010388* (forward primer, 5'- TCTGCGAGATGACAAGAATCA-3'; reverse primer, 5'- CAGCCACTTCTAGCTGCTGT-3'). Molecular genotyping was performed with T3 Super PCR Mix (TINGKE Biotechnology, Beijing, China) using leaf DNA as a template. The PCR products were examined by electrophoresis in 1% agarose gel. We found that the PCR product of CYCAS_034085 was amplified from all tested male but not female cycad samples, while the CYCAS_010388-specific PCR product was amplified in both male and female samples. We concluded that the GGM13-like transcription factor, CYCAS_034085, represents a potential candidate for a sex

determination gene in *Cycas*, and that a similar sex determination system emerged before the divergence of the ancient cycads.

13.3 Variant calling of Y chromosome

Population genetic analysis. Principal component analysis (PCA) was performed on the filtered SNP set using GCTA (v1.91.4beta3)[109]. A neighbor-joining (NJ) tree was constructed with 100 bootstraps using PHYLIP (v3.696)[110], and the tree layout was generated using FigTree (v1.4.3; http://tree.bio.ed.ac.uk/software/figtree/). Population structure was analyzed with the cluster number K ranging from 1 to 5 by ADMIXTURE (v1.3.0)[111] using a default 5-fold cross-validation (cv=5). Each K was run with 20 replicates and the output aligned by CLUMPP (v1.1.2)[112]. Genetic differentiation (F_{ST}) and nucleotide diversity (π) were calculated within a non-overlapping 100-kb window using VCFtools (v0.1.13)[113].

14. Pathogen and predator resistance

14.1 Immune receptor genes in the *C. panzhihuaensis* genome

Plants possess intracellular immune receptors and extracellular immune receptors[114]. Cycads are reported to display strong disease resistance; therefore, we investigated the immune-related genes across selected plants. The plant intracellular immune receptors are usually divided into several subfamilies according to their N-terminal domain identities: *CC+NBS+LRR*(*CNL*), *TIR+NBS+LRR*(*TNL*), and *RPW8+NBS+LRR*(*RNL*) (Supplementary Table 43). Truncated genes having lost their N-terminal domain or LRR domain could still be attributed to subfamilies by sequence specificity or phylogenetic analysis. The *C. panzhihuaensis* genome contains 146 intracellular immune receptor genes, which is highly expanded compared to seedless plants. However, the number of intracellular immune receptors was almost half (146 of 257) of that detected in *G. biloba*; *C. panzhihuaensis* contained the least number of intracellular immune receptors compared to other gymnosperms. Interestingly, many conifers contained a large number of intracellular immune receptors; for example, 1,391 intracellular immune receptor genes could be identified in *P. taeda*. In addition, we also searched the extracellular immune receptor which could be divided into LRR type and Lysm type extracellular immune receptors. Likewise, extracellular immune receptor genes displayed expansion in all gymnosperms compared to seedless plants.

14.2 Expansion of pathogenesis-related genes in the *C. panzhihuaensis* genome

Pathogenesis-related proteins, often called PR proteins, are a structurally diverse group of plant proteins that are toxic to invading fungal pathogens[115]. They are widely distributed in plants in trace amounts, but are produced in much greater concentration following pathogen attack or stress[116]. PR proteins exist in plant cells intracellularly and also in the intercellular spaces, particularly in the cell walls of different tissues[115]. Interestingly, gymnosperms not only displayed an expanded number of immune receptor related genes, but many PR gene families also appeared to be expanded compared to seedless plants: PR-4 family (barwin domain containing proteins) (PF00967), PR-1 family (cysteine-rich secretory protein family domain containing proteins) (PF00188), PR-5 family (thaumatin family domain containing protein) (PF00314), and a new PR family (PAR1 proteins domain containing protein) (PF06521) (Supplementary Table 44).

14.3 Expansion of programmed cell death-related genes in the *C. panzhihuaensis* genome

Plants have evolved surveillance systems and cellular responses to sustain their growth while protecting themselves against various environmental stresses, often through deploying programmed cell death (PCD) to balance survival signaling with proper development patterns and abiotic stresses or microbial infections[117]. Interestingly, we found many PCD-related gene families expanded in *C. panzhihuaensis* compared to other plants (Supplementary Table 45). Notably, *Cycas* and *Ginkgo* encode up to 18 plant polyphenol oxidases (PPOs), which is similar to that found in seedless plants (except for *M. polymorpha*); however, the PPOs appear to be lost in *G. montanum*, some conifers, and angiosperms. PPOs were recently found to be involved in regulation of cell death[118] and might also participate in biochemical seed defense mechanisms[119]. Genes encoding stigma-specific STIG1-like protein and arginine decarboxylase were specifically expanded in *C. panzhihuaensis* genome. Stigma-specific STIG1-like protein was reported to be involved in the regulation of cell death induced by extracellular ROS[120]. Arginine decarboxylases of *A. thaliana* play an important role in defense against the pathogenic bacterium *Pseudomonas viridiflava* [121].

14.4 Expansion of stress-tolerance related genes in the *C. panzhihuaensis* genome

We observed significant expansions of stress-tolerance related genes in the *C. panzhihuaensis* genome compared to seedless plants (Supplementary Table 46). 1) Heat shock protein: not only Cycas, but all gymnosperm genomes contained plenty of heat shock proteins, such as Hsp20/alpha crystallin family domain containing protein, Hsp70 protein and Hsp90 protein. 2) We further searched the ROS-related gene families across the selected plant genomes. We found that genes for class III peroxidase, laccase, and multi-copper oxidase exhibited different levels of expansion in genomes of gymnosperms compared to seedless plants. Notably, we found that genes encoding microsomal glutathione S-transferase and peroxiredoxin were significantly and specifically expanded in the s*C. panzhihuaensis* genome. Additionally, genes for the DETOXIFICATION protein, which functions in the metal stress response[122], were remarkably expanded in all gymnosperms compared to *G. biloba* and seedless plants. Stress-induced ROS might cause DNA damage[123], and we found that *C. panzhihuaensis* contains a large number of histone H2A and histone H4 protein-coding genes compared to the seedless plants and angiosperms. Histone proteins play an important role in DNA repair in plants[124]. Conifers also showed higher numbers of histone H2A protein genes in their genomes; however, histone H4 proteins did not show notable expansion in many conifers. The number of either histone H2A or histone H4 of *G. montanum* was similar to seedless plants. Gene families related to the hypoxia response were also expanded in gymnosperm genomes compared to seedless plants (e.g., plant cysteine oxidase and Protein EXORDIUM)[125,126]. Additionally, we found *C. panzhihuaensis* encoded more copies of 'hypoxia induced protein conserved region containing proteins' than seedless plants, angiosperms, or other gymnosperms. Besides the aforementioned gene families, many other stress resistance-related genes that are expand in *C. panzhihuaensis* or other gymnosperms compared to seedless plants are summarized in Table S44, including C-terminal domain small phosphatase[127], annexin[128], universal stress protein[129], BURP domain-containing protein[130], jacalin-related lectins[131], desiccation-related protein PCC13-62[132], transcription termination factor MTERF [133], SOUL heme-binding protein[134,135], SINA E3 Ubiquitin Ligases, VQ motif-containing protein[136], and wound-induced protein[137]. Notably, genes for C-terminal domain small phosphatase displayed enormous and specific expansion in the genome of *C. panzhihuaensis*. A phylogenetic analysis showed C-terminal domain phosphatase-like family (CPL) could be mainly

classified into two groups in plants, i.e., CPL-A and B. Group A could be further separated into two subfamilies (A1 and A2) (Supplementary Fig. 40). The genome of *C. panzhihuaensis* encodes only one CPL group A1 and A2, respectively. Interestingly, the expanded CPL of *C. panzhihuaensis* mainly clustered in the B2 and B3 groups.

SINA E3 ubiquitin ligases: They are versatile moderators of plant growth and stress response. This gene family was reported to be involved in autophagy and responses to drought stress[138]. Additionally, SINA E3 ligases also participate in plant immunity and symbiosis. We found that the E3 ubiquitin-protein ligase SINAT gene family showed a burst in copy number in the *C. panzhihuaensis* genome compared to other representative plant genomes. Abscisic stress-ripening protein (ASR) is a plant-specific, small, and hydrophilic protein[45]. ASR1 was the first member of the ASR gene family characterized from the stress-treated leaves of *Solanum lycopersicum* L.[139]. Then, a large number of ASR homologs was detected from a wide range of other plant species, including gymnosperms, monocots, and eudicots. However, they are absent in the model plant *Arabidopsis* (Table S46). ASR proteins are found both in the nucleus and the cytosol and are known to mainly participate in growth and fruit ripening[139,140], as well as in regulation of floral development and flowering time[141]. Moreover, they are ubiquitously expressed in various organs in response to ABA and abiotic stress, such as osmosis, high salinity, and dehydration. In conclusion, the expansion of ASR genes in gymnosperms may play a number of important roles in plant growth, physiology, and stress response.

14.5 Expansion of potato type protease inhibitor in *C. panzhihuaensis*

Serine protease inhibitors (PIs) are a large and complex group of plant proteins. Members of the potato type I (Pin1) and II (Pin2) proteinase inhibitor families are among the first and most extensively characterized plant PIs[142]. Many insects and phytopathogenic microorganisms use intracellular and extracellular serine proteases, which play important roles in pathogenesis[143]. Plants, however, are able to fight these pathogens through the activation of an intricate defense system that leads to the accumulation of various PIs, including Pin1 and Pin2. About 26 potato type protease inhibitors were identified in the *C. panzhihuaensis* genome, a number that is notably higher than detected in other plants. In addition, we found that the numbers of basic secretory protease and subtilisin-like protease genes were expanded in gymnosperms,

especially in conifers. Both types of proteases were reported to have multiple functions, such as stress resistance and defense from pathogens[144,145]. Four copies of the gene encoding the NtpR protein, which belongs to the peptidase C26 family, were identified in *C. panzhihuaensis* (Supplementary Table 47). However, most seedless plants only contained a single homolog. The NtpR protein is able to enhance plant resistance to *Ralstonia solanacearum*[146].

In addition, we found a large number (28 copies) of genes encoding THO complex subunits in the *C. panzhihuaensis* genome, which is over two-fold higher than the number in the angiosperms and seedless plants and even higher than in *G. biloba* (12 copies). The THO complex might contribute to the integrity of the endogenous trans-acting small interfering RNA pathway[147]. Previous studies suggested it might participate in the trafficking of siRNA precursors to the ARGONAUTE catalytic center[148]. Thus, THO complex subunits are required for the generation of functional messenger ribonucleoproteins (mRNPs) and play important roles in plant innate immunity and disease resistance[149].

14.6 Expansion of immune signaling transduction related gene families

The significant expansions of gene families related to immune defense and stress-resistance in *C. panzhihuaensis* and other gymnosperm genomes motivated us to look for whether gene families related to stress and immune signaling regulation or transduction have expanded in gymnosperms. Interestingly, we found that three kinase families and one pathogen-induced CaM-binding protein family (PICBP) were greatly expanded in *C. panzhihuaensis* and other gymnosperm genomes.

Ca^{2+} and calmodulin (CaM), a key Ca^{2+} sensor in all eukaryotes, have been implicated in defense responses in plants. CaM-binding protein can bind calmodulin in a calcium-dependent manner in plants[150,151]. Previous studies have indicated that the expression of PICBP in *Arabidopsis* is induced in response to avirulent *Pseudomonas syringae* pv. tomato carrying avrRpm1. Furthermore, PICBP is constitutively expressed in the *Arabidopsis* accelerated cell death2-2 mutant, suggesting an important role in Ca^{2+}-mediated defense signaling and cell death. Three copies of genes encoding PICBP were identified in the *C. panzhihuaensis* genome, but none were found in seedless plants except for *Salvinia cucullata*. Compared to seedless plants, *C. panzhihuaensis* and most other gymnosperms (except *G. montanum*) contain more kinase-encoding

genes, for LEAF RUST 10 DISEASE-RESISTANCE LOCUS RECEPTOR-LIKE PROTEIN KINASE-like (LRK10L), G-type lectin S-receptor-like serine/threonine-protein kinase, and cysteine-rich receptor-like protein kinase (Supplementary Table 48). LRK10L is a receptor-like serine/threonine-protein kinase involved in abscisic acid (ABA) signaling, which also acts as a positive regulator of abiotic stress response[116]. G-type lectin S-receptor-like serine/threonine-protein RLKs are responsible for sensing and transducing the extracellular environmental signals into the cell, and it is a positive regulator of plant tolerance to various stresses[152]. All gymnosperms, especially conifers, contain large numbers of genes for G-type lectin S-receptor-like serine/threonine-protein kinases compared to seedless plants. Cysteine-rich RLKs are an important class of RLK that play vital roles in disease resistance and cell death in plants[153]. A significant expansion (48) of cysteine-rich RLK occurred in gymnosperms compared to seedless plants (0-4 copies).

15. Analysis of the terpene synthase gene family in *C. panzhihuaensis*

Plant terpenes are secondary metabolites, synthesized as a result of selective adaptation to multiple ecological niches. In plants, terpene synthase (TPS) genes form a mid-sized gene family, and they synthesize a diverse array of terpenes[154]. We identified the TPS genes using two HMMER models of two conserved domain sequences, the TPS N-terminal domain (PF01397) and the TPS metal-binding domain (PF03936), as search queries against the proteomes of 16 selected species. The search results were combined, overlapping sequences were filtered, and each sequence was manually curated to ensure that the gene length was accurate. Additionally, we also double checked whether all the obtained TPS sequences contain both PF01397 and PF03936 domains.

Through careful manual curation, we identified 38 TPS genes in the *C. panzhihuaensis* genome. The TPS-a clade, TPS-b/g clade, and TPS-c/f/e/h clade, all of which were previously established subfamilies, were clearly found in our phylogenetic tree (Supplementary Table 49). Interestingly, we found that three TPS genes of *C. panzhihuaensis* and eight TPS genes of *C. debaoensis* clustered together and are sister to a clade composing the TPS-a clade and TPS-b/g clade, indicating the existence of a novel TPS subfamily in *Cycas*; this TPS subfamily displayed high similarity with the TPS-a clade and TPS-b/g clade. TPS-d genes were gymnosperm-specific. Our phylogenetic tree placed TPS-d into four main clades (TPS-d1-d4). The TPS-d1 clade

was sister to the TPS-c/f/e/h clade. The *C. panzhihuaensis* genome encodes nine TPS-d1 copies, similar to the number in *G. biloba* (seven). No sequence of *G. montanum* was found in the TPS-d1 clade, suggesting that *G. montanum* may have lost TPS-d1 homologs. We found 19 and 36 homologs of TPS-d2 in the *C. panzhihuaensis* and *C. debaoensis* genomes, respectively, which is higher than the TPS-d2 number in *G. biloba* (11). Neither *G. montanum* nor *S. giganteum* appears to encode TPS-d2 proteins. Partial sequences of *C. debaoensis* and *G. biloba* clustered together and formed a small clade in the TPS-d2 clade. In the TPS-d3 clade, sequences were grouped according to their respective taxa, for example, sequences from *G. biloba* grouped together to form a small clade (TPS-d3-*G. biloba*). Sequences from *G. montanum* grouped together to form another small clade (TPS-d3-*G. montanum*). Likewise, gene sequences from Conifers I (*P. abies*, *P. taeda*, *P. lambertiana*, *P. menzesii*) also grouped together and formed TPS-d3-Conifers I. However, the gene sequences of *S. giganteum* grouped into two small clades, one clustered with TPS-d3-*G. montanum*, while another clade clustered with the TPS-d3-*G. biloba*. We did not observe gene sequences from *C. panzhihuaensis* and *C. debaoensis* in the TPS-d3. The TPS-d4 clade consisted of the sequences from conifers and *G. biloba*. No sequences of either *G. montanum* or *Cycas* were found in the TPS-d4 clade. Interestingly, gene sequences of Conifers I, Conifers II, and *G. biloba* formed three small clades with their respective taxa, i.e., TPS-d4-Conifers I clade, TPS-d4-*S. giganteum*, and TPS-d4-*G. biloba*.

To further understand the function and physiology of TPS in *C. panzhihuaensis*, we compared gene expression levels of all TPS genes of *C. panzhihuaensis* in different tissues. 1) Many TPS genes were not expressed in stem and apical meristem of the stem; 2) most TPS genes were mainly expressed in higher amounts in roots (especially in primary root and coralloid roots), microsporophylls and pollen sac, and late-stage pollinated ovule and fertilized ovule; 3) three *Cycas*-specific TPS genes were mainly expressed in roots and male cone. Notably, one gene (CYCAS_009486) was specifically highly expressed in megagametophyte and post-pollinated and post-fertilized ovule. In plant terpene biosynthesis, oxidation of the hydrocarbon backbone produced by terpene synthases is typically carried out by cytochrome P450 oxygenases (CYPs). In recent years, there has been a significant increase in reports of CYPs involved in plant terpene pathways[98]. Therefore, to further understand the evolution of terpenoids in gymnosperms, we analyzed and summarized the number of various CYPs

across the sampled plant genomes. Gymnosperm genomes encoded a large number of CYPs compared to seedless plants and angiosperms, though most of CYPs are unclassified, which suggested potential diversity of secondary metabolites in gymnosperms (Supplementary Table 50).

16. Horizontal gene transfer and evolution of toxin genes in the *C. panzhihuaensis* genome

16.1 Identification of the horizontally transferred cytotoxin genes in *C. panzhihuaensis*

Horizontal gene transfer can accelerate biological evolution[155]. In this study, we identified horizontally transferred genes in *Cycas* and identified three gene families including four cytotoxin protein-encoding genes, which likely were obtained from fungi and confer resistance to insects. The upstream and downstream genes of the four toxin genes are all clearly plant genes, and we found no abnormality in read-mapping quality, demonstrating that the sequence is free from bacterial contamination. The cytotoxin protein consists of 2,000–3,000 amino acids. The gene in *C. panzhihuaensis* is very similar to that found in *Pseudomonas aeruginosa* (evalue = 0; bit score = 1,283), a Gram-negative bacterium, which can opportunistically infect bacteria and also plants[156]. It has been shown that this cytotoxin can be a protein toxic to insects in *Pseudomonas fluorescens*[157]. In addition, the cytotoxin gene was also reported to have been transferred into the fungus *Epichloë*, species of which are intercellular symbionts of grasses, and made the grass hosts resistant to insects[158].

In this study, we found for the first time that the cytotoxin was also transferred to *Cycas*. Both *C. panzhihuaensis* and *C. debaoensis* genomes encode this toxin protein. We performed phylogenetic analysis to understand the probable origin of the toxin protein. At least one sequence was selected from each subgroup of bacteria and fungi in blast search results for further phylogenetic analyses. These sequences from each subgroup were then used as queries to search the nr database to gain sufficient and balanced sampling. Protein sequences of *Mcl* from representative taxonomic groups were downloaded from nr blast results and sampled for phylogenetic analyses. Multiple sequence alignments were carefully inspected and adjusted before rigorous phylogenetic analyses were performed. To check whether the cytotoxin protein is due

to sequence contamination or a gene in the *Cycas* nuclear genome, we searched for homologs of the cytotoxin gene in our transcriptomes of cycads, which include all 10 cycad genera (i.e., *Cycas*, *Dioon*, *Macrozamia*, *Lepidozamia*, *Encephalartos*, *Bowenia*, *Ceratozamia*, *Stangeria*, *Microcycas*, and *Zamia*). Interestingly, we found that genes encoding the toxin proteins were only present in *Cycas*, and not in any other cycad genera (Supplementary Table 51). Combining the genome and transcriptome search results, we inferred that these cytotoxin-encoding genes were likely a result of horizontal gene transfer (HGT) from fungi to *C. panzhihuaensis*.

We further investigated the gene expression profiles of the four cytotoxin genes in various tissues of *C. panzhihuaensis*. Interestingly, all the toxin genes displayed similar expression patterns. They were mainly expressed in all three types of roots (notably, CYCAS_004918 was only expressed in precoralloid roots), male cones (microsporophylls and pollen sac), unpollinated ovules, and the early stage of pollinated ovules, and embryo. Horizontally transferred toxic genes could be an important way for plants to gain resistance to insects. Similarly, a gene transferred from *Chloroflexi* bacteria to ferns confers resistance to whitefly damage[159]. Interestingly, there were also gene transfer events from plants to whitefly, permitting insects to detoxify plant toxins[160]. Horizontal gene transfer is a natural transgenic process, and these transferred genes are usually harmless to plants, indicating that these genes can be potentially used in crop breeding.

16.2 Experimental verification of the function of cytotoxin

The diamondback moth *Plutella xylostella* and cotton bollworm *Helicoverpa armigera* were obtained from the PilotScale Base of Bio-Pesticides, Institute of Zoology, Chinese Academy of Sciences. All stages of *H. armigera* were maintained at 27 ± 1 °C and a relative humidity of 40%–60% on an artificial diet with a light : dark photoperiod of 16 : 8 h. To avoid cannibalism, larvae were raised separately in a 5.0 cm × 1.5 cm glass tube after the 3rd instar. *Plutella xylostella* was maintained at the same conditions but fed on radish seedlings (*Raphanus sativus* L.). Adults of both insects were fed with 10% honey solution for supplemental nutrition. Two-day-old 3rd instars of *P. xylostella* and *H. armigera*, with an average body weight of 1.33 mg/individual and 2.06 mg/individual, respectively, were used for the experiments. For *P. xylostella*, 2.03 μg cytoxin protein was injected per larva, and for *H. armigera*, 3.38

μg was injected. Larvae injected with phosphate buffer saline (PBS) were used as controls. In all, 30 larvae were injected for each replicate, and three replicates were carried out. The mortalities were recorded 24 h post injection (Fig. 5, main texts).

17. Transcriptome, phytohormones, and metabolome analyses of roots of *C. panzhihuaensis*

17.1 Comparative transcriptomics among the primary root, precoralloid roots, and coralloid roots of *C. panzhihuaensis*, respectively

An interesting feature of *C. panzhihuaensis* plants is their extraordinary coralloid roots which contain cyanobacteria[161]. To investigate the difference in physiological characteristics between primary roots and coralloid roots, as well as the possible factors triggering cyanobacterial symbiosis in coralloid roots, we performed comparative transcriptomics on the three tissues. We found that 1,126 and 3,104 genes were specifically expressed in primary roots and precoralloid roots, respectively. These precoralloid roots (without cyanobacteria) specifically expressed genes that were significantly enriched in many biological processes, such as ATP metabolic process, organophosphate metabolic process, carbohydrate derivative metabolic process, nucleotide metabolic process, cation transport, and small molecule metabolic process. In contrast, primary root-specific expressed genes were significantly enriched in molecular functions associated with plant-type cell wall organization, cell wall organization or biogenesis, DNA replication, and molybdate ion transport (Supplementary Table 52; Supplementary Fig. 41).

Additionally, in a comparison of gene expression between precoralloid roots and primary roots, we identified 2,667 genes that were more highly expressed (two-fold higher) in precoralloid roots than in primary roots. These highly expressed genes in precoralloid roots were significantly enriched for plant-type cell wall organization, oxidation-reduction process, single-organism metabolic process, and anion transport. In addition, 1,745 genes showed down-regulated expression in precoralloid roots compared to primary roots; these genes were mainly associated with basic biological process, e.g., purine ribonucleoside metabolic process, nucleotide metabolic process, ATP metabolic process, nucleoside triphosphate metabolic process, organophosphate biosynthetic process, and organonitrogen compound metabolic process, but some genes

possibly involved in response to stress, response to stimulus, and response to wounding also appeared to be down-regulated in precoralloid roots.

To understand how symbiotic cyanobacteria affect the physiology and metabolism of coralloid roots of the host *C. panzhihuaensis*, we compared gene expression profiles between coralloid roots (with cyanobacteria) and precoralloid roots. We found that 1,480 genes were specifically expressed in precoralloid roots, versus 1,005 genes that were specifically expressed in coralloid roots with cyanobacteria. GO enrichment analyses in the coralloid roots with cyanobacteria-specific expressed genes revealed that these genes were enriched in biological processes related to defense response and microtubule-based movement. The 1,480 genes specifically expressed in precoralloid roots were significantly enriched in various biological processes (such as cell wall organization or biogenesis, photosynthesis, oxidation-reduction process, response to oxidative stress, and multi-organism process). We found 938 genes of precoralloid roots showed two-fold up-regulated expression compared to that of coralloid roots (with cyanobacteria). GO enrichment analyses of these genes suggested that they might encode components of various biological processes (e.g., cell wall organization or biogenesis, plant-type cell wall organization or biogenesis, cellular component organization, oxidation-reduction process, photosynthesis, single-organism metabolic process, metabolic process, and response to oxidative stress). Conversely, coralloid roots (with cyanobacteria) had 559 genes that showed more than two-fold greater expression than that in precoralloid roots. These genes were significantly enriched in biological processes related to stress response, response to wounding, response to stimulus, oxidation-reduction process, and single-organism metabolic process.

17.2 Phytohormone analyses among the primary root and coralloid roots *C. panzhihuaensis* with and without cyanobacteria

We also performed phytohormone quantification of the three root types. Ethylene, gibberellin, salicylic acid, abscisic acid, and auxin showed similar trends, while the precoralloid roots produced a much higher amount of these five phytohormones than primary roots and coralloid roots. Primary roots accumulated more jasmonic acid and trans-zeatin-riboside than the other two kinds of roots (Supplementary Table 53; Supplementary Fig. 42).

17.3 Metabolomic analyses among the primary root and coralloid roots of *C. panzhihuaensis* with and without cyanobacteria

We compared metabolomic changes of different *Cycas* root tissues, i.e., the primary roots, precoralloid roots, and coralloid roots. Compared to the coralloid root, three metabolites accumulated at a level 10-fold higher in the primary root, i.e., gibberellin A4, imidazol-5-yl-pyruvate, and biocytin, and 19 metabolites accumulated at a level 10-fold higher in the primary root, e.g., biocytin, gibberellin A4, perillyl alcohol, glutarate semialdehyde, 2-methoxy-4-vinylphenol, and (S)-reticuline. Forty-seven metabolites accumulated at a level that was 10-fold higher in coralloid roots than in precoralloid roots (Supplementary Table 54).

17.4 Phylogenomic analyses of the symbiosis-related genes

Cycads are known to have the ability to have dual symbiosis (cyanobacterial symbiosis and mycorrhizal symbiosis)[162]. To investigate the symbiosis-related genes, and to compare these among cycads and other plants with various types of symbiosis, 34 symbiosis-related genes were analyzed for further investigation. The *C. panzhihuaensis* genome encodes 32 of these 34 genes. First, we compared the distribution of these genes between representative arbuscular mycorrhizal symbiotic (AMS) species and *C. panzhihuaensis*. *KinF* and *HYP3* were lost in *C. panzhihuaensis*, indicating these genes might be dispensable for arbuscular mycorrhizal symbiosis in *C. panzhihuaensis* (Supplementary Table 55). Second, although arbuscular mycorrhizal symbiosis and root-nodule symbiosis showed distinct differences, they require a common symbiosis pathway (CSP), and previous studies have shown that the key *CSP* genes (*RAD1*, *STR1/STR2*, *RAM1*, *CCaMK*, *CYCLOPS*, *SymRK*, *VAPYRIN*, *CASTOR/POLLUX*) are dispensable for the nitrogen-fixing symbiosis between *Azolla* and *Nostoc*[159]. Both *Cycas* and the hornwort *Anthoceros* are symbiotic with cyanobacteria and encode these CSP genes (also known as "key angiosperm AMF symbiosis genes")[45,163]. Third, the *A. punctatus* genome contains 27 of 34 CSP genes. The absence of seven symbiosis-related genes (*ABCB20*, *HYP4*, *EPP1*, *HYP3*, *KinG1/KinG2*, *NFP*, and *SYN*) in *A. punctatus* suggests that they are not required for with cyanobacterial symbiosis with *A. punctatus*. Fourth, we checked the expression levels of these 32 genes in three root types in *C. panzhihuaensis*. We found that the expression level of 10 genes (*RAD1*, *DHY*, *SymRK*, *EPP1*, V*APYRIN*, *CASTOR/POLLUX*, *NFP*, *CYTB561*, *GRAS*, *HEP*) in

precoralloid roots is over two-fold up-regulated compared to coralloid roots with cyanobacteria, while five genes in coralloid roots with cyanobacteria (*CCaMK*, *CYCLOPS*, *LIN*, *SYN* and *TAU*) showed significantly higher expression than the precoralloid roots.

We also identified conserved cyanobacterial symbiotic associated genes shared among cyanobacteria symbiotic plants, including *C. panzhihuaensis*, *Azolla filiculoides*, *Blasia pusilla*, *Anthoceros punctatus*, and *Azolla cf. caroliniana* and found that these genes have putative functions as trihelix transcription factor ASIL2, anion exchange protein 3 (AE 3), zinc finger protein, etc. (Supplemantary Table 56).

17.5 Identification of the cyanobacterial-derived genes in the *C. panzhihuaensis* nuclear genome

A thorough similarity search found three candidate cyanobacterial-derived genes in *C. panzhihuaensis*, including heme uptake and utilization protein huvX, DUF1825 family, and DEAD/DEAH box helicase. Our phylogenetic analysis indicates that these three candidate genes were acquired by horizontal gene transfer from cyanobacteria to *C. panzhihuaensis*. Hemin and hemoglobin serve as iron sources for microbes[164]. HuvX is an outer membrane receptor involved in heme uptake as an iron source in bacteria[165]. The cyanobacterial-derived HuvX gene found in *C. panzhihuaensis* might contribute to iron nutrient metabolism. The DUF1825 domain-containing protein belongs to an uncharacterized family of proteins principally found in cyanobacteria; the possible function of this gene needs further investigation. DEAD/DEAH-box helicases are enzymes that belong to the DEAD/H-box family of the SF2 helicase superfamily[166]. These enzymes are essential in RNA metabolism[167]. Recent studies have found that some DEAD/DEAH-box helicases play important roles in innate immunity. In bacteria, DEAD/DEAH-box helicases play a role in limitation of ribosome biogenesis, mRNA degradation, and possibly translation initiation. Cyanobacterial-derived DEAD/DEAH-box helicases may have a similar function in *C. panzhihuaensis* (Supplementary Fig. 43).

18. Expansion of cell cycle related genes in *C. panzhihuaensis*

Transcription factor *E2F*s are involved in control of the G1/S transition in animals and plants by mediating the transcriptional activation of many genes required for cell-cycle

progression and DNA replication. E2F and dimerization partner (DP) proteins form heterodimers and bind to the E2F-binding sites in target promoters[168]. Given the specifically huge increase of *E2F* transcription factor genes in *C. panzhihuaensis*, we further investigated cell-cycle-related genes (especially for the *E2F-RBR* pathway). Interestingly, we found three gene families related to the cell cycle (CYCT, CYCU/CYCP, and CDKC) to be expanded in *C. panzhihuaensis* and most gymnosperms. The expansion of the *CYCU/CYCP* gene family apparently occurred in all gymnosperms compared to seedless plants, while the expansion of *CYCT* and *CDKC* only occurred in some gymnosperms. For example, the *C. panzhihuaensis* genome encodes 19 homologs of *CYCT*. However, four, six, and nine copies of *CYCT*s were identified in *G. montanum*, *P. lambertiana*, and *G. biloba*, respectively, numbers that are similar to seedless plants. Markedly, we found 35 homologs of *CDKC* in the *C. panzhihuaensis* genome; as with the E2F transcription factors, *CDKC* also showed an increase in *C. panzhihuaensis*. Further phylogenetic analysis of the expanded *CDKC* gene family in *C. panzhihuaensis* showed that a large number of *CDKC* genes from *C. panzhihuaensis* clustered together and formed a separate clade, with one *CDKC* homolog from *G. montanum* also belonging to this new clade (no other gymnosperm homologs were included in this clade) (Supplementary Fig. 44; Supplementary Table 57).

19. Expansion of other gene families

We also found many expanded gene families that are associated with various plant physiological functions (Supplementary Table 58). *S-adenosylmethionine decarboxylase* (*SAMDC*) is necessary for biosynthesis of the polyamines spermidine and spermine. It plays a vital role in polyamine homeostasis and normal embryogenesis, growth, and development in plants[169]. *Cycas* and some species of *Picea* harbor more copies of SAMDC than other plants (including the seedless plants). Additionally, SAMDC and its orthologous genes are involved in responses of plants to biotrophic pathogens. For example, *CmSAMDC* shows a steep rise in its expression level when exposed to biotic stress; over-expression in *Cucumis melo* resulted in greatly reduced pathogen infection[170].

The *C. panzhihuaensis* genome contains 28 copies of THO complex subunit genes, which is more than two-fold higher than in the seedless plants examined. The transcription-export (THO/TREX) protein complex plays an important role in coupling

transcription with splicing and export of mRNA[171]. In *Arabidopsis*, that the THO/TREX complex functions in siRNA biosynthesis and plays an important role in plant innate immunity[149,172]. Thus, we speculate that the expansion of the THO complex genes in *Cycas* might aid in disease resistance.

Cycas, *Ginkgo*, and Pinaceae encode higher copy numbers of genes encoding the stem-specific protein TSJT1 than seedless plants. Stem-specific protein TSJT1 acts as a negative regulator in internode development[173]. However, little is known about the function of the stem-specific protein TSJT1 in plant growth and development; thus, the specific and detailed function of this expanded gene family in *Cycas*, *Ginkgo*, and Pinaceae needs further study.

We found that *C. panzhihuaensis* has nine genes that encode the Fantastic Four meristem regulator. In contrast, one and three copies of this gene were detected in the genomes of *A. filiculoides* and *S. moellendorffii*, respectively, and it is absent in *P. patens*[174]. The Fantastic Four proteins participate in regulating the size of the shoot meristem by modulating the CLV3-WUS feedback loop[174]. Considering the remarkable expansion of WUS transcription factors in *Cycas* compared to seedless plants, it is reasonable to speculate that a more complicated regulation network of shoot meristem development might exist in *Cycas*.

We also identified the expansion of the genes encoding the flowering physiology-related gene, FRIGIDA (FRI), in *C. panzhihuaensis* and Pinaceae, but not in *G. montanum, S. giganteum,* and *G. biloba* compared to seedless plants. FRI is required for the regulation of flowering time in the late-flowering phenotype of *Arabidopsis*[175]. Recent study suggests that homeologous FRI-like genes are involved in reproductive development and somatic embryogenesis of plants[176]. Thus, the expansion of FRI in gymnosperms might function in embryogenesis and the reproductive process.

A species-specific expansion of genes for 6-phosphogluconolactonase (6PGL) was found in the *C. panzhihuaensis* genome compared to other sampled plants. 6PGL catalyzes the hydrolysis of 6-phosphogluconolactone to 6-phosphogluconate in the pentose phosphate pathway and the regulation of the cellular redox state[177]. Thus, the expansion of 6PGL genes might contribute to the sugar-dependent expression of nitrate assimilation genes in root cells and play a crucial role in plant growth and development via the pentose phosphate pathway[178].

Finally, we identified more genes for aspartyl protease family proteins in the genomes of *C. panzhihuaensis* and some species of Pinaceae compared to seedless

plants. However, *G. montanum*, *S. giganteum*, and *G. biloba* did not exhibit an expansion of aspartic protease family proteins. Aspartic protease is one of the four large proteolytic enzyme families involved in plant growth and development[179]. Although it has been characterized in *Arabidopsis*, rice, and grape, very little is known about the aspartic protease family in gymnosperm species[180].

In barley, aspartic protease was found to participate in the autolysis of tracheary elements and sieve cells[179]. In addition, aspartic protease also plays a role in general physiology, such as protein processing, signal transduction, and stress responses[181]. Notably, a recent study reports ASPG1 encodes an aspartic protease that might be involved in *Arabidopsis* seed dormancy, seed longevity, and seed germination, which are all associated with the degradation of seed storage proteins and regulation of gibberellic acid signaling[182]. In addition, aspartic proteases also function in plant programmed cell death and disease resistance[183]. Interestingly, a recent study found that aspartic endopeptidases play an important role in the prevention of polytubey in *Arabidopsis*[184]. After successful fertilization, aspartic endopeptidases ECS1 and ECS2 are released in extracellular space and are exclusively expressed in the egg cell; transcripts are immediately degraded after gamete fusion. ECS1 and ECS2 are usually released by the fertilized egg cells to avoid polytubey. As *Cycas* is able to grow multiple pollen tubes for fertilization, it may need this enzyme to avoid polytubey. In summary, expansion of aspartic protease genes (100+ copy number) in *C. panzhihuaensis* might suggest increased functional diversity for physiology, growth, and development. Aspartic proteases were speculated to degrade a specific substrate and produce some substances necessary for pollen germination and pollen tube growth[185]. In addition, aspartic proteinase 39 coordinates with A36 and contributes to pollen and ovule development, including the apical cell wall constitution of the growing pollen tubes[186].

Furthermore, we discovered that the NmrA-like gene family, which includes bifunctional pinoresinol-lariciresinol reductase (PLR-Tp2) and phenylcoumaran benzylic ether reductase/Isoeugenol synthase (PCBER), experienced expansion in all gymnosperms when compared to seedless plants. The NmrA-like proteins were reported to regulate gene expression in *Phytophthora* to impact the infection cycle in tomato and play an important role in defense of this plant oomycete pathogen[187].

Supplementary References

1 Jones, D. L. *Cycads of the World-Ancient plant in today's landscape. Washington*. (Smithsonian Institution Press, 2002).

2 Calonje, M., Stevenson, D. & Osborne, R. The World List of Cycads, online edition. 2013-2021. Available from: http://www.cycadlist.org. (2021).

3 Butler, R. J., Barrett, P. M., Kenrick, P. & Penn, M. G. Testing co-evolutionary hypotheses over geological timescales: interactions between Mesozoic non-avian dinosaurs and cycads. *Biological Reviews* **84**, 73-89 (2009).

4 Condamine, F. L., Nagalingum, N. S., Marshall, C. R. & Morlon, H. Origin and diversification of living cycads: a cautionary tale on the impact of the branching process prior in Bayesian molecular dating. *BMC Evolutionary Biology* **15**, 1-18 (2015).

5 Raven, P. H., Evert, R. F. & Eichhorn, S. E. *Biology of plants*. (Macmillan, 2005).

6 Norstog, T. & Nicholls, K. The biology of the cycads Cornell University Press Ithaca. *New York* (1997).

7 Yang, Y.-Q., Huang, B.-H., Yu, Z.-X. & Liao, P.-C. Inferences of demographic history and fine-scale landscape genetics in *Cycas panzhihuaensis* and implications for its conservation. *Tree Genetics & Genomes* **11**, 1-15 (2015).

8 Xiao, S., Ji, Y., Liu, J. & Gong, X. Genetic characterization of the entire range of *Cycas panzhihuaensis* (Cycadaceae). *Plant Diversity* **42**, 7-18 (2020).

9 Liu, J. *et al.* Phylogeny of the gymnosperm genus *Cycas*L.(Cycadaceae) as inferred from plastid and nuclear loci based on a large-scale sampling: Evolutionary relationships and taxonomical implications. *Molecular Phylogenetics and Evolution* **127**, 87-97 (2018).

10 Xiao, L.-Q. & Möller, M. Nuclear ribosomal its functional paralogs resolve the phylogenetic relationships of a late-Miocene radiation cycad *Cycas* (Cycadaceae). *PloS ONE* **10**, e0117971 (2015).

11 Schnable, P. S. *et al.* The B73 maize genome: complexity, diversity, and dynamics. *Science* **326**, 1112-1115 (2009).

12 Zonneveld, B. Genome sizes for all genera of Cycadales. *Plant Biology* **14**, 253-256 (2012).

13 Guan, R. *et al.* Draft genome of the living fossil *Ginkgo biloba*. *Gigascience* **5**, s13742-13016-10154-13741 (2016).

14 Wan, T. *et al.* A genome for gnetophytes and early evolution of seed plants. *Nature Plants* **4**, 82-89 (2018).

15 Friedländer, M. R., Mackowiak, S. D., Li, N., Chen, W. & Rajewsky, N. miRDeep2 accurately identifies known and hundreds of novel microRNA genes in seven animal clades. *Nucleic Acids Research* **40**, 37-52 (2012).

16 Bo, X. & Wang, S. TargetFinder: a software for antisense oligonucleotide target site selection based on MAST and secondary structures of target mRNA. *Bioinformatics* **21**, 1401-1402 (2005).

17 Emms, D. & Kelly, S. STAG: species tree inference from all genes. *BioRxiv*, 267914 (2018).

18 Leebens-Mack, J. H. *et al.* One thousand plant transcriptomes and the phylogenomics of green plants. *Nature* **574**, 679–685 (2019).

19 Mirarab, S. *et al.* ASTRAL: genome-scale coalescent-based species tree estimation. *Bioinformatics* **30**, i541-i548 (2014).

20 Johnson, M. G. *et al.* HybPiper: Extracting coding sequence and introns for phylogenetics from high-throughput sequencing reads using target enrichment. *Applications in Plant Sciences* **4**, 1600016 (2016).

21 Dierckxsens, N., Mardulyn, P. & Smits, G. NOVOPlasty: *de novo* assembly of organelle genomes from whole genome data. *Nucleic Acids Research* **45**, e18–e18 (2017).

22 Kim, D. *et al.* TopHat2: accurate alignment of transcriptomes in the presence of insertions, deletions and gene fusions. *Genome Biology* **14**, 1–13 (2013).

23 Langmead, B. & Salzberg, S. L. Fast gapped-read alignment with Bowtie 2. *Nature Methods* **9**, 357–359 (2012).

24 Kalyaanamoorthy, S., Minh, B. Q., Wong, T. K., Von Haeseler, A. & Jermiin, L. S. ModelFinder: fast model selection for accurate phylogenetic estimates. *Nature Methods* **14**, 587-589 (2017).

25 Nguyen, L.-T., Schmidt, H. A., Von Haeseler, A. & Minh, B. Q. IQ-TREE: a fast and effective stochastic algorithm for estimating maximum-likelihood phylogenies. *Molecular Biology and Evolution* **32**, 268-274 (2015).

26 Than, C., Ruths, D. & Nakhleh, L. PhyloNet: a software package for analyzing and reconstructing reticulate evolutionary relationships. *BMC Bioinformatics* **9**, 1–16 (2008).

27 Liu, L. & Yu, L. Phybase: an R package for species tree analysis. *Bioinformatics*

26, 962–963 (2010).

28 Martin, S. H. & Van Belleghem, S. M. Exploring evolutionary relationships across the genome using topology weighting. *Genetics* **206**, 429–438 (2017).

29 Yang, Y. *et al.* Prickly waterlily and rigid hornwort genomes shed light on early angiosperm evolution. *Nature Plants* **6**, 215–222 (2020).

30 Wang, K. *et al.* Incomplete lineage sorting rather than hybridization explains the inconsistent phylogeny of the wisent. *Communications Biology* **1**, 1–9 (2018).

31 Wilson, V. R. & Owens, J. N. Cytoplasmic inheritance in *Podocarpus totara* (Podocarpaceae). *Acta Horticulturae* (2003).

32 Rabosky, D. L. *et al.* BAMM tools: an R package for the analysis of evolutionary dynamics on phylogenetic trees. *Methods in Ecology and Evolution* **5**, 701-707 (2014).

33 Zachos, J., Pagani, M., Sloan, L., Thomas, E. & Billups, K. Trends, rhythms, and aberrations in global climate 65 Ma to present. *Science* **292**, 686-693 (2001).

34 Ran, J.-H., Shen, T.-T., Wang, M.-M. & Wang, X.-Q. Phylogenomics resolves the deep phylogeny of seed plants and indicates partial convergent or homoplastic evolution between Gnetales and angiosperms. *Proceedings of the Royal Society B* **285**, 20181012 (2018).

35 Wickett, N. J. *et al.* Phylotranscriptomic analysis of the origin and early diversification of land plants. *Proceedings of the National Academy of Sciences* **111**, E4859-E4868 (2014).

36 Gitzendanner, M. A., Soltis, P. S., Wong, G. K. S., Ruhfel, B. R. & Soltis, D. E. Plastid phylogenomic analysis of green plants: a billion years of evolutionary history. *American Journal of Botany* **105**, 291-301 (2018).

37 Nagalingum, N. *et al.* Recent synchronous radiation of a living fossil. *Science* **334**, 796-799 (2011).

38 Wang, Y. *et al.* MCScanX: a toolkit for detection and evolutionary analysis of gene synteny and collinearity. *Nucleic Acids Research* **40**, e49-e49 (2012).

39 Nei, M. & Gojobori, T. Simple methods for estimating the numbers of synonymous and nonsynonymous nucleotide substitutions. *Molecular Biology and evolution* **3**, 418-426 (1986).

40 Yang, Z. PAML: a program package for phylogenetic analysis by maximum likelihood. *Bioinformatics* **13**, 555-556 (1997).

41 Jiao, Y. *et al.* Ancestral polyploidy in seed plants and angiosperms. *Nature* **473**, 97-100 (2011).
42 Wu, S., Han, B. & Jiao, Y. Genetic contribution of paleopolyploidy to adaptive evolution in angiosperms. *Molecular Plant* **13**, 59-71 (2020).
43 Qiao, X. *et al.* Gene duplication and evolution in recurring polyploidization–diploidization cycles in plants. *Genome Biology* **20**, 1-23 (2019).
44 Finet, C. *et al.* Evolution of the YABBY gene family in seed plants. *Evolution & Development* **18**, 116-126 (2016).
45 Li, F.-W. *et al. Anthoceros* genomes illuminate the origin of land plants and the unique biology of hornworts. *Nature Plants* **6**, 259-272 (2020).
46 Nishimura, M. T. *et al.* Loss of a callose synthase results in salicylic acid-dependent disease resistance. *Science* **301**, 969-972 (2003).
47 Yamamoto, M., Nakatsuka, S., Otani, H., Kohmoto, K. & Nishimura, S. (+)-Catechin acts as an infection-inhibiting factor in strawberry leaf. *Phytopathology* **90**, 595-600 (2000).
48 Chen, Z., Liang, J., Zhang, C. & Rodrigues, C. J. Epicatechin and catechin may prevent coffee berry disease by inhibition of appressorial melanization of *Colletotrichum kahawae*. *Biotechnology Letters* **28**, 1637-1640 (2006).
49 Singh, D. P., Jermakow, A. M. & Swain, S. M. Gibberellins are required for seed development and pollen tube growth in *Arabidopsis*. *The Plant Cell* **14**, 3133-3147 (2002).
50 Gomez, M. D., Ventimilla, D., Sacristan, R. & Perez-Amador, M. A. Gibberellins regulate ovule integument development by interfering with the transcription factor ATS. *Plant Physiology* **172**, 2403-2415 (2016).
51 Weis, B. L., Kovacevic, J., Missbach, S. & Schleiff, E. Plant-specific features of ribosome biogenesis. *Trends in Plant Science* **20**, 729-740 (2015).
52 Nuccio, M. L. & Thomas, T. L. ATS1 and ATS3: two novel embryo-specific genes in *Arabidopsis thaliana*. *Plant Molecular Biology* **39**, 1153-1163 (1999).
53 Zhang, H. *et al.* dbCAN2: a meta server for automated carbohydrate-active enzyme annotation. *Nucleic Acids Research* **46**, W95-W101 (2018).
54 Harris, P. J. Primary and secondary plant cell walls: a comparative overview. *New Zealand Journal of Forestry Science* **36**, 36 (2006).
55 Sarkar, P., Bosneaga, E. & Auer, M. Plant cell walls throughout evolution: towards a molecular understanding of their design principles. *Journal of*

Experimental Botany **60**, 3615-3635 (2009).
56 Amos, R. A. & Mohnen, D. Critical review of plant cell wall matrix polysaccharide glycosyltransferase activities verified by heterologous protein expression. *Frontiers in Plant Science* **10**, 915 (2019).
57 Thomas, L. H. *et al.* Structure of cellulose microfibrils in primary cell walls from collenchyma. *Plant Physiology* **161**, 465-476 (2013).
58 Popper, Z. A. & Fry, S. C. Primary cell wall composition of pteridophytes and spermatophytes. *New Phytologist* **164**, 165-174 (2004).
59 Volkmann, D. & Baluška, F. Gravity: one of the driving forces for evolution. *Protoplasma* **229**, 143-148 (2006).
60 Miedes, E., Vanholme, R., Boerjan, W. & Molina, A. The role of the secondary cell wall in plant resistance to pathogens. *Frontiers in Plant Science* **5**, 358 (2014).
61 Liu, X. L. *et al.* MALE GAMETOPHYTE DEFECTIVE 4 encodes a rhamnogalacturonan II xylosyltransferase and is important for growth of pollen tubes and roots in *Arabidopsis*. *The Plant Journal* **65**, 647-660 (2011).
62 Dumont, M. *et al.* The cell wall pectic polymer rhamnogalacturonan-II is required for proper pollen tube elongation: implications of a putative sialyltransferase-like protein. *Annals of Botany* **114**, 1177-1188 (2014).
63 Caffall, K. H. & Mohnen, D. The structure, function, and biosynthesis of plant cell wall pectic polysaccharides. *Carbohydrate Research* **344**, 1879-1900 (2009).
64 Broxterman, S. E. & Schols, H. A. Interactions between pectin and cellulose in primary plant cell walls. *Carbohydrate polymers* **192**, 263-272 (2018).
65 Cosgrove, D. J. Plant cell wall extensibility: connecting plant cell growth with cell wall structure, mechanics, and the action of wall-modifying enzymes. *Journal of Experimental Botany* **67**, 463-476 (2016).
66 Lamport, D. T. in *Advances in Botanical Research* Vol. 2. 151-218 (Elsevier, 1966).
67 Cannon, M. C. *et al.* Self-assembly of the plant cell wall requires an extensin scaffold. *Proceedings of the National Academy of Sciences* **105**, 2226-2231 (2008).
68 Lamport, D. T. Oxygen fixation into hydroxyproline of plant cell wall protein. *Journal of Biological Chemistry* **238**, 1438-1440 (1963).

69 Yaqoob, A. *et al.* Dual functions of Expansin in cell wall extension and compression during cotton fiber development. *Biologia* **75**, 2093-2101 (2020).

70 Lipchinsky, A. How do expansins control plant growth? A model for cell wall loosening via defect migration in cellulose microfibrils. *Acta Physiologiae Plantarum* **35**, 3277-3284 (2013).

71 Pezzotti, M., Feron, R. & Mariani, C. Pollination modulates expression of the PPAL gene, a pistil-specific β-expansin. *Plant Molecular Biology* **49**, 187-197 (2002).

72 Schindelman, G. *et al.* COBRA encodes a putative GPI-anchored protein, which is polarly localized and necessary for oriented cell expansion in *Arabidopsis*. *Genes & Development* **15**, 1115-1127 (2001).

73 Li, S. *et al. Arabidopsis* COBRA-LIKE 10, a GPI-anchored protein, mediates directional growth of pollen tubes. *The Plant Journal* **74**, 486-497 (2013).

74 Park, J., Cui, Y. & Kang, B.-H. AtPGL3 is an *Arabidopsis* BURP domain protein that is localized to the cell wall and promotes cell enlargement. *Frontiers in Plant Science* **6**, 412 (2015).

75 Chrost, B., Kolukisaoglu, U., Schulz, B. & Krupinska, K. An α-galactosidase with an essential function during leaf development. *Planta* **225**, 311-320 (2007).

76 Sasidharan, R., Voesenek, L. A. & Pierik, R. Cell wall modifying proteins mediate plant acclimatization to biotic and abiotic stresses. *Critical Reviews in Plant Sciences* **30**, 548-562 (2011).

77 Miedes, E. *et al.* Xyloglucan endotransglucosylase/hydrolase (XTH) overexpression affects growth and cell wall mechanics in etiolated *Arabidopsis* hypocotyls. *Journal of Experimental Botany* **64**, 2481-2497 (2013).

78 Van Sandt, V. S., Suslov, D., Verbelen, J.-P. & Vissenberg, K. Xyloglucan endotransglucosylase activity loosens a plant cell wall. *Annals of Botany* **100**, 1467-1473 (2007).

79 Wang, H., Lockwood, S. K., Hoeltzel, M. F. & Schiefelbein, J. W. The ROOT HAIR DEFECTIVE3 gene encodes an evolutionarily conserved protein with GTP-binding motifs and is required for regulated cell enlargement in *Arabidopsis*. *Genes & Development* **11**, 799-811 (1997).

80 Punja, Z. K. & Zhang, Y.-Y. Plant chitinases and their roles in resistance to fungal diseases. *Journal of Nematology* **25**, 526 (1993).

81 Kundu, S. & Sharma, R. Origin, evolution, and divergence of plant class C GH9

endoglucanases. *BMC Evolutionary Biology* **18**, 1-19 (2018).
82 Iakiviak, M. *et al.* Functional and modular analyses of diverse endoglucanases from *Ruminococcus albus* 8, a specialist plant cell wall degrading bacterium. *Scientific Reports* **6**, 1-13 (2016).
83 Babu, Y. & Bayer, M. Plant polygalacturonases involved in cell elongation and separation—The same but different? *Plants* **3**, 613-623 (2014).
84 Dekker, N. *et al.* Role of the α-glucanase Agn1p in fission-yeast cell separation. *Molecular Biology of the Cell* **15**, 3903-3914 (2004).
85 Balasubramanian, V., Vashisht, D., Cletus, J. & Sakthivel, N. Plant β-1, 3-glucanases: their biological functions and transgenic expression against phytopathogenic fungi. *Biotechnology Letters* **34**, 1983-1990 (2012).
86 Schröder, R., Atkinson, R. G. & Redgwell, R. J. Re-interpreting the role of endo-β-mannanases as mannan endotransglycosylase/hydrolases in the plant cell wall. *Annals of Botany* **104**, 197-204 (2009).
87 Iglesias-Fernández, R., Rodríguez-Gacio, M. C., Barrero-Sicilia, C., Carbonero, P. & Matilla, A. Three endo-β-mannanase genes expressed in the micropylar endosperm and in the radicle influence germination of *Arabidopsis thaliana* seeds. *Planta* **233**, 25-36 (2011).
88 Escamilla-Treviño, L. L. *et al. Arabidopsis thaliana* β-glucosidases BGLU45 and BGLU46 hydrolyse monolignol glucosides. *Phytochemistry* **67**, 1651-1660 (2006).
89 Leah, R., Kigel, J., Svendsen, I. & Mundy, J. Biochemical and molecular characterization of a barley seed β-glucosidase. *Journal of Biological Chemistry* **270**, 15789-15797 (1995).
90 Lee, K. H. *et al.* Activation of glucosidase via stress-induced polymerization rapidly increases active pools of abscisic acid. *Cell* **126**, 1109-1120 (2006).
91 Morant, A. V. *et al.* β-Glucosidases as detonators of plant chemical defense. *Phytochemistry* **69**, 1795-1813 (2008).
92 Liu, Q., Luo, L. & Zheng, L. Lignins: biosynthesis and biological functions in plants. *International Journal of Molecular Sciences* **19**, 335 (2018).
93 Sáiz-Jiménez, C. & De Leeuw, J. Lignin pyrolysis products: their structures and their significance as biomarkers. *Organic Geochemistry* **10**, 869-876 (1986).
94 Prado, N. *et al.* Nanovesicles are secreted during pollen germination and pollen tube growth: a possible role in fertilization. *Molecular Plant* **7**, 573–577 (2014).

95 Greenberg, J. T. Programmed cell death: a way of life for plants. *Proceedings of the National Academy of Sciences* **93**, 12094-12097 (1996).

96 Xu, Y. *et al.* HOTHEAD-Like HTH1 is involved in anther cutin biosynthesis and is required for pollen fertility in rice. *Plant and Cell Physiology* **58**, 1238-1248 (2017).

97 Ma, H. Molecular genetic analyses of microsporogenesis and microgametogenesis in flowering plants. *Annu. Rev. Plant Biol.* **56**, 393-434 (2005).

98 Yang, X. *et al.* Rice CYP703A3, a cytochrome P450 hydroxylase, is essential for development of anther cuticle and pollen exine. *Journal of Integrative Plant Biology* **56**, 979-994 (2014).

99 Dudchenko, O. *et al.* De novo assembly of the *Aedes aegypti* genome using Hi-C yields chromosome-length scaffolds. *Science* **356**, 92–95 (2017).

100 Delcher, A. L., Salzberg, S. L. & Phillippy, A. M. Using MUMmer to identify similar regions in large sequence sets. *Current Protocols in Bioinformatics*, 10.13. 11–10.13. 18 (2003).

101 Dobin, A. *et al.* STAR: ultrafast universal RNA-seq aligner. *Bioinformatics* **29**, 15–21 (2013).

102 Robinson, J. T. *et al.* Integrative genomics viewer. *Nature Biotechnology* **29**, 24–26 (2011).

103 Segawa, M., KISHI, S. & TATUNO, S. Sex chromosomes of *Cycas revoluta*. *The Japanese Journal of Genetics* **46**, 33-39 (1971).

104 Abraham, A. & Mathew, P. Cytological studies in the cycads: sex chromosomes in *Cycas*. *Annals of Botany* **26**, 261-266 (1962).

105 Abascal, F., Zardoya, R. & Telford, M. J. TranslatorX: multiple alignment of nucleotide sequences guided by amino acid translations. *Nucleic Acids Research* **38**, W7–W13 (2010).

106 Katoh, K., Kuma, K.-i., Toh, H. & Miyata, T. MAFFT version 5: improvement in accuracy of multiple sequence alignment. *Nucleic Acids Research* **33**, 511–518 (2005).

107 Capella-Gutiérrez, S., Silla-Martínez, J. M. & Gabaldón, T. trimAl: a tool for automated alignment trimming in large-scale phylogenetic analyses. *Bioinformatics* **25**, 1972–1973 (2009).

108 Minh, B. Q. *et al.* IQ-TREE 2: new models and efficient methods for

phylogenetic inference in the genomic era. *Molecular Biology and Evolution* **37**, 1530–1534 (2020).

109 Yang, J., Lee, S. H., Goddard, M. E. & Visscher, P. M. GCTA: a tool for genome-wide complex trait analysis. *The American Journal of Human Genetics* **88**, 76-82 (2011).

110 Felsenstein, J. *PHYLIP (phylogeny inference package), version 3.5 c*. (Joseph Felsenstein., 1993).

111 Alexander, D. H., Novembre, J. & Lange, K. Fast model-based estimation of ancestry in unrelated individuals. *Genome Research* **19**, 1655-1664 (2009).

112 Jakobsson, M. & Rosenberg, N. A. CLUMPP: a cluster matching and permutation program for dealing with label switching and multimodality in analysis of population structure. *Bioinformatics* **23**, 1801-1806 (2007).

113 Danecek, P. *et al.* The variant call format and VCFtools. *Bioinformatics* **27**, 2156-2158 (2011).

114 Zhou, J.-M. & Zhang, Y. Plant immunity: danger perception and signaling. *Cell* (2020).

115 Sudisha, J., Sharathchandra, R., Amruthesh, K., Kumar, A. & Shetty, H. S. in *Plant defence: biological control* 379-403 (Springer, 2012).

116 Ali, S. *et al.* Pathogenesis-related proteins and peptides as promising tools for engineering plants with multiple stress tolerance. *Microbiological Research* **212**, 29-37 (2018).

117 Gao, X., Ruan, X., Sun, Y., Wang, X. & Feng, B. BAKing up to survive a battle: functional dynamics of BAK1 in plant programmed cell death. *Frontiers in Plant Science* **9**, 1913 (2019).

118 Araji, S. *et al.* Novel roles for the polyphenol oxidase enzyme in secondary metabolism and the regulation of cell death in walnut. *Plant Physiology* **164**, 1191-1203 (2014).

119 Fuerst, E. P., Okubara, P. A., Anderson, J. V. & Morris, C. F. Polyphenol oxidase as a biochemical seed defense mechanism. *Frontiers in Plant Science* **5**, 689 (2014).

120 Wrzaczek, M., Brosché, M., Kollist, H. & Kangasjärvi, J. *Arabidopsis* GRI is involved in the regulation of cell death induced by extracellular ROS. *Proceedings of the National Academy of Sciences* **106**, 5412-5417 (2009).

121 Rossi, F. R., Marina, M. & Pieckenstain, F. L. Role of arginine decarboxylase

(ADC) in *Arabidopsis thaliana* defence against the pathogenic bacterium Pseudomonas viridiflava. *Plant Biology* **17**, 831-839 (2015).

122 Li, L., He, Z., Pandey, G. K., Tsuchiya, T. & Luan, S. Functional cloning and characterization of a plant efflux carrier for multidrug and heavy metal detoxification. *Journal of Biological Chemistry* **277**, 5360-5368 (2002).

123 Roldán-Arjona, T. & Ariza, R. R. Repair and tolerance of oxidative DNA damage in plants. *Mutation Research/Reviews in Mutation Research* **681**, 169-179 (2009).

124 Donà, M. & Scheid, O. M. DNA damage repair in the context of plant chromatin. *Plant Physiology* **168**, 1206-1218 (2015).

125 White, M. D. *et al.* Structures of *Arabidopsis thaliana* oxygen-sensing plant cysteine oxidases 4 and 5 enable targeted manipulation of their activity. *Proceedings of the National Academy of Sciences* **117**, 23140-23147 (2020).

126 Mustroph, A. *et al.* Profiling translatomes of discrete cell populations resolves altered cellular priorities during hypoxia in *Arabidopsis*. *Proceedings of the National Academy of Sciences* **106**, 18843-18848 (2009).

127 Koiwa, H. *et al.* C-terminal domain phosphatase-like family members (AtCPLs) differentially regulate *Arabidopsis thaliana* abiotic stress signaling, growth, and development. *Proceedings of the National Academy of Sciences* **99**, 10893-10898 (2002).

128 Konopka-Postupolska, D. *et al.* The role of annexin 1 in drought stress in *Arabidopsis*. *Plant Physiology* **150**, 1394-1410 (2009).

129 Jung, Y. J. *et al.* Universal stress protein exhibits a redox-dependent chaperone function in *Arabidopsis* and enhances plant tolerance to heat shock and oxidative stress. *Frontiers in Plant Science* **6**, 1141 (2015).

130 Ding, X., Hou, X., Xie, K. & Xiong, L. Genome-wide identification of BURP domain-containing genes in rice reveals a gene family with diverse structures and responses to abiotic stresses. *Planta* **230**, 149-163 (2009).

131 Song, M. *et al.* Association of jacalin-related lectins with wheat responses to stresses revealed by transcriptional profiling. *Plant Molecular Biology* **84**, 95-110 (2014).

132 Giarola, V., Jung, N. U., Singh, A., Satpathy, P. & Bartels, D. Analysis of pcC13-62 promoters predicts a link between cis-element variations and desiccation tolerance in Linderniaceae. *Journal of Experimental Botany* **69**, 3773-3784

(2018).

133 Quesada, V. The roles of mitochondrial transcription termination factors (MTERFs) in plants. *Physiologia Plantarum* **157**, 389-399 (2016).

134 Shanmugabalaji, V., Grimm, B. & Kessler, F. Characterization of a Plastoglobule-Localized SOUL4 Heme-Binding Protein in *Arabidopsis thaliana*. *Frontiers in Plant Science* **11**, 2 (2020).

135 Fortunato, A. E., Sordino, P. & Andreakis, N. Evolution of the SOUL heme-binding protein superfamily across eukarya. *Journal of Molecular Evolution* **82**, 279-290 (2016).

136 Jing, Y. & Lin, R. The VQ motif-containing protein family of plant-specific transcriptional regulators. *Plant Physiology* **169**, 371-378 (2015).

137 Yen, S.-K., Chung, M.-C., Chen, P.-C. & Yen, H. E. Environmental and developmental regulation of the wound-induced cell wall protein WI12 in the halophyte ice plant. *Plant Physiology* **127**, 517-528 (2001).

138 Zhang, C., Hao, Z., Ning, Y. & Wang, G.-L. SINA E3 ubiquitin ligases: Versatile moderators of plant growth and stress response. *Molecular Plant* **12**, 610-612 (2019).

139 Iusem, N. D., Bartholomew, D. M., Hitz, W. D. & Scolnik, P. A. Tomato (*Lycopersicon esculentum*) transcript induced by water deficit and ripening. *Plant Physiology* **102**, 1353 (1993).

140 Hong, S. H., Kim, I. J., Yang, D. C. & Chung, W. I. Characterization of an abscisic acid responsive gene homologue from *Cucumis melo*. *Journal of Experimental Botany* **53**, 2271-2272 (2002).

141 Sun, P. *et al.* A novel role for banana MaASR in the regulation of flowering time in transgenic *Arabidopsis*. *PloS ONE* **11**, e0160690 (2016).

142 Valueva, T., Revina, T., Gvozdeva, E., Gerasimova, N. & Ozeretskovskaya, O. Role of protease inhibitors in potato protection. *Russian Journal of Bioorganic Chemistry* **29**, 454-458 (2003).

143 Turra, D. & Lorito, M. Potato type I and II proteinase inhibitors: modulating plant physiology and host resistance. *Current Protein and Peptide Science* **12**, 374-385 (2011).

144 Herrmann, A. *et al.* Proteoglycans from Boswellia serrata Roxb. and B. carteri Birdw. and identification of a proteolytic plant basic secretory protein. *Glycobiology* **22**, 1424-1439 (2012).

145 Xu, Y. *et al.* Molecular evidence for origin, diversification and ancient gene duplication of plant subtilases (SBTs). *Scientific Reports* **9**, 1-10 (2019).

146 Tang, Y., Liu, Q., Liu, Y., Zhang, L. & Ding, W. Overexpression of NtPR-Q up-regulates multiple defense-related genes in *Nicotiana tabacum* and enhances plant resistance to *Ralstonia solanacearum*. *Frontiers in Plant Science* **8**, 1963 (2017).

147 Pan, H., Liu, S. & Tang, D. HPR1, a component of the THO/TREX complex, plays an important role in disease resistance and senescence in *Arabidopsis*. *The Plant Journal* **69**, 831-843 (2012).

148 Mallory, A. & Vaucheret, H. Form, function, and regulation of ARGONAUTE proteins. *The Plant Cell* **22**, 3879-3889 (2010).

149 Heath, C. G., Viphakone, N. & Wilson, S. A. The role of TREX in gene expression and disease. *Biochemical Journal* **473**, 2911-2935 (2016).

150 Zeng, H. *et al.* Involvement of calmodulin and calmodulin-like proteins in plant responses to abiotic stresses. *Frontiers in Plant Science* **6**, 600 (2015).

151 Reddy, V. S., Ali, G. S. & Reddy, A. Characterization of a pathogen-induced calmodulin-binding protein: mapping of four Ca 2+-dependent calmodulin-binding domains. *Plant Molecular Biology* **52**, 143-159 (2003).

152 Ye, Y. *et al.* The role of receptor-like protein kinases (RLKs) in abiotic stress response in plants. *Plant Cell Reports* **36**, 235-242 (2017).

153 Yadeta, K. A. *et al.* A cysteine-rich protein kinase associates with a membrane immune complex and the cysteine residues are required for cell death. *Plant Physiology* **173**, 771-787 (2017).

154 Chen, F., Tholl, D., Bohlmann, J. & Pichersky, E. The family of terpene synthases in plants: a mid-size family of genes for specialized metabolism that is highly diversified throughout the kingdom. *The Plant Journal* **66**, 212–229 (2011).

155 Jain, R., Rivera, M. C., Moore, J. E. & Lake, J. A. Horizontal gene transfer accelerates genome innovation and evolution. *Molecular Biology and Evolution* **20**, 1598-1602 (2003).

156 Péchy-Tarr, M. *et al.* Molecular analysis of a novel gene cluster encoding an insect toxin in plant-associated strains of *Pseudomonas fluorescens*. *Environmental Microbiology* **10**, 2368–2386 (2008).

157 Péchy-Tarr, M. *et al.* Molecular analysis of a novel gene cluster encoding an

insect toxin in plant-associated strains of Pseudomonas fluorescens. *Environmental Microbiology* **10**, 2368-2386 (2008).

158 Ambrose, K. V., Koppenhöfer, A. M. & Belanger, F. C. Horizontal gene transfer of a bacterial insect toxin gene into the Epichloë fungal symbionts of grasses. *Scientific Reports* **4**, 1-8 (2014).

159 Li, F.-W. *et al.* Fern genomes elucidate land plant evolution and cyanobacterial symbioses. *Nature Plants* **4**, 460-472 (2018).

160 Xia, J. *et al.* Whitefly hijacks a plant detoxification gene that neutralizes plant toxins. *Cell* **184**, 1693-1705. e1617 (2021).

161 Chang, A. C. G., Chen, T., Li, N. & Duan, J. Perspectives on endosymbiosis in coralloid roots: association of cycads and cyanobacteria. *Frontiers in Microbiology* **10**, 1888 (2019).

162 Gutiérrez-García, K. *et al.* Cycad coralloid roots contain bacterial communities including cyanobacteria and *Caulobacter* spp. that encode niche-specific biosynthetic gene clusters. *Genome Biology and Evolution* **11**, 319-334 (2019).

163 Radhakrishnan, G. V. *et al.* An ancestral signalling pathway is conserved in intracellular symbioses-forming plant lineages. *Nature Plants* **6**, 280-289 (2020).

164 Noya, F., Arias, A. & Fabiano, E. Heme compounds as iron sources for nonpathogenic Rhizobium bacteria. *Journal of Bacteriology* **179**, 3076 (1997).

165 Mouriño, S., Osorio, C. R. & Lemos, M. L. Characterization of heme uptake cluster genes in the fish pathogen *Vibrio anguillarum*. *Journal of Bacteriology* **186**, 6159 (2004).

166 Perčulija, V. & Ouyang, S. in *Helicases from All Domains of Life* 141-171 (Elsevier, 2019).

167 Tutak, K. & Rozwadowska, N. Discrete roles of RNA helicases in human male germline and spermatogenesis. *Journal of Applied Genetics*, 1-5 (2020).

168 Cao, L. *et al.* The ancient function of RB-E2F pathway: insights from its evolutionary history. *Biology Direct* **5**, 1-21 (2010).

169 Ge, C. *et al.* BUD2, encoding an S-adenosylmethionine decarboxylase, is required for *Arabidopsis* growth and development. *Cell Research* **16**, 446-456 (2006).

170 Liu, C. *et al.* The protective roles of S-adenosylmethionine decarboxylase (SAMDC) gene in melon resistance to powdery mildew infection. *Horticulture,*

Environment, and Biotechnology **55**, 557-567 (2014).

171 Jauvion, V., Elmayan, T. & Vaucheret, H. The conserved RNA trafficking proteins HPR1 and TEX1 are involved in the production of endogenous and exogenous small interfering RNA in *Arabidopsis*. *The Plant Cell* **22**, 2697-2709 (2010).

172 Pan, H., Liu, S. & Tang, D. The THO/TREX complex functions in disease resistance in *Arabidopsis*. *Plant Signaling & Behavior* **7**, 422-424 (2012).

173 Sircar, S. & Parekh, N. Functional characterization of drought-responsive modules and genes in *Oryza sativa*: a network-based approach. *Frontiers in Genetics* **6**, 256 (2015).

174 Wahl, V., Brand, L. H., Guo, Y.-L. & Schmid, M. The FANTASTIC FOUR proteins influence shoot meristem size in *Arabidopsis thaliana*. *BMC Plant Biology* **10**, 1-12 (2010).

175 Werner, J. D. *et al.* FRIGIDA-independent variation in flowering time of natural *Arabidopsis thaliana* accessions. *Genetics* **170**, 1197-1207 (2005).

176 Vieira, N. G., Ferrari, I. F., de Rezende, J. C., Mayer, J. L. S. & Mondego, J. M. C. Homeologous regulation of Frigida-like genes provides insights on reproductive development and somatic embryogenesis in the allotetraploid *Coffea arabica*. *Scientific Reports* **9**, 1-15 (2019).

177 Xiong, Y., DeFraia, C., Williams, D., Zhang, X. & Mou, Z. Characterization of *Arabidopsis* 6-phosphogluconolactonase T-DNA insertion mutants reveals an essential role for the oxidative section of the plastidic pentose phosphate pathway in plant growth and development. *Plant and Cell Physiology* **50**, 1277-1291 (2009).

178 Esposito, S. Nitrogen assimilation, abiotic stress and glucose 6-phosphate dehydrogenase: The full circle of reductants. *Plants* **5**, 24 (2016).

179 Cao, S. *et al.* Genome-wide characterization of aspartic protease (AP) gene family in Populus trichocarpa and identification of the potential PtAPs involved in wood formation. *BMC Plant Biology* **19**, 1-17 (2019).

180 Simões, I. & Faro, C. Structure and function of plant aspartic proteinases. *European Journal of Biochemistry* **271**, 2067-2075 (2004).

181 Faro, C. & Gal, S. Aspartic proteinase content of the *Arabidopsis* genome. *Current Protein and Peptide Science* **6**, 493-500 (2005).

182 Shen, W. *et al.* *Arabidopsis* aspartic protease ASPG1 affects seed dormancy,

seed longevity and seed germination. *Plant and Cell Physiology* **59**, 1415-1431 (2018).

183 Chen, J. *et al.* A triallelic system of S5 is a major regulator of the reproductive barrier and compatibility of indica–japonica hybrids in rice. *Proceedings of the National Academy of Sciences* **105**, 11436-11441 (2008).

184 Yu, X. *et al.* Fertilized egg cells secrete endopeptidases to avoid polytubey. *Nature* **592**, 433-437 (2021).

185 Huang, J. *et al.* OsAP65, a rice aspartic protease, is essential for male fertility and plays a role in pollen germination and pollen tube growth. *Journal of Experimental Botany* **64**, 3351-3360 (2013).

186 Gao, H. *et al.* Two membrane-anchored aspartic proteases contribute to pollen and ovule development. *Plant Physiology* **173**, 219-239 (2017).

187 Pham, J., Stam, R., Heredia, V. M., Csukai, M. & Huitema, E. An NMRA-like protein regulates gene expression in Phytophthora capsici to drive the infection cycle on tomato. *Molecular Plant-Microbe Interactions* **31**, 665-677 (2018).

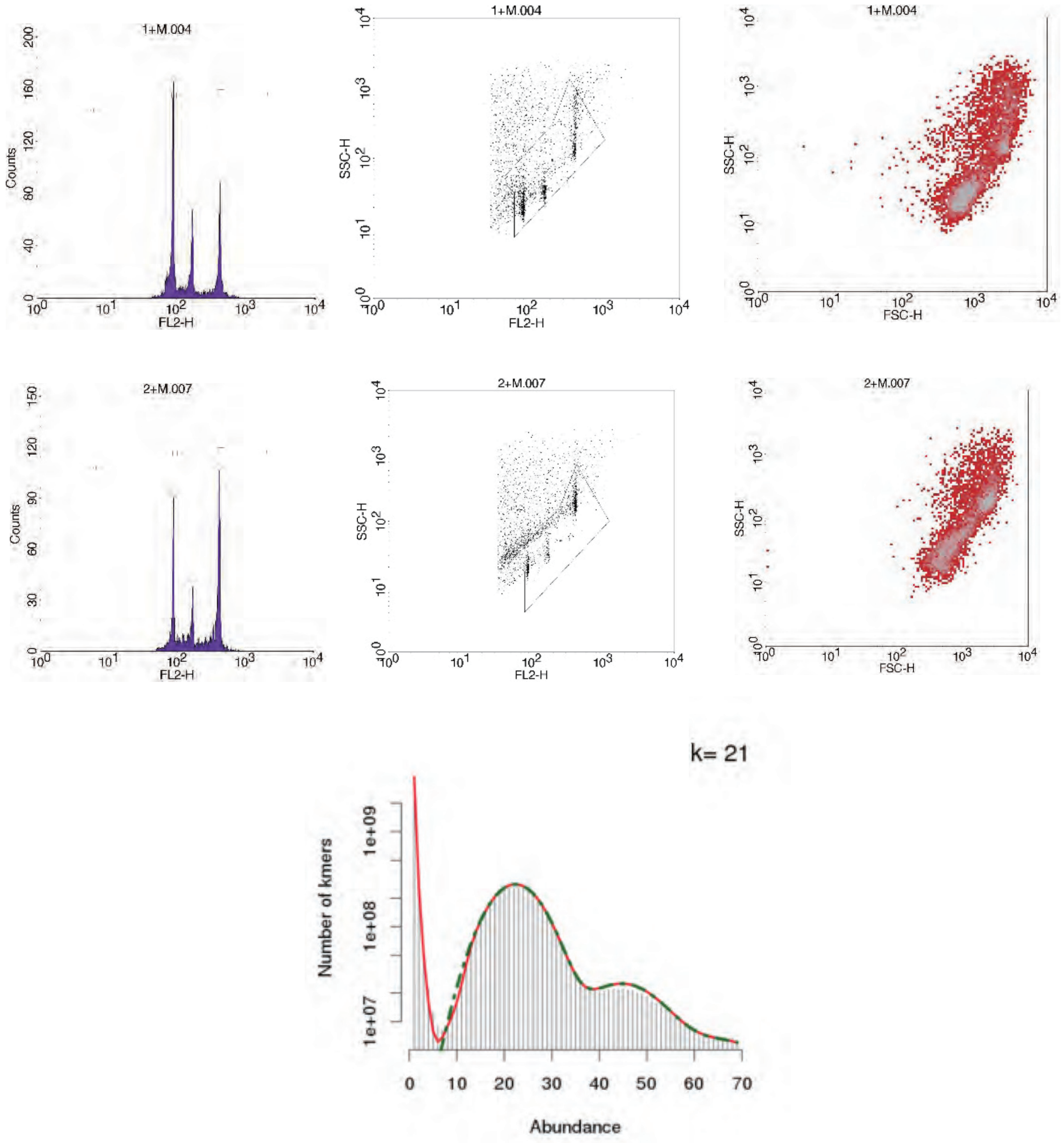

Supplementary Figure 1. Genome survey of *C. panzhihuaensis*. Genome size estimation of *C. panzhihuaensis* by flow cytometer and Kmer analysis.

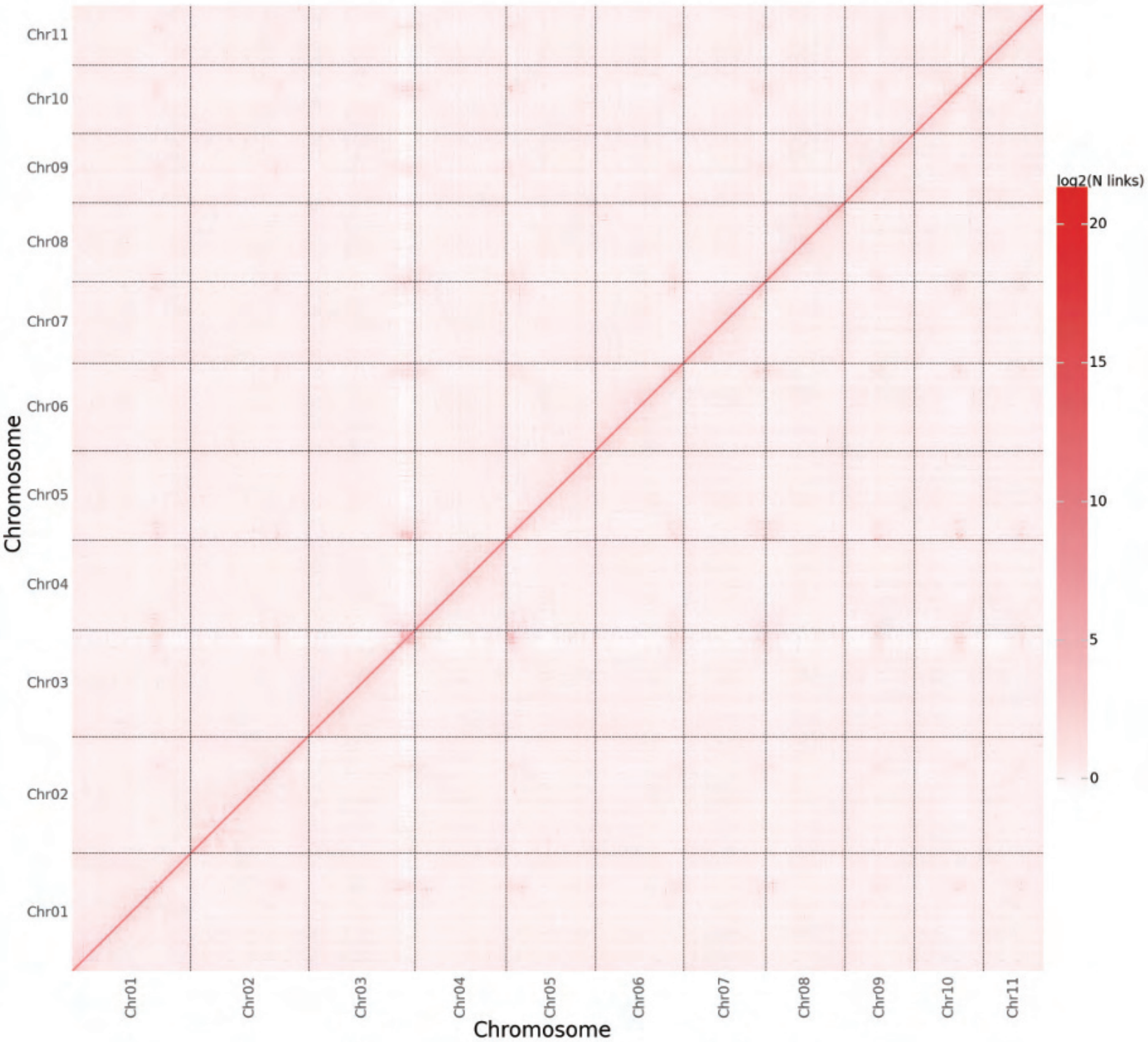

Supplementary Figure2. The Hi-C interaction heatmap of *C. panzhihuaensis*. The heatmap represents the contact matrices generated by aligning the Hi-C data to the chromosome scale assembly of the *C. panzhihuaensis* genome.

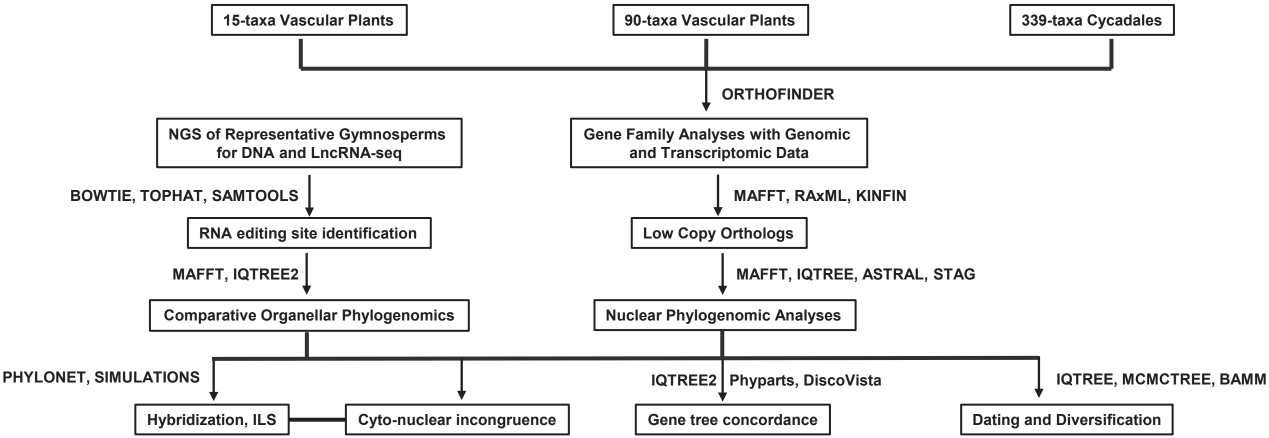

Supplementary Figure 3. The flowchart of phylogenetic reconstruction applied in current study.

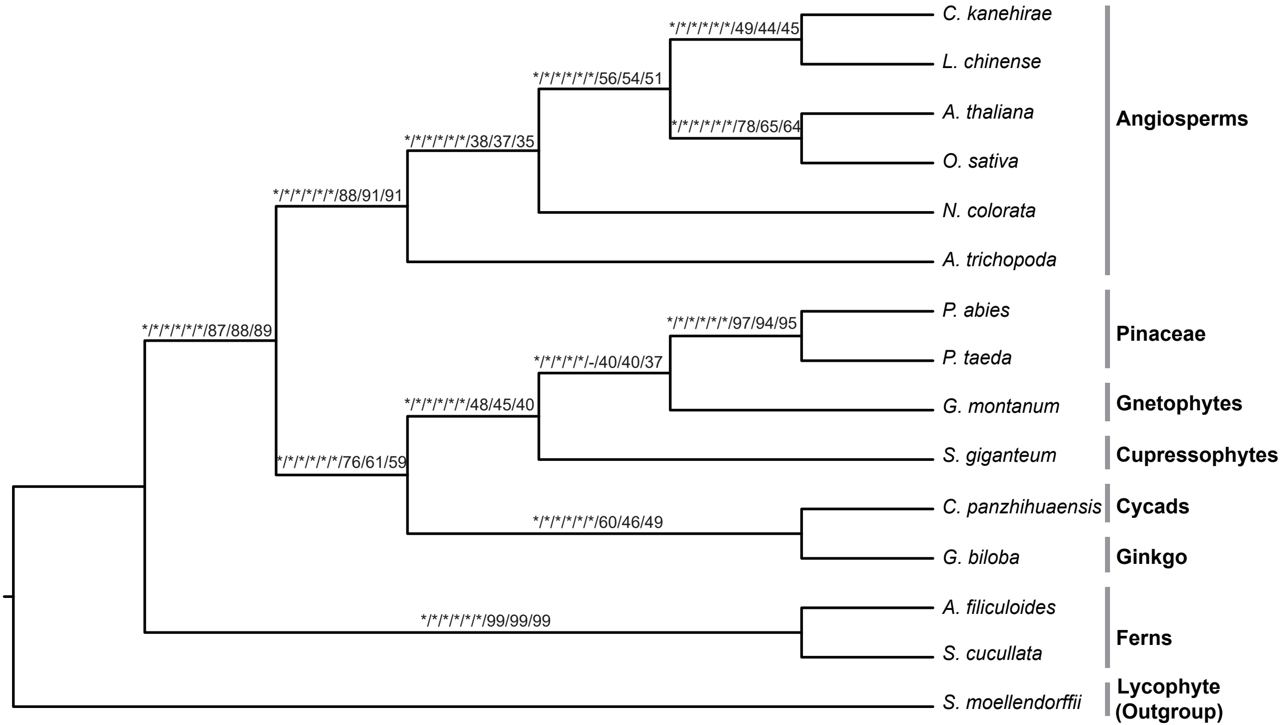

Supplementary Figure 4. Phylogenetic reconstruction of relationships among 15 vascular plants using nuclear genes. Posterior probabilities of the ASTRAL coalescent analyses for the protein-coding genes of nucleotide sequences, codon 1st + 2nd sequences, and amino acid sequences, IQTREE maximum likelihood bootstrap support values for the concatenated protein-coding genes of nucleotide sequences, codon 1st+2nd sequences, and amino acid sequences, posterior probabilities of STAG species tree inferences results of the low copy genes (1-4 copies) for the protein-coding genes of nucleotide sequences, codon 1st+2nd sequences, and amino acid sequences were indicated above the branches. Maximum support values were indicated as '*'.

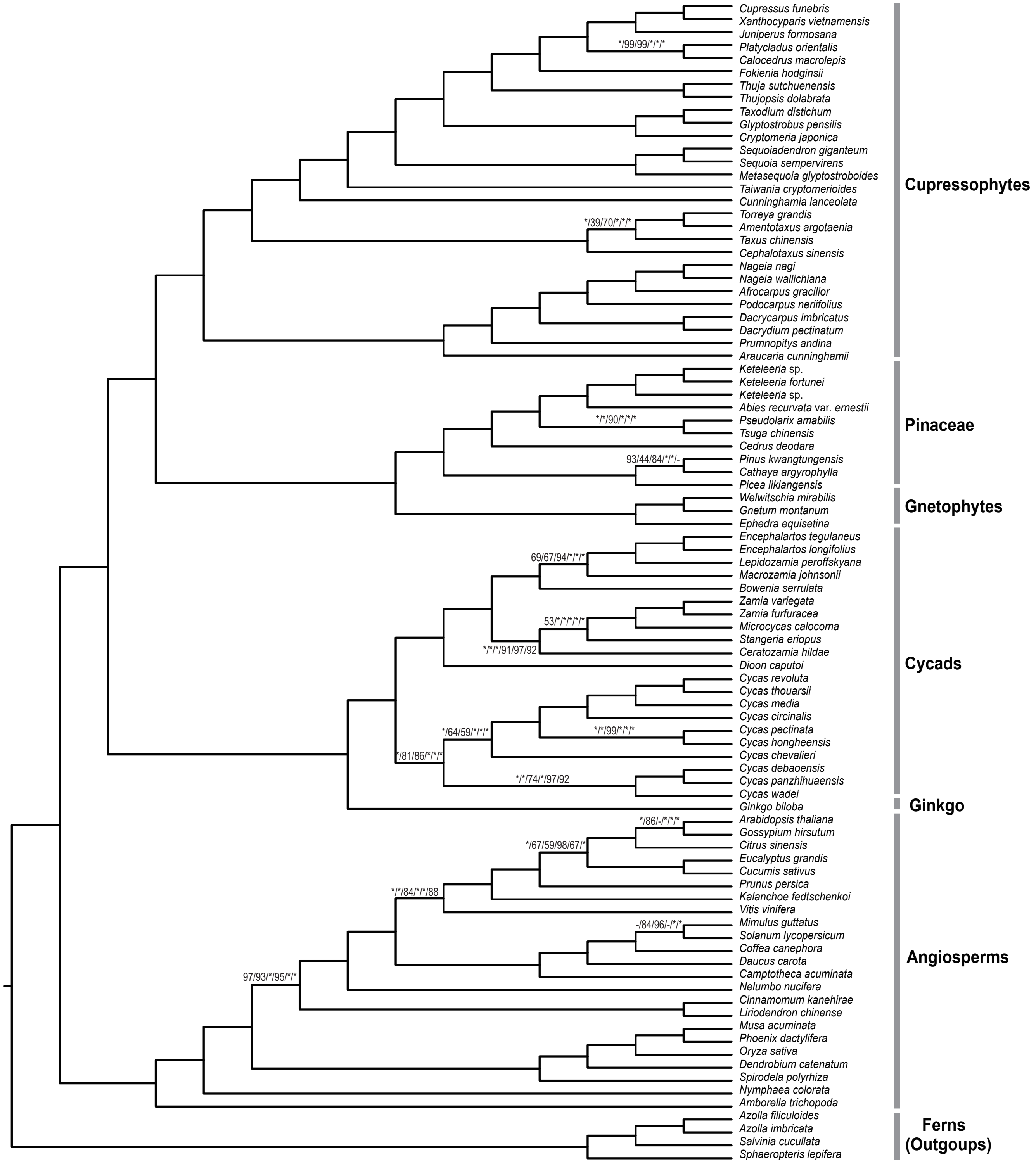

Supplementary Figure 5. Phylogenetic reconstruction of relationships among 97 vascular plants with nuclear genes. Posterior probabilities of the ASTRAL coalescent analyses for the protein-coding genes of nucleotide sequences, codon 1st+2nd sequences, and amino acid sequences. IQTREE maximum likelihood bootstrap support values for the concatenated protein-coding genes of nucleotide sequences, codon 1st+2nd sequences, and amino acid sequences, were indicated above the branches. Maximum support values by either analysis were indicated as '*'. Branches were maximally supported by all the analyses unless one word indicated.

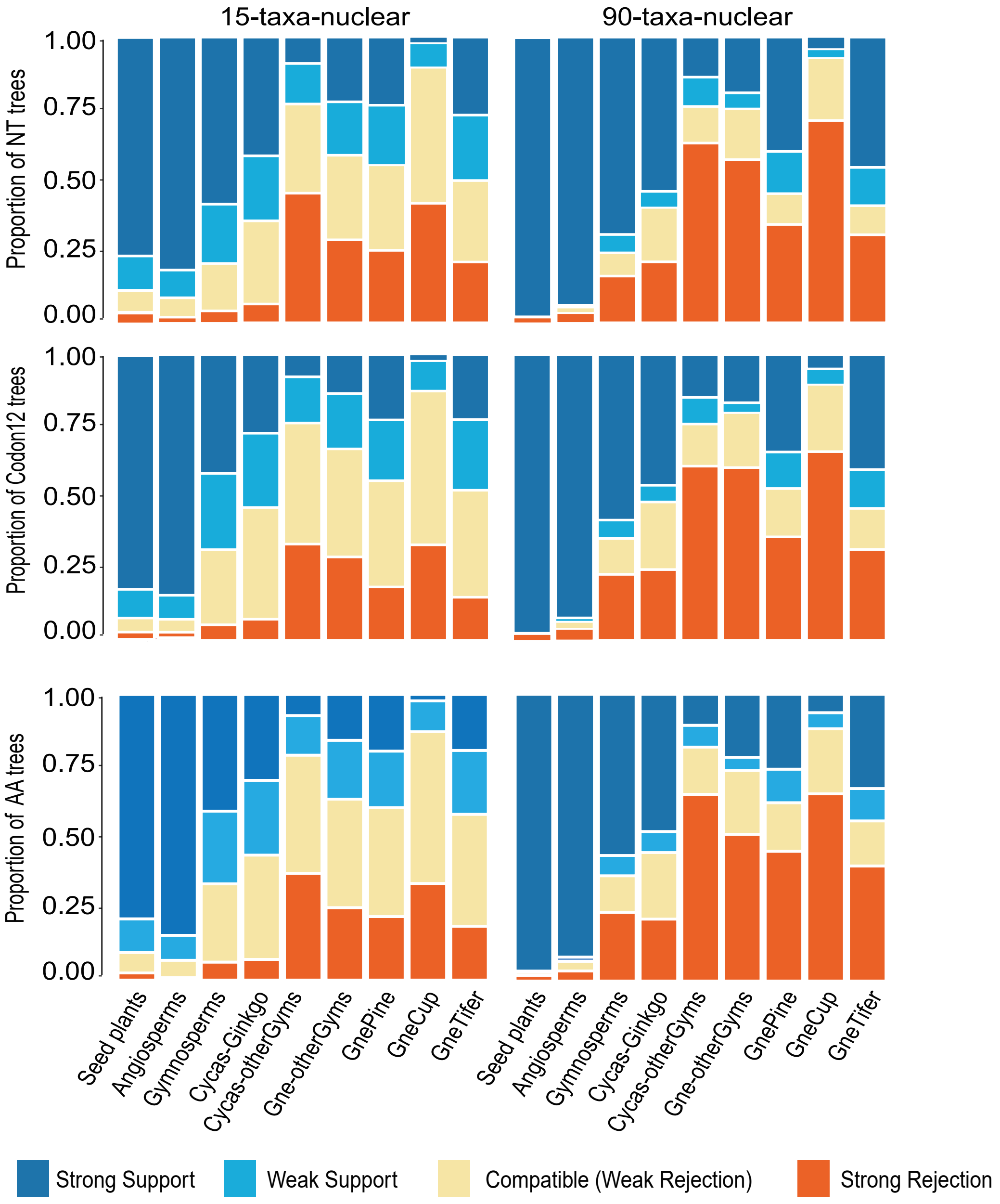

Supplementary Figure 6. Gene tree analysis of nuclear datasets. The proportion of ML gene trees for which important clades (x-axis) are highly (weakly) supported or rejected for two nuclear datasets for nucleotide dataset (upper), codon 1st+2nd positions (middle), and AA datasets (below), respectively. Weakly rejected clades correspond to clades that are not present in the tree, but are compatible if low support branches (below 75%) are collapsed.

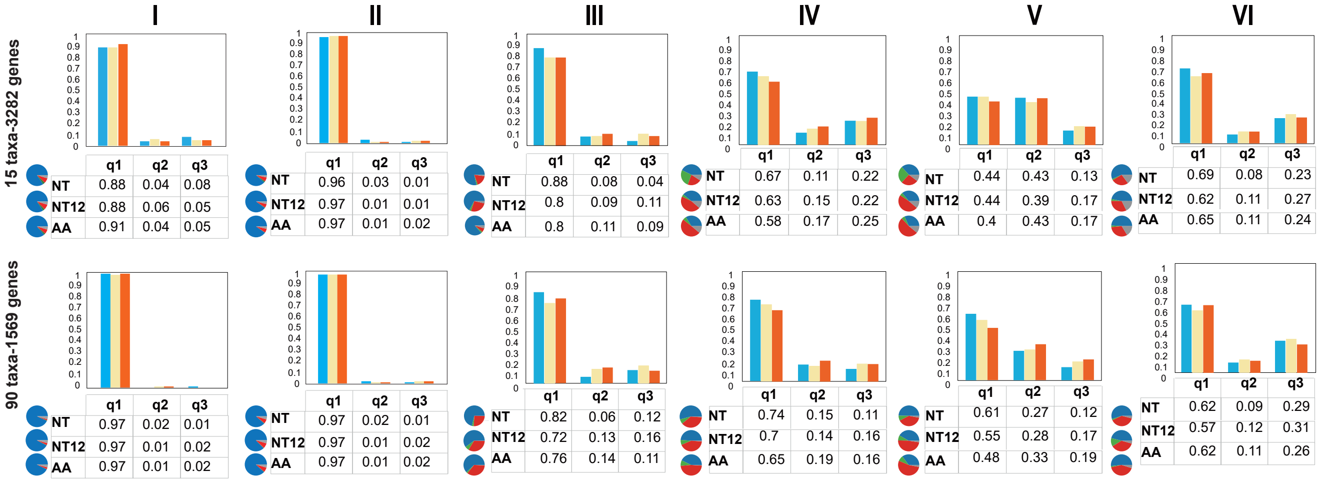

15 taxa-3282 genes

I	q1	q2	q3
NT	0.88	0.04	0.08
NT12	0.88	0.06	0.05
AA	0.91	0.04	0.05

II	q1	q2	q3
NT	0.96	0.03	0.01
NT12	0.97	0.01	0.01
AA	0.97	0.01	0.02

III	q1	q2	q3
NT	0.88	0.08	0.04
NT12	0.8	0.09	0.11
AA	0.8	0.11	0.09

IV	q1	q2	q3
NT	0.67	0.11	0.22
NT12	0.63	0.15	0.22
AA	0.58	0.17	0.25

V	q1	q2	q3
NT	0.44	0.43	0.13
NT12	0.44	0.39	0.17
AA	0.4	0.43	0.17

VI	q1	q2	q3
NT	0.69	0.08	0.23
NT12	0.62	0.11	0.27
AA	0.65	0.11	0.24

90 taxa-1569 genes

I	q1	q2	q3
NT	0.97	0.02	0.01
NT12	0.97	0.01	0.02
AA	0.97	0.01	0.02

II	q1	q2	q3
NT	0.97	0.02	0.01
NT12	0.97	0.01	0.02
AA	0.97	0.01	0.02

III	q1	q2	q3
NT	0.82	0.06	0.12
NT12	0.72	0.13	0.16
AA	0.76	0.14	0.11

IV	q1	q2	q3
NT	0.74	0.15	0.11
NT12	0.7	0.14	0.16
AA	0.65	0.19	0.16

V	q1	q2	q3
NT	0.61	0.27	0.12
NT12	0.55	0.28	0.17
AA	0.48	0.33	0.19

VI	q1	q2	q3
NT	0.62	0.09	0.29
NT12	0.57	0.12	0.31
AA	0.62	0.11	0.26

Supplementary Figure 7. Gene tree incongruence and quartet support for the six important nodes/clades (Fig. 1) for nucleotide (NT), Codon 1st+2rd (NT12), and amino acid (AA) trees for 15-taxa (upper) and 90-taxa (below) datasets, respectively. Gene tree incongruences are displayed as pie charts with blue, green, red, and gray representing concordance, top conflict, other conflict, and no signal, respectively. Quartet support is given as bar charts, with blue, yellow, and orange displaying the statistics of the quartet results inferred with ASTRAL III for NT, NT12, and AA gene trees, respectively.

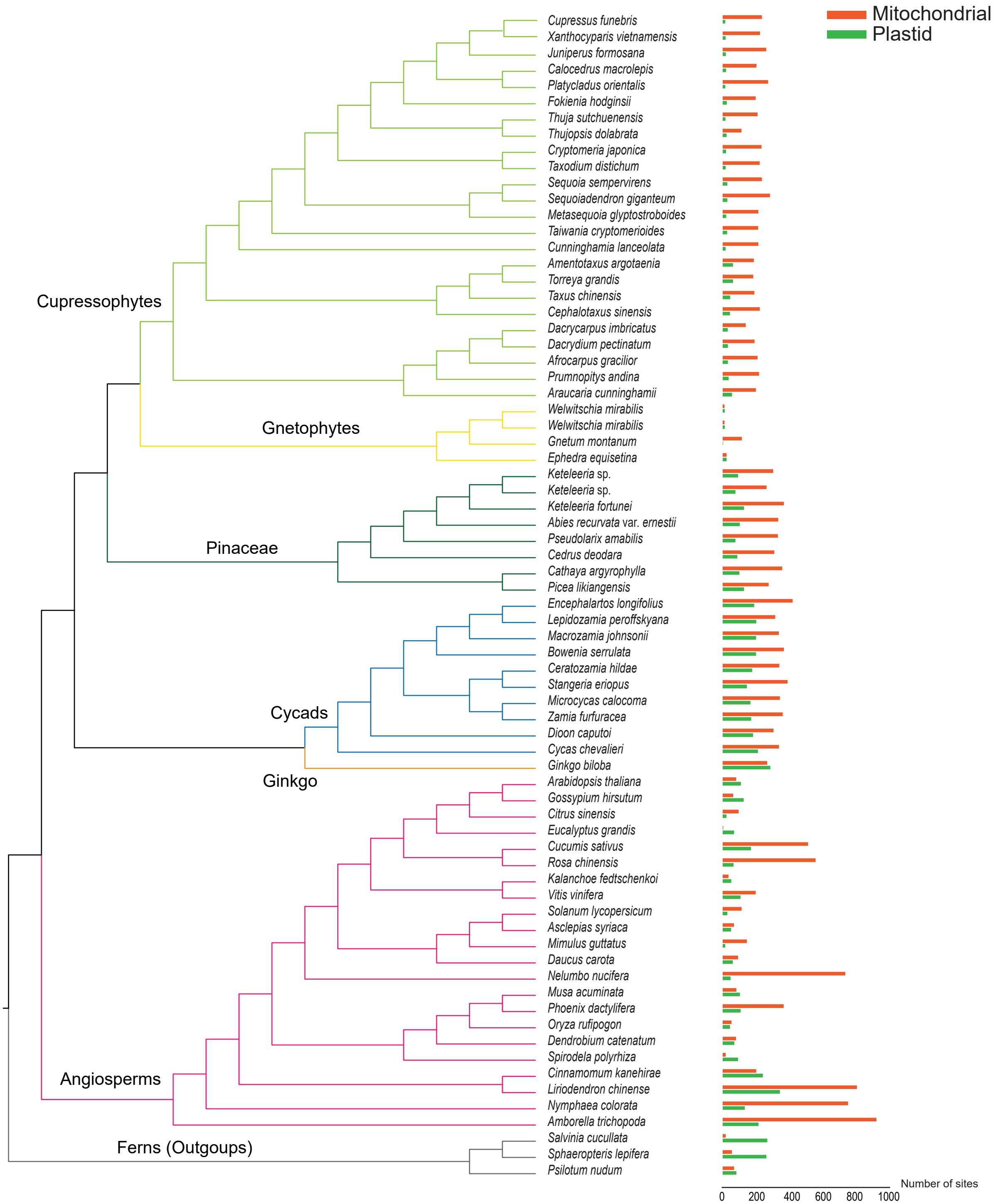

Supplementary Figure 8. RNA editing site abundance in the protein-coding genes of the organellar genomes of 72 vascular plants used in current study. The phylogeny is based on the 82 plastid protein-coding genes. As many gymnosperm mitochondrial genes were derived from the DNA resequencing data using Hybpiper, mitochondrial genes might be incomplete, hence the RNA editing sites indentified by mapping lncRNA-seq data to those incomplete genes may also be incomplete as well.

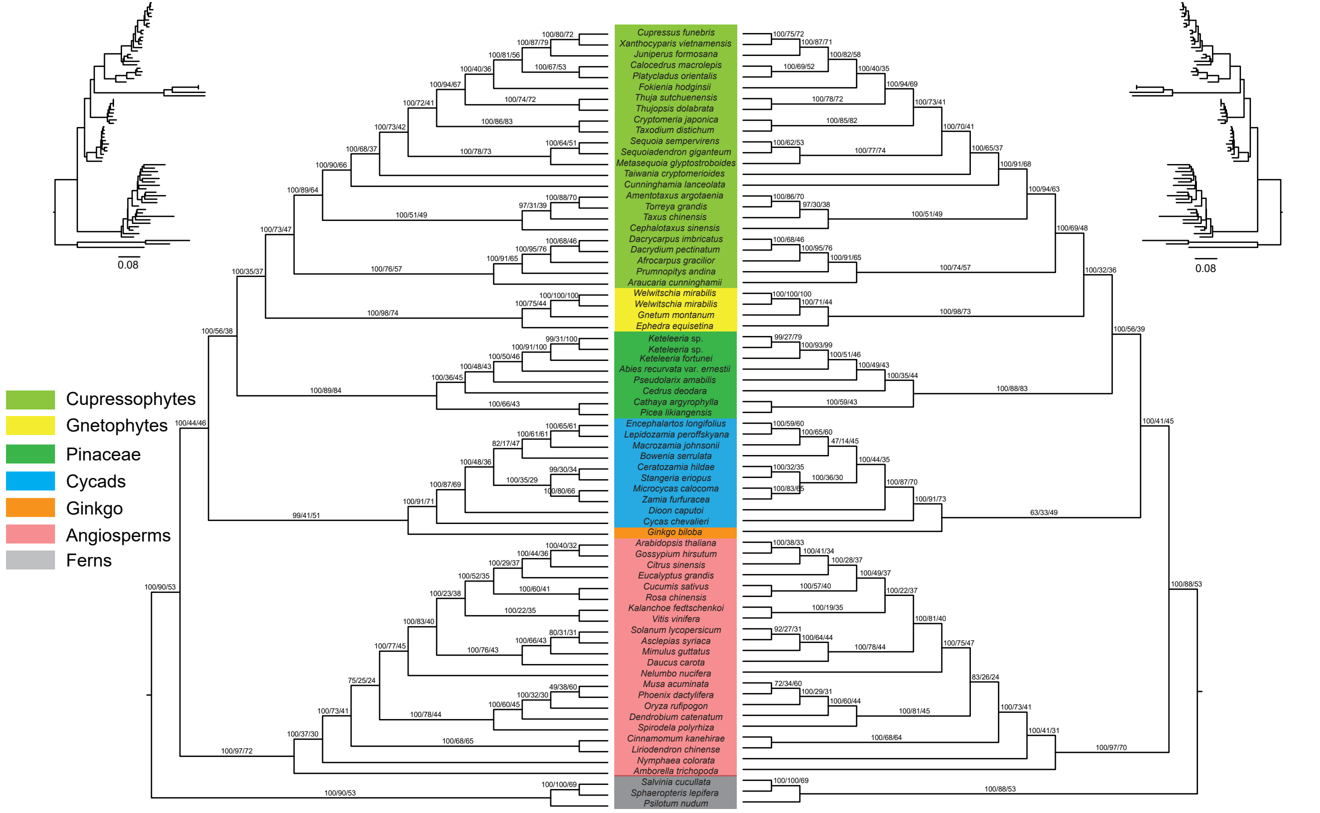

(a) Plastid tree from original protein-coding gene dataset **(b) Plastid tree from RNA editing site revised protein-coding gene dataset**

Supplementary Figure 9. Phylogenetic reconstruction of relationships among 72 vascular plants with plastid protein coding genes from orginal nucleotide sequences (a) and RNA editing site revised nucleotide sequences (b) using IQTREE2. Ultra-fast bootstrap support value/gene concordance factor/site concordance factor are all displayed on the branches.

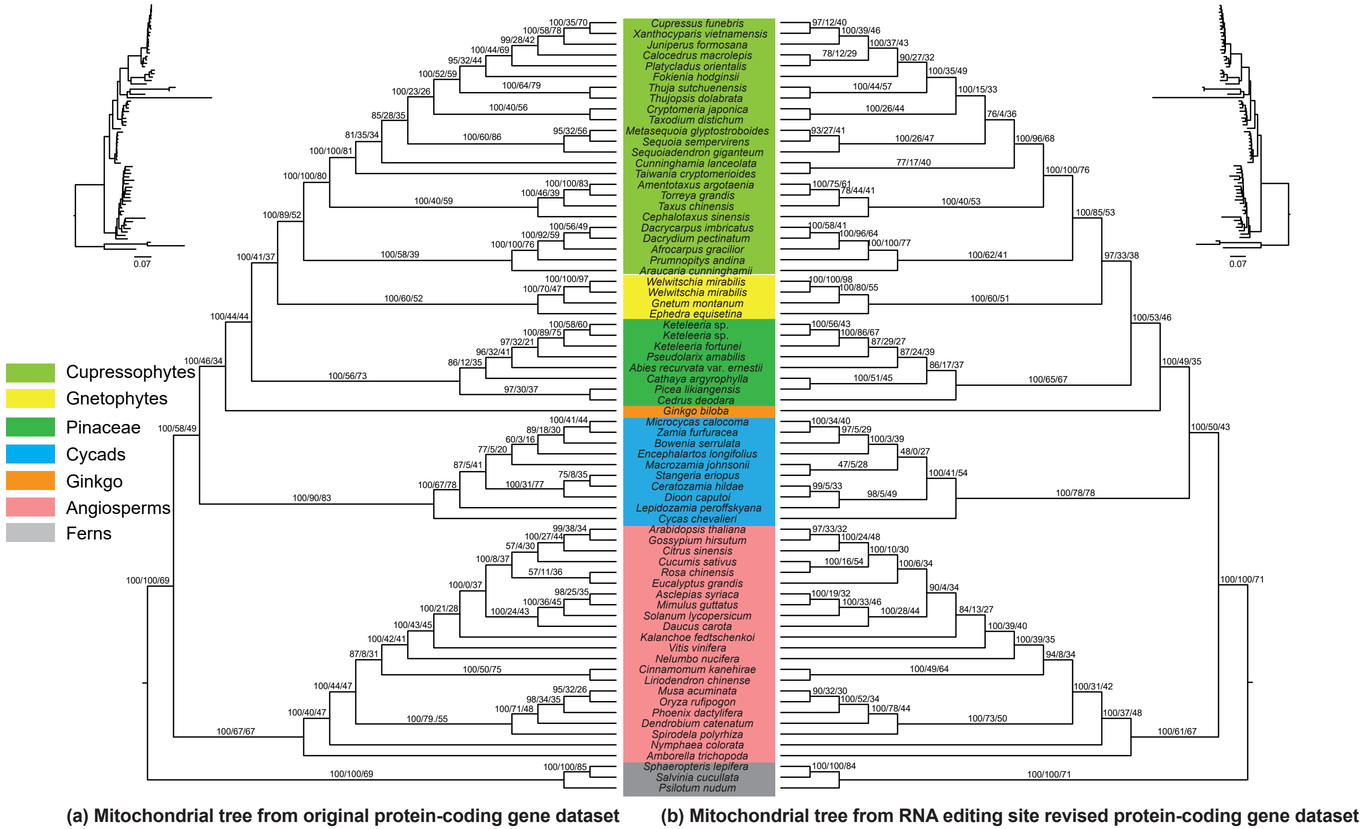

Supplementary Figure 10. Phylogenetic reconstruction of 72 vascular plants with mitochondrial protein coding genes from orginal nucleotide sequences (a) and RNA editing site revised nucleotide sequences (b) using IQTREE2. Ultra-fast bootstrap support value/gene concordance factor/site concordance factor is displayed above the branches.

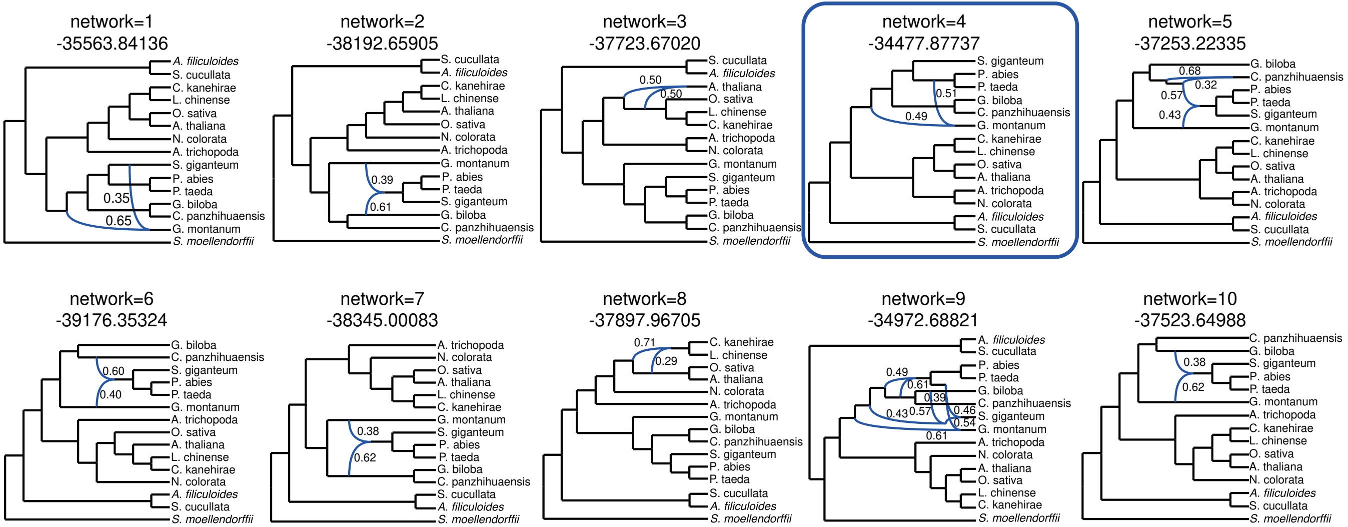

Supplementary Figure 11. PhyloNet results inferred with InferNetwork_MPL method based on 3,282 nuclear gene trees that have at least 85% taxa occupancy in all the 15 accessions.

(a)

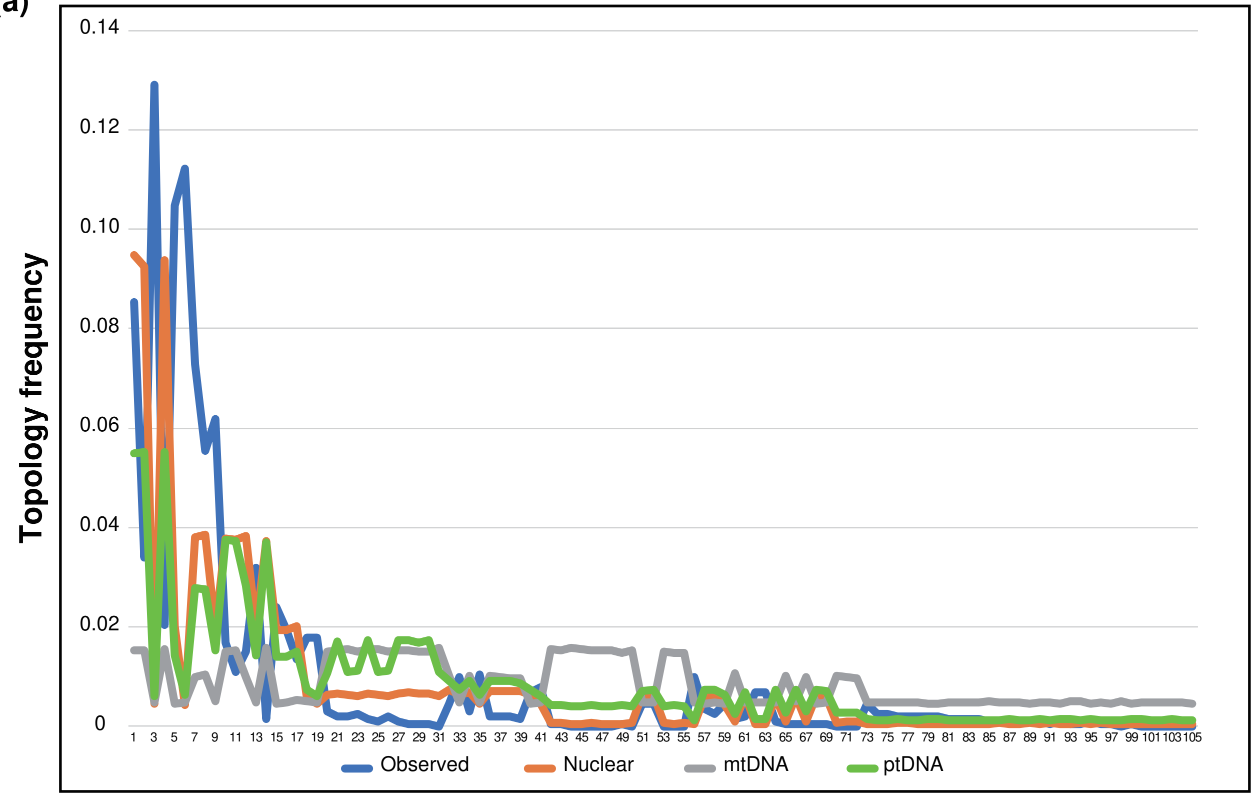

(b)

	Observed	Nuclear	mtDNA	ptDNA
**Observed**	1	.538**	-0.181	.461**
**Nuclear**	.538**	1	.415**	.894**
**mtDNA**	-0.181	.415**	1	.522**
**ptDNA**	.461**	.894**	.522**	1

Supplementary Figure 12. The distribution of the observed and simulated gene trees. (a) The topological frequencies of the observed nuclear gene trees, simulated nuclear gene trees, simulated mtDNA trees, and simulated ptDNA trees. (b) The pearson coefficient result of the topology frequencies in the above mentioned four data categories. The correlation test was performed with SPASS with 1,000 bootstrap replicates. ** indicate signicant at 0.01 level.

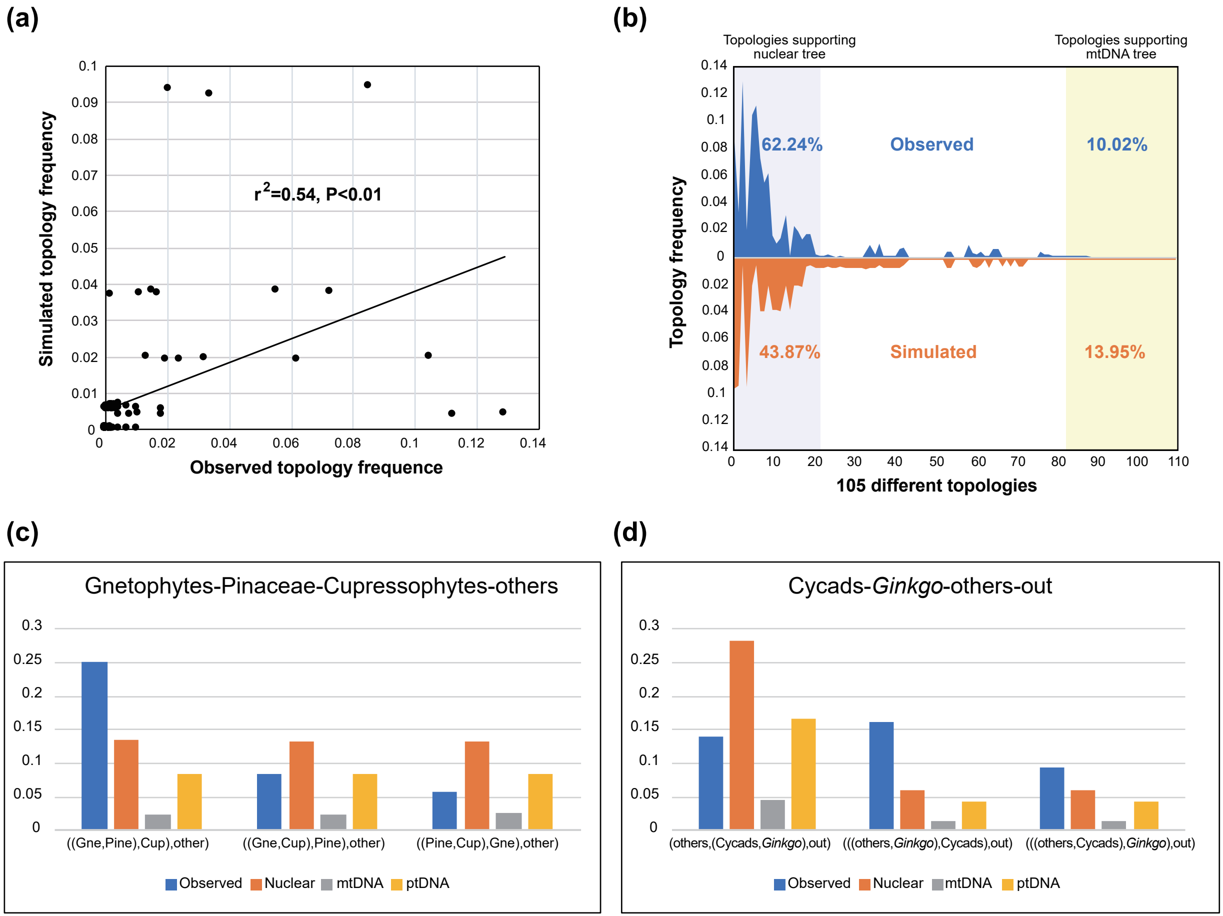

Supplementary Figure 13. The distribution of the observed and simulated nucler trees. (a) Comparison of the simulated frequencies based on coalescent probabilities of gene tree topologies of different classes. The correlation is significant at a confidence level of 0.01 (Two-sided hypothesis test). (b) Corresponding frequencies of observed gene trees and simulated gene trees showing essentially the same results as with *Arabidopsis* as the outgroup. (c) and (d) The topology frequency estimated by Twisst software of each group. Gne represents Gnetophytes, Pine represents Pinaceae, Cup represents Cupressophytes, other represents other gymnosperms, out represents outgroup *Arabidopsis.*

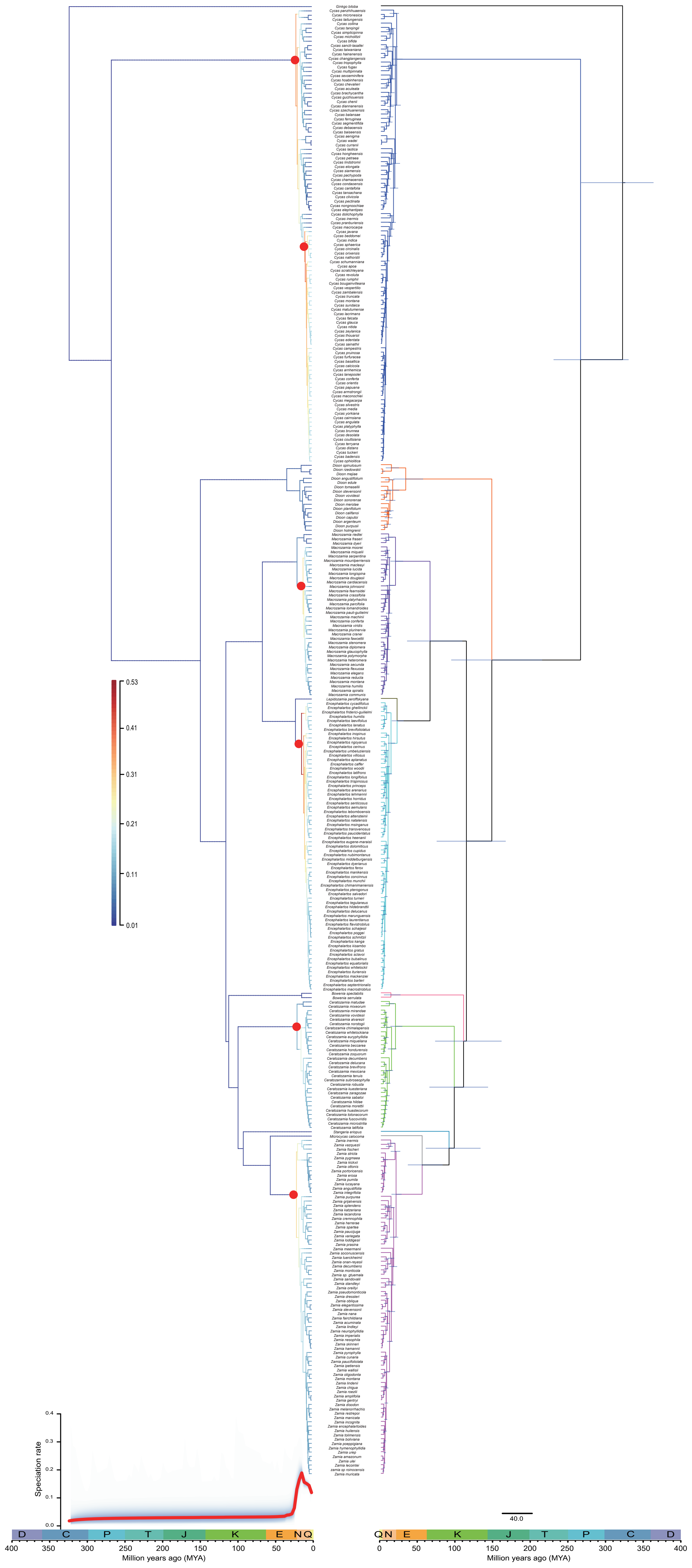

Supplementary Figure 14. The chronogram of 339 cycad species inferred with MCMCTree (left panel) based on 100 single-copy nuclear genes with concordant evolutionary histories. The diversification pattern was inferred with BAMM (right panel).

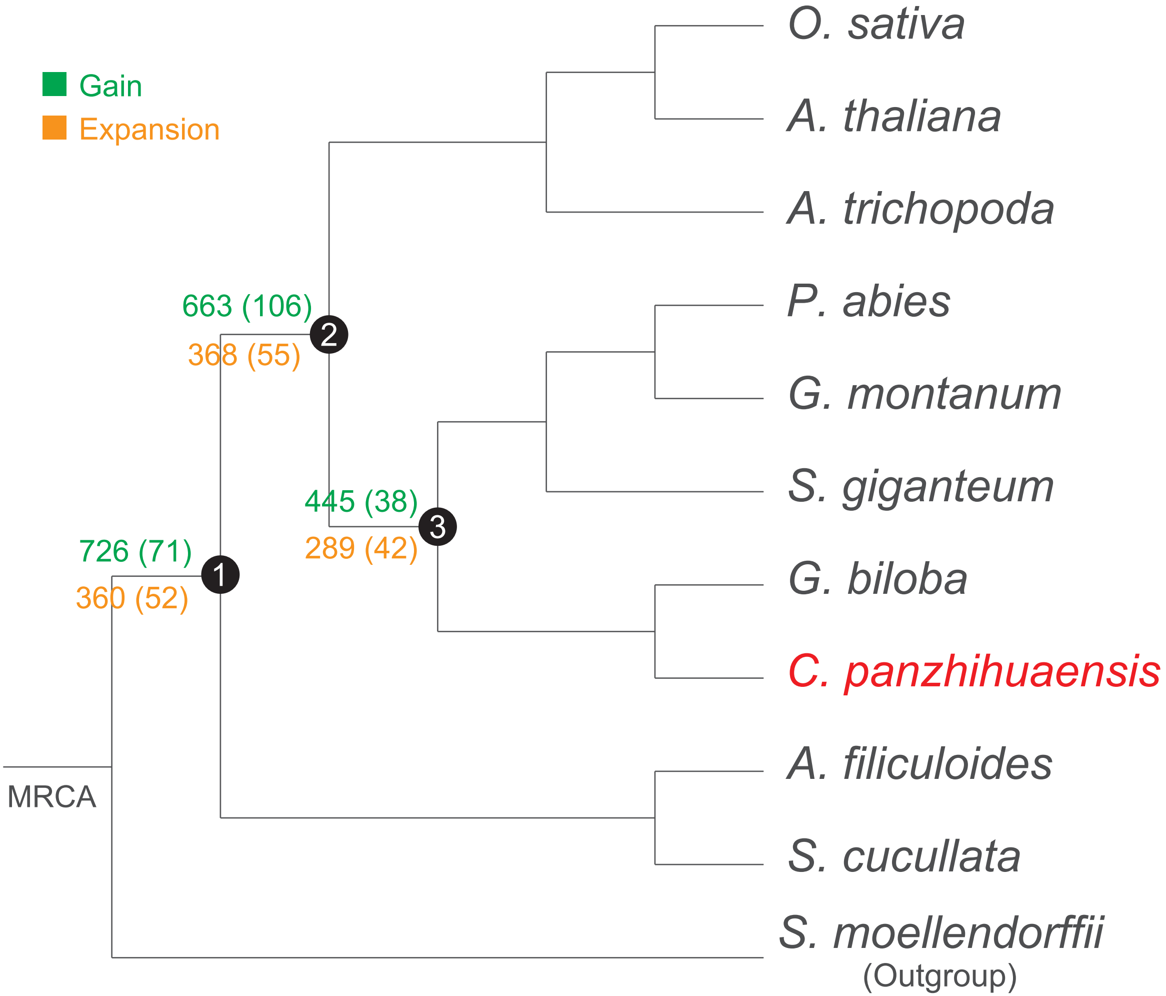

Supplementary Figure 15. The evolution of seed related genes. The number outside of the bracket shows gain and expansion of Orthogroups. The number in the bracket indicates the seed related Orthogroups. The tree of fig. 2b was used for this analysis.

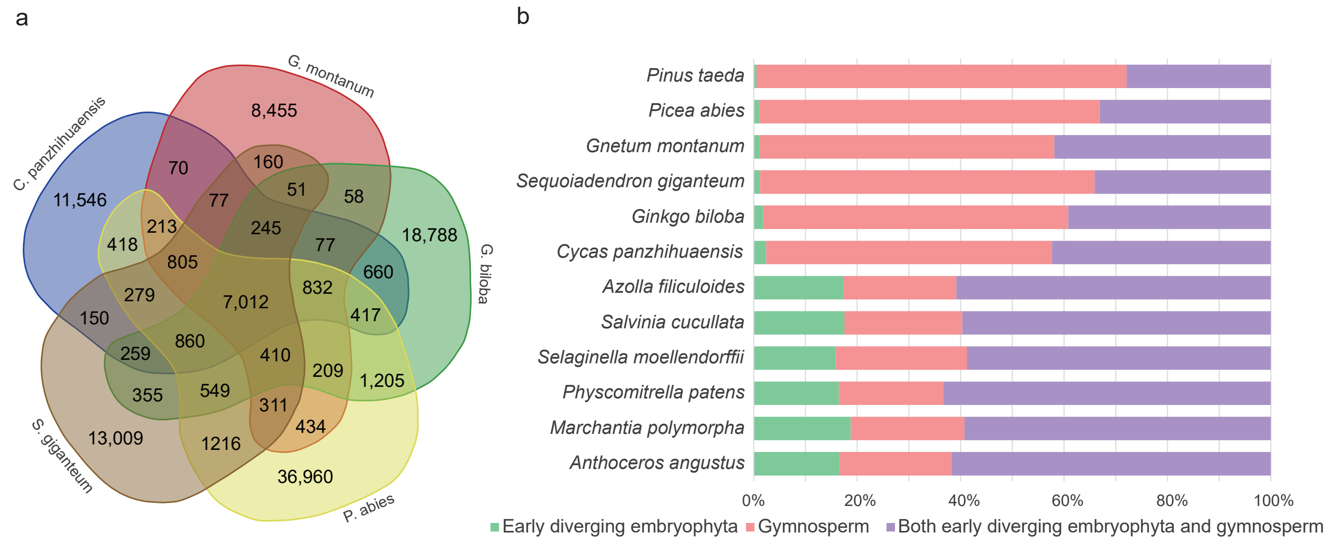

Supplementary Figure 16. Comparative genomics of *C. panzhihuaensis* and other gymnosperms. (a) Venn diagram showing unique and shared orthogroups among five representative gymnosperms. The KEGG enrichment of unique orthogroups of *C. panzhihuaensis* is summarized in Table S18. (b) The percentage of total proteins shared in both early-diverging embryophytes and gymnosperm (light purple), proteins shared among early-diverging embryophytes (light green), and proteins shared among embryophytes (pink).

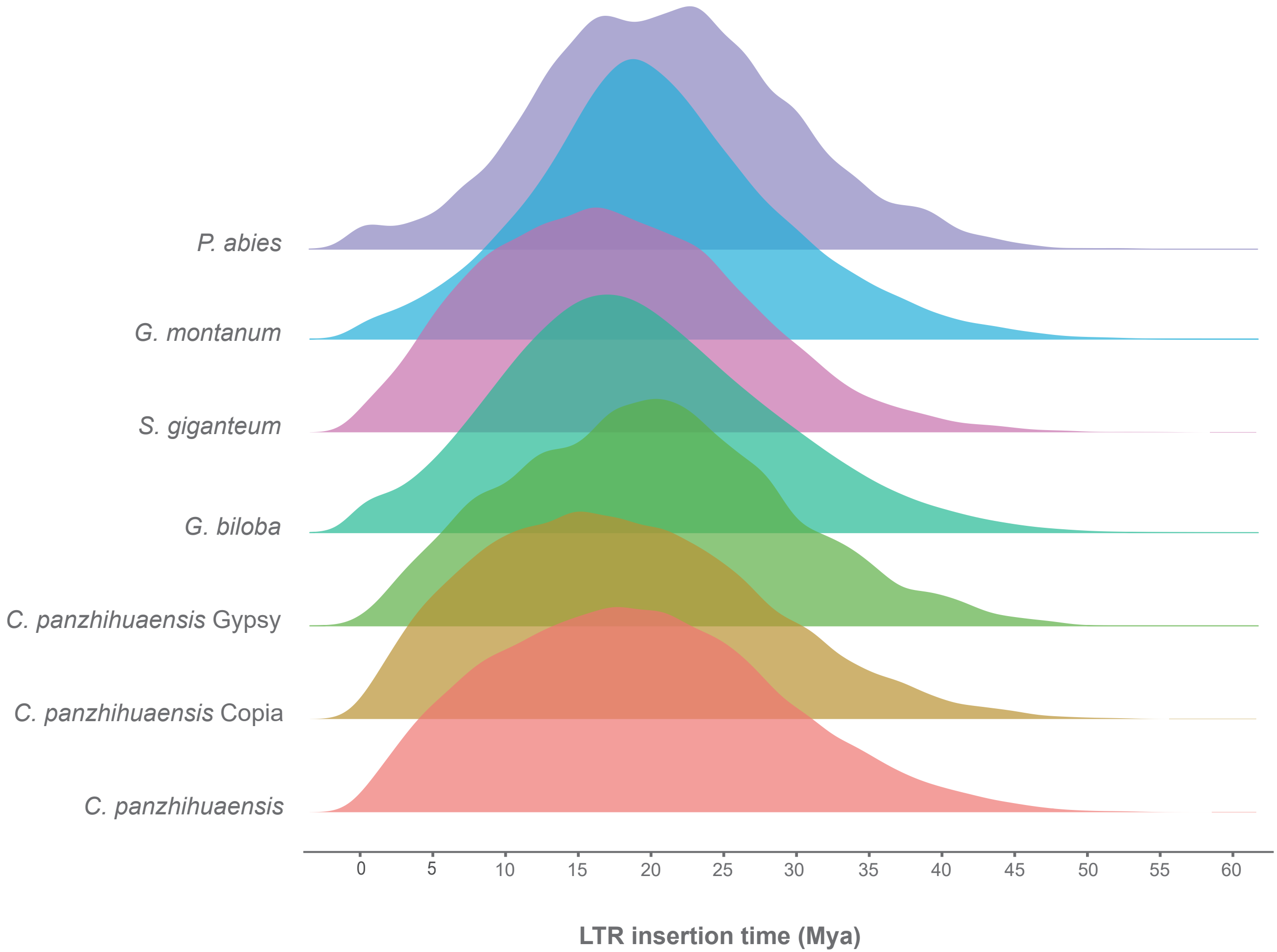

Supplementary Figure 17. Comparison of insertion dates of LTR-RTs among gymnosperms. Insertion time and copy number of Copia and Gypsy LTR retrotransposons of *C. panzhihuaensis*, and the comparison of the insertion time of all LTRs among gymnosperms.

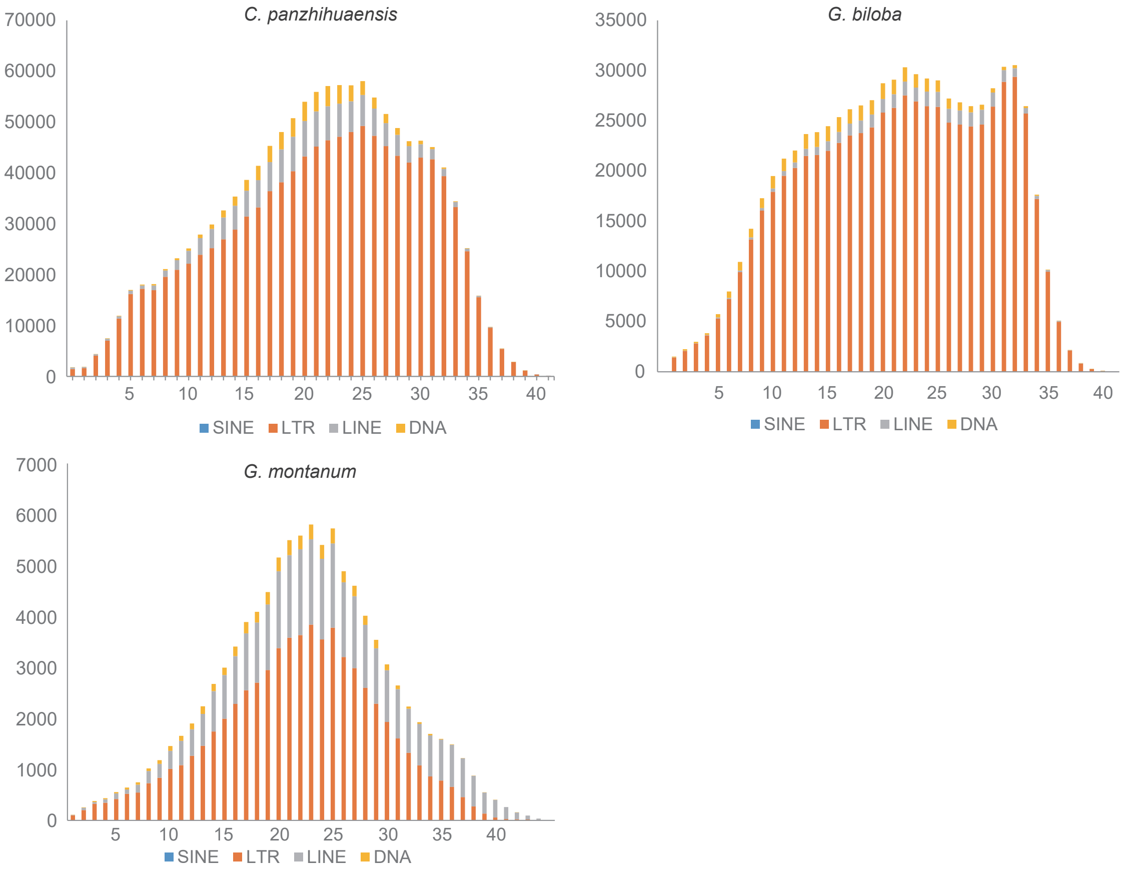

Supplementary Figure 18. Evolutionary history of TE in the introns of *C. panzhihuaensis* and other gymnosperms. Distribution of sequence divergence for main types of TEs in introns of *C. panzhihuaensis*, *G. biloba* and *G. montanum*. Divergence rate stands for the percentages of substitutions in matching regions compared to the consensus sequences to classify TEs generated by RepeatMasker.

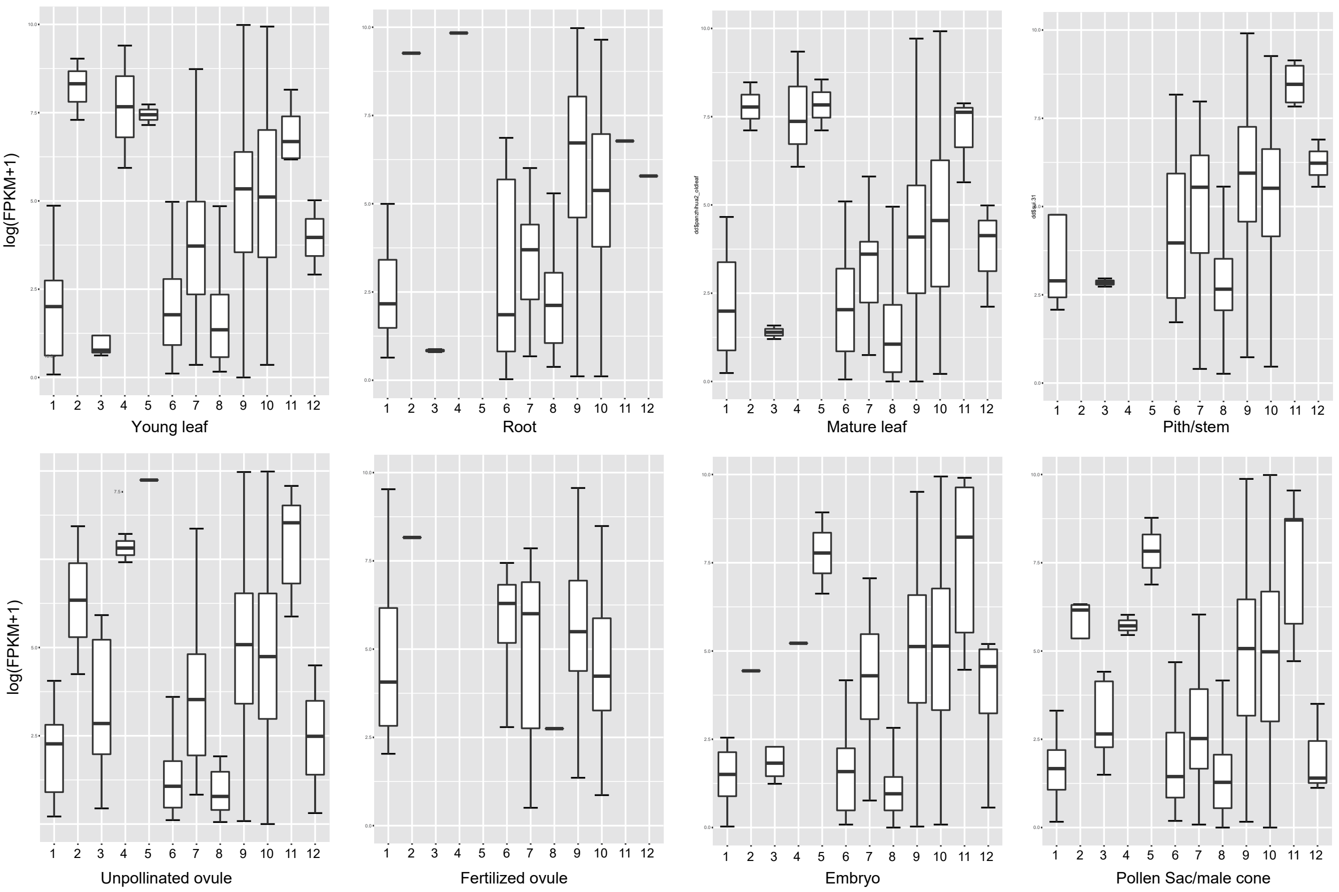

Supplementary Figure 19. Comparison of expression levels of intact various repeat elements in different tissues of *C. panzhihuaensis.* 1, DNA 2, DNA/CMC-EnSpm. 3, DNA/hAT. 4, DNA/MULE-MuDR. 5, DNA/PIF-Harbinger. 6,LINE. 7,LINE/L1. 8, LTR. 9, LTR/Copia. 10, LTR/Gypsy. 11, RC/Helitron. 12, SINE. The minimum, first quartile (Q1), median, third quartile (Q3), and maximum value was indicated in the box-plot by order after excluding the outliers. Only expressed repeat sequences were selected from the 12 type of various repeat elements to draw the boxplot.

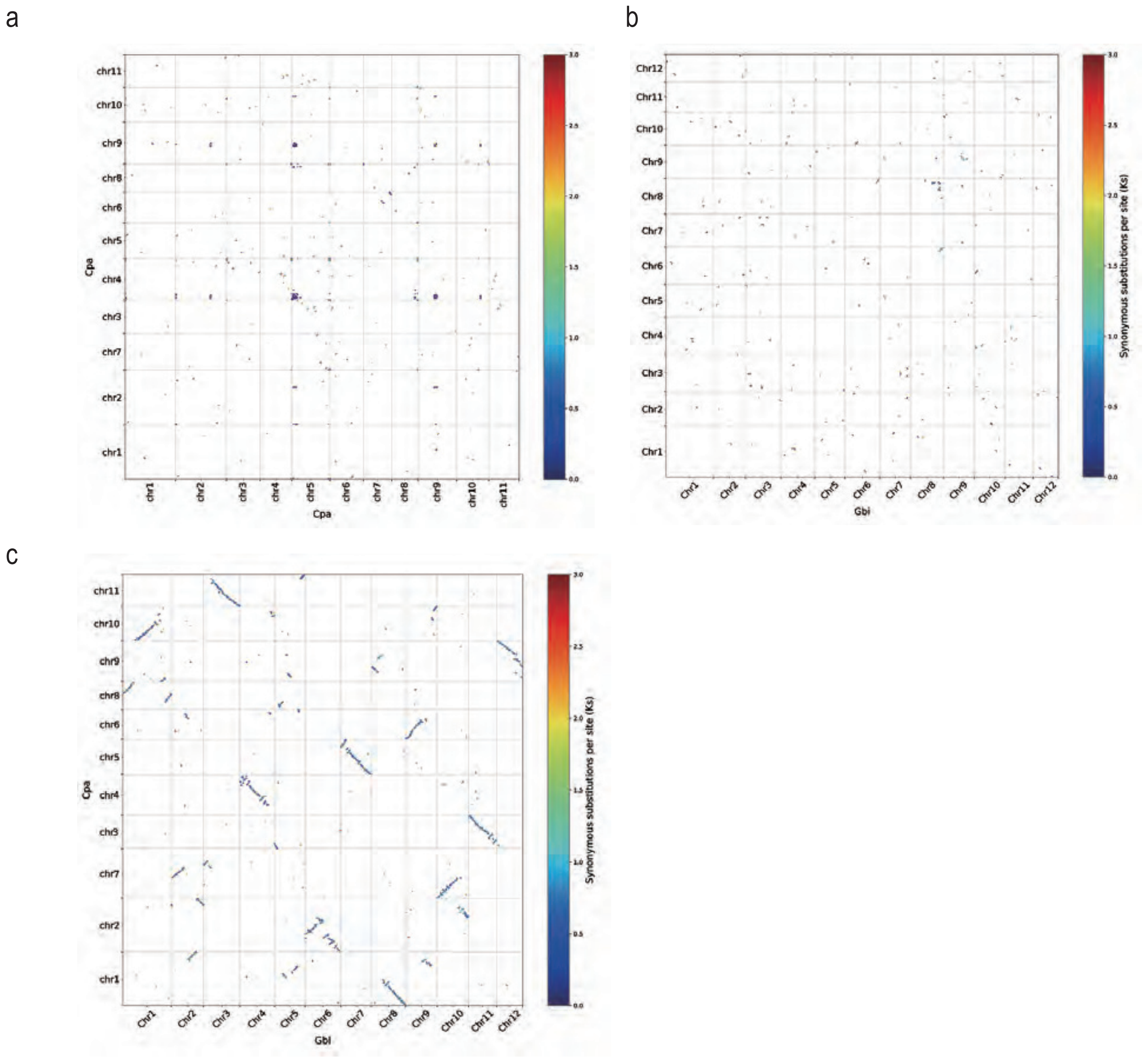

Supplementary Figure 20. The dot plots of paralogous blocks. (a) Dotplot of paralogs in the *C. panzhihuaensis* (Cpa) genome. (b) Dotplot of paralogs in the *G. biloba* (Gbi) genome. (c) Dotplot of othologs between *G. biloba* and *C. panzhihuaensis* genome.

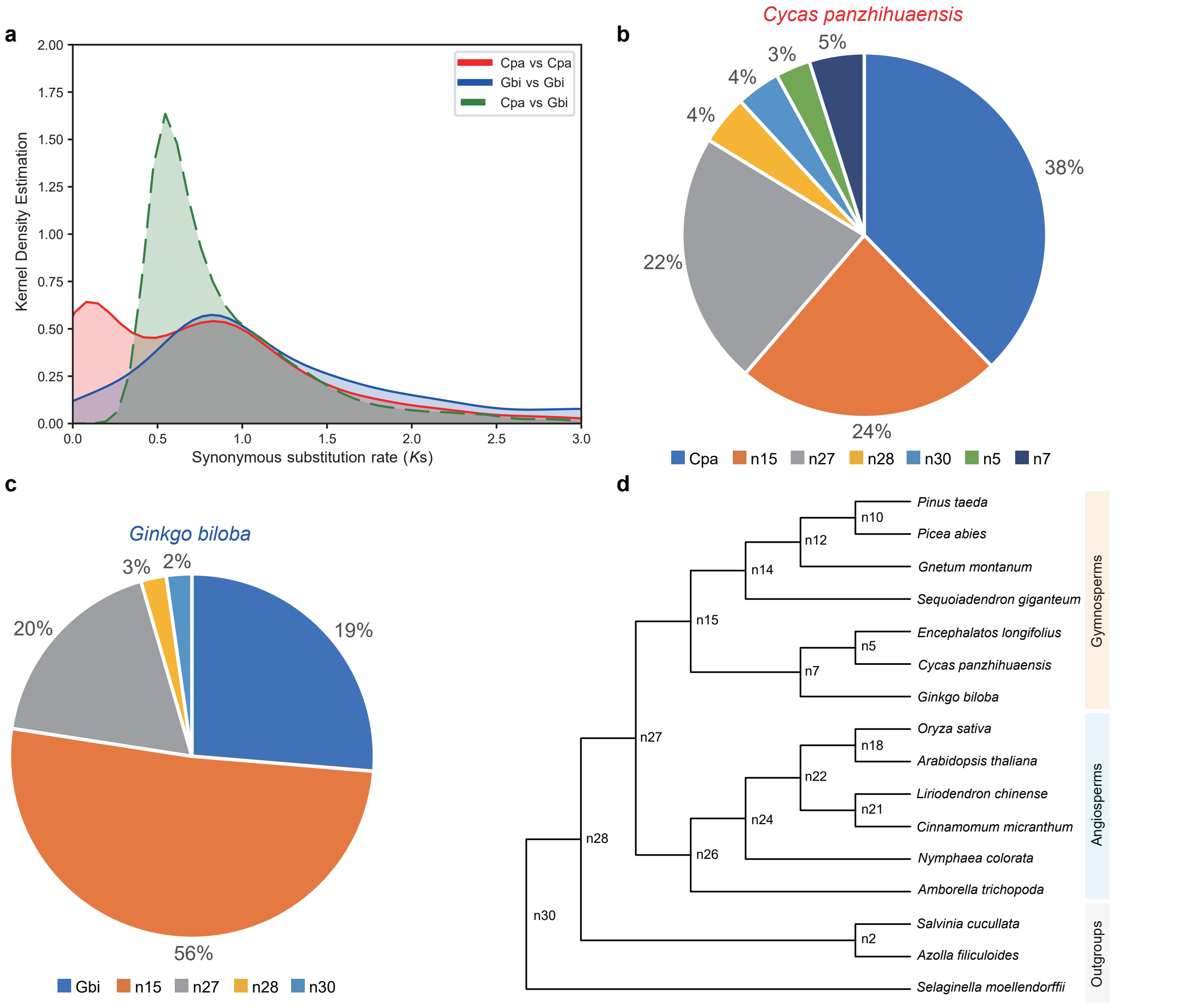

Supplementary Figure 21. Phylogenomic dating of paralogous genes generated Ks peak. (a) Distribution of synonymous substitutions per synonymous site (Ks) of syntenic orthologous (dashed curves) and paralogous genes (solid curves). Cpa: *Cycas panzhihuaensis*, Gbi: *Ginkgo biloba*. (b) The pie chart showing the duplication time of paralogous genes in *C. panzhihuaensis* genome, which generated a Ks peak ranging from 0.5-1.2, based on phylogenomic dating method. (c) The pie chart showing the duplication time of paralogous genes in *G. biloba* genome, which generated a Ks peak ranging from 0.5-1.2, based on phylogenomic dating method. (d) The phylogenetic tree with node name displayed the phylogenetic position of the duplication nodes in (b) and (c). The phylogenetic tree used here referred the tree of fig. 2a.

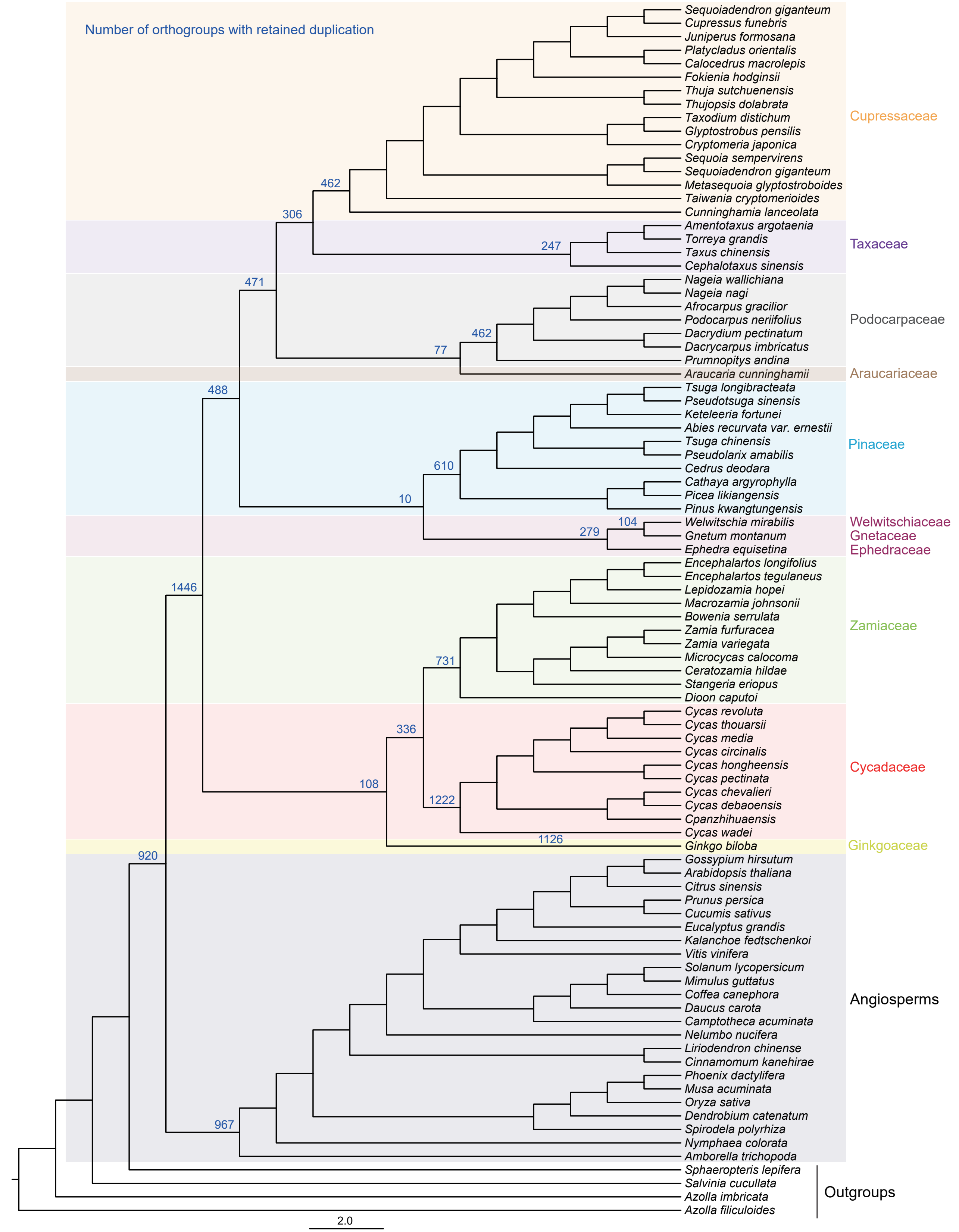

Supplementary Figure 22. Phylogenetic reconstruction of relationships among 90 plants, with a comprehensive and high-density sampling of gymnosperms in this study. The number of gene families with retained gene duplications of each node are shown above the branch across the phylogeny.

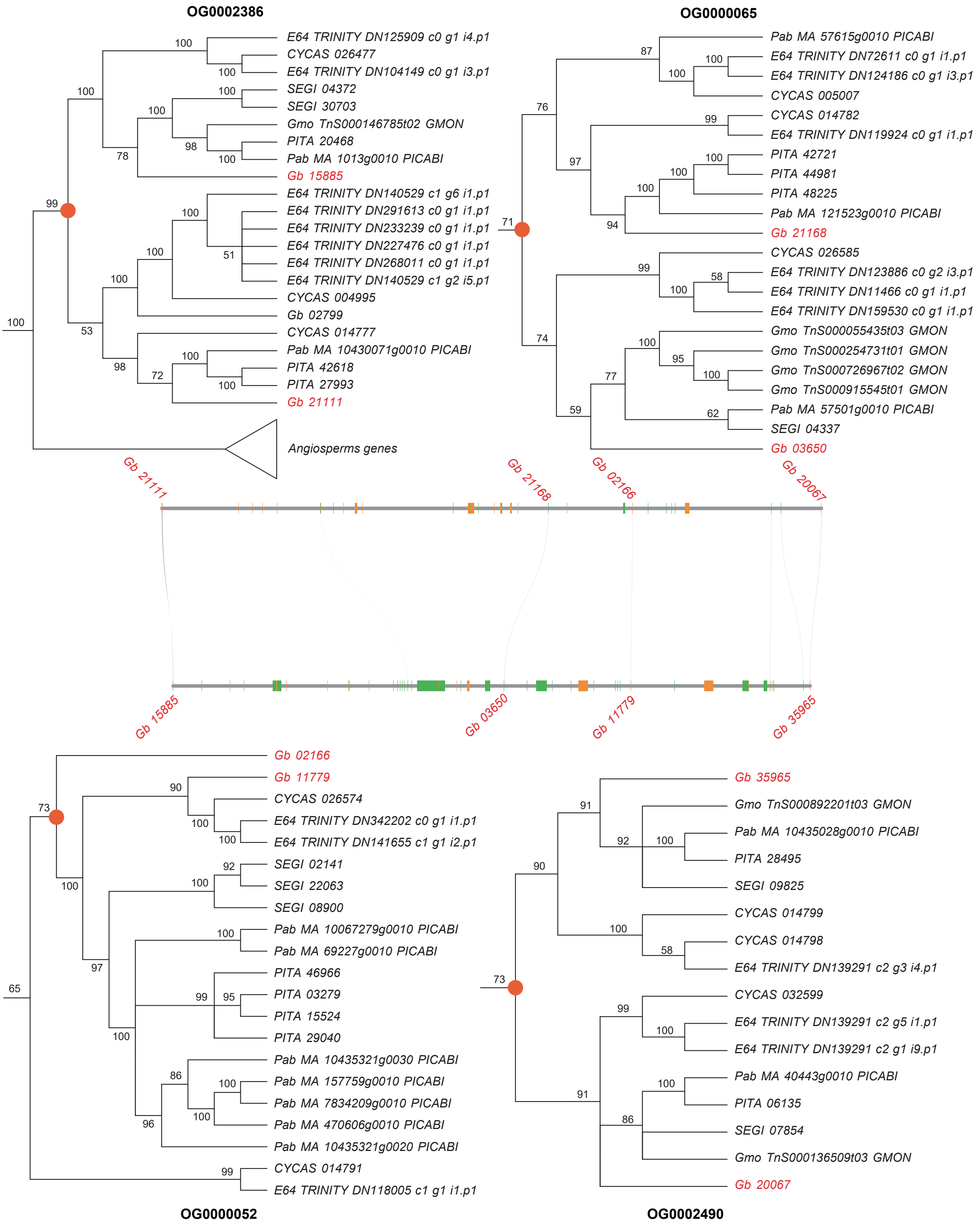

Supplementary Figure 23. Example showing both the phylogenomic and syntenic evidence supporting an ancestral polyploidy event in extant gymnosperms. Four pairs of paralogous genes in OG0002386, OG0000065, OG00000052 and OG0002490 were duplicated before the divergence of gymnosperms and after the split of angiosperms and gymnosperms based on phylogenetic trees. These pairs of duplicate genes are located on the same syntenic block identified in *G. biloba* genome.

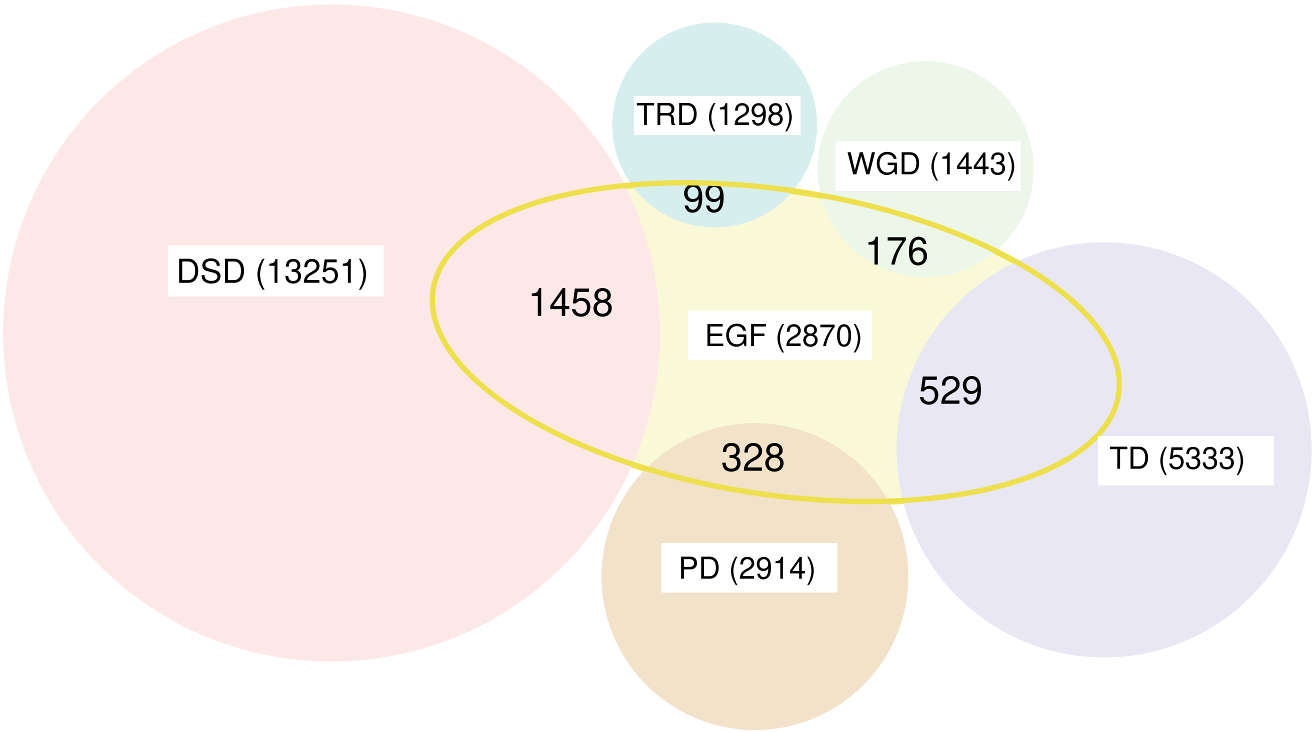

Supplementary Figure 24. Venn diagram shows the possible logical relations between members of expanded gene family and modes of duplication (WGD, TD, PD, TRD, and DSD). TD: tandem duplication, PD: proximal duplication, WGD: whole genome duplication, TRD: transposed duplication, DSD: dispersed duplication, EGFs: expanded gene families.

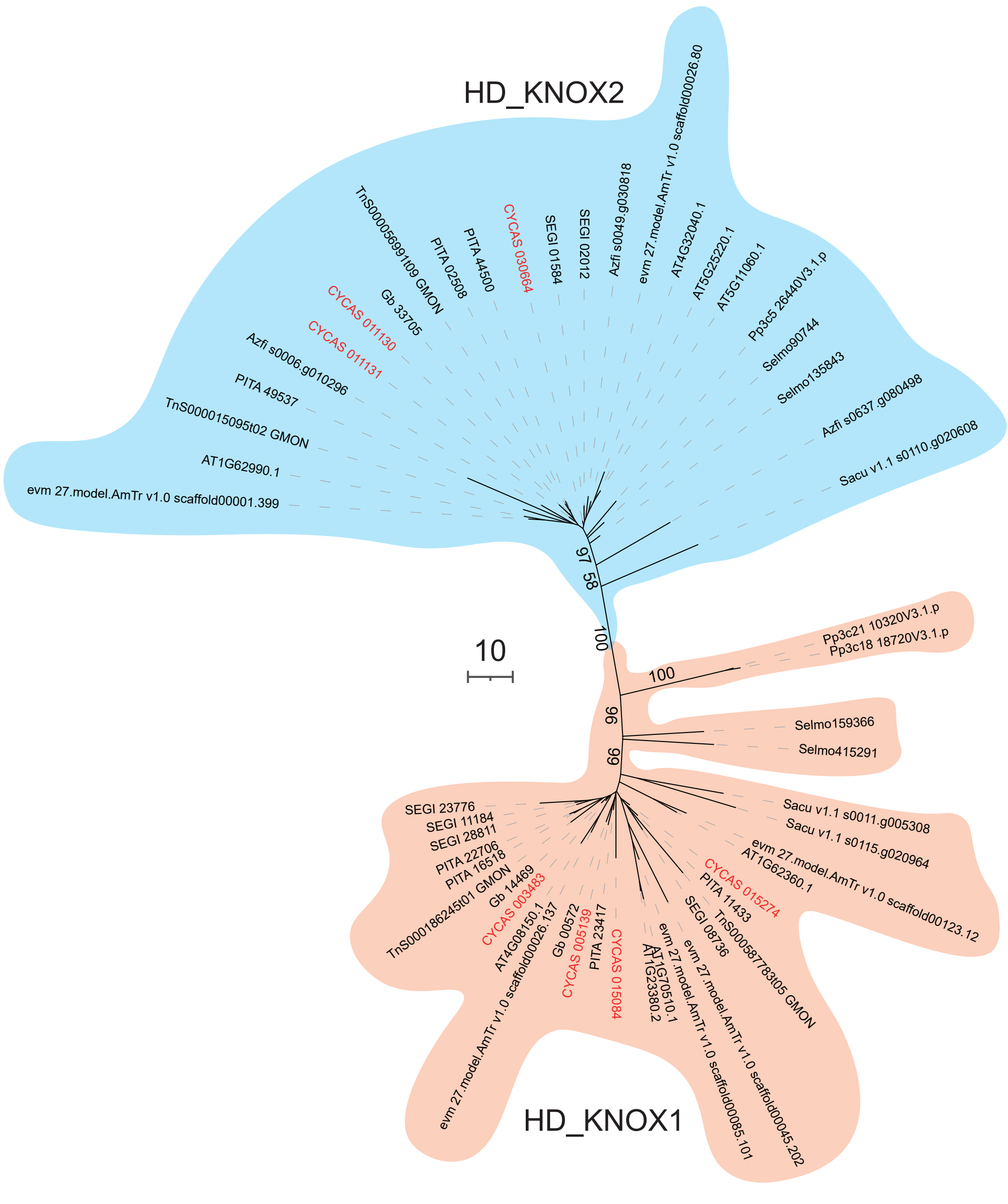

Supplementary Figure 25. Maximum-likelihood phylogenetic tree with bootstrap support of replicates of the HD_KNOX1 and HD_KNOX2. The tree of antifungal protein ginkbilobin was built by RAxML (estimating branch support values by bootstrap iterations with 500 replicates) with PROTGAMMAGTRX amino acid substitution mode. Scale bar indicates branch length and bootstrap values ≥ 50% are shown on the central branches.

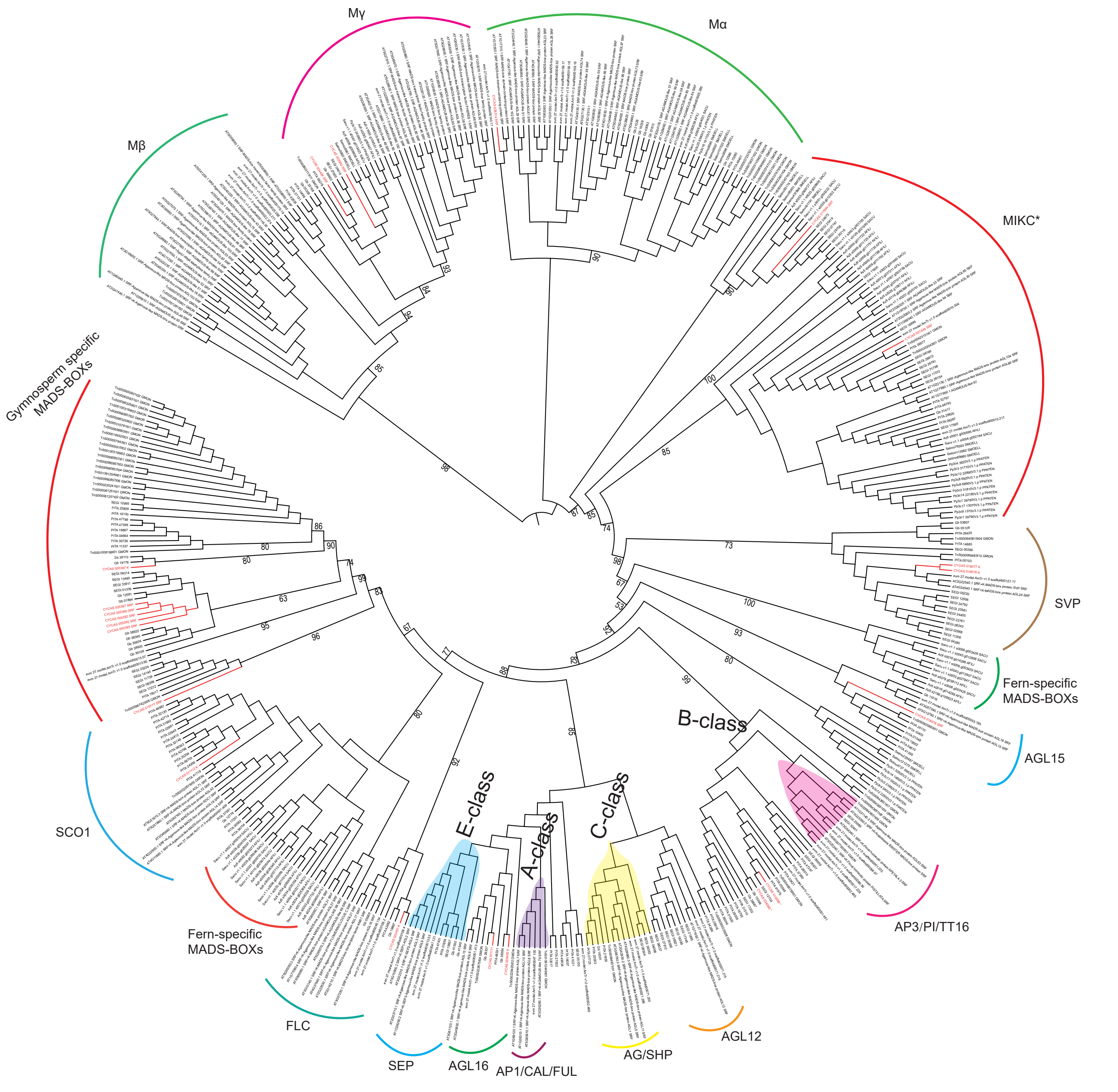

Supplementary Figure 26. Maximum-likelihood phylogenetic tree with bootstrap support of 5000 replicates of the MADS-BOX genes. The tree was built by IQ-TREE using the automatically selected best-fit model supports with ultrafast bootstrap approximation. The bootstrap values ≥ 50% are shown on the central branches.

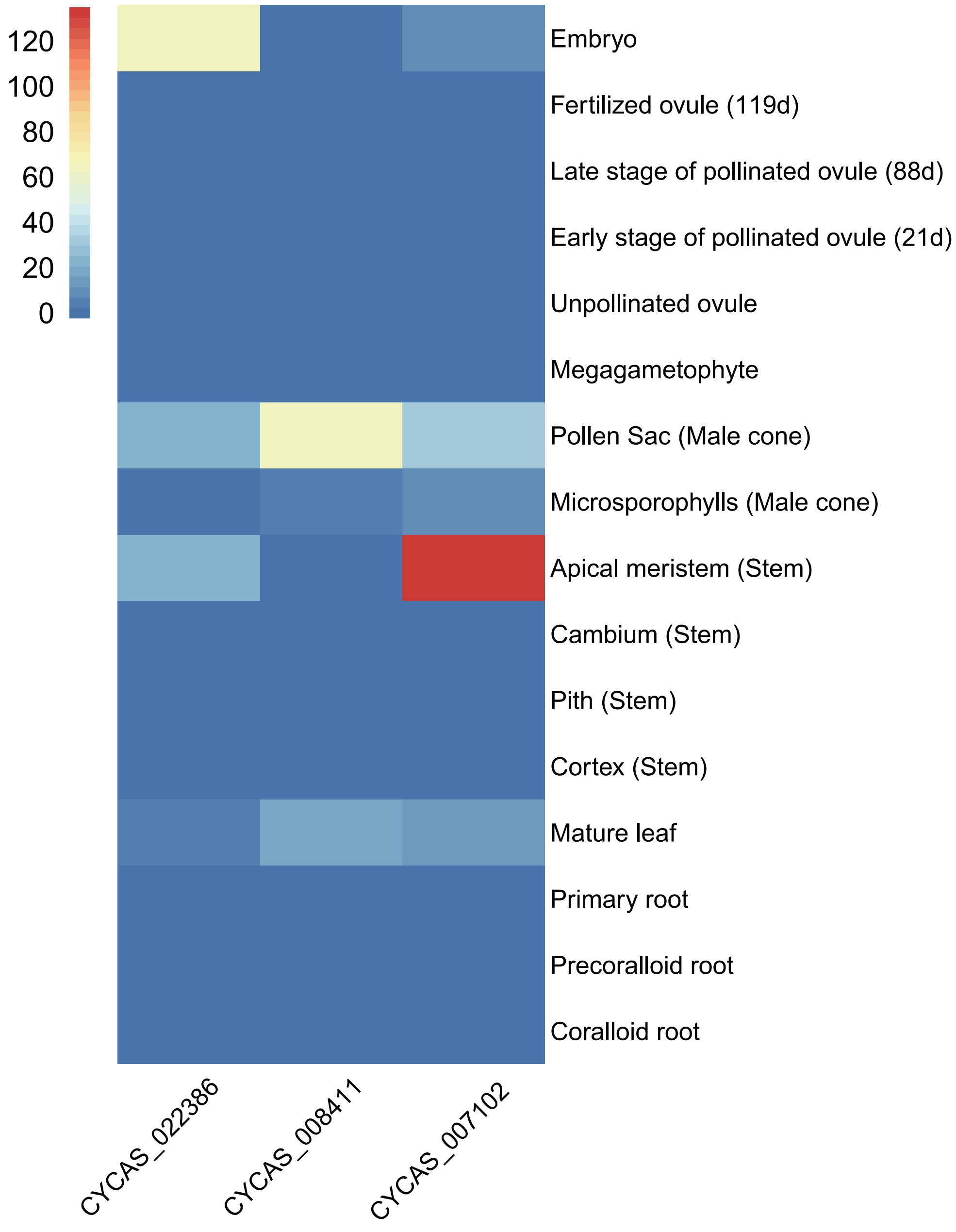

Supplementary Figure 27. Transcriptome Expression heatmap of YABBY transcripton factor in different tissues of *C. panzhihuaensis*. X-axis indicates the genes while the y-axis indicates the different tissues.

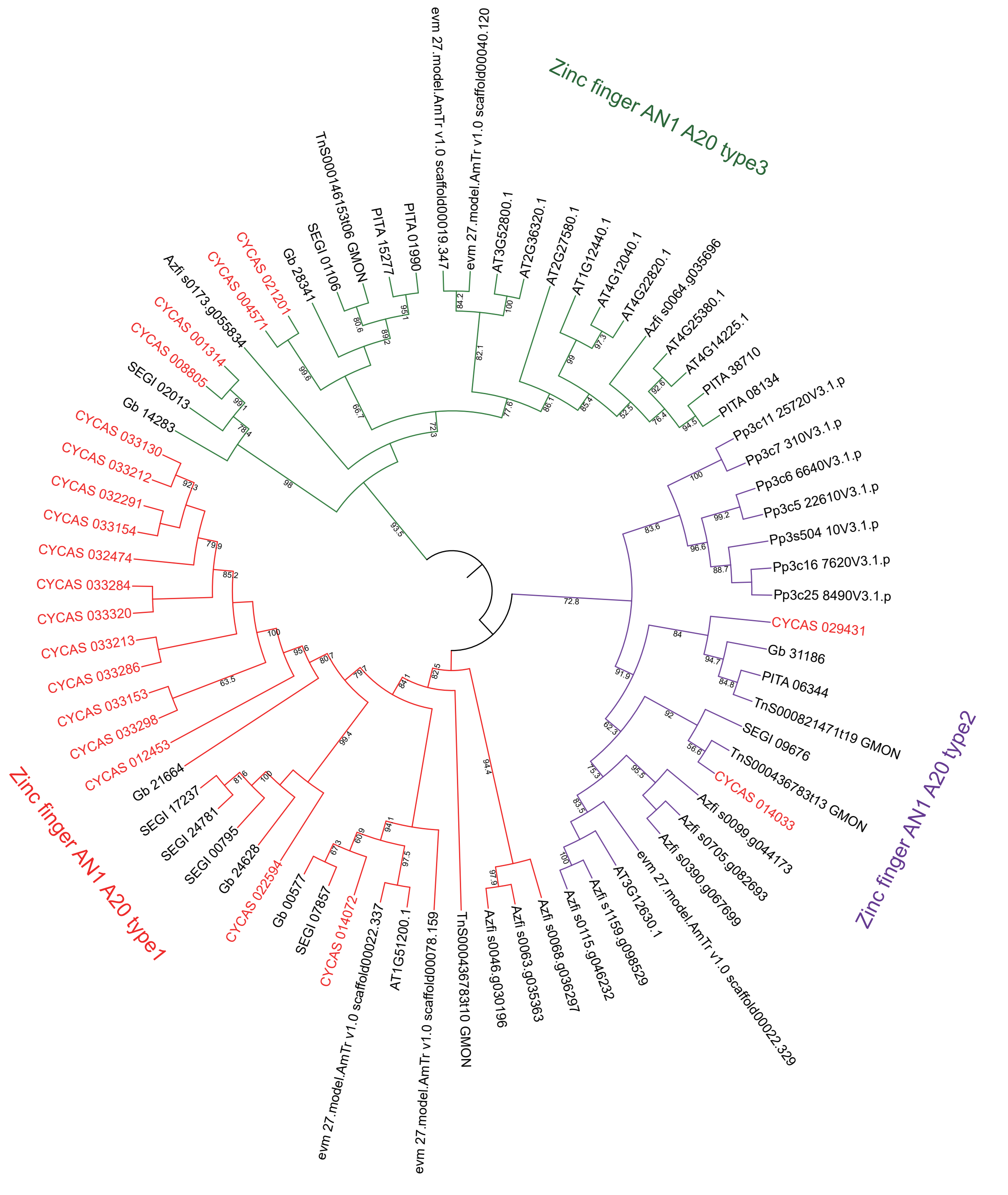

Supplementary Figure 28. Maximum-likelihood phylogenetic tree with bootstrap support of 5000 replicates of the Zinc fingers, AN1 and A20 type. The tree was built by IQ-TREE using the automatically selected best-fit model supports with ultrafast bootstrap approximation. The bootstrap values ≥ 50% are shown on the central branches.

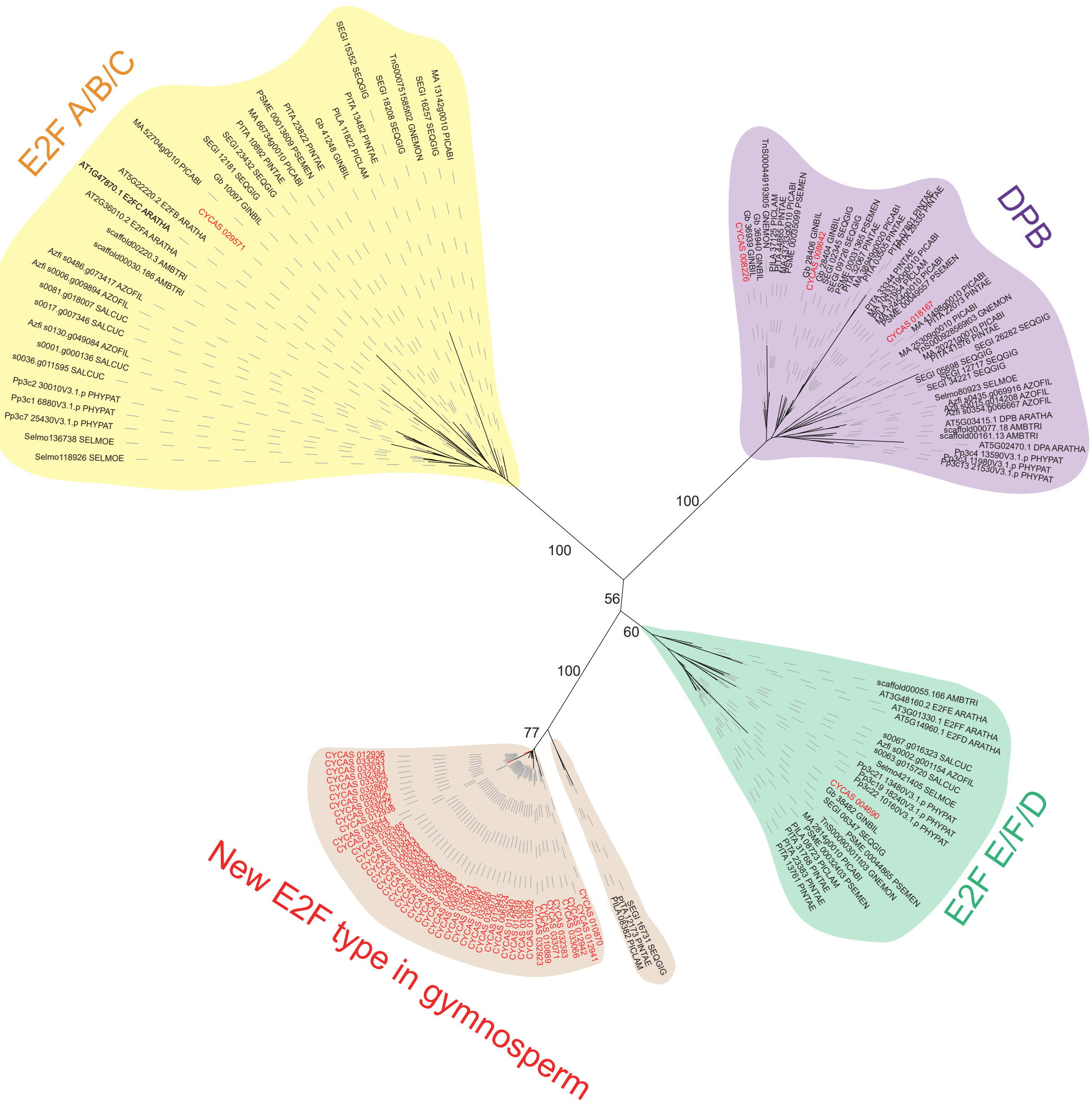

Supplementary Figure 29. Maximum-likelihood phylogenetic tree of E2F and DPB. The tree was built using RAxML (estimating branch support values by bootstrap iterations with 1000 replicates) with PROTGAMMAAUTO amino acid substitution model. The bootstrap values ≥ 50% are shown in the central branches. DBP, Dimerization partner protein.

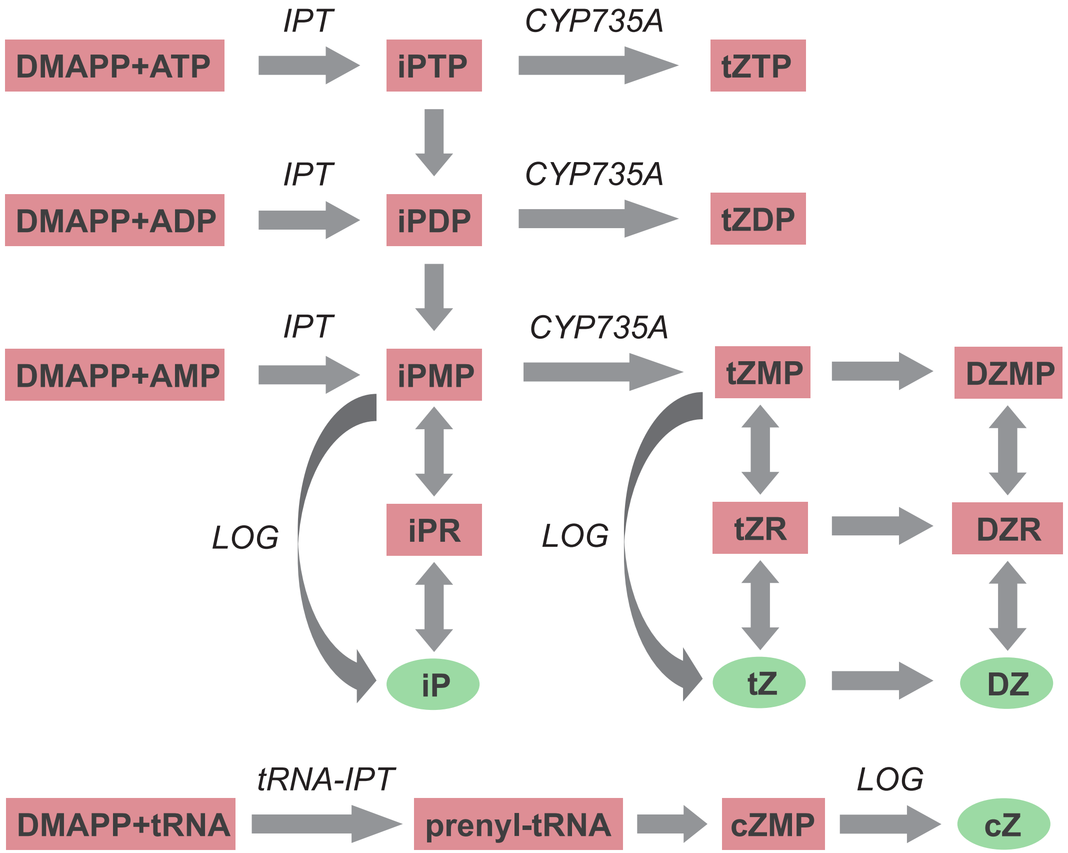

Supplementary Figure 30. The Cytokinins biosynthesis pathway of *C. panzhihuaensis*. The pink block represents intermediate products while the light green circle represents phytohormones. iP, Isopentenyladenine; tZ, transzeatin; DZ, dihydrozeatin; cZ, cis-zeatin; LOG, lonely guy; IPT, isopentenyl transferase; DMAPP, Dimethylallyl pyrophosphate; ADP, adenosine diphosphate; ATP, adenosine triphosphate; iPMP, isopentenyladenosine-5′-monophosphate; iPDP, isopentenyladenosine-5′-diphosphate; iPTP, isopentenyladenosine-5′-triphosphate; tZMP, trans-zeatin riboside-5′-monophosphate; tZDP, trans-zeatin riboside-5′-diphosphate; tZTP, trans-zeatin riboside-5′-triphosphate; tZR, trans-zeatin riboside; iPR, isopentenyladenine riboside.

S1: Ovule (Combining with 4 stages)
S2: Megagametophyte
S3: Cortex (Stem)
S4: Pollen Sac (Male cone)
S5: Apical meristem of (Stem)
S6: Microsporophylls (Male cone)
S7: Pith (Stem)
S8: Cambium (Stem)
S9: Primary root
S10: Mature leaf

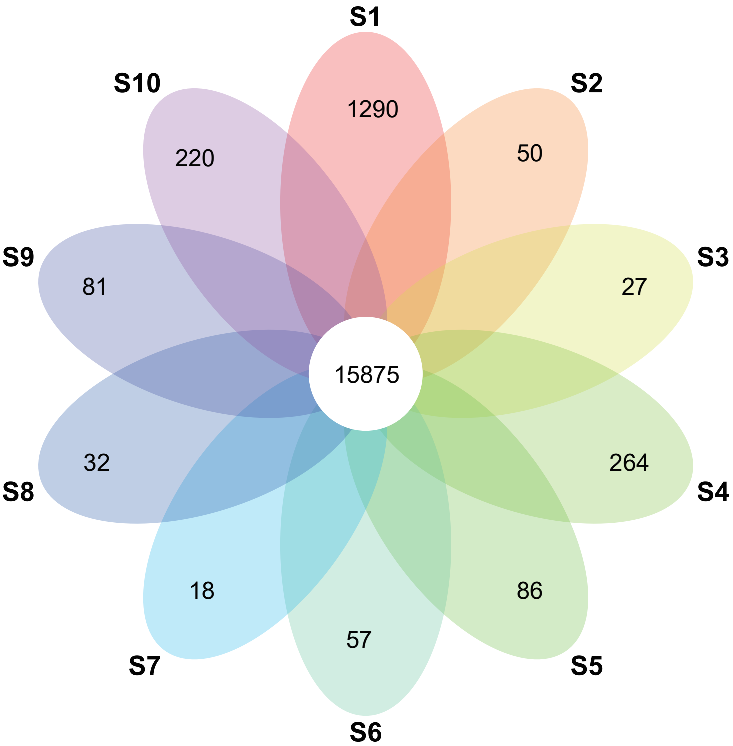

Supplementary Figure 31. Flower plot diagram showing the core and unique genes of different tissues in *C. panzhihuaensis*. The central circle shows the number of genes common to all tissues while the petals show the number of genes unique to each tissue.

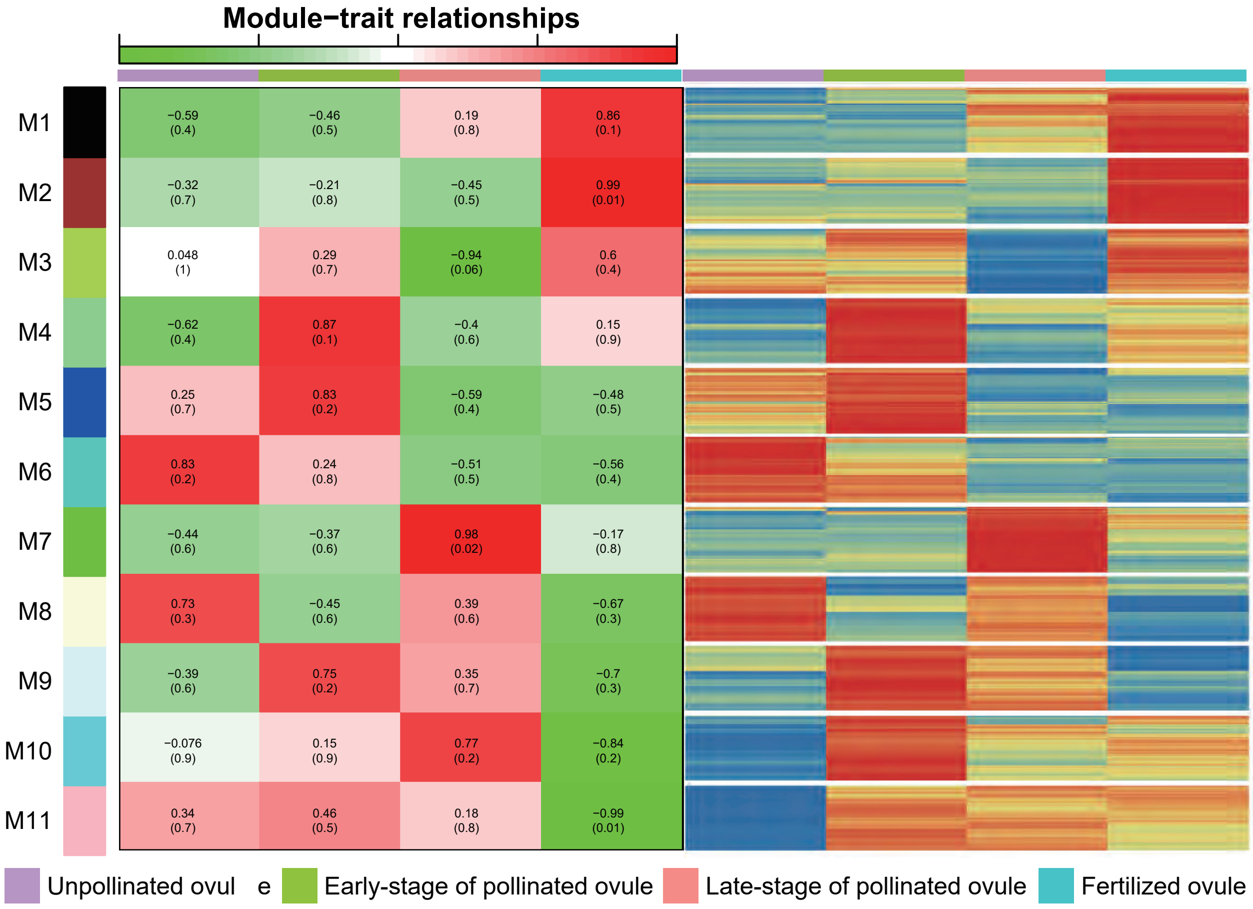

Supplementary Figure 32. WGCNA module identification and correlation analysis. The left hand panel represents the correlation of the identified modules of different seed development stages. Pearson correlation coefficient of each module with different tissues are given and colored according to the score. The right heatmap shows the relative expression level of different seed development stages.

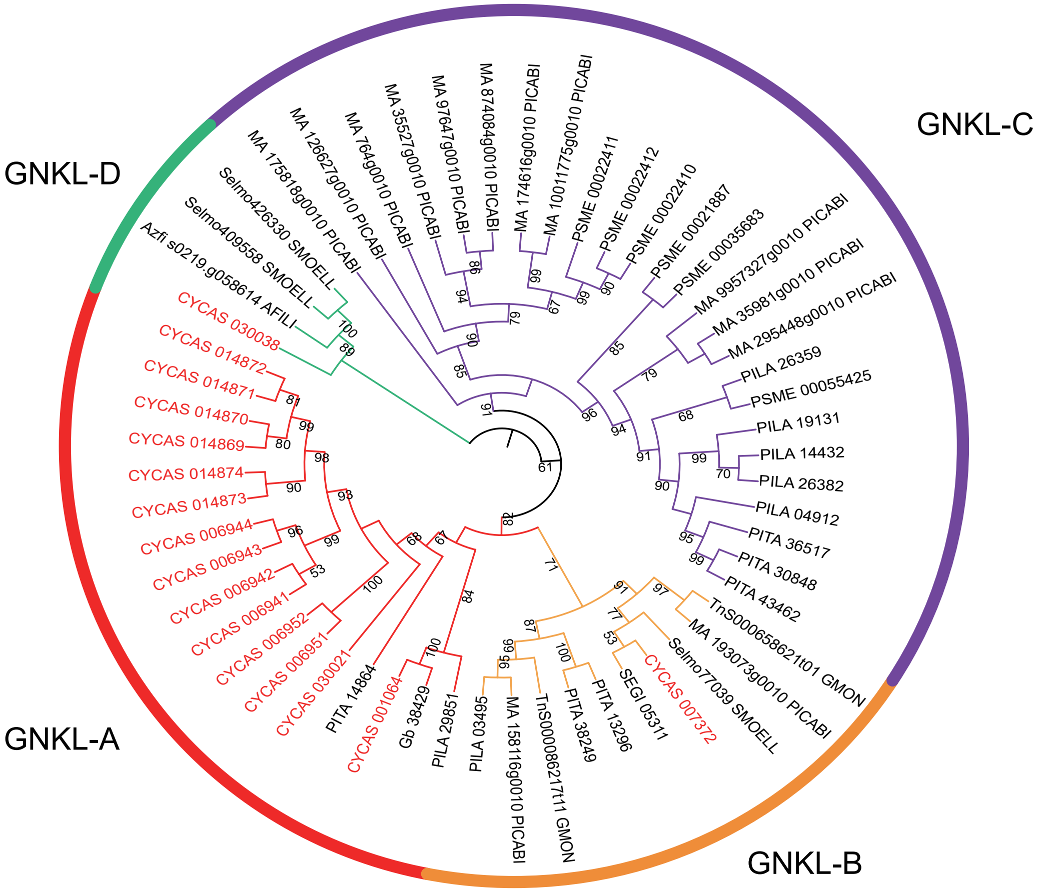

Supplementary Figure 33. Maximum-likelihood phylogeny of Ginkbilobin. The tree of antifungal protein ginkbilobin was built using RAxML (bootstrap support values shown on branches based on 500 replicates) with PROTGAMMAGTRX amino acid substitution model, the bootstrap values ≥ 50% are shown. Refer to the legend of extended Figure 4 for abbreviations of genes.

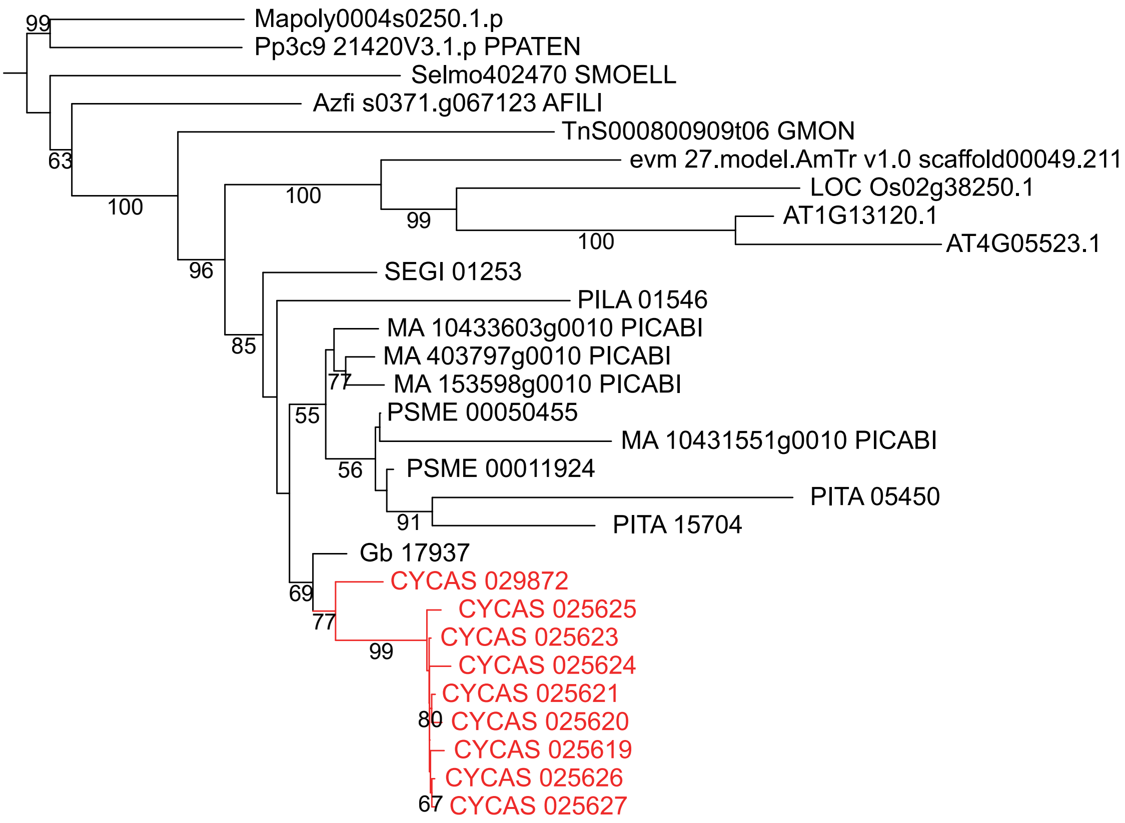

Supplementary Figure 34. Maximum-likelihood phylogeny of the Protein GLE1 homolog. The tree of GLE was built using RAxML (estimating branch support values by bootstrap iterations with 500 replicates) with PROTGAMMAGTRX amino acid substitution model, bootstrap values ≥ 50% are shown.

a

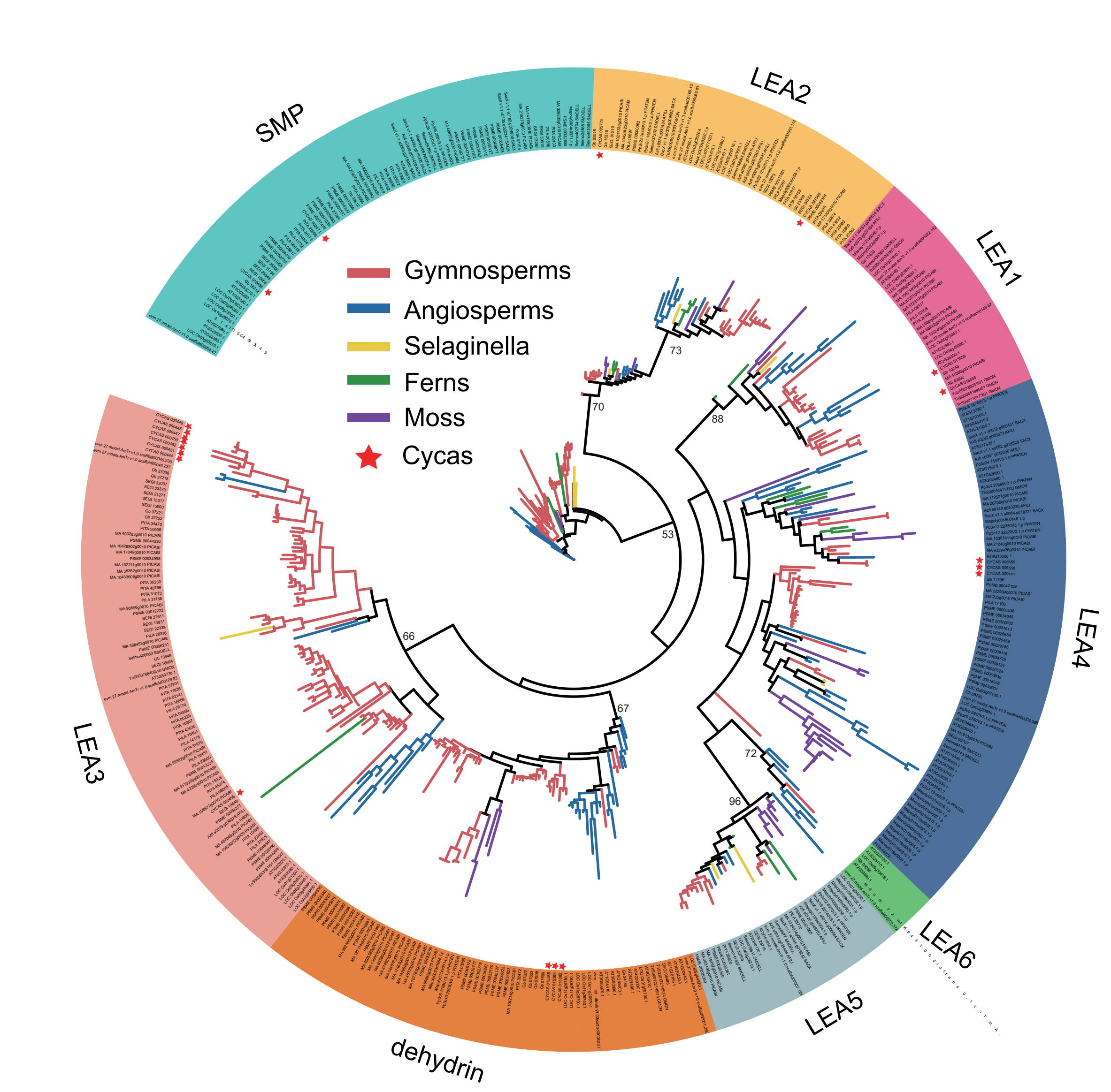

b

Group	A. thaliana	O. japonica	A. trichopoda	P. abies	P. taeda	P. lambertiana	P. menzesii	G. montanum	S. giganteum	G. biloba	C. panzhihuaensis	A. filiculoides	S. cucullata	S. moellendorffii	P. patens	M. polymorpha
LEA1	3	5	2	8	1	2	0	4	0	3	2	1	1	1	0	2
LEA2	3	5	3	3	7	3	3	1	3	3	2	4	1	2	3	2
LEA3	4	5	3	14	18	10	9	2	9	5	8	1	0	1	0	0
LEA4	18	2	1	8	1	1	15	1	15	2	3	3	3	2	8	13
LEA5	2	2	1	3	1	1	4	0	4	0	0	2	2	3	2	5
LEA6	3	1	2	0	0	0	0	0	0	1	0	0	0	0	0	0
dehydrin	10	7	2	13	2	0	20	2	20	5	3	0	0	0	2	2
SMP	6	6	1	6	12	6	19	0	19	2	2	1	4	4	2	2

Supplementary Figure 35. Inferred evolutionary history of LEA gene family. (a) The Phylogenetic tree was built using RAxML (bootstrap support values shown on branches based on 500 replicates) with PROTCATGTR amino acid substitution model. Bootstrap values ≥ 50% are shown on the central branches. (b) Statistics of LEA genes among representative species in embryophyta.

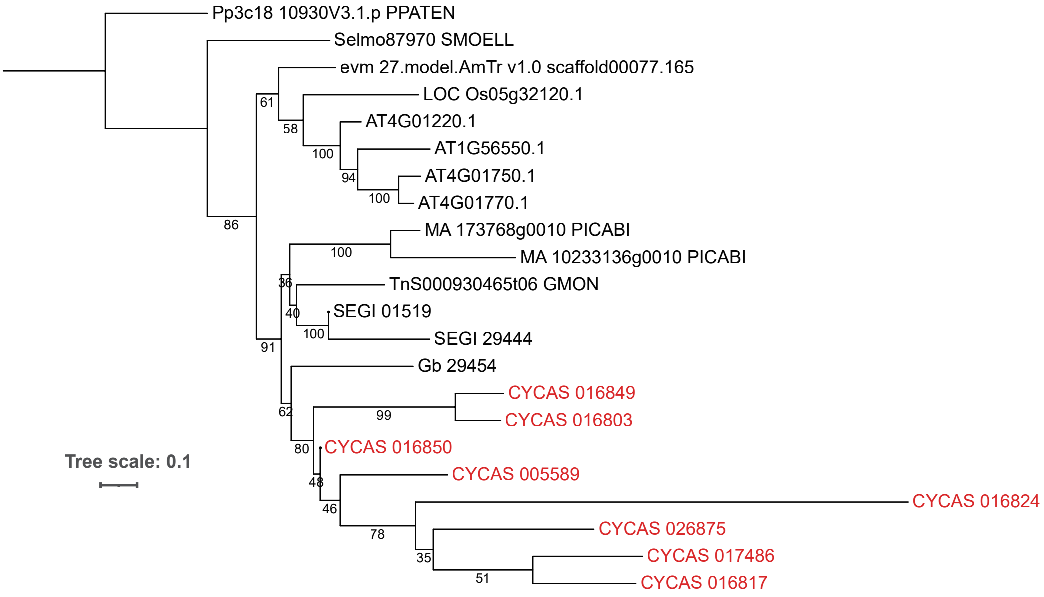

Supplementary Figure S36. The phylogenetic analysis of UDP-D-xylose:L-fucose alpha-1,3-D-xylosyltransferase gene family. The bootstrap values ≥ 50% are shown. The Maximum-likelihood tree was generated by RAxML with PROTCATGTR model and 500 bootstrap replicates.

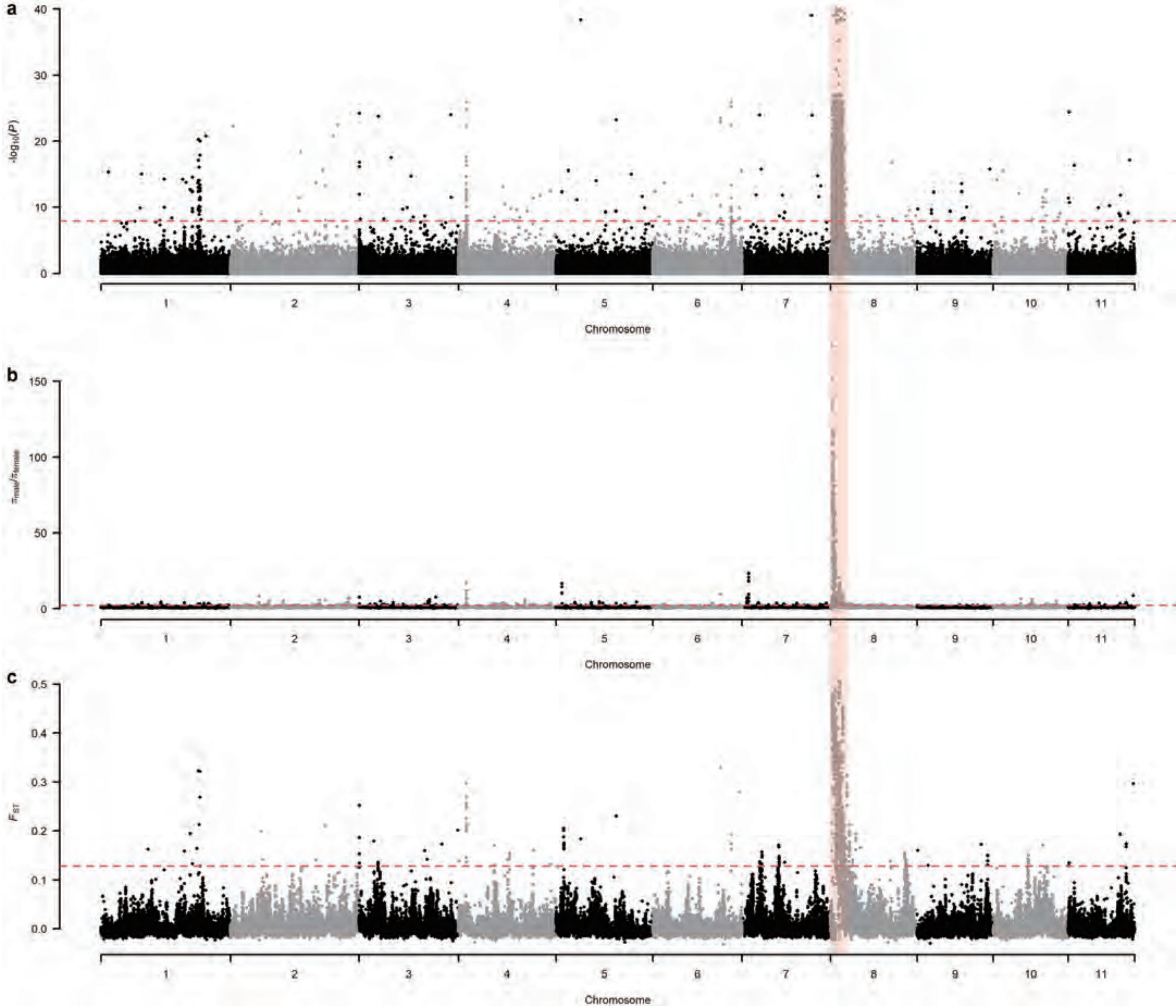

Supplementary Figure 37. Identification of sex differentiation region in the *C. panzhihuaensis* genome. a) Genome-wide association of sex in 31 male and 31 female individuals. The red horizontal dashed line represents the Bonferroni-corrected threshold for genome-wide significance (α = 0.05). b) Ratio of nucleotide diversity (π) in male and female *Cycas* population. The red horizontal dashed line represents the 95 percentile of the ratios. c) Differentiation index (FST) between male and female *Cycas* population. The red horizontal dashed line represents the 95 percentiles of the FST values. Orange rectangle represents the putative sex differentiation region on chromosome 8. P-values were calculated from a mixed linear model association of SNPs. The association analysis were performed once with a population of 31 male and 31 female individuals.

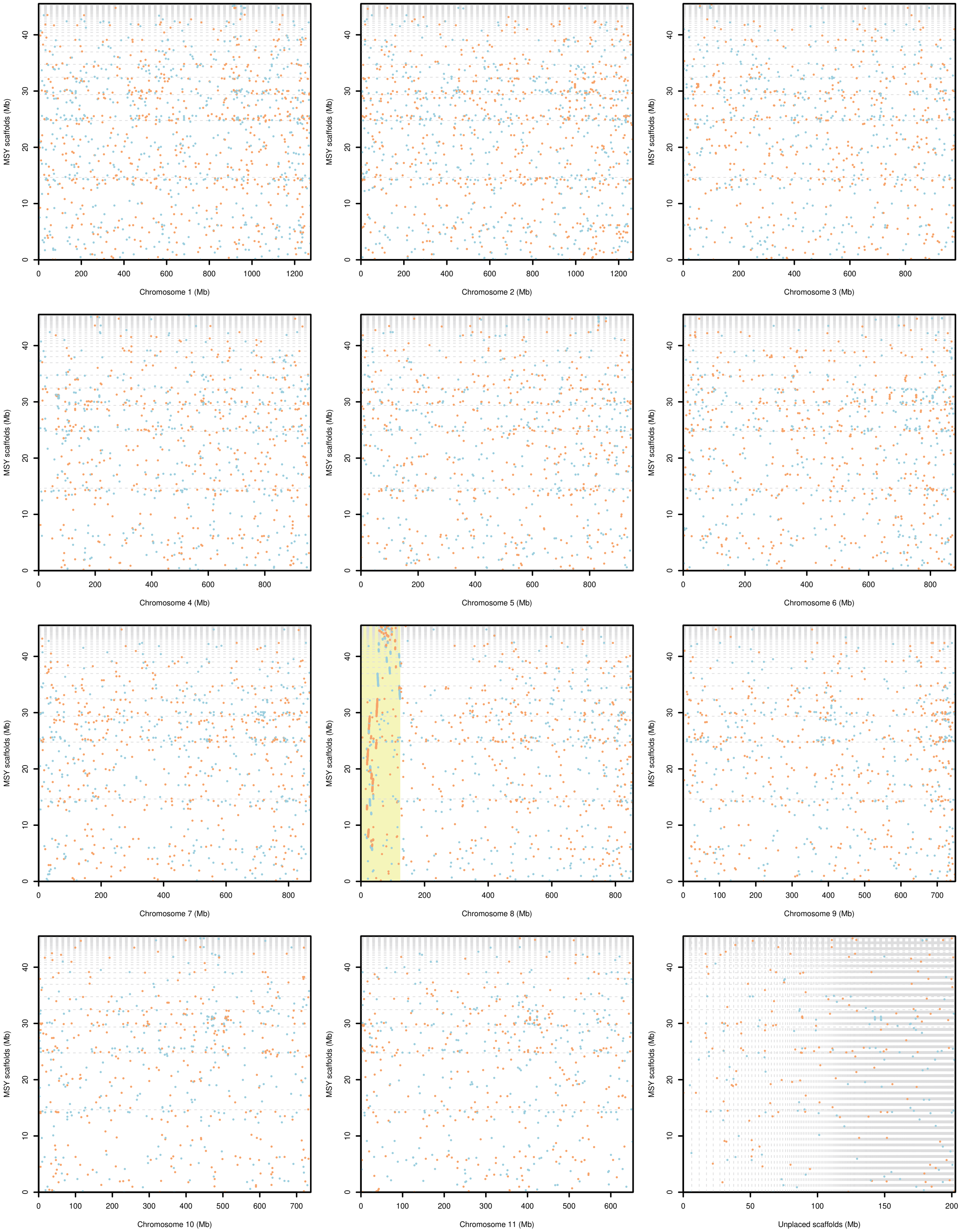

Supplementary Figure 38 Alignment of the MSY scaffolds and the *C. panzhihuaensis* genome. Scaffolds are separated by grey dashed lines. Red lines represent the alignments greater than 5 kb on the forward strand, and blue lines represent those on the reverse strand. The orange box indicates the sex differentiated region on chromosome 8.

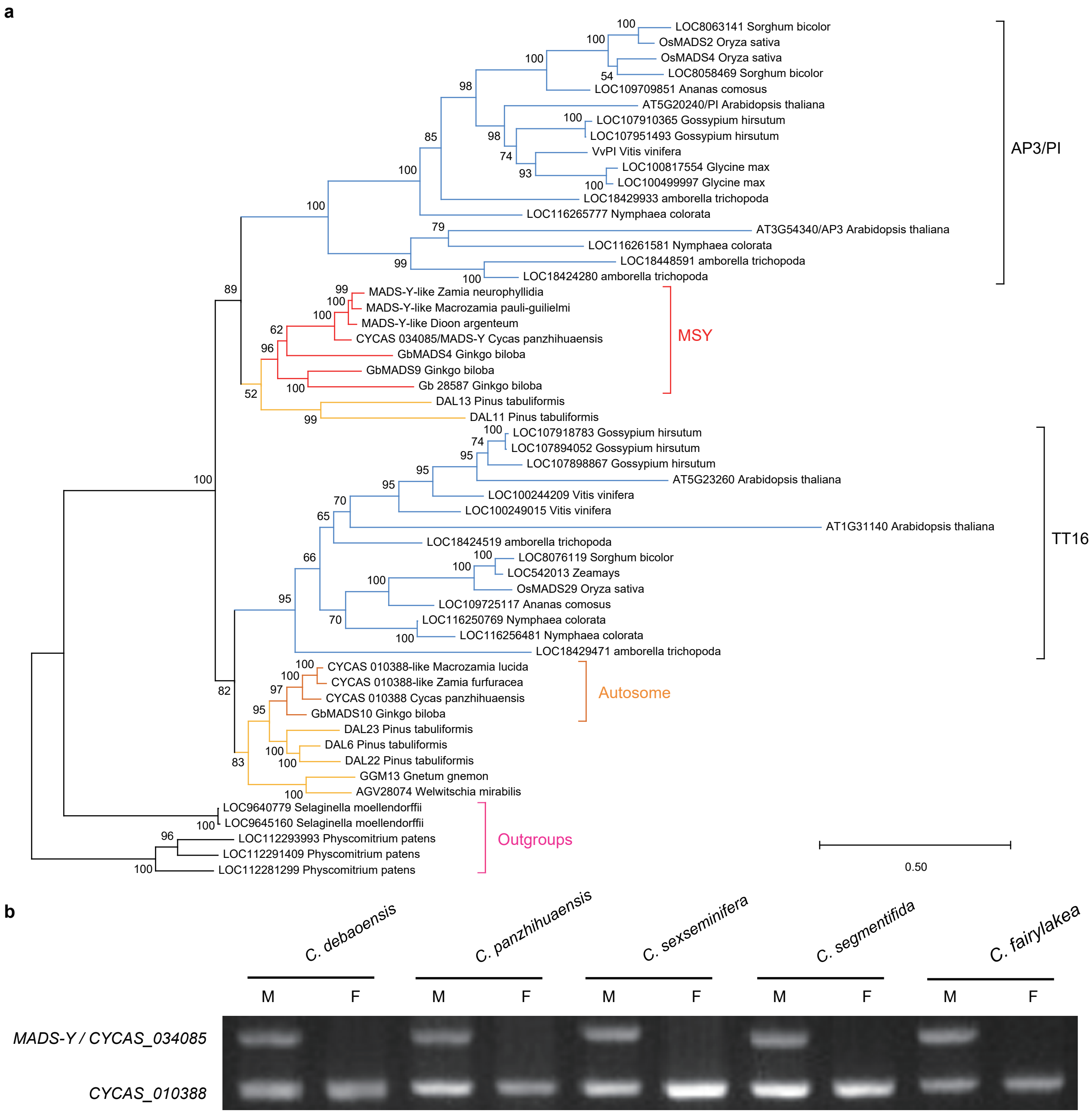

Supplementary Figure 39. Two MADS-box transcription factor genes differentially expressed in reproductive organs of *C. panzhihuaensis.* a) Phylogeny of CYCAS_034085 homologs across the green plants. The Maximum-likelihood tree generated by RAxML with PROTCATGTR model and 500 bootstrap replicates. b) Molecular genotyping of CYCAS_034085 and CYCAS_010388 in male (M) and female.

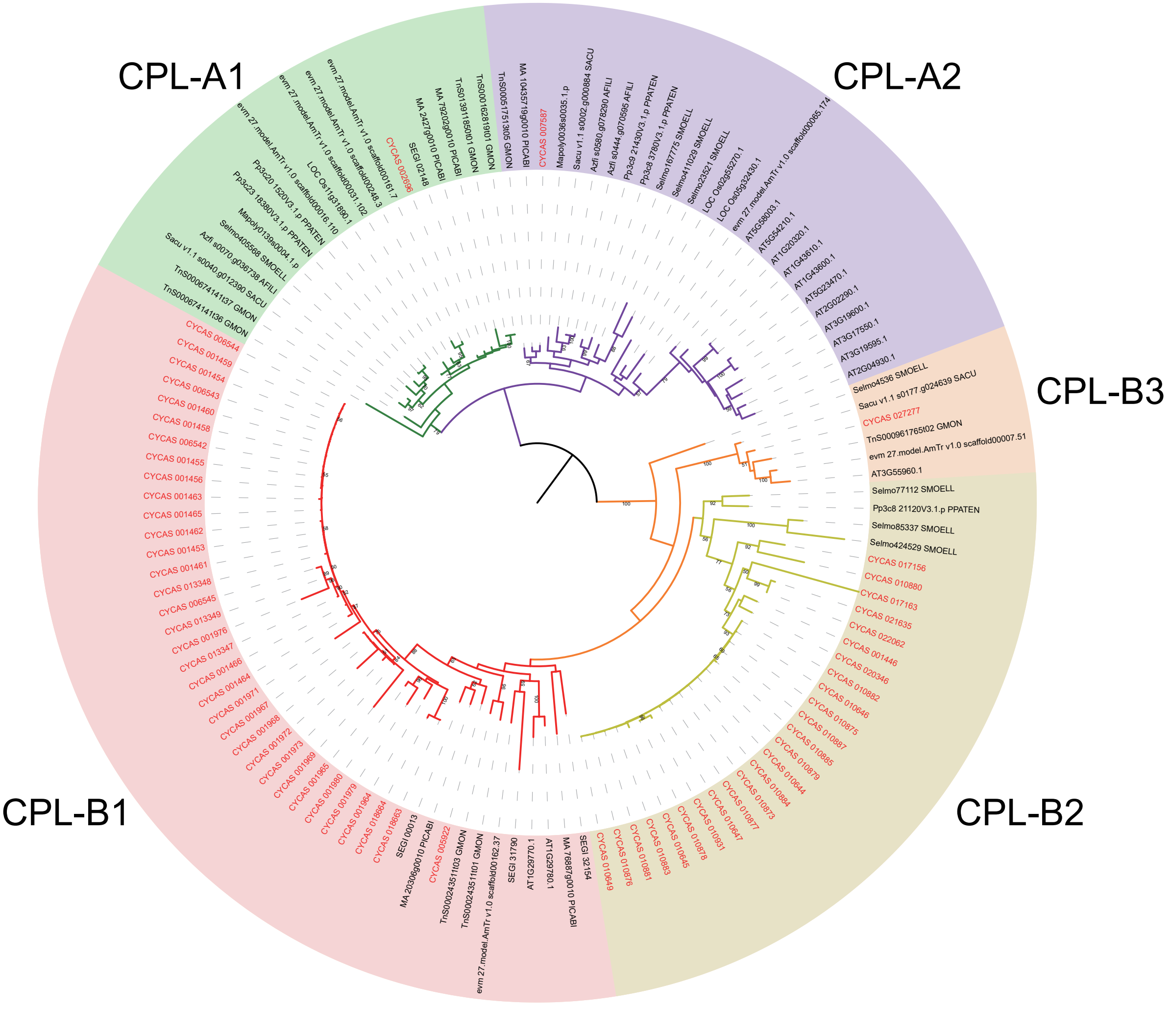

Supplementary Figure 40. Maximum-likelihood phylogeny of the C terminal domain phosphatase-lie family. The tree was built using RAxML (bootstrap support values shown on branches based on 500 replicates) with PROTGAMMAGTRX amino acid substitution model. Bootstrap values ≥ 50% are shown.

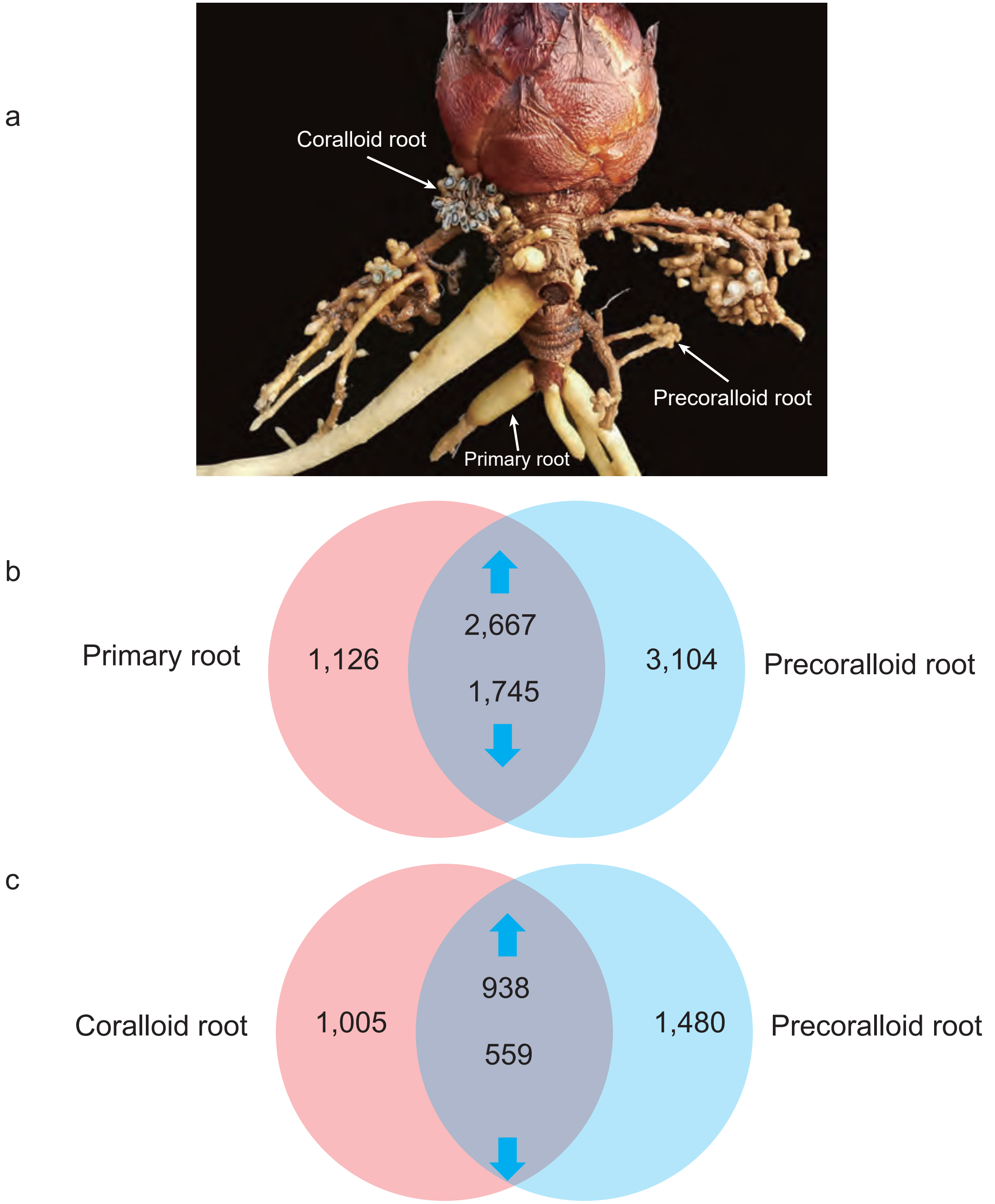

Supplementary Figure 41. Comparative transcriptomics analysis of three types of roots in *C. panzhihuaensis*. (a) Morphological characters of three different roots (Primary roots, coralloid roots and precoralloid roots). (b) Venn diagram of specific expressed genes of primary roots and precoralloid roots. (c) Venn diagram of specific expressed genes of coralloid roots and precoralloid roots.

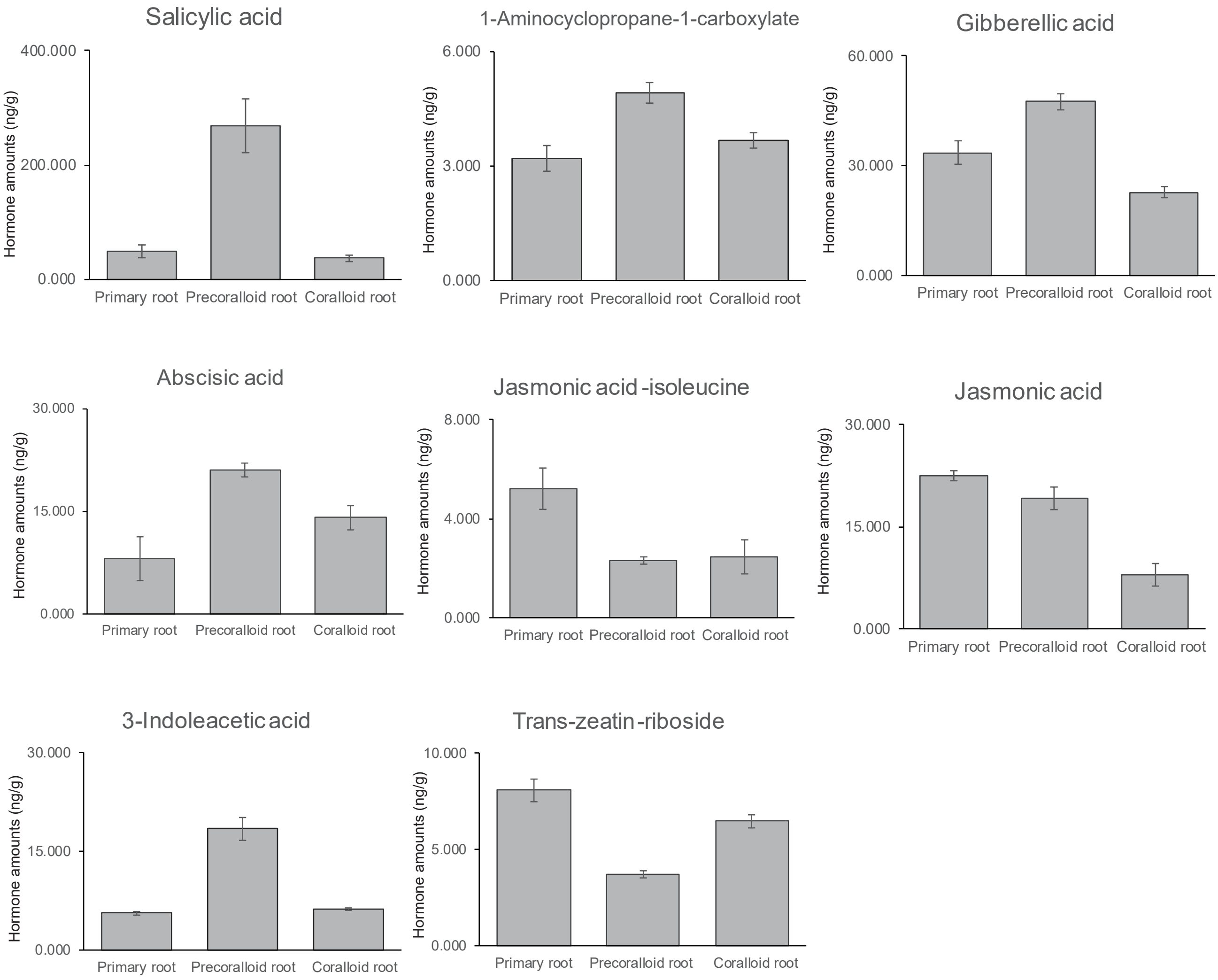

Supplementary Figure 42. Quantification of amounts of various plant hormone of three types of *C. panzhihuaensis* roots. The grey bar represents the amount of hormone among 3 different roots while the error bar represents the standard error (n=3 biologically independent experiments).

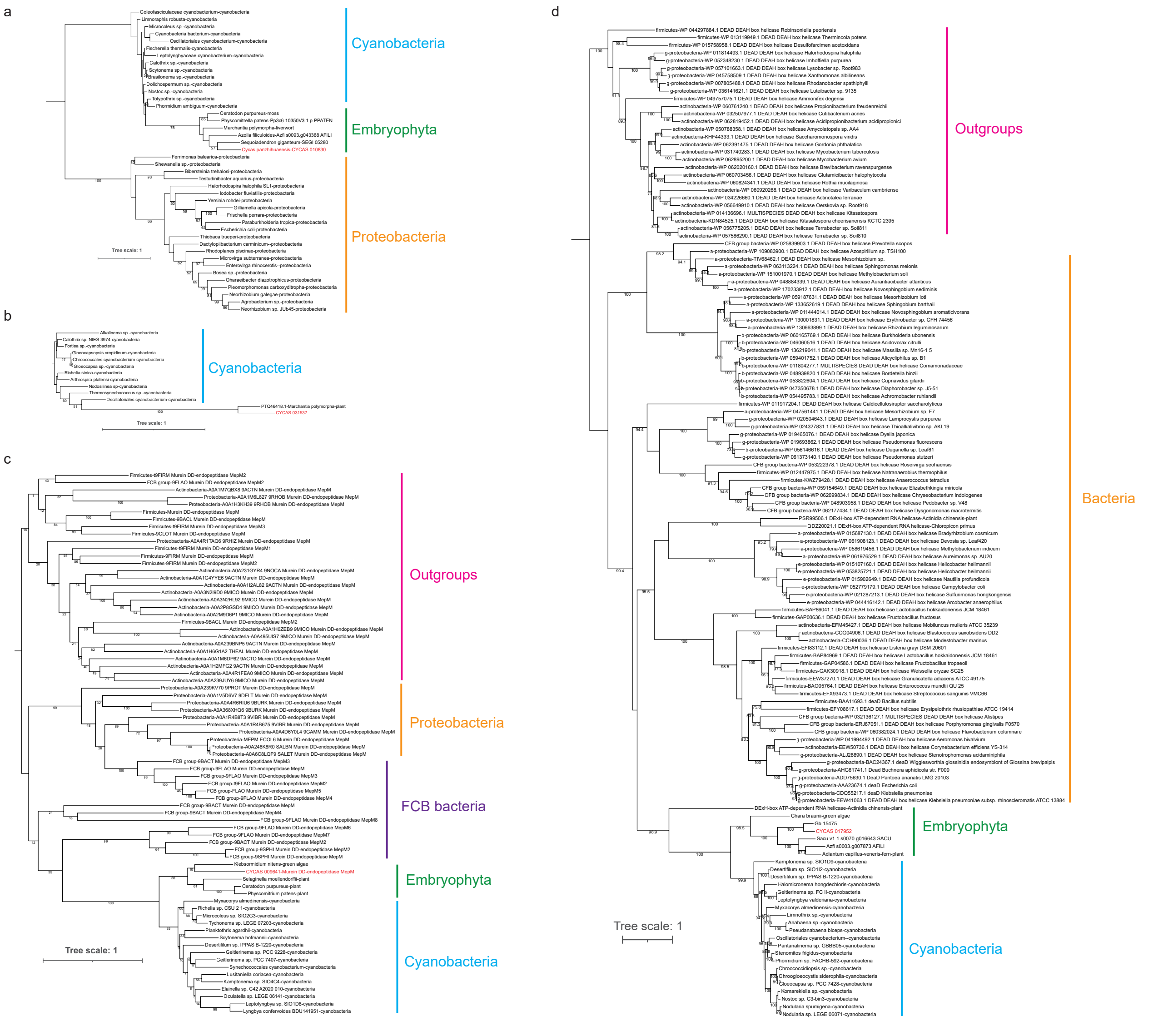

Supplementary Figure 43. Putative cyanobacteria derived genes in *C. panzhihuaensis*. (a) The phylogenetic tree of heme utilization protein HuvX. (b) The phylogenetic tree of DUF1825 family. (c) The phylogenetic tree of DEAD/DEAH box helicase. (d) The phylogenetic tree of peptidoglycan DD-metalloendopeptidase family protein. All trees in this analysis were built by RAxML (bootstrap support values shown on branches based on 500 replicates) with PROTCATGTR amino acid substitution model.

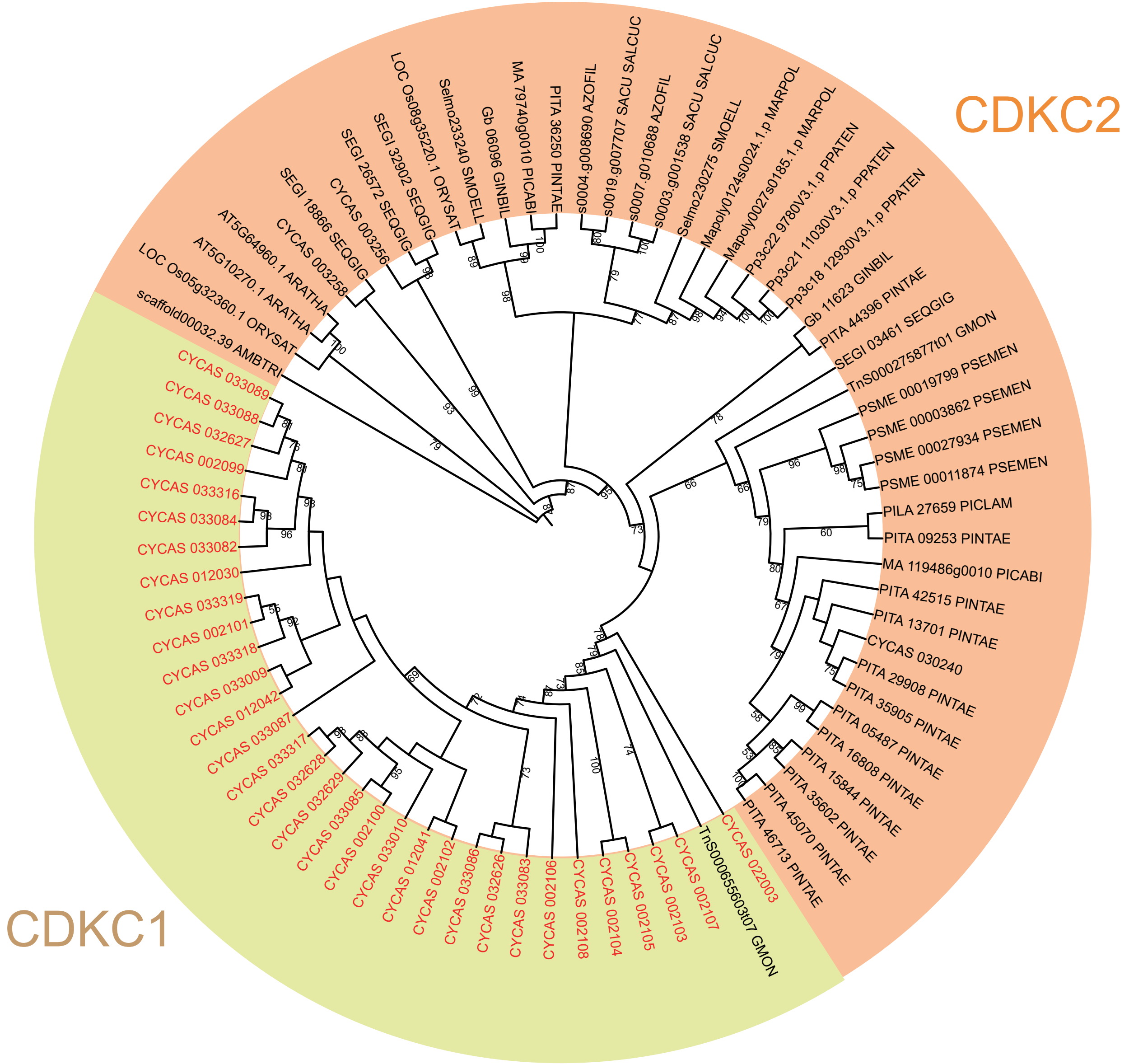

Supplementary Figure 44. Maximum-likelihood phylogeny of the CDKC gene family. Bootstrap values ≥ 50% are shown. The tree was built using RAxML (bootstrap support values shown on branches based on 500 replicates) with PROTCATGTR amino acid substitution model.
