# Supplementary material for: The Cycas genome and the early evolution of seed plants
Source: Nat Plants. 2022 Apr 18;8(4):389–401. doi: 10.1038/s41477-022-01129-7 (PMC9023351; doi:10.1038/s41477-022-01129-7)
Supplement: Supplementary file 2 — Reporting Summary [file 41477_2022_1129_MOESM2_ESM.pdf]

nature portfolio

Corresponding author(s): Shouzhou Zhang

Last updated by author(s): Feb 22, 2022

Reporting Summary

Nature Portfolio wishes to improve the reproducibility of the work that we publish. This form provides structure for consistency and transparency in reporting. For further information on Nature Portfolio policies, see our Editorial Policies and the Editorial Policy Checklist.

Statistics

For all statistical analyses, confirm that the following items are present in the figure legend, table legend, main text, or Methods section.

n/a	Confirmed	
☐	☒	The exact sample size (*n*) for each experimental group/condition, given as a discrete number and unit of measurement
☒	☐	A statement on whether measurements were taken from distinct samples or whether the same sample was measured repeatedly
☐	☒	The statistical test(s) used AND whether they are one- or two-sided<br>*Only common tests should be described solely by name; describe more complex techniques in the Methods section.*
☒	☐	A description of all covariates tested
☒	☐	A description of any assumptions or corrections, such as tests of normality and adjustment for multiple comparisons
☐	☒	A full description of the statistical parameters including central tendency (e.g. means) or other basic estimates (e.g. regression coefficient) AND variation (e.g. standard deviation) or associated estimates of uncertainty (e.g. confidence intervals)
☐	☒	For null hypothesis testing, the test statistic (e.g. *F*, *t*, *r*) with confidence intervals, effect sizes, degrees of freedom and *P* value noted<br>*Give P values as exact values whenever suitable.*
☒	☐	For Bayesian analysis, information on the choice of priors and Markov chain Monte Carlo settings
☒	☐	For hierarchical and complex designs, identification of the appropriate level for tests and full reporting of outcomes
☒	☐	Estimates of effect sizes (e.g. Cohen's *d*, Pearson's *r*), indicating how they were calculated

Our web collection on statistics for biologists contains articles on many of the points above.

Software and code

Policy information about availability of computer code

Data collection

The long read data were generated by the Nanopore PromethION sequencer, and the short read data were sequenced by the MGI-SEQ or Illumina HiSeq X platform, with 150-bp read length and 300-500 insert size.

Data analysis

The softwares used in this study are listed as follows:

wtdbg2 version2.5 (https://github.com/ruanjue/wtdbg2)
miniasm version 0.3 (https://github.com/lh3/miniasm)
NextDenovo version 2.3.0 (https://github.com/Nextomics/NextDenovo)
NextPolish version 1.2.4 (https://github.com/Nextomics/NextPolish)
3d-dna version 201008 (https://github.com/aidenlab/3d-dna)
Juicebox version 1.13.01 (https://github.com/aidenlab/Juicebox)
MITE-hunter version 11-2011 (http://target.iplantcollaborative.org/mite_hunter.html)
LTRharvest version 1.5.10 (http://genometools.org/)
BUSCO version 4.0.2 (https://busco.ezlab.org/)
RepeatModeler version 2.0.1 (http://repeatmasker.org/RepeatModeler/)
RepeatMasker version 4.0.7 (https://www.repeatmasker.org/)
LTRdigest version 1.5.10 (http://genometools.org/)
Genewise version 2 (https://www.ebi.ac.uk/Tools/psa/genewise/)
Hisat version 2.2.1 (http://www.ccb.jhu.edu/software/hisat/index.shtml)
Stringtie version 1.3.3b (https://ccb.jhu.edu/software/stringtie/)
AUGUSTUS version 3.2.3 (https://bioinf.uni-greifswald.de/augustus/)
EVidenceModeler version 1.1.1 (https://evidencemodeler.github.io/)
InterProScan version 5.30-69.0 (https://www.ebi.ac.uk/interpro/search/sequence/)
miRDeep2 version 0.1.3 (https://github.com/rajewsky-lab/mirdeep2)

TargetFinder2 (https://github.com/carringtonlab/TargetFinder)
IQ-TREE2 version 2.0.6 (http://www.iqtree.org/)
ASTRAL version 5.7.3 (https://github.com/smirarab/ASTRAL)
OrthoFinder version 2.3.11 (https://github.com/davidemms/OrthoFinder)
KinFin version 1.0.3 (https://github.com/DRL/kinfin)
TranslatorX version local (https://translatorx.org/)
STAG version 1.0.0 (https://github.com/bbenligiray/stag)
PHYPARTS version 0.0.1 (https://bitbucket.org/blackrim/phyparts/src/master/)
DISCOVISTA version 1.0 (https://github.com/esayyari/DiscoVista)
HybPiper version 1.3.1 (https://github.com/mossmatters/HybPiper)
NOVOPlasty version 4.3.1 (https://github.com/ndierckx/NOVOPlasty)
PHYLONET version 2.4 (https://bioinfocs.rice.edu/phylonet/index.html)
PHYBASE version 2.0 (https://github.com/lliu1871/phybase)
TWISST version 0.2 (https://github.com/simonhmartin/twisst)
TRINITY version 2.13.1 (https://github.com/trinityrnaseq/trinityrnaseq)
TRANSDECODER version 3.0.0 (https://github.com/TransDecoder/TransDecoder)
SORTADATE version 2018 (https://github.com/FePhyFoFum/SortaDate)
PAML version 4.9 (http://abacus.gene.ucl.ac.uk/software/paml.html)
MCMCTREE in PAML version 4.9 (http://abacus.gene.ucl.ac.uk/software/paml.html)
MCscanX (https://github.com/wyp1125/MCScanX)
MAFFT version 7 (https://mafft.cbrc.jp/alignment/software/linuxportable.html)
PAL2NAL version 14 (http://www.bork.embl.de/pal2nal/)
Partitionfinder version 2 (https://github.com/brettc/partitionfinder)
trimAl version 1.2 (https://github.com/inab/trimal)
RAxML version 8 (https://cme.h-its.org/exelixis/web/software/raxml/)
DupGen_finder version (https://github.com/qiao-xin/DupGen_finder)
SSK_finder version (https://github.com/BGI-Qingdao/SSK_finder)
Trimmonmatic version 0.38 (http://www.usadellab.org/cms/index.php?page=trimmomatic)
GVCFtyper version 201911 (https://www.sentieon.com/products/#dnaseq)
PLINK version 1.9 (https://github.com/chrchang/plink-ng/tree/master/2.0)
EMMAX version beta-07Mar2010 (https://github.com/topics/emmax)
GCTA version 1.91.4beta3 (https://gump.qimr.edu.au/gcta/)
VCFtools version 0.1.13 (https://vcftools.github.io/man_latest.html)
eXpress version 1.5.3 (https://github.com/adarob/eXpress)
DESeq2 version 1.34.0 (https://bioconductor.org/packages/release/bioc/html/DESeq2.html)
WGCNA (https://github.com/cran/WGCNA)
PHYLIP version 3.696 (https://csbf.stanford.edu/phylip/)
CLUMPP version 1.1.2 (http://rosenberglab.bioinformatics.med.umich.edu/clumpp.html)
FigTree version 1.4.3 (http://tree.bio.ed.ac.uk/software/figtree/)
ADMIXTURE version 1.3.0 (https://dalexander.github.io/admixture/)

For manuscripts utilizing custom algorithms or software that are central to the research but not yet described in published literature, software must be made available to editors and reviewers. We strongly encourage code deposition in a community repository (e.g. GitHub). See the Nature Portfolio guidelines for submitting code & software for further information.

Data

Policy information about availability of data

All manuscripts must include a data availability statement. This statement should provide the following information, where applicable:

- Accession codes, unique identifiers, or web links for publicly available datasets
- A description of any restrictions on data availability
- For clinical datasets or third party data, please ensure that the statement adheres to our policy

The genome and transcriptome data, genome assemblies, and annotations can be found at https://db.cngb.org/codeplot/datasets/public_dataset?id=PwRftGHfPs5qG3gE. The raw genomic, transcriptomic and HiC data generated in this study were deposited in the NCBI Sequence Read Archive (SRA, BioProject PRJNA734434), and the CNGB data center (https://db.cngb.org/) under project number CNP0001756.
Some known functional protein databases we used: Uniprot database (version 2021_01), KEGG (version 93.0), NCBI NR (version 20201015), KOG (version 20090331).

Field-specific reporting

Please select the one below that is the best fit for your research. If you are not sure, read the appropriate sections before making your selection.

☒ Life sciences ☐ Behavioural & social sciences ☐ Ecological, evolutionary & environmental sciences

For a reference copy of the document with all sections, see nature.com/documents/nr-reporting-summary-flat.pdf

Life sciences study design

All studies must disclose on these points even when the disclosure is negative.

Sample size	For genome sequencing, for each experiment, megagametophytes from a individual of Cycas panzhihuaensis was used. For genome and transcriptome short-read sequencing for phylogeny studies, one sample from a same individual for each taxon was used.
Data exclusions	The sequence reads with low quality are more likely derived from sequencing errors, and were thus excluded. To reduce the effect of sequencing error on assembly, we performed the quality control of raw data using Trimmonmatic (v. 0.38).
Replication	Since this is a genome sequencing project, no replication was applied for our genome sequencing experiment. For RNA-seq in gene expression studies and metabolite measurements, three pr two biological replicates were applied.
Randomization	Since this is a genome sequencing project. The data were generated from a single individual, no randomizations were required.
Blinding	Since this is a genome sequencing project. The data were generated from a single individual, no blinding experiment was performed.

Reporting for specific materials, systems and methods

We require information from authors about some types of materials, experimental systems and methods used in many studies. Here, indicate whether each material, system or method listed is relevant to your study. If you are not sure if a list item applies to your research, read the appropriate section before selecting a response.

Materials & experimental systems

n/a	Involved in the study
☒	☐ Antibodies
☒	☐ Eukaryotic cell lines
☒	☐ Palaeontology and archaeology
☒	☐ Animals and other organisms
☒	☐ Human research participants
☒	☐ Clinical data
☒	☐ Dual use research of concern

Methods

n/a	Involved in the study
☒	☐ ChIP-seq
☒	☐ Flow cytometry
☒	☐ MRI-based neuroimaging
