# Supplementary figures and images for: The Cycas genome and the early evolution of seed plants
Source: Nat Plants. 2022 Apr 18;8(4):389–401. doi: 10.1038/s41477-022-01129-7 (PMC9023351; doi:10.1038/s41477-022-01129-7)

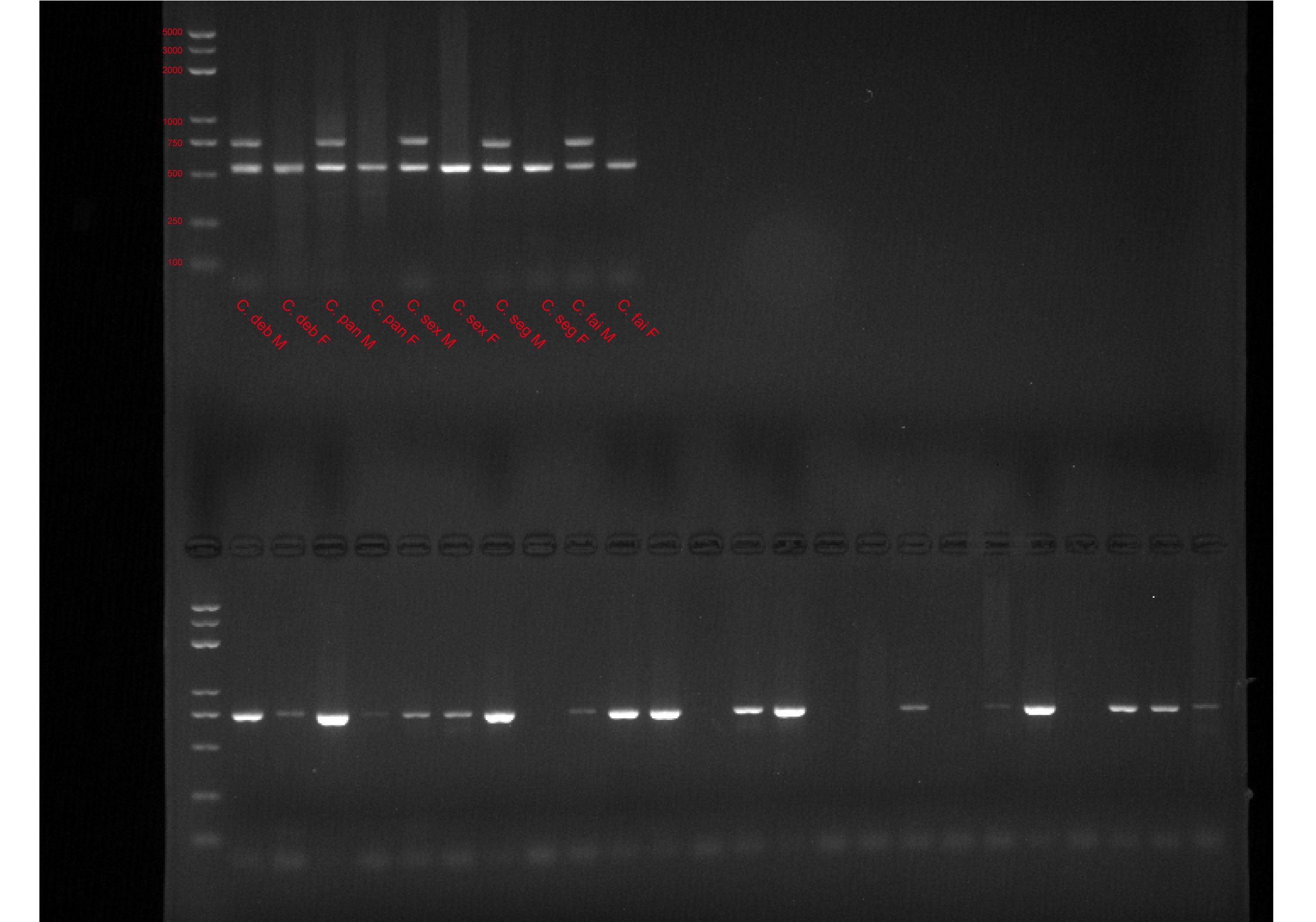

Supplement: Supplementary file 5 — Raw gels figure for Supplementary Fig. 39. [file 41477_2022_1129_MOESM5_ESM.tif]

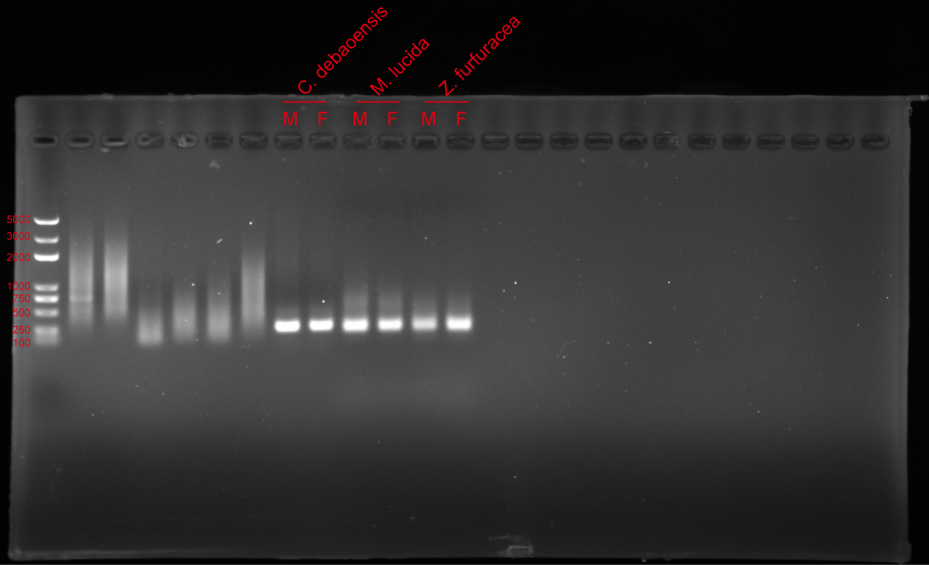

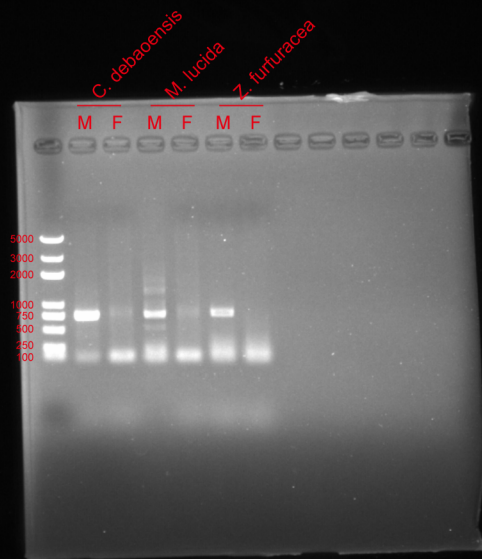

Supplement: Supplementary file 6 — Two unprocessed gel figures of Fig. 4 [file 41477_2022_1129_MOESM6_ESM.pdf]
